# Structure–Activity Relationship Study of 3-Alkynyl-6-aryl-isothiazolo[4,3-*b*]pyridines as Dual Inhibitors of the Lipid Kinases PIKfyve and PIP4K2C

**DOI:** 10.3390/ph18091341

**Published:** 2025-09-06

**Authors:** Demian Kalebic, Ling-Jie Gao, Belén Martinez-Gualda, Marwah Karim, Sirle Saul, Do Hoang Nhu Tran, Jef Rozenski, Leentje Persoons, Dominique Schols, Wim Dehaen, Shirit Einav, Steven De Jonghe

**Affiliations:** 1 Sustainable Chemistry for Metals and Molecules, Department of Chemistry, KU Leuven, Celestijnenlaan 200F, 3001 Leuven, Belgium; demian.kalebic@gmail.com (D.K.); wim.dehaen@kuleuven.be (W.D.); 2 Laboratory of Medicinal Chemistry, Department of Pharmaceutical and Pharmacological Sciences, Rega Institute for Medical Research, KU Leuven, Herestraat 49, Box 1030, 3000 Leuven, Belgium; ling-jie.gao@kuleuven.be (L.-J.G.); belen.mar.gual@gmail.com (B.M.-G.); jef.rozenski@kuleuven.be (J.R.); 3 Division of Infectious Diseases and Geographic Medicine, Department of Medicine, Stanford University School of Medicine, Stanford, CA 94305, USA; mkarim@stanford.edu (M.K.); sirlesaul@gmail.com (S.S.); dotran@stanford.edu (D.H.N.T.); seinav@stanford.edu (S.E.); 4 Molecular Genetics and Therapeutics in Virology and Oncology Research Group, Department of Microbiology, Immunology and Transplantation, Rega Institute for Medical Research, KU Leuven, Herestraat 49, Box 1043, 3000 Leuven, Belgium; leentje.persoons@kuleuven.be; 5 Molecular, Structural and Translational Virology Research Group, Department of Microbiology, Immunology and Transplantation, Rega Institute for Medical Research, KU Leuven, Herestraat 49, Box 1043, 3000 Leuven, Belgium; dominique.schols@kuleuven.be; 6 Department of Microbiology and Immunology, Stanford University School of Medicine, Stanford, CA 94305, USA; 7 Chan Zuckerberg Biohub, San Francisco, CA 94305, USA

**Keywords:** isothiazolo[4,3-*b*]pyridine, PIKfyve, PI5PI4Kγ, PIP4K2C, lipid kinase inhibitors, broad-spectrum antivirals, emerging viral infections

## Abstract

**Background/Objectives**: RMC-113, a 3-alkynyl-6-aryl-disubstituted isothiazolo[4,3-*b*]pyridine, is a dual inhibitor of the lipid kinases PIKfyve and PIP4K2C with broad-spectrum antiviral activity. The aim was to study the structure–activity relationship (SAR) of isothiazolo[4,3-*b*]pyridines as dual PIKfyve/PIP4K2C inhibitors. **Methods**: A series of isothiazolo[4,3-*b*]pyridines was synthesized by introducing structural variety at positions 3 and 6 of the central scaffold. The primary assay to guide the synthetic chemistry was a biochemical PIKfyve assay, with a number of analogues also tested for PIP4K2C binding affinity. Finally, isothiazolo[4,3-*b*]pyridines were also evaluated for antiviral and antitumoral activity in cell-based assays. **Results**: PIKfyve inhibition tolerated a wide variety of substituents on the aryl ring at position 6 of the isothiazolo[4,3-*b*]pyridine scaffold, with the 4-carboxamide analogue emerging as the most potent (IC_50_ = 1 nM). The SAR at position 3 was more restricted, although the introduction of electron-donating groups (such as a methyl and methoxy) on the pyridinyl ring yielded potent PIKfyve inhibitors, with IC_50_ values in the low nM range. The acetylenic moiety was essential for PIKfyve inhibition, and only the saturated ethyl linker displayed potent PIKfyve inhibition, albeit less active than the acetylene counterpart. The compounds were 2- to 5-fold less potent on PIP4K2C relative to PIKfyve. These dual PIKfyve/PIP4K2C inhibitors displayed antiviral activity against both the venezuelan equine encephalitis virus (VEEV) and the severe acute respiratory syndrome coronavirus 2 (SARS-CoV-2). A screening against a panel of cancer cell lines revealed antitumoral activity, although some of the potent PIKfyve/PIP5K2C inhibitors lacked antitumoral activity. **Conclusions**: Isothiazolo[4,3-*b*]pyridines are dual PIKfyve/PIP4K2C inhibitors displaying broad-spectrum antiviral, as well as antitumoral, activity.

## 1. Introduction

PIKfyve (named so after its function and domain structure phospho-inositide kinase for five position containing a Fyve finger) is a lipid kinase that phosphorylates the hydroxyl group at position 5 of the inositol ring of phosphatidylinositol-3-phosphate (PI(3)P), thereby yielding PI(3,5)P_2_ [1]. PI(3,5)P_2_ is involved in intracellular trafficking and lysosomal acidification and is thus crucial for the regulation of autophagy [2]. Hence, PIKfyve is involved in the regulation of endomembrane homeostasis and affects several aspects of endosome processing in the course of endocytic cargo transport [3]. PI(3,5)P_2_ is the biochemical precursor for PI(5)P, which is generated via dephosphorylation catalysed by a 3-phosphatase. It has been linked to intracellular membrane trafficking, autophagy regulation, peroxisome function and cholesterol homeostasis [4].

**Figure 1:**
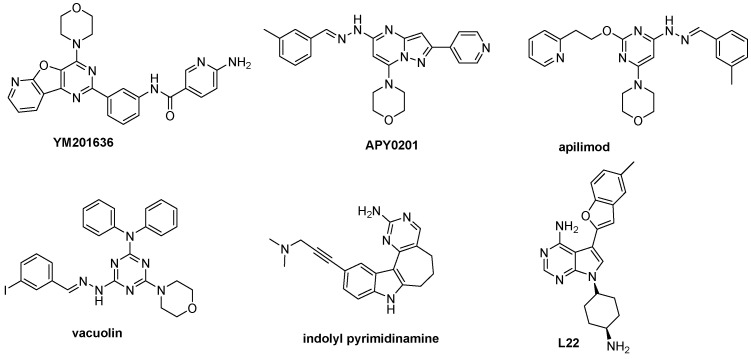
Existing PIKfyve inhibitors.

Several small molecule inhibitors of PIKfyve are known (Figure 1). YM201636 was the first reported inhibitor [5], with an IC_50_ of 33 nM. Since then, several other PIKfyve inhibitors, such as APY0201 [6], apilimod [7] and vacuolin-1 [8] have been described. More recently, different chemotypes were reported as PIKfyve inhibitors. For example, a series of indolyl pyrimidinamines [9] has potent PIKfyve activity with excellent kinome-wide selectivity. Pyrrolo[2,3-*d*]pyrimidine L22 is another very potent PIKfyve inhibitor (*K*_D_ = 0.47 nM) [10].

Recent studies indicate the potential of PIKfyve as therapeutic target in multiple disease areas, including viral infections, neurodegenerative disorders and cancers [11]. Apilimod display a strong antiproliferative effect in a large panel of non-Hodgkin lymphoma (NHL) B-cell lines, displaying IC_50_ values of less than 200 nM [12]. Oral administration of apilimod inhibits the growth of subcutaneous Burkitt lymphoma xenografts in mice. Apilimod is currently being evaluated in clinical trials for patients with B-cell malignancies [12]. Perturbation of endosomal trafficking is the mechanism by which PIKfyve inhibitors suppress the replication of various viruses, including Ebola virus [13], Marburg virus [14] and severe acute respiratory syndrome coronavirus 2 (SARS-CoV-2) [9,13]. Lastly, PIKfyve inhibition was shown to reduce both the trafficking of tau seeds into lysosomes and the induction of tau aggregation and to promote secretion of neurotoxic aggregates in Amyotrophic Lateral Sclerosis (ALS) patient iPSC-derived induced motor neurons, proposing a candidate strategy for the treatment of tauopathies [15,16].

**Figure 2:**
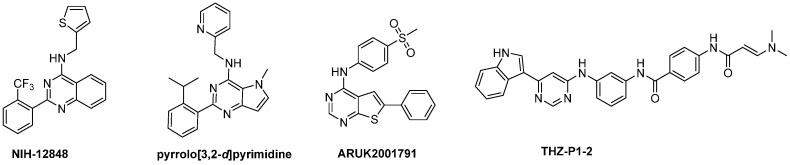
Reported PIP4K2C inhibitors.

PI-5-phosphate 4-kinases (PI5P4Ks) phosphorylate position 4 of PI-5-P to make PI(4,5)P_2_, which is an important precursor for second messengers inositol-1,4,5-triphosphate, diacylgycerol and phosphatidylinositol-3,4,5-trisphosphate [17]. In mammals, the PI5P4K family consists of three isoforms (α, β and γ), and the genes encoding the PI5P4Ks are called PIP4K2A, PIP4K2B and PIP4K2C. At the protein level, PIP4K2A and PIP4K2B are 83% identical, whereas PIP4K2C is approximately 60% identical to PIP4K2A and PIP4K2B. PIP4K2A is the most catalytically active, displaying significantly more activity compared to PIP4K2B, whereas PIP4K2C has very little inherent activity [18]. PIP4K2’s are important for cancer cell proliferation [19] and it is generally accepted that the PIP4K2’s are oncogenic. This spurred the search for small molecule inhibitors (Figure 2). NIH-12848 is a selective PIP4K2C inhibitor, with only moderate affinity (*K*_D_ = 2.8 µM) [20]. Subsequent optimization yielded a pyrrolo[3,2-*d*]pyrimidine analogue with stronger affinity for PIP4K2C (*K*_D_ = 68 nM) [21]. A series of thieno[2,3-*d*]pyrimidines (e.g., ARUK2001791) was recently disclosed that display low nM PIP4K2C potency, excellent kinase selectivity (including versus the other PIP4K2 isoforms) and appropriate pharmacokinetic properties to be used for in vivo experiments [22]. THZ-P1-2 is a covalent pan-PIP4K2 inhibitor, with PIP4K2C being the main target (*K*_D_ = 4.8 nM) and lower activity on PIP4K2A and PIP4K2B (IC_50_ values of 0.95 µM and 5.9 µM, respectively) [23]. Selective small molecule PIP4K2C inhibitors have not been studied in depth for their antiviral and/or antitumoral properties.

**Figure 3:**
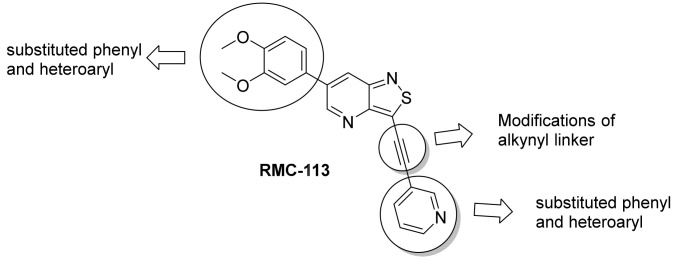
Hit compound RMC-113 and medicinal chemistry strategy.

We recently reported the discovery of a dual PIKfyve/PIP4K2C inhibitor, called RMC-113, based on an isothiazolo[4,3-*b*]pyridine scaffold [24]. RMC-113 displayed strong binding affinity for PIKfyve (*K*_D_ = 370 nM) and PIP4K2C (*K*_D_ = 46 nM) and was also a potent inhibitor of PIKfyve in an enzymatic assay (IC_50_ = 8 nM). Using RMC-113 as a chemical probe, this allowed us to validate PIP4K2C, beyond PIKfyve, as a druggable antiviral target with RMC-113 displaying broad-spectrum antiviral activity against SARS-CoV-2, the vaccine strain of VEEV (TC-83), dengue virus 2, Ebola virus and Marburg virus. However, this previous, mechanistic study did not include a structure–activity relationship (SAR) study, as only RMC-113 was investigated. Therefore, in order to demonstrate that RMC-113 is not a singleton hit and to study the structural features that were required for dual PIKfyve and PIP4K2C inhibition, we embarked upon the synthesis and SAR study of 3,6-disubstituted isothiazolo[4,3-*b*]pyridines (Figure 3). Three different regions of RMC-113 were subjected to structural modifications: (1) the dimethoxyphenyl moiety; (2) the alkynyl linker and (3) the pyridinyl moiety, whereas the central isothiazolo[4,3-*b*]pyridine scaffold was kept intact.

## 2. Results and Discussion

### 2.1. Chemistry

**Scheme 1:**
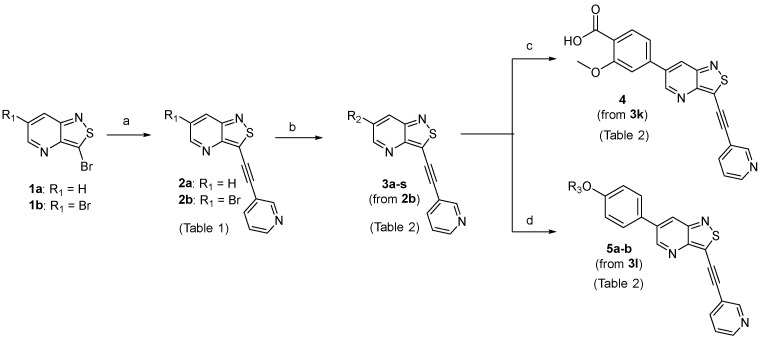
Synthesis of 3-(pyridin-3-ylethynyl)-6-aryl-isothiazolo[4,3-*b*]pyridines. *Reagents and conditions*: (a) 3-ethynylpyridine, CuI, Pd(PPh_3_)_2_Cl_2_, Et_3_N, THF, 30 °C; (b) from **2b**: boronic acid or ester, Pd(PPh_3_)_4_, K_2_CO_3_, dioxane/H_2_O (9:1), 90 °C, overnight; (c) from **3k**: LiOH, THF/H_2_O, 50 °C, overnight; (d) from **3l**: appropriate chloride, K_2_CO_3_, KI, DMF at 100 °C (for **5a**) or acetone at reflux (for **5b**).

The synthesis of 3-(pyridin-3-ylethynyl)isothiazolo[4,3-*b*]pyridines with structural variation at position 6 started from the key intermediates 3-bromo- and 3,6-dibromoisothiazolo[4,3-*b*]pyridine **1a** and **1b**, respectively, which were synthesized following known procedures [25] (Scheme 1). A regioselective Sonogashira cross-coupling reaction with 3-ethynylpyridine, using standard reaction conditions (copper iodide, Pd(PPh_3_)_2_Cl_2_ as catalyst, triethylamine as base and tetrahydrofuran as solvent), afforded the 3-(pyridin-3-ylethynyl)isothiazolo[4,3-*b*]pyridines **2a-b** in a good yield. It has been shown before that the presence of an external oxidant such as atmospheric oxygen can contribute to the undesirable homocoupling of acetylenes, also known as the Glaser coupling [26]. In order to avoid this copper-catalyzed oxidative homocoupling and to reduce as much as possible the amount of oxygen in the solvent, a continuous flow of argon was passed through the reaction mixture prior to the addition of the catalyst and also during the dropwise addition (over a period of 30 min) of a solution of the acetylene in THF. Both the continuous flow of argon and the slow addition of the acetylenes were crucial to allow easy purifications and hence to obtain a good yield. The subsequent Suzuki cross-coupling reaction with a wide range of boronic acids and boronic pinacol esters (using Pd(PPh_3_)_4_ as catalyst, potassium carbonate as base in a mixture of dioxane and water) furnished compounds **3a-s** in moderate to good yields.

The saponification of the methyl ester group of compound **3k** using lithium hydroxide in a mixture of tetrahydrofuran and water afforded the carboxylic acid **4** in excellent yield (94%). Compound **3l**, bearing a 4-hydroxyphenyl moiety, was subjected to alkylation reactions with 1-(2-chloroethyl)pyrrolidine and 4-(2-chloroethyl)morpholine yielding compounds **5a** (25%) and **5b** (48%), respectively.

**Scheme 2:**
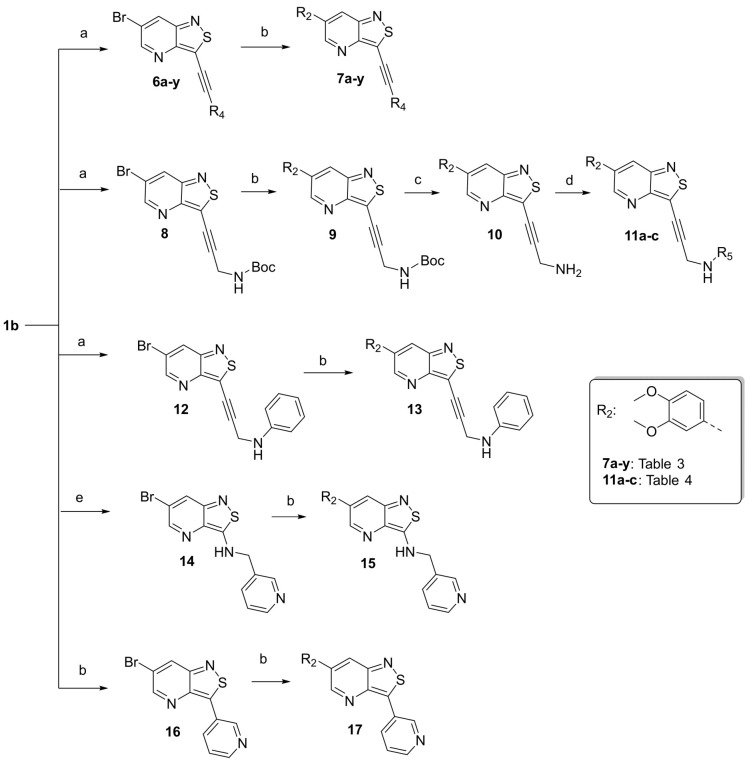
Synthesis of 3-substituted-6-(3,4-dimethoxyphenyl)-isothiazolo[4,3-*b*]pyridines. *Reagents and conditions*: (a) alkyne, CuI, Pd(PPh_3_)_2_Cl_2_, Et_3_N, THF, 30 °C; (b) boronic acid or ester, Pd(PPh_3_)_4_, K_2_CO_3_, dioxane/H_2_O (9:1), 90 °C, overnight; (c) HCl, dioxane, rt; (d) acid chloride or sulfonyl chloride or isocyanate, TEA, DCM, rt; (e) 3-(aminomethyl)pyridine, K_2_CO_3_, acetonitrile, reflux.

The synthesis of isothiazolo[4,3-*b*]pyridines with a 3,4-dimethoxyphenyl residue at position 6 and with structural modifications at position 3 is depicted in Scheme 2. Using similar reaction conditions as in Scheme 1, different alkynyl groups were introduced at position 3 of the scaffold, yielding a variety of 3-alkynyl substituted isothiazolo[4,3-*b*]pyridines **6a**-**y**. Intermediate **8** was obtained using N-Boc-propargylamine as coupling partner in the Sonogashira reaction. The synthesis of compound **12** with an anilinopropargyl moiety at position 3 of the scaffold, was achieved by a Sonogashira coupling with *N*-(propargyl)aniline (which was prepared by alkylation of aniline with propargyl bromide, using potassium carbonate as base). Applying a regioselective nucleophilic aromatic substitution (with 3-(aminomethyl)pyridine) and Suzuki coupling (using 3-pyridylboronic acid) on compound **1b** afforded the 3-substituted isothiazolo[4,3-*b*]pyridines **14** and **16**, respectively. The 3-substituted-6-bromo-isothiazolo[4,3-*b*]pyridines **6a**-**y**, **12**, **14** and **16** were subjected to a Suzuki coupling with 3,4-dimethoxyphenylboronic acid yielding final compounds **7a-y**, **13**, **15** and **17**. Intermediate **9** was obtained in a similar way. Deprotection of the *tert*-butoxycarbonyl (Boc) group using a 4M hydrogen chloride solution in 1,4-dioxane [27] afforded the propargylamino derivative **10**. Coupling with benzoyl chloride, phenyl isocyanate and benzenesulfonyl chloride, using triethylamine as base, gave the corresponding amide **11a**, urea **11b** and sulfonamide **11c**, respectively.

**Scheme 3:**
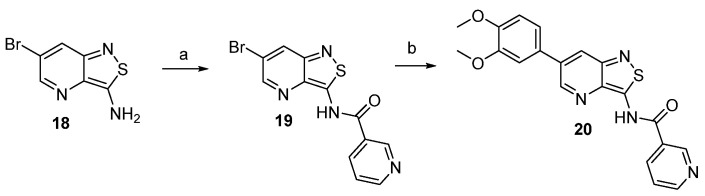
Synthesis of a 3-*N*-acyl-isothiazolo[4,3-*b*]pyridine. *Reagents and conditions*: (a) nicotinoyl chloride, Et_3_N, DCM, rt; (b) boronic acid, Pd(PPh_3_)_4_, K_2_CO_3_, dioxane/H_2_O, 90 °C, overnight.

The exocyclic amino group of 3-amino-6-bromo-isothiazolo[4,3-*b*]pyridine **18** (synthesized according to a literature procedure) [25] was coupled with nicotinoyl chloride yielding amide **19**. A Suzuki reaction furnished target compound **20** (Scheme 3).

**Scheme 4:**
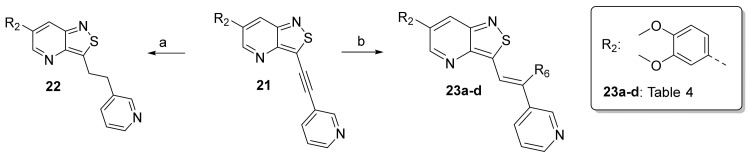
Structural modifications of the alkynyl linker. *Reagents and conditions*: (a) Pd(OH)_2_/C 20 wt%, EtOH/THF, 60 °C, 3 days; (b) methanol (for **23a**), 2-(dimethylamino)ethan-1-ol (for **23b**), *t*-BuOK, 80 °C or morpholine, 80 °C (for **23c**) or 2-mercaptoethanol, K_2_CO_3_, dioxane, 80 °C (for **23d**).

**Figure 4:**
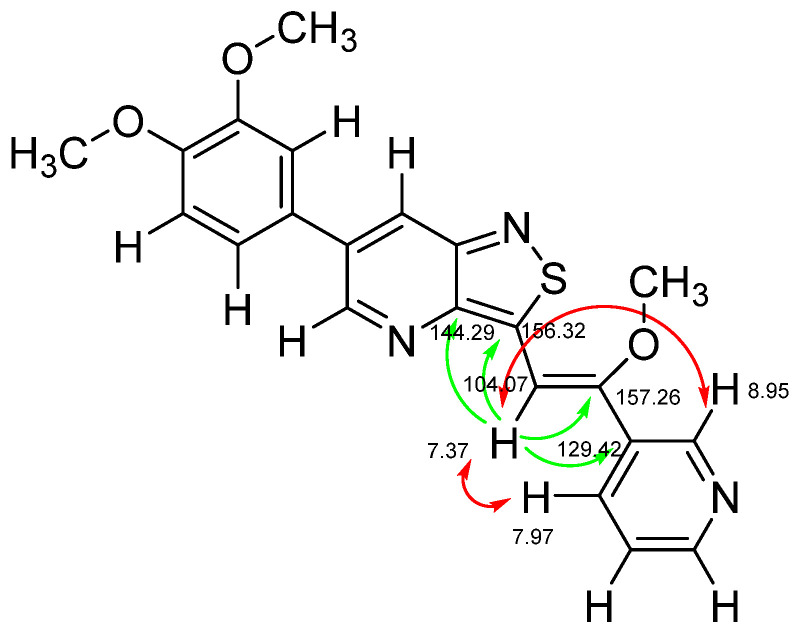
Key chemical shifts, HMBC couplings and NOESY correlations in compound **23a** (green arrows: HMBC couplings; red arrows: NOE correlation).

The insertion of structural modifications on the acetylenic moiety is shown in Scheme 4. The alkyne functionality of the known compound **21** (RMC-113) [28] was reduced by catalytic hydrogenation using palladium hydroxide and hydrogen gas at atmospheric pressure, yielding the corresponding alkane **22** in low yield (20%). The addition of various nucleophiles to the alkynyl moiety of compound **21** yielded compounds **23a-d**. Theoretically, four possible isomers can be formed, arising from the attack of the nucleophile to one of the ethynyl carbons and the possible formation of (*E*)- or (*Z*)-diastereomers. However, one major product was isolated. In order to unequivocally identify the structure (and especially the regio- and stereochemistry of the alkenyl moiety), the information from a combination of one-dimensional (^1^H- and ^13^C-NMR) and two-dimensional (COSY, HSQC, HMBC and NOESY) NMR spectra was applied (Figure 3). Compound **23a** was selected as a representative example, with the structures of the remaining compounds **23b-d** being interpreted in analogy. From ^1^H-NMR, ^13^C-NMR, COSY and HSQC spectra, the formation of the ethenyl moiety was evident. The ^1^H-NMR spectrum of compound **23a** showed a vinylic proton at δ_H_ = 7.37 ppm, whereas the ^13^C-NMR spectrum showed olefinic carbons at δ_C_ 104.07 ppm and 157.26 ppm. The exact position of the methoxy group on the double bond was confirmed by HMBC. The vinylic proton (δ_H_ = 7.37 ppm) showed long-range HMBC correlations to C-3 (δ_C_ = 156.32 ppm) and C-3a (δ_C_ = 144.29 ppm). Finally, in the NOESY spectrum of compound **23a**, the signal at δ_H_ 7.37 ppm (assigned to the vinylic proton) showed correlations with δ_H_ 7.97 and 8.95 (both protons on the 3-pyridyl moiety), and hence the stereochemistry of the double bond was assigned as being the (*Z*)-diastereomer (Figure 4).

### 2.2. Enzymatic Assays

#### 2.2.1. PIKfyve Inhibition

The primary assay guiding the medicinal chemistry was a biochemical PIKfyve assay, in which apilimod was included as positive control, yielding very low IC_50_ values (IC_50_ = 0.000786 µM), in agreement with literature values. RMC-113 (compound **21**), the lead compound, was included as a reference molecule. At the start of the project, no information was available with respect to the importance of the 3,4-dimethoxyphenyl and pyridyl moiety for PIKfyve inhibition and, therefore, a first round of SAR focused on these two groups.

The 6-unsubstituted analogue **2a** displayed greatly diminished potency on PIKfyve (IC_50_ = 1.15 µM), relative to RMC-113. Similarly, replacing the 3-pyridinyl moiety by an aliphatic *n*-propyl side chain afforded derivative **7a**, with 100-fold reduced activity on PIKfyve (IC_50_ = 0.85 µM), relative to RMC-113 (Table 1). Consequently, the subsequent SAR study focused on the insertion of various (hetero)aryl groups at position 6 and various bulky groups at position 3.

In a first round of SAR (Table 2), the focus was on the replacement of both dimethoxy groups by other electron-donating substituents, including a single methoxy group at various positions, such as in derivatives **3a** and **3b**, both exhibiting equipotency on PIKfyve as RMC-113. Similarly, the 2-methyl (compound **3c**), is as active as RMC-113 as PIKfyve inhibitor (both having an IC_50_ value of 8 nM), whereas the 4-dimethylamino (compound **3d**), and the 4-amino-3-methoxy (compound **3f**) congeners are more potent PIKfyve inhibition than RMC-113. In contrast, the 4-isopropoxy analogue (compound **3e**) was slightly less active (IC_50_ = 20 nM) than RMC-113 (IC_50_ = 8 nM). Since the presence of methoxy groups may impact metabolic stability, various halogens were introduced on the phenyl moiety. In addition, these halogens are also electron-withdrawing, allowing the study the importance of the electronics of the phenyl ring. The presence of a fluorine (compounds **3g** and **3h**) or a chlorine (compound **3i**) was well tolerated, as evidenced by the good PIKfyve potency of the respective analogs. In order to address potential aqueous solubility issues for future in vivo animal studies, water solubilizing groups were introduced. The presence of a carboxylic acid on the phenyl group (compound **4**) led to decreased PIKfyve inhibition (IC_50_ = 49 nM versus 8 nM for RMC-113). In contrast, insertion of an ethylene glycol side chain or basic amines yielded compounds **3i** and **5a**-**b**, respectively, that were even more potent than RMC-113, making this position ideally suited to manipulate physicochemical properties.

A carboxylic acid functions as a chemical handle that allows for easy introduction of structural variety and the convenient exploration of the SAR by the synthesis of a series of amides. Indeed, various carboxamides, either at the *meta* (compounds **3p**-**s**) or *para* (compounds **3m**-**o**) position demonstrated potent PIKfyve inhibition. Especially a carboxamide at position 4 (compound **3m**) or a methoxyethylaminocarbonyl at position 3 (compound **3s**) of the phenyl ring gave rise to very potent PIKfyve inhibitors.

An aliphatic chain on the ethynyl group did not lead to potent PIKfyve inhibition (Table 1), and hence, we focused on various aromatic derivatives instead of the 3-pyridinyl group. The 3,4-dimethoxyphenyl group was selected as substituent at position 6 of the scaffold, since RMC-113 (compound **21**) was already extensively biologically profiled [24], which allowed then for easier comparison. The synthesis of a phenyl analogue (compound **7b**), halogenated phenyl derivatives (compounds **7c**-**e**) as well as an anisole (compound **7f**) was effected (Table 3). These compounds are 2-8-fold less potent on PIKfyve relative to RMC-113, pointing towards an essential role of the 3-pyridinyl group.

Therefore, subsequent efforts focused on the introduction of various small substituents (methyl, methoxy, ethoxy, fluorine, cyano, trifluoromethyl and COOCH_3_) on the 3-pyridinyl moiety, affording compounds **7g**-**v**. The substitution pattern had a profound impact on PIKfyve inhibition. The presence of electron-withdrawing groups on the pyridine ring (at various positions) had a detrimental impact on PIKfyve inhibition (when compared to the unsubstituted analogue RMC-113), exemplified by the methylester analogue **7m** (IC_50_ = 0.12 µM), the trifluoromethyl derivative **7n** (IC_50_ = 0.68 µM), and especially the picolinonitrile analogue **7q** which was completely devoid of PIKfyve inhibition (IC_50_ > 10 µM). Similarly, the insertion of a fluorine at different positions of the pyridinyl ring afforded compounds **7j**, **7k** and **7r** that were less active as PIKfyve inhibitors than lead compound RMC-113.

Compounds **7i** and **7l** emerged as the most potent PIKfyve inhibitors in this series (IC_50_ values of 2 and 3 nM, respectively), suggesting that the presence of an electron-donating group (methyl or methoxy) is beneficial for PIKfyve inhibition. The position of this substituent on the 3-pyridinyl ring is of paramount importance for PIKfyve inhibition. The 5-methoxy-3-pyridinyl analogue **7l** (IC_50_ = 3 nM) is 200-fold more potent than the 4-methoxy-3-pyridinyl analogue **7h** (IC_50_ = 0.59 µM) and 6-fold more potent than the 6-methoxy-3-pyridinyl analogue **7o** (IC_50_ = 0.019 µM). Similarly, the 5-methyl-3-pyridinyl analogue **7i** (IC_50_ = 0.002 µM) is more potent than the 4-methyl-3-pyridinyl and 6-methyl-3-pyridinyl congeners **7g** and **7p**, displaying IC_50_ values of 0.36 and 0.033 µM, respectively. In general, the insertion of substituents at position 2 of the 3-pyridinyl ring (yielding compounds **7s**-**u**) was detrimental for PIKfyve inhibition, with IC_50_ values exceeding 100 nM. Only the 2-fluoro-5-methyl-3-pyridinyl analogue **7v** displayed potent PIKfyve inhibitory activity with an IC_50_ value of 42 nM.

Fusing the pyridinyl group with a phenyl ring yielded the quinoline analogue **7w** (IC_50_ = 5.69 µM), with 700-fold less potency on PIKfyve relative to RMC-113. This suggested that increasing the steric bulk at this position was not tolerated for PIKfyve inhibition. Moving the nitrogen around in the aromatic ring yielded the 4-pyridinyl analogue **7x** and the 2-pyridinyl analogue **7y** that both displayed potent PIKfyve inhibition, albeit less active than the corresponding 3-pyridinyl congener.

To probe the importance of the alkynyl linker for PIKfyve inhibition, various modifications were made. The acetylene moiety is a rigid linker, which might negatively impact aqueous solubility. Also, since this triple bond reacts with various nucleophiles (Scheme 4), it might lead to irreversible inhibition of proteins. Altogether, this spurred us to explore the SAR of the alkynyl moiety (Table 4). Deletion of the triple bond yielded the 3-(pyridin-3-yl)-isothiazolo[4,3-*b*]pyridine analogue **17**, having greatly reduced PIKfyve potency (IC_50_ = 0.602 µM), relative to RMC-113. Replacing the triple bond with an aminomethylene or amide linker furnished compounds **15** and **20**, respectively, that were practically inactive. The presence of a fully saturated ethyl linker yielded compound **22** that maintained PIKfyve inhibition, albeit less potent than its acetylenic congener RMC-113, with an IC_50_ value of 33 nM. Furthermore, the addition of oxygen-, nitrogen- and sulfur-containing nucleophiles to the triple bond afforded alkenyl analogues (compounds **23a**-**d**) with diminished PIKfyve inhibition, relative to the alkynyl derivative RMC-113. Overall, these data revealed a clear preference for an ethynyl linker for PIKfvye inhibition. Therefore, a propargylamine was introduced, since it retained the alkynyl linker, but allowed for easy structural variation of the terminal amino functionality. Conversion of this amino group into a carboxamide (compound **11a**), a sulfonamide (compound **11b**), a urea (compound **11c**) or an aniline (compound **13**) yielded derivatives that were less potent on PIKfvye than RMC-113, with IC_50_ values between 0.18 and 10 µM.

#### 2.2.2. PIP5K2C Binding Assay

Due the low enzymatic activity of PIP4K2C [29], a classical biochemical enzymatic assay to screen for inhibitors is not trivial, and therefore a binding assay was used. Compound **21** (RMC-113), known for its dual activity as PIP4K2C binder (*K*_D_ = 0.046 µM) and PIKfyve inhibitor (IC_50_ = 0.008 µM) was included as a reference (Table 5) [24]. From the newly synthesized isothiazolo[4,3-*b*]pyridines, various potent PIKfvye inhibitors were selected with structural variation of the dimethoxyphenyl moiety, with either electron-donating (compounds **3a** and **3f**) or electron-withdrawing (compounds **3i**, **3m**, **4** and **3s**) groups. In addition, a few representatives from the analogues that were prepared to probe the SAR of the pyridinyl ring were also selected. Since in this series, there is more variation in the SAR, compounds were selected that are potent PIKfyve inhibitors (compounds **7e**, **7k**, **7l**, **7o** and **7r**), have intermediate PIKfyve inhibitory activity (compound **7w**) or completely lack PIKfyve inhibition (compound **7q**).

Compounds that lacked (compound **7q**) or showed moderate (compound **7w**) PIKfyve inhibition, were also devoid of PIP4K2C binding affinity (*K*_D_ values of 30 µM and 2 µM, respectively). All other compounds evaluated were potent PIKfyve inhibitors (with IC_50_ values in the range of 1.6 to 98 nM) that showed a 2- to 5-fold loss in potency in the PIP4K2C assay (when compared to the PIKfyve data), with *K*_D_ values in range of 30 to 110 nM.

### 2.3. Cellular Assays

PIKfyve emerged as a promising drug target in various diseases, including virology and oncology. In addition, we recently validated PIP4K2C as a cellular target for the development of antiviral agents [24], although its role as drug target in oncology is less clear. Therefore, a representative selection of the newly synthesized PIKfyve inhibitors was evaluated for antiviral and antitumoral activity.

#### 2.3.1. Antiviral Activity

PIKfyve inhibitors decrease endocytic trafficking of various viruses (such as filoviruses and coronaviruses) and hence PIKfyve inhibition has been proposed as a broad-spectrum antiviral strategy [13]. PIP4K2C regulates viral entry, viral RNA replication and viral assembly/egress [24]. The lead compound RMC-113 has been shown to display antiviral activity against SARS-CoV-2, the vaccine strain of VEEV (TC-83), dengue virus 2, Ebola virus and Marburg virus [24]. As part of the current SAR study, the antiviral profiling was limited to two unrelated viruses: SARS-CoV-2, a beta-coronavirus, and the venezuelan equine encephalitis virus (VEEV), an alphavirus belonging to the family of Togaviridae. In order to demonstrate a selective antiviral effect, compounds were also evaluated for potential cytotoxicity against U-87 MG and VeroE6/TMPRS2 cells, that were used for VEEV and SARS-CoV-2 screening, respectively. RMC-113 showed potent antiviral activity against SARS-CoV-2 and VEEV (vaccine strain, TC-83) with low µM EC_50_ values for both viruses and without apparent cytotoxicity, in agreement with previous studies (Table 6) [24]. Although apilimod is a very potent PIKfyve inhibitor, this did not translate in improved antiviral efficacy, when compared to RMC-113. A selection of the newly synthesized isothiazolo[4,3-*b*]pyridines, with either structural variations on the phenyl ring at position 6 (compounds **3a**, **3h**, **3f**, **3m**, **4**, **5a**-**b** and **3s**) or on the pyridinyl ring (compounds **7e**-**f**, **7k**-**m**, **7o**-**p**, **7r** and **7w**) was investigated for antiviral activity. The majority of the compounds demonstrated a comparable antiviral profile (i.e., active against both viruses without displaying cytotoxicity) as RMC-113. Although compounds **3m**, **3s** and **7l** are more potent PIKfyve inhibitors than RMC-113, they still have a similar antiviral activity, compared to RMC-113. Compound **7w** was the least active PIKfvye inhibitor (IC_50_ = 5.69 µM) that was studied for its antiviral activity. It was less potent against VEEV (EC_50_ = 2.08 µM) than RMC-113, whereas in the SARS-CoV-2 screening, only cytotoxicity was observed. Overall, there is no clear correlation between enzymatic potency and antiviral activity. This might be due to the fact that a cellular antiviral effect is the result of various factors, including solubility, permeability, efflux, stability, and interaction with various targets.

RMC-113 has been shown to have activity against viruses from different families (i.e., Coronaviridae, Togaviridae, Filoviridae, Flaviviridae) and, hence, can be designated as a broad-spectrum antiviral agent. The newly synthesized analogues are very close analogues of RMC-113 and therefore, most likely, these are also candidates for the development of broad-spectrum antiviral agents.

#### 2.3.2. Antitumoral Activity

PIKfyve inhibitors are known to inhibit the proliferation of various types of cancer cells [30]. The role of PIP5K2C in cellular proliferation has not been investigated yet. Therefore, a selection of dual PIKfyve/PIP5K2C inhibitors was screened for antitumoral activity against a panel of cancer cell lines, representing solid (Capan-1: pancreatic ductal adenocarcinoma cell line; HCT-116: colon cancer cell line; LN-229: human glioblastoma cell line; NCI-H460: human non-small-cell lung cell line) and hematological (DND-41: T-cell leukemia cell line; HL-60: acute myeloid leukaemia cell line; K-562: chronic myelogenous leukemia cell line; Z-138: B-cell acute lymphoblastic leukemia cell line) cancers. Various known PIKfyve inhibitors (apilimod, vacuolin-1 and YM-201636) were included as controls (Table 7). In line with prior reports, apilimod shows potent antiproliferative activity against all cell lines investigated [12], whereas vacuolin-1 and YM-201636 showed reduced activity [31]. The lead compound **21** (RMC-113) showed low micromolar activity against all cancer cell lines tested. Potent PIKfyve inhibitors with structural modifications of the dimethoxyphenyl moiety (such as compounds **3g**, **3b** and **3m**) showed a comparable antitumoral effect to RMC-113. In contrast, potent PIKfyve inhibitors harboring structural variations on the pyridinyl moiety showed lower (compounds **7l**) or no (compounds **7i** and **7p**) antitumoral activity. These findings suggest that other factors, besides PIKfvye and/or PIP5K2C, also play a role in the anticancer activity of this compound class.

## 3. Materials and Methods

**General**

For all reactions, analytical grade solvents were used. Argon was used to carry out reactions under an inert atmosphere. Melting points were recorded with a Stuart SMP20 melting point apparatus. ^1^H and ^13^C NMR spectra were recorded on a Bruker Avance 300 MHz instrument (^1^H NMR, 300 MHz; ^13^C NMR, 75 MHz), 500 MHz instrument (^1^H NMR, 500 MHz; ^13^C NMR, 125 MHz) or a 600 MHz instrument (^1^H NMR, 600 MHz; ^13^C NMR, 150 MHz), using tetramethylsilane as internal standard for ^1^H NMR spectra and DMSO-d_6_ (39.5 ppm) or CDCl_3_ (77.2 ppm) for ^13^C NMR spectra. Abbreviations used are s = singlet, d = doublet, t = triplet, q = quartet, m = multiplet, b = broad. Coupling constants are expressed in Hz. High-resolution mass spectra were acquired on a quadrupole orthogonal acceleration time-of-flight mass spectrometer (Synapt G2 HDMS, Waters, Milford, MA, USA). Samples were infused at 3 mL/min and spectra were obtained in positive or negative ionization mode with a resolution of 15,000 (FWHM) using leucine enkephalin as lock mass. Precoated aluminum sheets (Fluka silica gel/TLC-cards, 254 nm) were used for TLC. Column chromatography was performed on silica gel 0.060–0.200 mm, 60 (Acros Organics). The ratio or percentage of solvents in the mobile phase is indicated as (*v*/*v*) or percentage (%), respectively. The elution procedure is indicated as percentage at starting point, percentage of end point and running time when a gradient is applied.

**3-Amino-2-cyanopyridine**
This compound was prepared from 3-nitro-2-cyanopyridine (436 mg, 2.9 mmol, 1.0 eq.) and iron powder (810 mg, 14.5 mmol, 5 eq.) in acetic acid (10 mL) using a previously described protocol [25]. The title compound was isolated without further purification as a light-brown solid in 90% yield (313 mg, 2.63 mmol).
^1^H NMR (400 MHz, DMSO-*d*_6_) δ: 7.86 (dd, *J* = 4.3, 1.4 Hz, 1H), 7.31 (dd, *J* = 8.6, 4.3 Hz, 1H), 7.21 (dd, *J* = 8.6, 1.5 Hz, 1H), 6.25 (s, 2H).
^13^C NMR (101 MHz, DMSO-*d*_6_) δ: 148.8, 139.0, 128.1, 123.1, 117.0, 114.5.
HRMS *m*/*z* [M+H]^+^ calcd for C_6_H_5_N_3_ 120.0556, found 120.0563.
**3-Aminopyridine-2-carbothioamide**
This compound was prepared from 3-amino-2-cyanopyridine (300 mg, 2.5 mmol, 1.0 eq.) and phosphorus pentasulfide (2.22 g, 5 mmol, 2 eq.) in ethanol (10 mL) using a previously described protocol [25]. The crude mixture was purified by silica gel chromatography (PE/EtOAc, 8:2), yielding the title compound as a light-brown solid in 65% yield (252 mg, 1.64 mmol).
^1^H NMR (400 MHz, CDCl_3_) δ: 9.57 (s, 1H), 7.87 (dd, *J* = 4.1, 1.5 Hz, 1H), 7.26 (s, 1H), 7.19 (dd, *J* = 8.4, 4.1 Hz, 1H), 7.07 (dd, *J* = 8.4, 1.4 Hz, 1H), 6.86 (s, 2H).
^13^C NMR (101 MHz, CDCl_3_) δ: 195.2, 146.8, 136.2, 129.9, 128.1, 126.9.
HRMS *m*/*z* [M+H]^+^ calcd for C_6_H_7_N_3_S 154.0433, found 154.0439.
**Isothiazolo[4,3-*b*]pyridin-3-amine**
This compound was prepared from 3-aminopyridine-2-carbothioamide (237 mg, 1.55 mmol, 1.0 eq.) and an 30% aqueous hydrogenperoxide solution (310 μL, 3.1 mmol, 2 eq.) in methanol (5 mL) using a previously described protocol [25]. The title compound was isolated without further purification as a dark-yellow solid in 82% yield (192 mg, 1.27 mmol).
^1^H NMR (400 MHz, DMSO-*d*_6_) δ: 8.28 (dd, *J* = 3.8, 1.4 Hz, 1H), 7.83 (s, 2H), 7.69 (dd, *J* = 9.0, 1.4 Hz, 1H), 7.24 (dd, *J* = 9.0, 3.8 Hz, 1H).
^13^C NMR (101 MHz, DMSO-*d*_6_) δ: 172.4, 153.6, 143.4, 134.8, 127.9, 123.6.
HRMS *m*/*z* [M+H]^+^ calcd for C_6_H_5_N_3_S 152.0277, found 152.0278.
**3-Bromoisothiazolo[4,3-*b*]pyridine (1a)**
Isothiazolo[4,3-*b*]pyridin-3-amine (170 mg, 1.12 mmol, 1.0 eq.) was dissolved in 48% aqueous HBr (17 mL) and stirred for 10 min at room temperature. CuBr (323 mg, 2.25 mmol, 2 eq.) was added and the mixture was cooled to 0 °C. An aqueous solution (8.5 mL) of NaNO_2_ (233 mg, 3.37 mmol, 3 eq.) was added dropwise (0.5 mL min*^−^*^1^). The reaction mixture was stirred for 2 h at 0 °C then overnight at room temperature. The mixture was cooled to 0 °C, neutralized with a 2M NaOH solution and extracted with EtOAc (30 mL) three times. The combined organic layers were washed with brine (30mL) and dried over Na_2_SO_4_. The solvent was removed in vacuo and the crude was purified by silica gel chromatography (PE/EtOAc, 8:2) yielding the title compound as a yellow solid in 61% yield (147 mg, 0.68 mmol).
^1^H NMR (400 MHz, CDCl_3_) δ: 8.87 (dd, *J* = 3.9, 1.5 Hz, 1H), 8.12 (dd, *J* = 9.0, 1.5 Hz, 1H), 7.40 (dd, *J* = 9.0, 3.8 Hz, 1H).
^13^C NMR (101 MHz, CDCl_3_) δ: 154.9, 152.2, 146.5, 135.8, 130.0, 123.6.
HRMS *m*/*z* [M+H]^+^ calcd for C_6_H_3_BrN_2_S 214.9274, found 214.9285.
**Sonogashira coupling at position 3 of the isothiazolo[4,3-*b*]pyridine scaffold**
General procedure
A solution of 3,6-dibromoisothiazolo[4,3-*b*]pyridine **1b** (1 eq.) [25] and triethylamine (3 eq.) in THF, was degassed with a flow of argon for 5 min. Then, Pd(PPh_3_)_2_Cl_2_ (0.02 eq.) and CuI (0.01 eq.) were added and the reaction mixture was allowed to reach 30 °C. Subsequently, a solution of the appropriate acetylene in THF was added slowly over a period of 30 min. The reaction was degassed a second time, filled with argon and stirred at 30 °C overnight. After disappearance of the starting material as monitored by TLC, the volatiles were evaporated in *vacuo* and the crude residue was purified by silica gel flash chromatography. Compounds **2a**-**b** and **6a-y** were made according to this procedure.
**3-(Pyridin-3-ylethynyl)isothiazolo[4,3-b]pyridine (2a)**
This compound was prepared from 3-bromoisothiazolo[4,3-*b*]pyridine **1a** (60 mg, 0.28 mmol, 1.0 eq.) and 3-ethynylpyridine (86 mg, 0.84 mmol, 3 eq.), Pd(PPh_3_)_2_Cl_2_ (4 mg, 0.007 mmol, 0.025 eq.) and CuI (0.6 mg, 0.003 mmol, 0.01 eq.) using the general Sonogashira coupling procedure. The crude mixture was purified by silica gel chromatography (PE/EtOAc, 7:3), yielding the title compound as a brown solid in 80% yield (53 mg, 0.22 mmol).
^1^H NMR (300 MHz, CDCl_3_) δ: 8.91 (s, 1H), 8.89 (dd, *J* = 3.9, 1.5 Hz, 1H), 8.64 (dd, *J* = 4.9, 1.7 Hz, 1H), 8.18 (dd, *J* = 8.9, 1.5 Hz, 1H), 7.97 (dt, *J* = 7.9, 1.9 Hz, 1H), 7.43 (dd, *J* = 9.0, 3.8 Hz, 1H), 7.36 (ddd, *J* = 7.9, 4.9, 0.9 Hz, 1H).
^13^C NMR (75 MHz, CDCl_3_) δ: 154.93, 152.29, 151.92, 149.77, 148.84, 143.61, 138.71, 130.07, 123.44, 123.14, 119.24, 104.15, 80.01.
HRMS *m*/*z* [M+H]^+^ calcd for C_13_H_7_N_3_S 238.0433, found 238.0440.
**6-Bromo-3-(pyridin-3-ylethynyl)isothiazolo[4,3-b]pyridine (2b)**
This compound was obtained from 3,6-dibromoisothiazolo[4,3-*b*]pyridine (101 mg, 0.34 mmol, 1.0 eq.), 3-ethynylpyridine (105 mg, 1.02 mmol, 3 eq.), Pd(PPh_3_)_2_Cl_2_ (5 mg, 0.007 mmol, 0.02 eq.) and CuI (0.6 mg, 0.003 mmol, 0.01 eq.) using the general Sonogashira coupling procedure. The crude residue was purified by flash chromatography using a mixture of hexane and ethyl acetate (in a ratio of 7:3) as mobile phase, affording the title compound as a light-yellow solid in 67% yield (72 mg, 0.23 mmol). Spectral data are in agreement with literature [28].
^1^H NMR (600 MHz, CDCl_3_) δ: 7.37 (ddd, *J* = 7.9, 4.9, 0.8 Hz, 1H, arom H), 7.94–7.98 (m, 1H, arom H), 8.36 (d, *J* = 2.1 Hz, 1H, arom H), 8.65 (dd, *J* = 4.9, 1.6 Hz, 1H, arom H), 8.84 (d, *J* = 2.1 Hz, 1H, arom H), 8.90 (d, *J* = 1.5 Hz, 1H, arom H).
HR-MS *m*/*z* [M+H]^+^ calcd for C_13_H_6_BrN_3_S 315.9539, found 315.9533.
**6-Bromo-3-(pent-1-yn-1-yl)isothiazolo[4,3-*b*]pyridine (6a)**
This compound was obtained from 3,6-dibromoisothiazolo[4,3-*b*]pyridine (101 mg, 0.34 mmol, 1.0 eq.), pent-1-yne (69 mg, 1.02 mmol, 3 eq.), Pd(PPh_3_)_2_Cl_2_ (5 mg, 0.007 mmol, 0.02 eq.) and CuI (0.6 mg, 0.003 mmol, 0.01 eq.) using the general Sonogashira coupling procedure. The crude residue was purified by flash chromatography using a mixture of hexane and diethyl ether (in a ratio of 95:5) as mobile phase, affording the title compound as a white solid in 71% yield (67.7 mg, 0.24 mmol).
^1^H NMR (300 MHz, CDCl_3_) *δ*: 1.10 (t, *J* = 7.4 Hz, 3H, CH_3_), 1.75 (h, *J* = 7.2 Hz, 2H, CH_2_), 2.65 (t, *J* = 7.1 Hz, 2H, CH_2_), 8.29 (d, *J* = 2.1 Hz, 1H, arom H), 8.77 (d, *J* = 2.1 Hz, 1H, arom H) ppm.
HRMS *m*/*z* [M+H]^+^ calcd for C_11_H_9_BrN_2_S 280.9743, found 280.9744.
**6-Bromo-3-(phenylethynyl)isothiazolo[4,3-b]pyridine (6b)**
This compound was obtained from 3,6-dibromoisothiazolo[4,3-*b*]pyridine (101 mg, 0.34 mmol, 1.0 eq.), ethynylbenzene (104 mg, 1.02 mmol, 3 eq.), Pd(PPh_3_)_2_Cl_2_ (5 mg, 0.007 mmol, 0.02 eq.) and CuI (0.6 mg, 0.003 mmol, 0.01 eq.) using the general Sonogashira coupling procedure. The crude residue was purified by flash chromatography using a mixture of hexane and diethyl ether (in a ratio of 95:5) as mobile phase, affording the title compound as a white solid in 81% (87 mg, 0.27 mmol).
^1^H NMR (300 MHz, CDCl_3_) δ: 8.82 (d, *J* = 2.0 Hz, 1H), 8.33 (d, *J* = 2.0 Hz, 1H), 7.67 (m, 2H), 7.43 (m, 3H) ppm.
^13^C NMR (75 MHz, CDCl_3_) δ: 154.90, 152.47, 146.71, 145.93, 131.96, 131.28, 129.89, 128.54, 121.68, 120.83, 103.01 ppm.
**6-Bromo-3-((4-fluorophenyl)ethynyl)isothiazolo[4,3-*b*]pyridine (6c)**
This compound was obtained from 3,6-dibromoisothiazolo[4,3-*b*]pyridine (101 mg, 0.34 mmol, 1.0 eq.), 1-ethynyl-4-fluorobenzene (122 mg, 1.02 mmol, 3 eq.), Pd(PPh_3_)_2_Cl_2_ (5 mg, 0.007 mmol, 0.02 eq.) and CuI (0.6 mg, 0.003 mmol, 0.01 eq.) using the general Sonogashira coupling procedure. The crude residue was purified by flash chromatography using a mixture of hexane and ethyl acetate (in a ratio of 95:5) as mobile phase, affording the title compound as a white solid in 89% yield (100.5 mg, 0.3 mmol).
^1^H NMR (300 MHz, CDCl_3_) *δ*: 7.07–7.16 (m, 2H, arom H), 7.62–7.71 (m, 2H, arom H), 8.34 (d, *J* = 2.1 Hz, 1H, arom H), 8.82 (d, *J* = 2.1 Hz, 1H, arom H) ppm.
HRMS *m*/*z* [M+H]^+^ calcd for C_14_H_6_BrFN_2_S 332.9492, found 332.9483.
**6-Bromo-3-((3-chlorophenyl)ethynyl)isothiazolo[4,3-*b*]pyridine (6d)**
This compound was obtained from 3,6-dibromoisothiazolo[4,3-*b*]pyridine (101 mg, 0.34 mmol, 1.0 eq.), 1-ethynyl-3-chlorobenzene (139 mg, 1.02 mmol, 3 eq.), Pd(PPh_3_)_2_Cl_2_ (5 mg, 0.007 mmol, 0.02 eq.) and CuI (0.6 mg, 0.003 mmol, 0.01 eq.) using the general Sonogashira coupling procedure. The crude residue was purified by flash chromatography using a mixture of hexane and ethyl acetate (in a ratio of 95:5) as mobile phase, affording the title compound as a light-yellow solid in 83% yield (98.6 mg, 0.28 mmol).
^1^H NMR (300 MHz, CDCl_3_) *δ*: 7.31–7.44 (m, 2H, arom H) 7.52–7.58 (m, 1H, arom H), 7.65–7.68 (m, 1H, arom H), 8.34 (d, *J* = 2.1 Hz, 1H, arom H), 8.83 (d, *J* = 2.1 Hz, 1H, arom H) ppm.
HRMS *m*/*z* [M+H]^+^ calcd for C_14_H_6_BrClN_2_S 348.9197, found 348.9206.
**6-Bromo-3-((2-fluorophenyl)ethynyl)isothiazolo[4,3-*b*]pyridine (6e)**
This compound was obtained from 3,6-dibromoisothiazolo[4,3-*b*]pyridine (101 mg, 0.34 mmol, 1.0 eq.), 1-ethynyl-2-fluorobenzene (123 mg, 1.02 mmol, 3 eq.), Pd(PPh_3_)_2_Cl_2_ (5 mg, 0.007 mmol, 0.02 eq.) and CuI (0.6 mg, 0.003 mmol, 0.01 eq.) using the general Sonogashira coupling procedure. The crude residue was purified by flash chromatography using a mixture of hexane and ethyl acetate (in a ratio of 95:5) as mobile phase, affording the title compound as a white solid in 78% yield (88.3 mg, 0.26 mmol).
^1^H NMR (300 MHz, CDCl_3_) *δ*: 7.12–7.24 (m, 2H, arom H), 7.37–7.48 (m, 1H, arom H), 7.59–7.70 (m, 1H, arom H), 8.34 (d, *J* = 2.1 Hz, 1H, arom H), 8.83 (d, *J* = 2.1 Hz, 1H, arom H) ppm.
HRMS *m*/*z* [M+H]^+^ calcd for C_14_H_6_BrFN_2_S 332.9492, found 332.9483.
**6-Bromo-3-((3-methoxyphenyl)ethynyl)isothiazolo[4,3-*b*]pyridine (6f)**
This compound was obtained from 3,6-dibromoisothiazolo[4,3-*b*]pyridine (101 mg, 0.34 mmol, 1.0 eq.), 3-ethynylanisole (135 mg, 1.02 mmol, 3 eq.), Pd(PPh_3_)_2_Cl_2_ (5 mg, 0.007 mmol, 0.02 eq.) and CuI (0.6 mg, 0.003 mmol, 0.01 eq.) using the general p Sonogashira coupling rocedure. The crude residue was purified by flash chromatography using a mixture of hexane and diethyl ether (in a ratio of 95:5) as mobile phase, affording the title compound as a yellow solid in 77% yield (90.4 mg, 0.26 mmol).
^1^H NMR (300 MHz, CDCl_3_) *δ*: 3.85 (s, 3H, OCH_3_), 6.99 (ddd, *J* = 8.0, 2.5, 1.4 Hz, 1H, arom H), 7.17–7.23 (m, 1H, arom H), 7.25–7.35 (m, 2H, arom H), 8.34 (d, *J* = 2.1 Hz, 1H, arom H), 8.83 (d, *J* = 2.1 Hz, 1H, arom H).
HRMS *m*/*z* [M+H]^+^ calcd for C_15_H_9_BrN_2_OS 344.9693, found 344.9693.
**6-Bromo-3-((6-methylpyridin-3-yl)ethynyl)isothiazolo[4,3-*b*]pyridine (6g)**
This compound was obtained from 3,6-dibromoisothiazolo[4,3-*b*]pyridine (101 mg, 0.34 mmol, 1.0 eq.), 5-ethynyl-2-methylpyridine (120 mg, 1.02 mmol, 3 eq.), Pd(PPh_3_)_2_Cl_2_ (5 mg, 0.007 mmol, 0.02 eq.) and CuI (0.6 mg, 0.003 mmol, 0.01 eq.) using the general Sonogashira coupling procedure. The crude residue was purified by flash chromatography using a mixture of dichloromethane and diethyl ether (in a ratio of 10:0.3) as mobile phase, affording the title compound as a yellow solid in 83% yield (93.2 mg, 0.28 mmol).
^1^H NMR (300 MHz, CDCl_3_) *δ*: 2.61 (s, 3H, CH_3_), 7.22 (d, *J* = 8.1 Hz, 1H, arom H), 7.84 (dd, *J* = 8.0, 2.1 Hz, 1H, arom H), 8.34 (d, *J* = 2.0 Hz, 1H, arom H), 8.78 (d, *J* = 1.5 Hz 1H, arom H), 8.83 (d, *J* = 2.0 Hz, 1H, arom H) ppm.
  HRMS *m*/*z* [M+H]^+^ calcd for C_14_H_8_BrN_3_S 329.9696, found 329.9696.
**6-Bromo-3-((6-methoxypyridin-3-yl)ethynyl)isothiazolo[4,3-*b*]pyridine (6h)**
This compound was obtained from 3,6-dibromoisothiazolo[4,3-*b*]pyridine (101 mg, 0.34 mmol, 1.0 eq.), 5-ethynyl-2-methoxypyridine (136 mg, 1.02 mmol, 3 eq.), Pd(PPh_3_)_2_Cl_2_ (5 mg, 0.007 mmol, 0.02 eq.) and CuI (0.6 mg, 0.003 mmol, 0.01 eq.) using the general Sonogashira coupling procedure. The crude residue was purified by flash chromatography using a mixture of hexane and ethyl acetate (in a ratio of 9:1) as mobile phase, affording the title compound as a yellow solid in 64% yield (75.3 mg, 0.22 mmol).
^1^H NMR (300 MHz, CDCl_3_) *δ*: 3.99 (s, 3H, OCH_3_), 6.79 (d, *J* = 8.6 Hz, 1H arom H), 7.82 (dd, *J* = 2.3, 8.6 Hz, 1H, arom H), 8.34 (d, *J* = 2.0 Hz, 1H, arom H), 8.49 (d, *J* = 2.0 Hz, 1H, arom H), 8.82 (d, *J* = 2.0 Hz, 1H, arom H) ppm.
HRMS *m*/*z* [M+H]^+^ calcd for C_14_H_8_BrN_3_OS 345.9645, found 345.9651.
**6-Bromo-3-((5-methylpyridin-3-yl)ethynyl)isothiazolo[4,3-*b*]pyridine (6i)**
This compound was obtained from 3,6-dibromoisothiazolo[4,3-*b*]pyridine (101 mg, 0.34 mmol, 1.0 eq.), 3-ethynyl-5-methylpyridine (120 mg, 1.02 mmol, 3 eq.), Pd(PPh_3_)_2_Cl_2_ (5 mg, 0.007 mmol, 0.02 eq.) and CuI (0.6 mg, 0.003 mmol, 0.01 eq.) using the general Sonogashira coupling procedure. The crude residue was purified by flash chromatography using a mixture of hexane and acetone (in a ratio of 9:1) as mobile phase, affording the title compound as a yellow solid in 79% yield (88.7 mg, 0.27 mmol).
^1^H NMR (300 MHz, CDCl_3_) *δ*: 2.39 (s, 3H, CH_3_), 7.77 (s, 1H arom H), 8.34 (d, *J* = 2.0 Hz, 1H, arom H), 8.47 (s, 1H, arom H), 8.71 (s, 1H, arom H), 8.83 (d, *J* = 2.0 Hz, 1H, arom H) ppm.
HRMS *m*/*z* [M+H]^+^ calcd for C_14_H_8_BrN_3_S 329.9696, found 329.9694.
**6-Bromo-3-((6-fluoropyridin-3-yl)ethynyl)isothiazolo[4,3-*b*]pyridine (6j)**
This compound was obtained from 3,6-dibromoisothiazolo[4,3-*b*]pyridine (101 mg, 0.34 mmol, 1.0 eq.), 5-ethynyl-2-fluoropyridine (124 mg, 1.02 mmol, 3 eq.), Pd(PPh_3_)_2_Cl_2_ (5 mg, 0.007 mmol, 0.02 eq.) and CuI (0.6 mg, 0.003 mmol, 0.01 eq.) using the general Sonogashira coupling procedure. The crude residue was purified by flash chromatography using a mixture of hexane and ethyl acetate (in a ratio of 9:1) as mobile phase, affording the title compound as a yellow solid in 68% yield (77.3 mg, 0.23 mmol).
^1^H NMR (300 MHz, CDCl_3_) *δ*: 7.02 (dd, *J* = 2.9, 8.5 Hz, 1H arom H), 8.06 (dt, *J* = 2.3, 8.0 Hz, 1H, arom H), 8.36 (d, *J* = 2.0 Hz, 1H, arom H), 8.55 (d, *J* = 1.5 Hz, 1H, arom H), 8.84 (d, *J* = 2.0 Hz, 1H, arom H) ppm.
HRMS *m*/*z* [M+H]^+^ calcd for C_13_H_5_BrFN_3_S 333.9445, found 333.9446.
**6-Bromo-3-((2-fluoropyridin-3-yl)ethynyl)isothiazolo[4,3-*b*]pyridine (6k)**
This compound was obtained from 3,6-dibromoisothiazolo[4,3-b]pyridine (101 mg, 0.34 mmol, 1.0 eq.), methyl 3-ethynyl-2-fluoropyridine (138 mg, 1.02 mmol, 3 eq.), Pd(PPh_3_)_2_Cl_2_ (5 mg, 0.007 mmol, 0.02 eq.) and CuI (0.6 mg, 0.003 mmol, 0.01 eq.) using the general Sonogashira coupling procedure. The crude residue was purified by flash chromatography using a mixture of hexane and ethyl acetate (in a ratio of 9:1) as mobile phase, affording the title compound as a beige solid in 81% yield (92.1 mg, 0.28 mmol).
^1^H NMR (300 MHz, CDCl_3_) *δ*: 7.26–7.31 (m, 1H, arom H), 8.03–8.12 (m, 1H, arom H), 8.28 (d, *J* = 4.4 Hz, 1H, arom H), 8.37 (d, *J* = 2.0 Hz, 1H, arom H), 8.85 (d, *J* = 1.9 Hz, 1H, arom H) ppm.
HRMS *m*/*z* [M+H]^+^ calcd for C_13_H_5_BrFN_3_S 333.9445, found 333.9445.
**6-Bromo-3-((5-methoxypyridin-3-yl)ethynyl)isothiazolo[4,3-*b*]pyridine (6l)**
This compound was obtained from 3,6-dibromoisothiazolo[4,3-*b*]pyridine (101 mg, 0.34 mmol, 1.0 eq.), 3-ethynyl-5-methoxypyridine (136 mg, 1.02 mmol, 3 eq.), Pd(PPh_3_)_2_Cl_2_ (5 mg, 0.007 mmol, 0.02 eq.) and CuI (0.6 mg, 0.003 mmol, 0.01 eq.) using the general Sonogashira coupling procedure. The crude residue was purified by flash chromatography using a mixture of hexane and ethyl acetate (in a ratio of 4:1) as mobile phase, affording the title compound as a yellow solid in 73% yield (85.9 mg, 0.25 mmol).
^1^H NMR (300 MHz, CDCl_3_) *δ*: 3.91 (s, 3H, OCH_3_). 7.44 (bs, 1H, arom H), 8.36 (bs, 2H, arom H), 8.50 (s, 1H, arom H), 8.85 (d, *J* = 1.7 Hz, 1H, arom H) ppm.
HRMS *m*/*z* [M+H]^+^ calcd for C_14_H_8_BrN_3_OS 345.9645, found 345.9646.
**Methyl 5-((6-bromoisothiazolo[4,3-*b*]pyridin-3-yl)ethynyl)nicotinate (6m)**
This compound was obtained from 3,6-dibromoisothiazolo[4,3-*b*]pyridine (101 mg, 0.34 mmol, 1.0 eq.), methyl 5-ethynylnicotinate (165 mg, 1.02 mmol, 3 eq.), Pd(PPh_3_)_2_Cl_2_ (5 mg, 0.007 mmol, 0.02 eq.) and CuI (0.6 mg, 0.003 mmol, 0.01 eq.) using the general Sonogashira coupling procedure. The crude residue was purified by flash chromatography using a mixture of hexane and acetone (in a ratio of 4:1) as mobile phase, affording the title compound as a beige solid in 65% yield (82.7 mg, 0.22 mmol).
^1^H NMR (300 MHz, CDCl_3_) *δ*: 4.00 (s, 3H, COOCH_3_), 8.38 (d, *J* = 1.9 Hz, 1H, arom H), 8.57 (t, *J* = 1.8 Hz, 1H, arom H), 8.86 (d, *J* = 1.9 Hz, 1H, arom H), 9.04 (d, *J* = 1.8 Hz, 1H, arom H), 9.23 (d, *J* = 1.8 Hz, 1H, arom H) ppm.
HRMS *m*/*z* [M+H]^+^ calcd for C_15_H_8_BrN_3_O_2_S 373.9594, found 373.9589.
**6-Bromo-3-((6-(trifluoromethyl)pyridin-3-yl)ethynyl)isothiazolo[4,3-*b*]pyridine (6n)**
This compound was obtained from 3,6-dibromoisothiazolo[4,3-*b*]pyridine (101 mg, 0.34 mmol, 1.0 eq.), 5-ethynyl-2-(trifluoromethyl)pyridine (175 mg, 1.02 mmol, 3 eq.), Pd(PPh_3_)_2_Cl_2_ (5 mg, 0.007 mmol, 0.02 eq.) and CuI (0.6 mg, 0.003 mmol, 0.01 eq.) using the general Sonogashira coupling procedure. The crude residue was purified by flash chromatography using a mixture of hexane and ethyl acetate (in a ratio of 9:1) as mobile phase, affording the title compound as a light-yellow solid in 75% yield (98.0 mg, 0.25 mmol).
^1^H NMR (300 MHz, CDCl_3_) *δ*: 7.76 (d, *J* = 8.1 Hz, 1H, arom H), 8.14 (d, *J* = 7.9 Hz, 1H, arom H), 8.38 (d, *J* = 1.8 Hz, 1H, arom H), 8.86 (d, *J* = 1.7 Hz, 1H, arom H), 8.98 (s, 1H, arom H) ppm.
HRMS *m*/*z* [M+H]^+^ calcd for C_14_H_5_BrF_3_N_3_S 383.9413, found 383.9413.
**6-Bromo-3-((4-methoxypyridin-3-yl)ethynyl)isothiazolo[4,3-*b*]pyridine (6o)**
This compound was obtained from 3,6-dibromoisothiazolo[4,3-*b*]pyridine (101 mg, 0.34 mmol, 1.0 eq.), 3-ethynyl-4-methoxypyridine (136 mg, 1.02 mmol, 3 eq.), Pd(PPh_3_)_2_Cl_2_ (5 mg, 0.007 mmol, 0.02 eq.) and CuI (0.6 mg, 0.003 mmol, 0.01 eq.) using the general Sonogashira coupling procedure. The crude residue was purified by flash chromatography using a mixture of hexane and ethyl acetate (in a ratio of 85:15) as mobile phase, affording the title compound as an orange solid in 71% yield (79 mg, 0.23 mmol).
^1^H NMR (400 MHz, Chloroform-*d*) δ 8.83 (d, *J* = 2.1 Hz, 1H), 8.75 (s, 1H), 8.55 (s, 1H), 8.34 (d, *J* = 2.0 Hz, 1H), 6.89 (d, *J* = 5.4 Hz, 1H), 4.00 (s, 3H) ppm.
^13^C NMR (101 MHz, CDCl_3_) δ 165.7, 154.9, 154.0, 152.8, 152.1, 147.0, 145.4, 131.4, 121.0, 102.1, 82.7, 56.1 ppm.
**6-Bromo-3-((4-methylpyridin-3-yl)ethynyl)isothiazolo[4,3-*b*]pyridine (6p)**
This compound was obtained from 3,6-dibromoisothiazolo[4,3-*b*]pyridine (101 mg, 0.34 mmol, 1.0 eq.), 5-ethynylpicolinonitrile (131 mg, 1.02 mmol, 3 eq.), Pd(PPh_3_)_2_Cl_2_ (5 mg, 0.007 mmol, 0.02 eq.) and CuI (0.6 mg, 0.003 mmol, 0.01 eq.) using the general Sonogashira coupling procedure. The crude residue was purified by flash chromatography using a mixture of hexane and ethyl acetate (in a ratio of 4:1) as mobile phase, affording the title compound as a yellow solid in 55% yield (59 mg, 0.18 mmol).
^1^H NMR (400 MHz, Chloroform-*d*) δ 8.84 (d, *J* = 2.1 Hz, 1H), 8.81 (s, 1H), 8.50 (d, *J* = 5.1 Hz, 1H), 8.35 (d, *J* = 2.1 Hz, 1H), 7.23 (d, *J* = 5.0 Hz, 1H), 2.59 (s, 3H) ppm.
^13^C NMR (101 MHz, CDCl_3_) δ 155.1, 153.0, 152.5, 149.9, 149.5, 147.1, 145.1, 131.4, 124.6, 121.1, 119.5, 104.1, 82.9, 20.5 ppm.
**5-((6-Bromoisothiazolo[4,3-*b*]pyridin-3-yl)ethynyl)picolinonitrile (6q)**
This compound was obtained from 3,6-dibromoisothiazolo[4,3-*b*]pyridine (101 mg, 0.34 mmol, 1.0 eq.), 3-ethynyl-5-fluoropyridine (124 mg, 1.02 mmol, 3 eq.), Pd(PPh_3_)_2_Cl_2_ (5 mg, 0.007 mmol, 0.02 eq.) and CuI (0.6 mg, 0.003 mmol, 0.01 eq.) using the general Sonogashira coupling procedure. The crude residue was purified by flash chromatography using a mixture of hexane and ethyl acetate (in a ratio of 4:1) as mobile phase, affording the title compound as a light-brown solid in 53% yield (58 mg, 0.17 mmol).
^1^H NMR (400 MHz, Chloroform-*d*) δ 8.96 (d, *J* = 1.5 Hz, 1H), 8.87 (d, *J* = 1.9 Hz, 1H), 8.39 (d, *J* = 2.1 Hz, 1H), 8.09 (dd, *J* = 8.1, 2.1 Hz, 1H), 7.76 (dd, *J* = 8.1, 0.9 Hz, 1H) ppm.
^13^C NMR (101 MHz, CDCl_3_) δ 155.1, 153.5, 153.3, 147.3, 143.5, 139.6, 133.3, 131.6, 128.0, 122.6, 121.3, 116.8, 102.7, 83.4 ppm.
**6-Bromo-3-((5-fluoropyridin-3-yl)ethynyl)isothiazolo[4,3-b]pyridine** (**6r**)
This compound was obtained from 3,6-dibromoisothiazolo[4,3-*b*]pyridine (101 mg, 0.34 mmol, 1.0 eq.), 3-ethynyl-5-fluoropyridine (124 mg, 1.02 mmol, 3 eq.), Pd(PPh_3_)_2_Cl_2_ (5 mg, 0.007 mmol, 0.02 eq.) and CuI (0.6 mg, 0.003 mmol, 0.01 eq.) using the general Sonogashira coupling procedure. The crude residue was purified by flash chromatography using a mixture of hexane and ethyl acetate (in a ratio of 7:3) as mobile phase, affording the title compound as an off-white solid in 56% yield (60 mg, 0.18 mmol).
^1^H NMR (400 MHz, Chloroform-*d*) δ 8.85 (d, *J* = 2.1 Hz, 1H), 8.72 (s, 1H), 8.53 (d, *J* = 2.7 Hz, 1H), 8.37 (d, *J* = 2.1 Hz, 1H), 7.69 (ddd, *J* = 8.7, 2.8, 1.6 Hz, 1H) ppm.
^13^C NMR (101 MHz, CDCl_3_) δ 155.1, 153.2, 148.3 (d, *J* = 4.1 Hz), 147.2, 144.2, 139.1, 138.9, 131.6, 125.5, 125.3, 121.2, 103.2 (d, *J* = 2.5 Hz), 80.5 ppm.
HRMS *m*/*z* [M+H]^+^ calcd for C_13_H_5_BrFN_3_S 333.9445, found 333.9454.
**6 -Bromo-3-((2-ethoxypyridin-3-yl)ethynyl)isothiazolo[4,3-b]pyridine (6s)**
This compound was obtained from 3,6-dibromoisothiazolo[4,3-b]pyridine (101 mg, 0.34 mmol, 1.0 eq.), 2-ethoxy-3-ethynylpyridine (150 mg, 1.02 mmol, 3 eq.), Pd(PPh_3_)_2_Cl_2_ (5 mg, 0.007 mmol, 0.02 eq.) and CuI (0.6 mg, 0.003 mmol, 0.01 eq.) using the general Sonogashira coupling procedure. The crude residue was purified by flash chromatography using a mixture of hexane and ethyl acetate (in a ratio of 9:1) as mobile phase, affording the title compound as a yellow solid in 82% yield (101 mg, 0.28 mmol).
^1^H NMR (400 MHz, Chloroform-*d*) δ 8.82 (d, *J* = 2.1 Hz, 1H), 8.34 (d, *J* = 2.1 Hz, 1H), 8.20 (dd, *J* = 5.0, 2.0 Hz, 1H), 7.88 (dd, *J* = 7.4, 2.0 Hz, 1H), 6.92 (dd, *J* = 7.4, 5.0 Hz, 1H), 4.50 (q, *J* = 7.1 Hz, 2H), 1.48 (t, *J* = 7.1 Hz, 3H) ppm.
^13^C NMR (101 MHz, CDCl_3_) δ 163.5, 155.0, 152.7, 148.3, 147.0, 145.9, 142.0, 131.4, 121.0, 116.3, 106.2, 104.2, 81.4, 62.8, 14.7 ppm.
HRMS *m*/*z* [M+H]^+^ calcd for C_15_H_10_BrN_3_OS 359.9801, found 359.9799.
**6-Bromo-3-((5-fluoro-2-methoxypyridin-3-yl)ethynyl)isothiazolo[4,3-b]pyridine** (**6t**)
This compound was obtained from 3,6-dibromoisothiazolo[4,3-b]pyridine (188 mg, 0.64 mmol, 1.0 eq.), 3-ethynyl-5-fluoro-2-methoxypyridine (290 mg, 1.92 mmol, 3 eq.), Pd(PPh_3_)_2_Cl_2_ (10 mg, 0.014 mmol, 0.02 eq.) and CuI (1.2 mg, 0.006 mmol, 0.01 eq.) using the general Sonogashira coupling procedure. The crude residue was purified by flash chromatography using a mixture of hexane and ethyl acetate (in a ratio of 9:1) as mobile phase, affording the title compound as a yellow solid in 78% yield (183 mg, 0.50 mmol).
^1^H NMR (400 MHz, Chloroform-*d*) δ 8.84 (d, *J* = 2.1 Hz, 1H), 8.35 (d, *J* = 2.1 Hz, 1H), 8.06 (d, *J* = 3.0 Hz, 1H), 7.66 (dd, *J* = 7.7, 3.0 Hz, 1H), 4.04 (s, 3H).
^13^C NMR (101 MHz, CDCl_3_) δ 159.9, 155.6, 154.9, 153.1, 152.9, 147.0, 144.8, 135.1, 134.8, 131.3, 129.2, 129.0, 120.9, 106.5, 106.4, 102.0, 101.9, 82.0, 54.7 ppm.
HRMS *m*/*z* [M+H]^+^ calcd for C_14_H_7_BrFN_3_OS 363.9550, found 363.9571.
**6-Bromo-3-((2,6-dimethoxypyridin-3-yl)ethynyl)isothiazolo[4,3-*b*]pyridine (6u)**
This compound was obtained from 3,6-dibromoisothiazolo[4,3-*b*]pyridine (188 mg, 0.64 mmol, 1.0 eq.), 3-ethynyl-2,6-dimethoxypyridine (313 mg, 1.92 mmol, 3 eq.), Pd(PPh_3_)_2_Cl_2_ (10 mg, 0.014 mmol, 0.02 eq.) and CuI (1.2 mg, 0.006 mmol, 0.01 eq.) using the general Sonogashira coupling procedure. This compound was obtained using 3-ethynyl-2,6-dimethoxypyridine. The crude residue was purified by flash chromatography using a mixture of hexane and ethyl acetate (in a ratio of 1:1) as mobile phase, affording the title compound as orange solid in 82% yield (200 mg, 0.53 mmol).
^1^H NMR (400 MHz, Chloroform-*d*) 8.80 (d, *J* = 2.1 Hz, 1H), 8.31 (d, *J* = 2.1 Hz, 1H), 7.77 (d, *J* = 8.2 Hz, 1H), 6.37 (d, *J* = 8.2 Hz, 1H), 4.06 (s, 3H), 3.98 (s, 3H).
^13^C NMR (101 MHz, CDCl_3_) 163.9, 163.4, 154.8, 152.2, 146.6, 146.4, 144.3, 131.2, 120.7, 105.1, 102.0, 96.4, 80.1, 54.2, 53.9.
HRMS *m*/*z* [M+H]^+^ calcd for C_15_H_10_BrN_3_O_2_S 375.9750, found 375.9747.
**6-Bromo-3-((2-fluoro-5-methylpyridin-3-yl)ethynyl)isothiazolo[4,3-*b*]pyridine** (**6v**)
This compound was obtained from 3,6-dibromoisothiazolo[4,3-*b*]pyridine (188 mg, 0.64 mmol, 1.0 eq.), 3-ethynyl-2-fluoro-5-methylpyridine (259 mg, 1.92 mmol, 3 eq.), Pd(PPh_3_)_2_Cl_2_ (10 mg, 0.014 mmol, 0.02 eq.) and CuI (1.2 mg, 0.006 mmol, 0.01 eq.) using the general Sonogashira coupling procedure. The crude residue was purified by flash chromatography using a mixture of hexane and ethyl acetate (in a ratio of 9:1) as mobile phase, affording the title compound as a light-brown solid in 91% yield (205 mg, 0.59 mmol).
^1^H NMR (400 MHz, Chloroform-*d*) 8.85 (d, *J* = 2.1 Hz, 1H), 8.36 (d, *J* = 2.1 Hz, 1H), 8.07 (dt, *J* = 2.1, 0.9 Hz, 1H), 7.88 (ddd, *J* = 8.6, 2.4, 0.8 Hz, 1H), 2.37 (s, 4H).
^13^C NMR (101 MHz, CDCl_3_) 162.0, 159.6, 154.9, 153.0, 148.3, 148.1, 147.1, 144.4, 144.0, 144.0, 131.4, 130.9, 130.9, 121.0, 105.5, 105.2, 99.9, 99.9, 82.1, 82.1, 17.3 ppm. HRMS *m*/*z* [M+H]^+^ calcd for C_14_H_7_BrFN_3_S 347.9601, found 347.9603.
**6-Bromo-3-(quinolin-3-ylethynyl)isothiazolo[4,3-*b*]pyridine (6w)**
This compound was obtained from 3,6-dibromoisothiazolo[4,3-*b*]pyridine (101 mg, 0.34 mmol, 1.0 eq.), 3-ethynylquinoline (156 mg, 1.02 mmol, 3 eq.), Pd(PPh_3_)_2_Cl_2_ (5 mg, 0.007 mmol, 0.02 eq.) and CuI (0.6 mg, 0.003 mmol, 0.01 eq.) using the general Sonogashira coupling procedure. The crude residue was purified by flash chromatography using a mixture of hexane and acetone (in a ratio of 9:1) as mobile phase, affording the title compound as a yellow solid in 81% yield (100 mg, 0.27 mmol).
^1^H NMR (300 MHz, CDCl_3_) *δ*: 7.62 (t, *J* = 7.5 Hz, 1H, arom H), 7.75–7.83 (m, 1H, arom H), 7.85 (d, *J* = 8.1 Hz, 1H, arom H), 8.14 (d, *J* = 8.5 Hz, 1H, arom H), 8.37 (d, *J* = 2.0 Hz, 1H, arom H), 8.49 (d, *J* = 1.9 Hz, 1H, arom H), 8.86 (d, *J* = 2.0 Hz, 1H, arom H), 9.11 (d, *J* = 2.0 Hz, 1H, arom H) ppm.
HRMS *m*/*z* [M+H]^+^ calcd for C_17_H_8_BrN_3_S 365.9696, found 365.9686.
**6-Bromo-3-(pyridin-4-ylethynyl)isothiazolo[4,3-*b*]pyridine (6x)**
This compound was obtained from 3,6-dibromoisothiazolo[4,3-*b*]pyridine (101 mg, 0.34 mmol, 1.0 eq.), 4-ethynylpyridine (105 mg, 1.02 mmol, 3 eq.), Pd(PPh_3_)_2_Cl_2_ (5 mg, 0.007 mmol, 0.02 eq.) and CuI (0.6 mg, 0.003 mmol, 0.01 eq.) using the general Sonogashira coupling procedure. The crude residue was purified by flash chromatography using a mixture of hexane and acetone (in a ratio of 4:1) as mobile phase, affording the title compound as a light-yellow solid in 59% yield (63 mg, 0.2 mmol).
^1^H NMR (300 MHz, CDCl_3_) *δ*: 7.52 (dd, *J* = 4.5, 1.5 Hz, 2H, arom H), 8.37 (d, *J* = 2.1 Hz, 1H, arom H), 8.70 (d, *J* = 5.9 Hz, 2H, arom H), 8.85 (d, *J* = 2.1 Hz, 1H, arom H) ppm. HRMS *m*/*z* [M+H]^+^ calcd for C_13_H_6_BrN_3_S 315.9539, found 315.9532.
**6-Bromo-3-(pyridin-2-ylethynyl)isothiazolo[4,3-*b*]pyridine (6y)**
This compound was obtained from 3,6-dibromoisothiazolo[4,3-b]pyridine (101 mg, 0.34 mmol, 1.0 eq.), 2-ethynylpyridine (105 mg, 1.02 mmol, 3 eq.), Pd(PPh_3_)_2_Cl_2_ (5 mg, 0.007 mmol, 0.02 eq.) and CuI (0.6 mg, 0.003 mmol, 0.01 eq.) using the general Sonogashira coupling procedure. The crude residue was purified by flash chromatography using a mixture of hexane and ethyl acetate (in a ratio of 7:3) as mobile phase, affording the title compound as a light-yellow solid in 74% yield (79 mg, 0.25 mmol).
^1^H NMR (300 MHz, CDCl_3_) *δ*: 7.30–7.38 (m, 1H, arom H), 7.68–7.80 (m, 2H, arom H), 8.35 (d, *J* = 2.1 Hz, 1H, arom H), 8.70 (d, *J* = 4.8 Hz, 1H, arom H), 8.84 (d, *J* = 2.1 Hz, 1H, arom H) ppm.
HRMS *m*/*z* [M+H]^+^ calcd for C_13_H_6_BrN_3_S 315.9539, found 315.9535.
***tert*-Butyl (3-(6-bromoisothiazolo[4,3-*b*]pyridin-3-yl)prop-2-yn-1-yl)carbamate (8)**
This compound was obtained from 3,6-dibromoisothiazolo[4,3-*b*]pyridine (101 mg, 0.34 mmol, 1.0 eq.), *N*-Boc-propargylamine (158 mg, 1.02 mmol, 3 eq.), Pd(PPh_3_)_2_Cl_2_ (5 mg, 0.007 mmol, 0.02 eq.) and CuI (0.6 mg, 0.003 mmol, 0.01 eq.) using the general Sonogashira coupling procedure. The crude residue was purified by flash chromatography using a mixture of hexane and ethyl acetate (in a ratio of 4:1) as mobile phase, affording the title compound as a light-orange solid in 89% yield (111.2 mg, 0.3 mmol).
^1^H NMR (300 MHz, CDCl_3_) *δ*: 1.47 (s, 9H, 3 × CH_3_), 4.40 (d, *J* = 5.1 Hz, 2H, CH_2_), 4.93 (bs, 1H, NH), 8.32 (d, *J* = 2.0 Hz, 1H, arom H), 8.79 (d, *J* = 2.0 Hz, 1H, arom H) ppm. HR-MS *m*/*z* [M+H]^+^ calcd for C_14_H_14_BrN_3_O_2_S 368.0063, found 368.0065.
**Suzuki coupling at position 6 of the isothiazolo[4,3-*b*]pyridine scaffold**
General procedure
A solution of the appropriate 3-substituted-6-bromoisothiazolo[4,3-*b*]pyridine analogue (1 eq.) in a mixture of dioxane/water (ratio 9:1) was degassed with argon and subsequently, the corresponding boronic acid or ester (1.2 eq.), Pd(PPh_3_)_4_ (0.02 eq.) and K_2_CO_3_ (2 eq.) were added. The mixture was degassed a second time, filled with argon and stirred at 90 °C overnight. After completion of the reaction as monitored by TLC, the reaction mixture was cooled down to room temperature and the volatiles were evaporated to dryness. The resulting residue was purified by silica gel flash chromatography and precipitated with diethyl ether yielding the title compounds. Compounds **3a-p**, **7a-y** were synthesized according to this procedure.
**6-(3-Methoxyphenyl)-3-(pyridin-3-ylethynyl)isothiazolo[4,3-*b*]pyridine (3a)**
This compound was obtained from the precursor **2b** (51 mg, 0.16 mmol, 1.0 eq.), 3-methoxyphenylboronic acid (29 mg, 0.19 mmol, 1.2 eq.), Pd(PPh_3_)_4_ (5 mg, 0.004 mmol, 0.027 eq.) and K_2_CO_3_ (45 mg, 0.32 mmol, 2 eq.) using general Suzuki coupling procedure. The crude residue was purified by flash chromatography using a mixture of hexane and acetone (in a ratio of 7:3) as mobile phase, affording the title compound as a light yellow solid in 69% yield (37.4 mg, 0.11 mmol).
^1^H NMR (600 MHz, CDCl_3_) *δ*: 3.91 (s, 3H, OCH_3_), 7.03 (ddd, *J* = 8.3, 2.6, 0.8 Hz, 1H, arom H), 7.21–7.23 (m, 1H, arom H), 7.28–7.30 (m, 1H, arom H), 7.37 (ddd, *J* = 7.9, 4.9, 0.7 Hz, 1H, arom H), 7.45–7.49 (m, 1H, arom H), 7.98 (dt, *J* = 7.9, 1.9 Hz, 1H, arom H), 8.27 (d, *J* = 2.1 Hz, 1H, arom H), 8.65 (dd, *J* = 4.7, 1.1 Hz, 1H, arom H), 8.93 (d, *J* = 0.9 Hz, 1H, arom H), 9.15 (d, *J* = 2.1 Hz, 1H, arom H) ppm.
^13^C NMR (150 MHz, CDCl_3_) *δ*: 55.41 (OCH_3_), 80.02 (C triple bond), 104.15 (C triple bond), 113.39 (CH), 114.19 (CH), 119.23 (C), 119.96 (CH), 123.13 (CH), 126.44 (CH), 130.45 (CH), 136.44 (C), 138.12 (C), 138.69 (CH), 143.46 (C), 148.02 (C), 149.81 (CH), 152.33 (CH), 152.44 (CH), 155.12 (C), 160.28 (C) ppm.
HRMS *m*/*z* [M+H]^+^ calcd for C_20_H_13_N_3_OS 344.0852, found 344.0835.
**6-(2-Methoxyphenyl)-3-(pyridin-3-ylethynyl)isothiazolo[4,3-*b*]pyridine (3b)**
This compound was obtained from the precursor **2b** (51 mg, 0.16 mmol, 1.0 eq.), 2-methoxyphenylboronic acid (29 mg, 0.19 mmol, 1.2 eq.), Pd(PPh_3_)_4_ (5 mg, 0.004 mmol, 0.027 eq.) and K_2_CO_3_ (45 mg, 0.32 mmol, 2 eq.) using general Suzuki coupling procedure. The crude residue was purified by flash chromatography using a mixture of hexane and ethyl acetate (in a ratio of 1:1) as mobile phase, affording the title compound as a light yellow solid in 74% yield (40.2 mg, 0.12 mmol).
^1^H NMR (600 MHz, CDCl_3_) *δ*: 3.86 (s, 3H, OCH_3_), 7.06 (d, *J* = 8.2 Hz, 1H, arom H), 7.12 (td, *J* = 7.5, 0.9 Hz, 1H, arom H), 7.36 (ddd, *J* = 7.9, 5.0, 0.5 Hz, 1H, arom H), 7.41–7.48 (m, 2H, arom H), 7.98 (dt, *J* = 7.9, 1.9 Hz, 1H, arom H), 8.21 (d, *J* = 2.0 Hz, 1H, arom H), 8.64 (d, *J* = 3.7 Hz, 1H, arom H), 8.92 (s, 1H, arom H), 9.09 (d, *J* = 1.9 Hz, 1H, arom H) ppm.
^13^C NMR (150 MHz, CDCl_3_) *δ*: 55.53 (OCH_3_), 80.16 (C triple bond), 103.74 (C triple bond), 111.37 (CH), 119.33 (C), 121.32 (CH), 123.11 (CH), 125.95 (C), 128.57 (CH), 130.46 (CH), 130.85 (CH), 134.75 (C), 138.68 (CH), 142.84 (C), 147.40 (C), 149.71 (CH), 152.32 (CH), 154.54 (CH), 155.34 (C), 156.72 (C) ppm.
HRMS *m*/*z* [M+H]^+^ calcd for C_20_H_13_N_3_OS 344.0852, found 344.0875.
**3-(Pyridin-3-ylethynyl)-6-(2-tolyl)isothiazolo[4,3-*b*]pyridine (3c)**
This compound was obtained from the precursor **2b** (51 mg, 0.16 mmol, 1.0 eq.), 2-tolylboronic acid (26 mg, 0.19 mmol, 1.2 eq.), Pd(PPh_3_)_4_ (5 mg, 0.004 mmol, 0.027 eq.) and K_2_CO_3_ (45 mg, 0.32 mmol, 2 eq.) using general Suzuki coupling procedure. The crude residue was purified by flash chromatography using a mixture of hexane and diethyl ether (in a ratio of 3:2) as mobile phase, affording the title compound as a light yellow solid in 81% yield (41.8 mg, 0.13 mmol).
^1^H NMR (600 MHz, CDCl_3_) *δ*: 2.35 (s, 3H, OCH_3_), 7.29–7.42 (m, 5H, arom H), 7.98 (dt, *J* = 7.9, 1.9 Hz, 1H, arom H), 8.08 (d, *J* = 2.0 Hz, 1H, arom H), 8.64 (dd, *J* = 4.9, 1.6 Hz, 1H, arom H), 8.89 (d, *J* = 2.0 Hz, 1H, arom H), 8.93 (d, *J* = 1.5 Hz, 1H, arom H) ppm. ^13^C NMR (150 MHz, CDCl_3_) *δ*: 20.42 (CH_3_), 79.99 (C triple bond), 104.13 (C triple bond), 119.20 (C), 123.12 (CH), 126.38 (CH), 128.66 (CH), 128.79 (CH), 129.93 (CH), 130.84 (CH), 135.74 (C), 136.82 (C), 137.57 (C), 138.69 (CH), 143.44 (C), 147.60 (C), 149.78 (CH), 152.30 (CH), 153.76 (CH), 154.87 (C) ppm.
HR-MS *m*/*z* [M+H]^+^ calcd for C_20_H_13_N_3_S 328.0903, found 328.0912.
***N*,*N*-Dimethyl-4-(3-(pyridin-3-ylethynyl)isothiazolo[4,3-*b*]pyridin-6-yl)aniline (3d)**
This compound was obtained from the precursor **2b** (51 mg, 0.16 mmol, 1.0 eq.), 4-(dimethylamino)phenylboronic acid (32 mg, 0.19 mmol, 1.2 eq.), Pd(PPh_3_)_4_ (5 mg, 0.004 mmol, 0.027 eq.) and K_2_CO_3_ (45 mg, 0.32 mmol, 2 eq.) using general Suzuki coupling procedure. The crude residue was purified by flash chromatography using a mixture of hexane and acetone (in a ratio of 4:1) as mobile phase and a second time using dichloromethane and methanol (10:0.2), affording the title compound as an orange solid in 74% yield (41.7 mg, 0.12 mmol).
^1^H NMR (600 MHz, CDCl_3_) *δ*: 3.07 (s, 6H, 2 ࠹NCH_3_), 6.84–6.91 (m, 2H, arom H), 7.38 (ddd, *J* = 7.9, 4.9, 0.7 Hz, 1H, arom H), 7.62–7.68 (m, 2H, arom H), 7.95–8.03 (m, 1H, arom H), 8.18 (d, *J* = 2.1 Hz, 1H, arom H), 8.65 (dd, *J* = 4.8, 1.2 Hz, 1H, arom H), 8.94 (s, 1H, arom H), 9.19 (d, *J* = 2.1 Hz, 1H, arom H) ppm.
^13^C NMR (150 MHz, CDCl_3_) *δ*: 40.28 (CH_3_), 80.26 (C triple bond), 103.64 (C triple bond), 112.74 (CH), 119.37 (C), 123.11 (CH), 123.63 (CH), 123.71 (C), 128.24 (CH), 136.49 (C), 138.68 (CH), 142.71 (C), 147.28 (C), 149.68 (CH), 150.86 (C), 152.31 (CH), 152.73 (CH), 155.72 (C) ppm.
HR-MS *m*/*z* [M+H]^+^ calcd for C_21_H_16_N_4_S 357.1168, found 357.1167.
**6-(4-Isopropoxyphenyl)-3-(pyridin-3-ylethynyl)isothiazolo[4,3-*b*]pyridine (3e)**
This compound was obtained from the precursor **2b** (51 mg, 0.16 mmol, 1.0 eq.), 4-isopropoxyphenylboronic acid (34 mg, 0.19 mmol, 1.2 eq.), Pd(PPh_3_)_4_ (5 mg, 0.004 mmol, 0.027 eq.) and K_2_CO_3_ (45 mg, 0.32 mmol, 2 eq.) using general Suzuki coupling procedure. The crude residue was purified by flash chromatography using a mixture of hexane and ethyl acetate (in a ratio of 7:3) as mobile phase, affording the title compound as a light yellow solid in 81% yield (47.5 mg, 0.13 mmol).
^1^H NMR (500 MHz, CDCl_3_) *δ*: 1.40 (s, 3H, CH_3_), 1.42 (s, 3H, CH_3_), 4.62–4.70 (hept, *J* = 6.0 Hz, 1H, CH), 7.06 (d, *J* = 8.7 Hz, 2H, arom H), 7.38 (dd, *J* = 7.9, 4.9 Hz, 1H, arom H), 7.65 (d, *J* = 8.7 Hz, 2H, arom H), 7.99 (dt, *J* = 7.9, 1.7 Hz, 1H, arom H), 8.21 (d, *J* = 2.0 Hz, 1H, arom H), 8.66 (d, *J* = 4.8 Hz, 1H, arom H), 8.94 (s, 1H, arom H), 9.16 (d, *J* = 2.0 Hz, 1H, arom H) ppm.
^13^C NMR (126 MHz, CDCl_3_) *δ*: 22.03 (CH_3_), 70.10 (CH), 80.15 (C triple bond), 103.99 (C triple bond), 116.57 (CH), 119.32 (C), 123.18 (CH), 125.16 (CH), 128.61 (C), 128.80 (CH), 136.24 (C), 138.76 (CH), 143.18 (C), 147.62 (C), 149.80 (CH), 152.34 (CH), 152.57 (CH), 155.40 (C), 158.84 (C) ppm.
HRMS *m*/*z* [M+H]^+^ calcd for C_22_H_17_N_3_OS 372.1165, found 372.1155.
**2-Methoxy-4-(3-(pyridin-3-ylethynyl)isothiazolo[4,3-*b*]pyridin-6-yl)aniline (3f)**
This compound was obtained from the precursor **2b** (51 mg, 0.16 mmol, 1.0 eq.), 4-amino-3-methoxyphenylboronic acid (32 mg, 0.19 mmol, 1.2 eq.), Pd(PPh_3_)_4_ (5 mg, 0.004 mmol, 0.027 eq.) and K_2_CO_3_ (45 mg, 0.32 mmol, 2 eq.) using general Suzuki coupling procedure. The crude residue was purified by flash chromatography using a mixture of hexane and acetone (in a ratio of 10:0.1) as mobile phase and a second time using dichloromethane and methanol (10:0.1), affording the title compound as a light yellow solid in 77% yield (43.6 mg, 0.12 mmol).
^1^H NMR (600 MHz, CDCl_3_) *δ*: 3.96 (s, 3H, OCH_3_), 4.08 (bs, 2H, NH_2_), 6.84 (d, *J* = 8.0 Hz, 1H, arom H), 7.11 (d, *J* = 1.9 Hz, 1H, arom H), 7.18 (dd, *J* = 8.0, 1.9 Hz, 1H, arom H), 7.36 (ddd, *J* = 7.9, 4.9, 0.7 Hz, 1H, arom H), 7.97 (dt, *J* = 7.9, 1.9 Hz, 1H, arom H), 8.16 (d, *J* = 2.1 Hz, 1H, arom H), 8.64 (dd, *J* = 4.9, 1.5 Hz, 1H, arom H), 8.92 (d, *J* = 1.4 Hz, 1H, arom H), 9.15 (d, *J* = 2.1 Hz, 1H, arom H) ppm.
^13^C NMR (150 MHz, CDCl_3_) *δ*: 55.59 (OCH_3_), 80.18 (C triple bond), 103.77 (C triple bond), 109.25 (CH), 114.97 (CH), 119.30 (C), 120.60 (CH), 123.10 (CH), 124.28 (CH), 126.38 (C), 136.77 (C), 137.51 (C), 138.66 (CH), 142.87 (C), 147.41 (C), 147.67 (C), 149.70 (CH), 152.29 (CH), 152.69 (CH), 155.52 (C) ppm.
HRMS *m*/*z* [M+H]^+^ calcd for C_20_H_14_N_4_OS 359.0961, found 359.0965.
**6-(4-Fluorophenyl)-3-(pyridin-3-ylethynyl)isothiazolo[4,3-*b*]pyridine (3g)**
This compound was obtained from the precursor **2b** (51 mg, 0.16 mmol, 1.0 eq.), 4-fluorophenylboronic acid (27 mg, 0.19 mmol, 1.2 eq.), Pd(PPh_3_)_4_ (5 mg, 0.004 mmol, 0.027 eq.) and K_2_CO_3_ (45 mg, 0.32 mmol, 2 eq.) using general Suzuki coupling procedure. The crude residue was purified by flash chromatography using a mixture of hexane and ethyl acetate (in a ratio of 1:1) as mobile phase, affording the title compound as a light yellow solid in 81% yield (42.4 mg, 0.13 mmol).
^1^H NMR (600 MHz, CDCl_3_) *δ*: 7.22–7.28 (m, 2H, arom H), 7.37 (ddd, *J* = 7.9, 4.9, 0.8 Hz, 1H, arom H), 7.65–7.71 (m, 2H, arom H), 7.98 (dt, *J* = 7.9, 1.9 Hz, 1H, arom H), 8.23 (d, *J* = 2.1 Hz, 1H, arom H), 8.65 (dd, *J* = 4.9, 1.6 Hz, 1H, arom H), 8.93 (d, *J* = 1.5 Hz, 1H, arom H), 9.11 (d, *J* = 2.1 Hz, 1H, arom H) ppm.
^13^C NMR (150 MHz, CDCl_3_) *δ*: 79.95 (C triple bond), 104.27 (C triple bond), 116.48 (d, *J* = 21.8 Hz, CH), 119.20 (C), 123.24 (CH), 126.25 (CH), 129.36 (d, *J* = 8.3 Hz, CH), 132.85 (C), 135.57 (C), 138.70 (CH), 143.62 (C), 147.90 (C), 149.85 (CH), 152.12 (CH), 152.33 (CH), 155.00 (C), 163.36 (d, *J* = 249.6 Hz, C-F) ppm.
^19^F NMR (471 MHz, CDCl_3_) *δ*: -112.426 ppm.
HRMS *m*/*z* [M+H]^+^ calcd for C_19_H_10_FN_3_S 332.0652, found 332.0652.
**6-(2-Fluorophenyl)-3-(pyridin-3-ylethynyl)isothiazolo[4,3-*b*]pyridine (3h)**
This compound was obtained from the precursor **2b** (51 mg, 0.16 mmol, 1.0 eq.), 2-fluorophenylboronic acid (27 mg, 0.19 mmol, 1.2 eq.), Pd(PPh_3_)_4_ (5 mg, 0.004 mmol, 0.027 eq.) and K_2_CO_3_ (45 mg, 0.32 mmol, 2 eq.) using general Suzuki coupling procedure. The crude residue was purified by flash chromatography using a mixture of hexane and ethyl acetate (in a ratio of 1:1) as mobile phase, affording the title compound as a light-yellow solid in 72% yield (37.6 mg, 0.11 mmol).
^1^H NMR (600 MHz, CDCl_3_) *δ*: 7.24–7.28 (m, 1H, arom H), 7.33 (td, *J* = 7.5, 1.1 Hz, 1H, arom H), 7.37 (ddd, *J* = 7.9, 4.9, 0.8 Hz, 1H, arom H), 7.44–7.49 (m, 1H, arom H), 7.56 (td, *J* = 7.7, 1.7 Hz, 1H, arom H), 7.96–8.00 (m, 1H, arom H), 8.29 (dd, *J* = 2.0, 0.9 Hz, 1H, arom H), 8.65 (dd, *J* = 4.9, 1.6 Hz, 1H, arom H), 8.92–8.94 (d, *J* = 1.5 Hz, 1H, arom H), 9.08 (t, *J* = 2.1 Hz, 1H, arom H) ppm.
^13^C NMR (150 MHz, CDCl_3_) *δ*: 79.95 (C triple bond), 104.22 (C triple bond), 116.57 (d, *J* = 22.2 Hz, CH), 119.21 (C), 123.13 (CH), 124.64 (d, *J* = 13.5 Hz, C), 125.01 (d, *J* = 3.3 Hz, CH), 128.87 (d, *J* = 2.0 Hz, CH), 130.75 (d, *J* = 1.8 Hz, CH), 130.76 (d, *J* = 8.2 Hz, CH), 131.69 (C), 138.71 (CH), 143.59 (C), 147.84 (C), 149.82 (CH), 152.32 (CH), 152.98 (d, *J* = 3.5 Hz, CH), 154.82 (C), 159.96 (d, *J* = 249.6 Hz, C-F) ppm. ^19^F NMR (471 MHz, CDCl_3_) *δ*: −116.62 ppm.
HRMS *m*/*z* [M+H]^+^ calcd for C_19_H_10_FN_3_S 332.06521, found 332.0646.
**6-(3-Chlorophenyl)-3-(pyridin-3-ylethynyl)isothiazolo[4,3-*b*]pyridine (3i)**
This compound was obtained from the precursor **2b** (51 mg, 0.16 mmol, 1.0 eq.), 3-chlorophenylboronic acid (30 mg, 0.19 mmol, 1.2 eq.), Pd(PPh_3_)_4_ (5 mg, 0.004 mmol, 0.027 eq.) and K_2_CO_3_ (45 mg, 0.32 mmol, 2 eq.) using general Suzuki coupling procedure. The crude residue was purified by flash chromatography using a mixture of hexane and ethyl acetate (in a ratio of 1:1) as mobile phase, affording the title compound as a light yellow solid in 79% yield (43.2 mg, 0.12 mmol).
^1^H NMR (600 MHz, CDCl_3_) *δ*: 7.36–7.39 (m, 1H, arom H), 7.44–7.51 (m, 2H, arom H), 7.58 (dt, *J* = 7.2, 1.6 Hz, 1H, arom H), 7.69 (t, *J* = 1.6 Hz, 1H, arom H), 7.98 (dt, *J* = 7.9, 1.9 Hz, 1H, arom H), 8.26 (d, *J* = 2.1 Hz, 1H, arom H), 8.65 (dd, *J* = 4.8, 1.5 Hz, 1H, arom H), 8.93 (d, *J* = 1.4 Hz, 1H, arom H), 9.10 (d, *J* = 2.1 Hz, 1H, arom H) ppm.
^13^C NMR (150 MHz, CDCl_3_) *δ*: 79.90 (C triple bond), 104.39 (C triple bond), 119.16 (C), 123.15 (CH), 125.75 (CH), 126.78 (CH), 127.67 (CH), 128.98 (CH), 130.62 (CH), 135.19 (C), 135.35 (C), 138.56 (C), 138.71 (CH), 143.79 (C), 148.16 (C), 149.86 (CH), 151.80 (CH), 152.32 (CH), 154.81 (C) ppm.
HRMS *m*/*z* [M+H]^+^ calcd for C_19_H_10_ClN_3_S 348.0357, found 348.0362.
**6-(4-(2-Methoxyethoxy)phenyl)-3-(pyridin-3-ylethynyl)isothiazolo[4,3-*b*]pyridine (3j)**
This compound was obtained from the precursor **2b** (51 mg, 0.16 mmol, 1.0 eq.), 4-(2-methoxyethoxy)phenylboronic acid (37 mg, 0.19 mmol, 1.2 eq.), Pd(PPh_3_)_4_ (5 mg, 0.004 mmol, 0.027 eq.) and K_2_CO_3_ (45 mg, 0.32 mmol, 2 eq.) using general Suzuki coupling procedure. The crude residue was purified by flash chromatography using a mixture of hexane and acetone (in a ratio of 4:1) as mobile phase, affording the title compound as a light yellow solid in 79% yield (48.4 mg, 0.12 mmol).
^1^H NMR (500 MHz, CDCl_3_) *δ*: 3.48 (s, 3H, OCH_3_), 3.76–3.83 (m, 2H, OCH_2_), 4.18–4.22 (m, 2H, OCH_2_), 7.10 (d, *J* = 8.7 Hz, 2H, arom H), 7.36 (dd, *J* = 7.9, 4.9 Hz, 1H, arom H), 7.64 (d, *J* = 8.7 Hz, 2H, arom H), 7.97 (dt, *J* = 7.9, 1.8 Hz, 1H, arom H), 8.19 (d, *J* = 2.0 Hz, 1H, arom H), 8.64 (dd, *J* = 4.9, 1.4 Hz, 1H, arom H), 8.92 (d, *J* = 1.4 Hz, 1H, arom H), 9.13 (d, *J* = 2.0 Hz, 1H, arom H) ppm.
^13^C NMR (126 MHz, CDCl_3_) *δ*: 59.31 (OCH_3_), 67.48 (OCH_2_), 70.95 (OCH_2_), 80.15 (C triple bond), 104.01 (C triple bond), 115.53 (CH), 119.29 (C), 123.17 (CH), 125.27 (CH), 128.74 (CH), 129.16 (C), 136.10 (C), 138.73 (CH), 143.20 (C), 147.64 (C), 149.80 (CH), 152.34 (CH), 152.48 (CH), 155.33 (C), 159.65 (C) ppm.
HRMS *m*/*z* [M+H]^+^ calcd for C_22_H_17_N_3_O_2_S 388.1114, found 388.1115.
**Methyl 2-methoxy-4-(3-(pyridin-3-ylethynyl)isothiazolo[4,3-*b*]pyridin-6-yl)benzoate (3k)**
This compound was obtained from the precursor **2b** (51 mg, 0.16 mmol, 1.0 eq.), 3-methoxy-4-methoxycarbonylphenylboronic acid (40 mg, 0.19 mmol, 1.2 eq.), Pd(PPh_3_)_4_ (5 mg, 0.004 mmol, 0.027 eq.) and K_2_CO_3_ (45 mg, 0.32 mmol, 2 eq.) using general Suzuki coupling procedure and DMF as solvent. The reaction mixture was stirred at 120 °C overnight. The residue was purified by precipitation using subsequently, methanol, dichloromethane and diethyl ether, affording the title compound as a light yellow solid in 75% yield (47.6 mg, 0.12 mmol).
^1^H NMR (300 MHz, DMSO) *δ*: 3.83 (s, 1H, OCH_3_), 3.98 (s, 1H, OCH_3_), 7.53–7.61 (m, 2H, arom H), 7.65 (s, 1H, arom H), 7.81 (d, *J* = 8.0 Hz, 1H, arom H), 8.12–8.18 (m, 1H, arom H), 8.70 (d, *J* = 4.8 Hz, 1H, arom H), 8.73 (d, *J* = 1.7 Hz, 1H, arom H), 8.91 (s, 1H, arom H), 9.34 (d, *J* = 1.7 Hz, 1H, arom H) ppm.
HRMS *m*/*z* [M+H]^+^ calcd for C_22_H_15_N_3_O_3_S 402.0907, found 402.0903.
**4-(3-(Pyridin-3-ylethynyl)isothiazolo[4,3-*b*]pyridin-6-yl)phenol (3l)**
This compound was obtained from the precursor **2b** (51 mg, 0.16 mmol, 1.0 eq.), 4-hydroxyphenylboronic acid (26 mg, 0.19 mmol, 1.2 eq.), Pd(PPh_3_)_4_ (5 mg, 0.004 mmol, 0.027 eq.) and K_2_CO_3_ (45 mg, 0.32 mmol, 2 eq.) using general Suzuki coupling procedure. The crude residue was purified by flash chromatography using a mixture of hexane and acetone (in a ratio of 7:3) as mobile phase, affording the title compound as a light yellow solid in 81% yield (42.1 mg, 0.13 mmol).
^1^H NMR (500 MHz, DMSO) *δ*: 6.92–6.97 (m, 2H, arom H), 7.55 (dd, *J* = 7.9, 4.9 Hz, 1H, arom H), 7.75–7.80 (m, 2H, arom H), 8.14 (dt, *J* = 7.9, 1.9 Hz, 1H, arom H), 8.40 (d, *J* = 2.1 Hz, 1H, arom H), 8.69 (dd, *J* = 4.9, 1.5 Hz, 1H, arom H), 8.90 (d, *J* = 2.0 Hz, 1H, arom H), 9.25 (d, *J* = 2.0 Hz, 1H, arom H), 9.89 (s, 1H, OH) ppm.
^13^C NMR (126 MHz, DMSO) *δ*: 80.34 (C triple bond), 103.59 (C triple bond), 116.24 (CH), 118.45 (C), 123.84 (CH), 123.92 (CH), 126.45 (C), 129.04 (CH), 135.75 (C), 138.88 (CH), 142.00 (C), 147.19 (C), 150.24 (CH), 151.67 (CH), 152.61 (CH), 155.16 (C), 158.65 (C) ppm.
HRMS *m*/*z* [M+H]^+^ calcd for C_19_H_11_N_3_OS 330.0696, found 330.0685.
**4-(3-(Pyridin-3-ylethynyl)isothiazolo[4,3-*b*]pyridin-6-yl)benzamide (3m)**
This compound was obtained from the precursor **2b** (51 mg, 0.16 mmol, 1.0 eq.), 4-aminocarbonylphenylboronic acid (32 mg, 0.19 mmol, 1.2 eq.), Pd(PPh_3_)_4_ (5 mg, 0.004 mmol, 0.027 eq.) and K_2_CO_3_ (45 mg, 0.32 mmol, 2 eq.) using general Suzuki coupling procedure. The crude residue was purified by flash chromatography using a mixture of dichloromethane and methanol (in a ratio of 10:0.3) as mobile phase, affording the title compound as a beige solid in 78% yield (44.3 mg, 0.12 mmol).
^1^H NMR (600 MHz, DMSO) *δ*: 7.50 (s, 1H, NH), 7.57 (ddd, *J* = 7.9, 4.9, 0.8 Hz, 1H, arom H), 8.03–8.08 (m, 4H), 8.12 (s, 1H, NH), 8.15–8.18 (m, 1H, arom H), 8.66 (d, *J* = 2.1 Hz, 1H, arom H), 8.70 (dd, *J* = 4.9, 1.6 Hz, 1H, arom H), 8.92 (dd, *J* = 2.1, 0.7 Hz, 1H, arom H), 9.34 (d, *J* = 2.1 Hz, 1H, arom H) ppm.
^13^C NMR (150 MHz, DMSO) *δ*: 80.25 (C triple bond), 103.95 (C triple bond), 118.42 (C), 123.96 (CH), 126.40 (CH), 127.64 (CH), 128.42 (CH), 134.47 (C), 134.92 (C), 138.64 (C), 138.93 (CH), 142.54 (C), 147.90 (C), 150.32 (CH), 151.71 (CH), 152.38 (CH), 154.76 (C), 167.35 (C) ppm.
HRMS *m*/*z* [M+H]^+^ calcd for C_20_H_12_N_4_OS 357.0805, found 357.0790.
***N*-Ethyl-4-(3-(pyridin-3-ylethynyl)isothiazolo[4,3-*b*]pyridin-6-yl)benzamide (3n)**
This compound was obtained from the precursor **2b** (51 mg, 0.16 mmol, 1.0 eq.), 4-(*N*-ethylaminocarbonyl)phenylboronic acid (37 mg, 0.19 mmol, 1.2 eq.), Pd(PPh_3_)_4_ (5 mg, 0.004 mmol, 0.027 eq.) and K_2_CO_3_ (45 mg, 0.32 mmol, 2 eq.) using general Suzuki coupling procedure. The crude residue was purified by flash chromatography using a mixture of hexane and acetone (in a ratio of 7:3) as mobile phase, affording the title compound as a light yellow solid in 75% yield (45.5 mg, 0.12 mmol).
  ^1^H NMR (600 MHz, CDCl_3_) *δ*: 1.30 (t, *J* = 7.3 Hz, 3H, CH_3_), 3.51–3.56 (m, 2H, CH_2_), 6.26 (t, *J* = 5.1 Hz, 1H, NH), 7.38 (dd, *J* = 7.8, 4.9 Hz, 1H, arom H), 7.75–7.79 (m, 2H, arom H), 7.93–7.97 (m, 2H, arom H), 7.98 (dt, *J* = 7.9, 1.9 Hz, 1H, arom H), 8.30 (d, *J* = 2.1 Hz, 1H, arom H), 8.65 (d, *J* = 4.0 Hz, 1H, arom H), 8.93 (s, 1H, arom H), 9.14 (d, *J* = 2.1 Hz, 1H, arom H) ppm.
^13^C NMR (150 MHz, CDCl_3_) *δ*: 14.90 (CH_3_), 35.06 (CH_2_), 79.91 (C triple bond), 104.39 (C triple bond), 119.17 (C), 123.19 (CH), 126.82 (CH), 127.71 (CH), 127.91 (CH), 135.07 (C), 135.43 (C), 138.71 (CH), 139.55 (C), 143.77 (C), 148.17 (C), 149.85 (CH), 151.86 (CH), 152.30 (CH), 154.85 (C), 166.59 (C) ppm.
HRMS *m*/*z* [M+H]^+^ calcd for C_22_H_16_N_4_OS 385.1118, found 385.1112.
***N*,*N*-Dimethyl-4-(3-(pyridin-3-ylethynyl)isothiazolo[4,3-*b*]pyridin-6-yl)benzamide (3o)**
This compound was obtained from the precursor **2b** (51 mg, 0.16 mmol, 1.0 eq.), 4-(*N*,*N*-dimethylaminocarbonyl)phenylboronic acid (37 mg, 0.19 mmol, 1.2 eq.), Pd(PPh_3_)_4_ (5 mg, 0.004 mmol, 0.027 eq.) and K_2_CO_3_ (45 mg, 0.32 mmol, 2 eq.) using general Suzuki coupling procedure. The crude residue was purified by flash chromatography using a mixture of hexane and acetone (in a ratio of 3:2) as mobile phase, affording the title compound as a light yellow solid in 79% yield (48.0 mg, 0.12 mmol).
^1^H NMR (600 MHz, CDCl_3_) *δ*: 3.06 (s, 3H, CH_3_), 3.16 (s, 3H, CH_3_), 7.37 (dd, *J* = 7.8, 4.9 Hz, 1H, arom H), 7.61 (d, *J* = 8.2 Hz, 2H, arom H), 7.75 (d, *J* = 8.2 Hz, 2H, arom H), 7.99 (dt, *J* = 7.9, 1.8 Hz, 1H, arom H), 8.29 (d, *J* = 2.0 Hz, 1H, arom H), 8.65 (dd, *J* = 4.8, 1.5 Hz, 1H, arom H), 8.93 (d, *J* = 1.5 Hz, 1H, arom H), 9.15 (d, *J* = 2.0 Hz, 1H, arom H) ppm.
^13^C NMR (150 MHz, CDCl_3_) *δ*: 35.39 (CH_3_), 39.56 (CH_3_), 79.91 (C triple bond), 104.32 (C triple bond), 119.16 (C), 123.14 (CH), 126.66 (CH), 127.58 (CH), 128.15 (CH), 135.69 (C), 136.82 (C), 137.85 (C), 138.69 (CH), 143.68 (C), 148.09 (C), 149.83 (CH), 151.99 (CH), 152.29 (CH), 154.91 (C), 170.76 (C) ppm.
HRMS *m*/*z* [M+H]^+^ calcd for C_22_H_16_N_4_OS 385.1118, found 385.1108.
**(4-(3-(Pyridin-3-ylethynyl)isothiazolo[4,3-*b*]pyridin-6-yl)phenyl)(pyrrolidin-1-yl)methanone (3p)**
This compound was obtained from the precursor **2b** (51 mg, 0.16 mmol, 1.0 eq.), 4-(pyrrolidine-1-carbonyl)phenylboronic acid (42 mg, 0.19 mmol, 1.2 eq.), Pd(PPh_3_)_4_ (5 mg, 0.004 mmol, 0.027 eq.) and K_2_CO_3_ (45 mg, 0.32 mmol, 2 eq.) using general Suzuki coupling procedure. The crude residue was purified by flash chromatography using a mixture of hexane and acetone (in a ratio of 3:2) as mobile phase, affording the title compound as a light yellow solid in 81% yield (52.5 mg, 0.13 mmol).
^1^H NMR (600 MHz, CDCl_3_) *δ* 1.93 (p, *J* = 6.6 Hz, 2H, CH_2_), 1.96–2.05 (m, 2H, CH_2_), 3.51 (t, *J* = 6.6 Hz, 2H, CH_2_), 3.70 (t, *J* = 7.0 Hz, 2H, CH_2_), 7.37 (ddd, *J* = 7.9, 4.9, 0.7 Hz, 1H, arom H), 7.71 (d, *J* = 8.4 Hz, 2H, arom H), 7.75 (d, *J* = 8.4 Hz, 2H, arom H), 7.99 (dt, *J* = 7.9, 1.9 Hz, 1H, arom H), 8.29 (d, *J* = 2.1 Hz, 1H, arom H), 8.65 (dd, *J* = 4.9, 1.6 Hz, 1H, arom H), 8.93 (d, *J* = 1.4 Hz, 1H, arom H), 9.15 (d, *J* = 2.1 Hz, 1H, arom H) ppm.
^13^C NMR (150 MHz, CDCl_3_) *δ*: 24.41 (CH_2_), 26.40 (CH_2_), 46.27 (CH_2_), 49.61 (CH_2_), 79.91 (C triple bond), 104.30 (C triple bond), 119.15 (C), 123.13 (CH), 126.65 (CH), 127.46 (CH), 128.19 (CH), 135.69 (C), 137.64 (C), 138.04 (C), 138.69 (CH), 143.66 (C), 148.09 (C), 149.82 (CH), 151.99 (CH), 152.28 (CH), 154.91 (C), 168.80 (C) ppm.
HRMS *m*/*z* [M+H]^+^ calcd for C_24_H_18_N_4_OS 411.1274, found 411.1271.
***N*,*N*-Dimethyl-3-(3-(pyridin-3-ylethynyl)isothiazolo[4,3-*b*]pyridin-6-yl)benzamide (3q)**
This compound was obtained from the precursor **2b** (51 mg, 0.16 mmol, 1.0 eq.), 3-(*N*,*N*-dimethylaminocarbonyl)phenylboronic acid (37 mg, 0.19 mmol, 1.2 eq.), Pd(PPh_3_)_4_ (5 mg, 0.004 mmol, 0.027 eq.) and K_2_CO_3_ (45 mg, 0.32 mmol, 2 eq.) using general Suzuki coupling procedure. The crude residue was purified by flash chromatography using a mixture of hexane and acetone (in a ratio of 3:2) as mobile phase, affording the title compound as a light yellow solid in 76% yield (46.2 mg, 0.12 mmol).
^1^H NMR (600 MHz, CDCl_3_) *δ*: 3.06 (s, 3H, CH_3_), 3.17 (s, 3H, CH_3_), 7.37 (ddd, *J* = 7.9, 4.9, 0.7 Hz, 1H, arom H), 7.53 (dt, *J* = 7.6, 1.1 Hz, 1H, arom H), 7.59 (t, *J* = 7.7 Hz, 1H, arom H), 7.76–7.72 (m, 1H, arom H), 7.77–7.78 (m, 1H, arom H), 7.99 (dt, *J* = 7.9, 1.9 Hz, 1H, arom H), 8.29 (d, *J* = 2.1 Hz, 1H, arom H), 8.65 (dd, *J* = 4.9, 1.6 Hz, 1H, arom H), 8.93 (d, *J* = 1.4 Hz, 1H, arom H), 9.14 (d, *J* = 2.1 Hz, 1H, arom H) ppm.
^13^C NMR (150 MHz, CDCl_3_) *δ*: 35.41 (CH_3_), 39.61 (CH_3_), 79.93 (C triple bond), 104.30 (C triple bond), 119.17 (C), 123.13 (CH), 126.26 (CH), 126.69 (CH), 127.41 (CH), 128.62 (CH), 129.42 (CH), 135.74 (C), 137.09 (C), 137.57 (C), 138.70 (CH), 143.67 (C), 148.07 (C), 149.82 (CH), 151.99 (CH), 152.29 (CH), 154.90 (C), 170.79 (C) ppm.
HRMS *m*/*z* [M+H]^+^ calcd for C_22_H_16_N_4_OS 385.1118, found 385.1118.
**(3-(3-(Pyridin-3-ylethynyl)isothiazolo[4,3-*b*]pyridin-6-yl)phenyl)(pyrrolidin-1-yl)methanone (3r)**
This compound was obtained from the precursor **2b** (51 mg, 0.16 mmol, 1.0 eq.), 3-(pyrrolidine-1-carbonyl)phenylboronic acid pinacol ester (61 mg, 0.19 mmol, 1.2 eq.), Pd(PPh_3_)_4_ (5 mg, 0.004 mmol, 0.027 eq.) and K_2_CO_3_ (45 mg, 0.32 mmol, 2 eq.) using general Suzuki coupling procedure. The crude residue was purified by flash chromatography using a mixture of hexane and acetone (in a ratio of 3:2) as mobile phase, affording the title compound as a yellow solid in 69% yield (44.8 mg, 0.11 mmol).
  ^1^H NMR (600 MHz, CDCl_3_) *δ*: 1.89–1.96 (m, 2H, CH_2_), 1.98–2.04 (m, 2H, CH_2_), 3.51 (t, *J* = 6.6 Hz, 2H, CH_2_), 3.70 (t, *J* = 7.0 Hz, 2H, CH_2_), 7.37 (ddd, *J* = 7.9, 4.9, 0.8 Hz, 1H), 7.59 (t, *J* = 7.6 Hz, 1H, arom H), 7.62–7.65 (m, 1H, arom H), 7.74–7.77 (m, 1H, arom H), 7.88 (bs, 1H, arom H), 7.98 (dt, *J* = 7.9, 1.8 Hz, 1H, arom H), 8.29 (d, *J* = 2.0 Hz, 1H, arom H), 8.65 (dd, *J* = 4.9, 1.5 Hz, 1H, arom H), 8.93 (d, *J* = 1.4 Hz, 1H, arom H), 9.15 (d, *J* = 2.0 Hz, 1H, arom H) ppm.
  ^13^C NMR (150 MHz, CDCl_3_) *δ*: 24.40 (CH_2_), 26.37 (CH_2_), 46.28 (CH_2_), 49.65 (CH_2_), 79.93 (C triple bond), 104.27 (C triple bond), 119.15 (C), 123.12 (CH), 126.36 (CH), 126.64 (CH), 127.43 (CH), 128.81 (CH), 129.32 (CH), 135.79 (C), 136.91 (C), 138.40 (C), 138.69 (CH), 143.62 (C), 148.03 (C), 149.79 (CH), 152.01 (CH), 152.26 (CH), 154.89 (C), 168.85 (C) ppm.
HRMS *m*/*z* [M+H]^+^ calcd for C_24_H_18_N_4_OS 411.1274, found 411.1271.
***N*-(2-Methoxyethyl)-3-(3-(pyridin-3-ylethynyl)isothiazolo[4,3-*b*]pyridin-6-yl)benzamide (3s)**
This compound was obtained from the precursor **2b** (51 mg, 0.16 mmol, 1.0 eq.), 3-(2-methoxyethylaminocarbonyl)benzeneboronic acid pinacol ester (62 mg, 0.19 mmol, 1.2 eq.), Pd(PPh_3_)_4_ (5 mg, 0.004 mmol, 0.027 eq.) and K_2_CO_3_ (45 mg, 0.32 mmol, 2 eq.) using general Suzuki coupling procedure. The crude residue was purified by flash chromatography using a mixture of hexane and acetone (in a ratio of 3:2) as mobile phase, affording the title compound as a light yellow solid in 75% yield (49.1 mg, 0.12 mmol).
^1^H NMR (600 MHz, CDCl_3_) *δ*: 3.41 (s, 3H, OCH_3_), 3.61 (t, *J* = 5.0 Hz, 2H, CH_2_), 3.71 (dd, *J* = 10.3, 5.3 Hz, 2H, CH_2_), 6.70 (bs, 1H, NH), 7.37 (dd, *J* = 7.8, 4.9 Hz, 1H, arom H), 7.62 (t, *J* = 7.7 Hz, 1H, arom H), 7.83 (d, *J* = 7.8 Hz, 1H, arom H), 7.86 (d, *J* = 7.7 Hz, 1H, arom H), 7.96–8.02 (m, 1H, arom H), 8.16 (bs, 1H, arom H), 8.31 (d, *J* = 2.0 Hz, 1H, arom H), 8.65 (dd, *J* = 4.8, 1.3 Hz, 1H, arom H), 8.92 (d, *J* = 1.2 Hz, 1H, arom H), 9.15 (d, *J* = 2.0 Hz, 1H, arom H) ppm.
^13^C NMR (150 MHz, CDCl_3_) *δ*: 39.84 (CH_2_), 58.86 (OCH_3_), 71.07 (CH_2_), 79.94 (C triple bond), 104.33 (C triple bond), 119.18 (C), 123.14 (CH), 126.54 (CH), 126.80 (CH), 127.04 (CH), 129.58 (CH), 130.41 (CH), 135.67 (C), 135.76 (C), 137.28 (C), 138.71 (CH), 143.71 (C), 148.10 (C), 149.83 (CH), 151.98 (CH), 152.30 (CH), 154.88 (C), 166.82 (C) ppm.
HRMS *m*/*z* [M+H]^+^ calcd for C_23_H_18_N_4_O_2_S 415.1223, found 415.1223.
**6-(3,4-Dimethoxyphenyl)-3-(pent-1-yn-1-yl)isothiazolo[4,3-*b*]pyridine (7a)**
This compound was obtained from the precursor **2g** (49 mg, 0.17 mmol, 1.0 eq.), 3,4-dimethoxyphenylboronic acid (36 mg, 0.20 mmol, 1.2 eq.), Pd(PPh_3_)_4_ (5 mg, 0.004 mmol, 0.027 eq.) and K_2_CO_3_ (45 mg, 0.32 mmol, 2 eq.) using general Suzuki coupling procedure. The crude residue was purified by flash chromatography using a mixture of hexane and ethyl acetate (in a ratio of 3:2) as mobile phase and a second purification using a mixture of hexane and dichloromethane (in a ratio of 2:3), affording the title compound as a light-yellow solid in 74% yield (44.3 mg, 0.13 mmol).
^1^H NMR (600 MHz, CDCl_3_) *δ*: 1.11 (t, *J* = 7.4 Hz, 3H, CH_3_), 1.77 (h, *J* = 7.3 Hz, 2H, CH_2_), 2.67 (t, *J* = 7.1 Hz, 2H, CH_2_), 3.96 (s, 3H, OCH_3_), 3.98 (s, 3H, OCH_3_), 7.02 (d, *J* = 8.3 Hz, 1H, arom H), 7.18 (d, *J* = 2.1 Hz, 1H, arom H), 7.27 (dd, *J* = 8.2, 2.2 Hz, 1H, arom H), 8.16 (d, *J* = 2.1 Hz, 1H, arom H), 9.08 (d, *J* = 2.1 Hz, 1H, arom H) ppm.
^13^C NMR (150 MHz, CDCl_3_) *δ*: 13.69 (CH_3_), 21.80 (CH_2_), 22.59 (CH_2_), 56.05 (OCH_3_), 68.81 (C triple bond), 110.47 (CH), 110.93 (C triple bond), 111.76 (CH), 120.19 (CH), 125.38 (CH), 129.62 (C), 136.01 (C), 145.59 (C), 147.56 (C), 149.66 (C), 149.88 (C), 151.74 (CH), 155.15 (C) ppm.
HRMS *m*/*z* [M+H]^+^ calcd for C_19_H_18_N_2_O_2_S 339.1162, found 339.1162.
**6-(3,4-Dimethoxyphenyl)-3-(phenylethynyl)isothiazolo[4,3-b]pyridine (7b)**
This compound was prepared according to a procedure described in the literature [28].
**6-(3,4-Dimethoxyphenyl)-3-((4-fluorophenyl)ethynyl)isothiazolo[4,3-*b*]pyridine (7c)**
This compound was obtained from the precursor **6c** (50 mg, 0.15 mmol, 1.0 eq.), 3,4-dimethoxyphenylboronic acid (33 mg, 0.18 mmol, 1.2 eq.), Pd(PPh_3_)_4_ (5 mg, 0.004 mmol, 0.027 eq.) and K_2_CO_3_ (45 mg, 0.32 mmol, 2 eq.) using general Suzuki coupling procedure. The crude residue was purified by flash chromatography using a mixture of hexane and ethyl acetate (in a ratio of 7:3) as mobile phase, affording the title compound as a light yellow solid in 83% yield (48.5 mg, 0.12 mmol).
^1^H NMR (600 MHz, CDCl_3_) *δ*: 3.96 (s, 3H, OCH_3_), 3.99 (s, 3H, OCH_3_), 7.03 (d, *J* = 8.3 Hz, 1H, arom H), 7.09–7.14 (m, 2H, arom H), 7.19 (d, *J* = 2.1 Hz, 1H, arom H), 7.28 (dd, *J* = 8.3, 2.1 Hz, 1H, arom H), 7.66–7.70 (m, 2H, arom H), 8.19 (d, *J* = 2.1 Hz, 1H, arom H), 9.13 (d, *J* = 2.0 Hz, 1H, arom H) ppm.
^13^C NMR (150 MHz, CDCl_3_) *δ*: 56.04 (OCH_3_), 106.85 (C triple bond), 110.43 (CH), 111.76 (CH), 115.93 (d, *J* = 22.2 Hz, CH), 118.10 (C), 120.20 (CH), 125.35 (CH), 129.44 (C), 133.96 (d, *J* = 8.5 Hz, CH) 136.17 (C), 144.15 (C), 147.52 (C), 149.68 (C), 149.94 (C), 152.11 (CH), 155.23 (C), 163.28 (d, *J* = 252.1 Hz, C-F) ppm.
^19^F NMR (471 MHz, CDCl_3_) *δ*: −108.32 ppm.
HRMS *m*/*z* [M+H]^+^ calcd for C_22_H_15_FN_2_O_2_S 391.0911, found 391.0899.
**6-(3,4-Dimethoxyphenyl)-3-((3-chlorophenyl)ethynyl)isothiazolo[4,3-*b*]pyridine (7d)**
This compound was obtained from the precursor **6d** (50 mg, 0.14 mmol, 1.0 eq.), 3,4-dimethoxyphenylboronic acid (33 mg, 0.18 mmol, 1.2 eq.), Pd(PPh_3_)_4_ (5 mg, 0.004 mmol, 0.027 eq.) and K_2_CO_3_ (45 mg, 0.32 mmol, 2 eq.) using general Suzuki coupling procedure. The crude residue was purified by flash chromatography using a mixture of hexane and ethyl acetate (in a ratio of 7:3) as mobile phase, affording the title compound as a light yellow solid in 84% yield (49.2 mg, 0.12 mmol).
^1^H NMR (500 MHz, CDCl_3_) *δ*: 3.96 (s, 3H, OCH_3_), 3.99 (s, 3H, OCH_3_), 7.03 (d, *J* = 8.3 Hz, 1H arom H), 7.19 (d, *J* = 2.1 Hz, 1H, arom H), 7.28 (dd, *J* = 8.3, 2.1 Hz, 1H, arom H), 7.33–7.37 (m, 1H, arom H), 7.39–7.42 (m, 1H, arom H), 7.57 (dt, *J* = 7.5, 1.3 Hz, 1H, arom H), 7.69 (t, *J* = 1.6 Hz, 1H, arom H), 8.20 (d, *J* = 2.1 Hz, 1H, arom H), 9.13 (d, *J* = 2.1 Hz, 1H, arom H) ppm.
^13^C NMR (126 MHz, CDCl_3_) *δ*: 56.04 (OCH_3_), 78.03 (C triple bond), 106.09 (C triple bond), 110.42 (CH), 111.76 (CH), 120.21 (CH), 123.60 (C), 125.33 (CH), 129.37 (C), 129.72 (CH), 129.83 (CH), 129.93 (CH), 131.63 (CH), 134.40 (C), 136.22 (C), 143.62 (C), 147.64 (C), 149.67 (C), 149.96 (C), 152.28 (CH), 155.23 (C) ppm.
HRMS *m*/*z* [M+H]^+^ calcd for C_22_H_15_ClN_2_O_2_S 407.0615, found 407.0581.
**6-(3,4-Dimethoxyphenyl)-3-((2-fluorophenyl)ethynyl)isothiazolo[4,3-*b*]pyridine (7e)**
This compound was obtained from the precursor **6e** (50 mg, 0.14 mmol, 1.0 eq.), 3,4-dimethoxyphenylboronic acid (33 mg, 0.18 mmol, 1.2 eq.), Pd(PPh_3_)_4_ (5 mg, 0.004 mmol, 0.027 eq.) and K_2_CO_3_ (45 mg, 0.32 mmol, 2 eq.) using general Suzuki coupling procedure. The crude residue was purified by flash chromatography using a mixture of hexane and ethyl acetate (in a ratio of 7:3) as mobile phase, affording the title compound as a light yellow solid in 81% yield (47.4 mg, 0.12 mmol).
^1^H NMR (500 MHz, CDCl_3_) *δ*: 3.96 (s, 3H, OCH_3_), 3.99 (s, 3H, OCH_3_), 7.03 (d, *J* = 8.4 Hz, 1H, arom H), 7.14–7.22 (m, 1H), 7.28 (dd, *J* = 8.3, 2.2 Hz, 1H, arom H), 7.39–7.45 (m, 1H, arom H), 7.68 (td, *J* = 7.3, 1.7 Hz, 1H, arom H), 8.20 (d, *J* = 2.1 Hz, 1H, arom H), 9.14 (d, *J* = 2.1 Hz, 1H, arom H) ppm.
^13^C NMR (126 MHz, CDCl_3_) *δ*: 56.05 (OCH_3_), 81.67 (C triple bond), 101.02 (C triple bond), 110.46 (CH), 110.83 (d, *J* = 15.5 Hz, C), 111.79 (CH), 115.76 (d, *J* = 20.5 Hz, CH), 120.22 (CH), 124.12 (d, *J* = 3.3 Hz, CH), 125.30 (CH), 129.46 (C), 131.52 (d, *J* = 7.9 Hz, CH), 133.53 (CH), 136.18 (C), 143.73 (C), 147.69 (C), 149.68 (C), 149.95 (C), 152.30 (CH), 155.20 (C), 162.52 (d, *J* = 253.9 Hz, C-F) ppm.
^19^F NMR (471 MHz, CDCl_3_) *δ*: −107.77 ppm.
HRMS *m*/*z* [M+H]^+^ calcd for C_22_H_15_FN_2_O_2_S 391.0911, found 391.0899.
**6-(3,4-Dimethoxyphenyl)-3-((3-methoxyphenyl)ethynyl)isothiazolo[4,3-*b*]pyridine (7f)**
This compound was obtained from the precursor **6e** (50 mg, 0.14 mmol, 1.0 eq.), 3,4-dimethoxyphenylboronic acid (33 mg, 0.18 mmol, 1.2 eq.), Pd(PPh_3_)_4_ (5 mg, 0.004 mmol, 0.027 eq.) and K_2_CO_3_ (45 mg, 0.32 mmol, 2 eq.) using general Suzuki coupling procedure. This compound was obtained using the precursor **6f** and 3,4-dimethoxyphenylboronic acid. The crude residue was purified by flash chromatography using a mixture of hexane and ethyl acetate (in a ratio of 7:3) as mobile phase, affording the title compound as a yellow solid in 72% yield (41.9 mg, 0.10 mmol).
^1^H NMR (600 MHz, CDCl_3_) *δ*: 3.85 (s, 3H, OCH_3_), 3.96 (s, 3H, OCH_3_), 3.99 (s, 3H, OCH_3_), 6.99 (ddd, *J* = 8.1, 2.6, 1.3 Hz, 1H, arom H), 7.03 (d, *J* = 8.3 Hz, 1H, arom H), 7.19 (d, *J* = 2.1 Hz, 1H, arom H), 7.21 (dd, *J* = 2.6, 1.3 Hz, 1H, arom H), 7.27–7.30 (m, 2H, arom H), 7.32 (t, *J* = 7.8 Hz, 1H, arom H), 8.20 (d, *J* = 2.1 Hz, 1H, arom H), 9.13 (d, *J* = 2.1 Hz, 1H, arom H) ppm.
^13^C NMR (150 MHz, CDCl_3_) *δ*: 55.40 (OCH_3_), 56.04 (OCH_3_), 108.04 (C), 110.43 (CH), 111.77 (CH), 116.29 (CH), 116.57 (CH), 120.21 (CH), 122.87 (C), 124.52 (CH), 125.37 (CH), 129.47 (C), 129.57 (CH), 136.15 (C), 144.33 (C), 147.49 (C), 149.67 (C), 149.93 (C), 152.08 (CH), 155.23 (C), 159.36 (C) ppm.
  HR-MS *m*/*z* [M+H]^+^ calcd for C_23_H_18_N_2_O_3_S 403.1111, found 403.1093.
**6-(3,4-Dimethoxyphenyl)-3-((6-methylpyridin-3-yl)ethynyl)isothiazolo[4,3-*b*]pyridine (7g)**
This compound was obtained from the precursor **6g** (50 mg, 0.15 mmol, 1.0 eq.), 3,4-dimethoxyphenylboronic acid (33 mg, 0.18 mmol, 1.2 eq.), Pd(PPh_3_)_4_ (5 mg, 0.004 mmol, 0.03 eq.) and K_2_CO_3_ (42 mg, 0.3 mmol, 2 eq.) using general Suzuki coupling procedure. The crude residue was purified by flash chromatography using a mixture of hexane and acetone (in a ratio of 7:3) as mobile phase, affording the title compound as a yellow solid in 79% yield (46.3 mg, 0.12 mmol).
^1^H NMR (600 MHz, CDCl_3_) *δ*: 2.62 (s, 3H, CH_3_), 3.97 (s, 3H, OCH_3_), 3.99 (s, 3H, OCH_3_), 7.03 (d, *J* = 8.3 Hz, 1H, arom H), 7.19 (d, *J* = 2.1 Hz, 1H, arom H), 7.23 (d, *J* = 8.0 Hz, 1H, arom H), 7.29 (dd, *J* = 8.3, 2.2 Hz, 1H, arom H), 7.86 (dd, *J* = 8.0, 2.2 Hz, 1H, arom H), 8.21 (d, *J* = 2.1 Hz, 1H, arom H), 8.81 (d, *J* = 1.6 Hz, 1H, arom H), 9.14 (d, *J* = 2.1 Hz, 1H, arom H) ppm.
^13^C NMR (150 MHz, CDCl_3_) *δ*: 24.67 (CH_3_), 56.05 (OCH_3_), 79.43 (C), 104.66 (C), 110.42 (CH), 111.71 (CH), 116.14 (C), 120.22 (CH), 122.85 (CH), 125.38 (CH), 129.37 (C), 136.25 (C), 138.90 (CH), 143.65 (C), 147.57 (C), 149.66 (C), 149.97 (C), 151.71 (CH), 152.30 (CH), 155.23 (C), 159.28 (C) ppm.
HRMS *m*/*z* [M+H]^+^ calcd for C_22_H_17_N_3_O_2_S 388.1114, found 388.1111.
**6-(3,4-Dimethoxyphenyl)-3-((6-methoxypyridin-3-yl)ethynyl)isothiazolo[4,3-*b*]pyridine (7h)**
This compound was obtained from the precursor **6h** (48 mg, 0.14 mmol, 1.0 eq.), 3,4-dimethoxyphenylboronic acid (32 mg, 0.17 mmol, 1.2 eq.), Pd(PPh_3_)_4_ (5 mg, 0.004 mmol, 0.03 eq.) and K_2_CO_3_ (39 mg, 0.28 mmol, 2 eq.) using general Suzuki coupling procedure. The crude residue was purified by flash chromatography using a mixture of hexane and acetone (in a ratio of 8:2) as mobile phase and a second time using dichloromethane and methanol (10:0.1), affording the title compound as a yellow solid in 71% yield (41.3 mg, 0.10 mmol).
^1^H NMR (300 MHz, CDCl_3_) *δ*: 3.97 (s, 3H, OCH_3_), 3.99 (s, 3H, OCH_3_), 4.00 (s, 3H, OCH_3_), 6.80 (d, *J* = 8.6 Hz, 1H, arom H), 7.03 (d, *J* = 8.3 Hz, 1H, arom H), 7.19 (d, *J* = 1.9 Hz, 1H, arom H), 7.26–7.32 (m, 1H, arom H), 7.84 (dd, *J* = 2.3, 8.6 Hz, 1H, arom H), 8.20 (d, *J* = 1.9 Hz, 1H, arom H), 8.51 (d, *J* = 2.0 Hz, 1H, arom H), 9.12 (d, *J* = 2.0 Hz, 1H, arom H) ppm.
^13^C NMR (75 MHz, CDCl_3_) *δ*: 53.90 (OCH_3_), 56.08 (OCH_3_), 78.57 (C), 105.16 (C), 110.47 (CH), 111.01 (CH), 111.81 (CH), 111.92 (C), 120.25 (CH), 125.41 (CH), 129.46 (C), 136.23 (C), 141.27 (CH), 144.16 (C), 147.47 (C), 149.71 (C), 149.98 (C), 150.71 (CH), 152.13 (CH), 155.24 (C), 164.24 (C) ppm.
HRMS *m*/*z* [M+H]^+^ calcd for C_22_H_17_N_3_O_3_S 404.1063, found 404.1065.
**6-(3,4-Dimethoxyphenyl)-3-((5-methylpyridin-3-yl)ethynyl)isothiazolo[4,3-*b*]pyridine (7i)**
This compound was obtained from the precursor **6i** (50 mg, 0.15 mmol, 1.0 eq.), 3,4-dimethoxyphenylboronic acid (33 mg, 0.18 mmol, 1.2 eq.), Pd(PPh_3_)_4_ (5 mg, 0.004 mmol, 0.03 eq.) and K_2_CO_3_ (42 mg, 0.3 mmol, 2 eq.) using general Suzuki coupling procedure. The crude residue was purified by flash chromatography using a mixture of hexane and acetone (in a ratio of 7:3) as mobile phase, affording the title compound as a yellow solid in 81% yield (47.5 mg, 0.12 mmol).
^1^H NMR (300 MHz, CDCl_3_) *δ*: 2.39 (s, 3H, CH_3_), 3.97 (s, 3H, OCH_3_), 3.99 (s, 3H, OCH_3_), 7.04 (d, *J* = 8.3 Hz, 1H, arom H), 7.20 (d, *J* = 1.7 Hz, 1 H, arom H), 7.26–7.31 (m, 1H, arom H), 7.80 (s, 1H, arom H), 8.21 (d, *J* = 1.8 Hz, 1H), 8.47 (s, 1H, arom H), 8.73 (s, 1H, arom H), 9.14 (d, *J* = 1.8 Hz, 1H, arom H) ppm.
^13^C NMR (75 MHz, CDCl_3_) *δ*: 18.25 (CH_3_), 56.08 (OCH_3_), 79.79 (C), 104.42 (C), 110.47 (CH), 111.82 (CH), 118.68 (C), 120.27 (CH), 125.40 (CH), 129.38 (C), 132.88 (C), 136.32 (C), 139.09 (CH), 143.48 (C), 147.70 (C), 149.48 (CH), 149.73 (C), 150.02 (C), 150.50 (CH), 152.41 (CH), 155.28 (C) ppm.
HRMS *m*/*z* [M+H]^+^ calcd for C_22_H_17_N_3_O_2_S 388.1114, found 388.1115.
**6-(3,4-Dimethoxyphenyl)-3-((6-fluoropyridin-3-yl)ethynyl)isothiazolo[4,3-*b*]pyridine (7j)**
This compound was obtained from the precursor **6j** (50 mg, 0.15 mmol, 1.0 eq.), 3,4-dimethoxyphenylboronic acid (33 mg, 0.18 mmol, 1.2 eq.), Pd(PPh_3_)_4_ (5 mg, 0.004 mmol, 0.03 eq.) and K_2_CO_3_ (42 mg, 0.3 mmol, 2 eq.) using general Suzuki coupling procedure. The crude residue was purified by flash chromatography using a mixture of hexane and acetone (in a ratio of 9:1) as mobile phase, affording the title compound as a yellow solid in 75% yield (43.9 mg, 0.11 mmol).
^1^H NMR (500 MHz, CDCl_3_) *δ*: 3.97 (s, 3H, OCH_3_), 4.00 (s, 3H, OCH_3_), 7.00–7.05 (m, 2H, arom H), 7.19 (d, *J* = 1.8 Hz, 1H, arom H), 7.29 (dd, *J* = 8.3, 2.0 Hz, 1H, arom H), 8.04–8.10 (m, 1H, arom H), 8.22 (d, *J* = 2.0 Hz, 1H, arom H), 8.56 (s, 1H, arom H), 9.15 (d, *J* = 2.0 Hz, 1H, arom H) ppm. ^19^F NMR (282 MHz, CDCl_3_) *δ*: −63.82 ppm.
^13^C NMR (126 MHz, CDCl_3_) *δ*: 56.03 (OCH_3_), 79.94 (C), 102.56 (C), 109.73 (CH, d, *J* = 37.9 Hz), 110.34 (CH), 111.72 (CH), 117.13 (C), 120.22 (CH), 125.38 (CH), 129.23 (C), 136.28 (C), 142.98 (C), 143.85 (CH, d, *J* = 8.3 Hz), 147.67 (C), 149.66 (C), 149.97 (C), 151.03 (CH, d, *J* = 15.6 Hz), 152.51 (CH), 155.23 (C), 163.22 (CF, d, *J* = 244.3 Hz) ppm.
HRMS *m*/*z* [M+H]^+^ calcd for C_21_H_14_FN_3_O_2_S 392.0863, found 392.0870.
**Methyl 5-((6-(3,4-dimethoxyphenyl)isothiazolo[4,3-*b*]pyridin-3-yl)ethynyl)nicotinate (7k)**
This compound was obtained from the precursor **6k** (65 mg, 0.15 mmol, 1.0 eq.), 3,4-dimethoxyphenylboronic acid (33 mg, 0.18 mmol, 1.2 eq.), Pd(PPh_3_)_4_ (5 mg, 0.004 mmol, 0.03 eq.) and K_2_CO_3_ (42 mg, 0.3 mmol, 2 eq.) using general Suzuki coupling procedure. The crude residue was purified by flash chromatography using a mixture of hexane and acetone (in a ratio of 9:1) as mobile phase, affording the title compound as a light yellow solid in 76% yield (44.5 mg, 0.11 mmol).
^1^H NMR (300 MHz, CDCl_3_) *δ*: 3.97 (s, 3H, OCH_3_), 3.99 (s, 3H, OCH_3_), 7.03 (d, *J* = 8.3 Hz, 1H, arom H), 7.19 (d, *J* = 1.9 Hz, 1H, arom H), 7.24–7.32 (m, 2H, arom H), 8.04–8.13 (m, 1H, arom H), 8.21 (d, *J* = 2.0 Hz, 1H, arom H), 8.28 (d, *J* = 4.3 Hz, 1H, arom H), 9.15 (d, *J* = 1.9 Hz, 1H, arom H) ppm.
^19^F NMR (282 MHz, CDCl_3_) *δ* -61.97 ppm.
^13^C NMR (75 MHz, CDCl_3_) *δ*: 56.08 (OCH_3_), 83.12 (C), 98.68 (d, *J* = 5.1 Hz, C), 106.54 (d, *J* = 31.0 Hz, C), 110.45 (CH), 111.81 (CH), 120.27 (CH), 121.2 (d, *J* = 4.4 Hz, CH), 125.37 (CH), 129.31 (C), 136.36 (C), 142.72 (C), 143.58 (CH), 147.90 (C), 148.15 (d, *J* = 14.3 Hz, CH), 149.88 (d, *J* = 23.5 Hz, C), 152.68 (CH), 155.24 (C), 160.34 (d, *J* = 244.4 Hz, C) ppm.
HRMS *m*/*z* [M+H]^+^ calcd for C_21_H_14_FN_3_O_2_S 392.0863, found 392.0859.
**6-(3,4-Dimethoxyphenyl)-3-((5-methoxypyridin-3-yl)ethynyl)isothiazolo[4,3-*b*]pyridine (7l)**
This compound was obtained from the precursor **6l** (48.5 mg, 0.14 mmol, 1.0 eq.), 3,4-dimethoxyphenylboronic acid (32 mg, 0.17 mmol, 1.2 eq.), Pd(PPh_3_)_4_ (5 mg, 0.004 mmol, 0.03 eq.) and K_2_CO_3_ (39 mg, 0.28 mmol, 2 eq.) using general Suzuki coupling procedure. The crude residue was purified by flash chromatography using a mixture of hexane and acetone (in a ratio of 4:1) as mobile phase, affording the title compound as a yellow solid in 71% yield (41.3 mg, 0.10 mmol).
^1^H NMR (300 MHz, DMSO) *δ*: 3.84 (s, 3H, OCH_3_). 3.91 (s, 6H, 2 x OCH_3_), 7.12 (d, *J* = 8.8 Hz, 1H, arom H), 7.50 (bs, 2H, arom H), 7.73 (s, 1H, arom H), 8.41 (d, *J* = 2.4 Hz, 1H, arom H), 8.48 (s, 1H, arom H), 8.54 (s, 1H, arom H), 9.31 (s, 1H, arom H) ppm.
^13^C NMR (75 MHz, DMSO) *δ*: 56.11 (OCH_3_), 56.21 (OCH_3_), 56.34 (OCH_3_), 80.49 (C), 103.98 (C), 111.51 (CH), 112.68 (CH), 119.08 (C), 120.63 (CH), 122.39 (CH), 125.00 (CH), 128.74 (C), 136.02 (C), 139.67 (CH), 142.41 (C), 144.09 (CH), 147.73 (C), 149.85 (C), 150.25 (C), 153.10 (CH), 155.47 (C), 155.52 (C) ppm.
HRMS *m*/*z* [M+H]^+^ calcd for C_22_H_17_N_3_O_3_S 404.1063, found 404.1067.
**Methyl 5-((6-(3,4-dimethoxyphenyl)isothiazolo[4,3-*b*]pyridin-3-yl)ethynyl)nicotinate (7m)**
This compound was obtained from the precursor **6m** (49 mg, 0.13 mmol, 1.0 eq.), 3,4-dimethoxyphenylboronic acid (32 mg, 0.17 mmol, 1.3 eq.), Pd(PPh_3_)_4_ (5 mg, 0.004 mmol, 0.03 eq.) and K_2_CO_3_ (39 mg, 0.28 mmol, 2.2 eq.) using general Suzuki coupling procedure. The crude residue was purified by flash chromatography using a mixture of hexane and acetone (in a ratio of 4:1) as mobile phase, affording the title compound as an orange solid in 69% yield (39.8 mg, 0.09 mmol).
^1^H NMR (300 MHz, CDCl_3_) *δ*: 3.97 (s, 3H, OCH_3_), 4.00 (s, 6H, 2 x OCH_3_), 7.04 (d, *J* = 8.3 Hz, 1H, arom H), 7.20 (d, *J* = 1.8 Hz, 1H, arom H), 7.30 (dd, *J* = 8.3, 1.9 Hz, 1H, arom H), 8.23 (d, *J* = 1.9 Hz, 1H, arom H), 8.59 (t, *J* = 1.8 Hz, 1H, arom H), 9.06 (d, *J* = 1.8 Hz, 1H, arom H), 9.16 (d, *J* = 1.9 Hz, 1H, arom H), 9.22 (d, *J* = 1.8 Hz, 1H, arom H) ppm.
^13^C NMR (150 MHz, CDCl_3_) *δ*: 52.70 (OCH_3_), 56.05 (OCH_3_), 81.01 (C), 102.68 (C), 110.41 (CH), 111.78 (CH), 119.29 (C), 120.26 (CH), 125.40 (CH), 125.73 (C), 129.25 (C), 136.40 (C), 139.62 (CH), 142.69 (C), 147.82 (C), 149.71 (C), 150.03 (C), 150.51 (CH), 152.66 (CH), 155.27 (C), 155.36 (CH),164.84 (C) ppm.
HRMS *m*/*z* [M+H]^+^ calcd for C_23_H_17_N_3_O_4_S 432.1012, found 432.1021.
**6-(3,4-Dimethoxyphenyl)-3-((6-(trifluoromethyl)pyridin-3-yl)ethynyl)isothiazolo[4,3-*b*]pyridine (7n)**
This compound was obtained from the precursor **6n** (50 mg, 0.13 mmol, 1.0 eq.), 3,4-dimethoxyphenylboronic acid (32 mg, 0.17 mmol, 1.3 eq.), Pd(PPh_3_)_4_ (5 mg, 0.004 mmol, 0.03 eq.) and K_2_CO_3_ (39 mg, 0.28 mmol, 2.2 eq.) using general Suzuki coupling procedure. The crude residue was purified by flash chromatography using a mixture of hexane and acetone (in a ratio of 9:1) as mobile phase, affording the title compound as a yellow solid in 74% yield (42.5 mg, 0.10 mmol).
^1^H NMR (600 MHz, CDCl_3_) *δ*: 3.97 (s, 3H, OCH_3_), 4.00 (s, 3H, OCH_3_), 7.04 (d, *J* = 8.4 Hz, 1H, arom H), 7.20 (d, *J* = 2.1 Hz, 1H, arom H), 7.29 (dd, *J* = 8.3, 2.2 Hz, 1H, arom H), 7.76 (dd, *J* = 8.1, 0.6 Hz, 1H, arom H), 8.16 (dd, *J* = 8.0, 1.6 Hz, 1H, arom H), 8.23 (d, *J* = 2.1 Hz, 1H, arom H), 9.00 (d, *J* = 1.6 Hz, 1H, arom H), 9.17 (d, *J* = 2.1 Hz, 1H, arom H) ppm.
^19^F NMR (282 MHz, CDCl_3_) *δ*: −68.07 ppm.
^13^C NMR (151 MHz, CDCl_3_) *δ*: 56.03 (OCH_3_), 56.05 (OCH_3_), 82.19 (C), 102.13 (C), 110.38 (CH), 111.77 (CH), 120.02 (CH), 120.25 (CH), 122.23 (C), 125.39 (CH), 129.14 (C), 136.44 (C), 139.97 (CH), 142.23 (C), 147.61 (q, *J* = 35.3 Hz, C). 147.85 (C), 149.71 (C), 150.06 (C), 152.16 (CH), 152.80 (CH), 155.27 (C) ppm.
HRMS *m*/*z* [M+H]^+^ calcd for C_22_H_14_F_3_N_3_O_2_S 442.0831, found 442.0828.
**6-(3,4-Dimethoxyphenyl)-3-((4-methoxypyridin-3-yl)ethynyl)isothiazolo[4,3-b]pyridine (7o)**
This compound was obtained from the precursor **6o** (62 mg, 0.15 mmol, 1.0 eq.), 3,4-dimethoxyphenylboronic acid (39 mg, 0.22 mmol, 1.2 eq.), Pd(PPh_3_)_4_ (5.5 mg, 0.005 mmol, 0.03 eq.) and K_2_CO_3_ (50 mg, 0.36 mmol, 2 eq.) using general Suzuki coupling procedure. The crude residue was purified by flash chromatography using a mixture of hexane and ethyl acetate (in a ratio of 4:1) as mobile phase, affording the title compound as an orange solid in 71% yield (51 mg, 0.13 mmol).
^1^H NMR (400 MHz, Chloroform-*d*) δ 9.14 (d, *J* = 2.1 Hz, 1H), 8.76 (s, 1H), 8.52 (d, *J* = 5.8 Hz, 1H), 8.20 (d, *J* = 2.1 Hz, 1H), 7.29 (dd, *J* = 8.3, 2.2 Hz, 1H), 7.20 (d, *J* = 2.1 Hz, 1H), 7.04 (d, *J* = 8.4 Hz, 1H), 6.89 (d, *J* = 5.8 Hz, 1H), 4.02 (s, 3H), 3.99 (s, 3H), 3.97 (s, 3H) ppm.
^13^C NMR (101 MHz, CDCl_3_) δ 165.72, 155.35, 154.06, 152.45, 151.98, 150.11, 149.84, 147.84, 143.96, 136.37, 129.62, 125.48, 120.39, 111.94, 110.61, 106.36, 101.14, 83.25, 56.2, 56.1 ppm. HRMS *m*/*z* [M+H]^+^ calcd for C_22_H_17_N_3_O_3_S 404.1063, found 404.1058.
**6-(3,4-Dimethoxyphenyl)-3-((4-methylpyridin-3-yl)ethynyl)isothiazolo[4,3-b]pyridine (7p)**
This compound was obtained from the precursor **6p** (46 mg, 0.14 mmol, 1.0 eq.), 3,4-dimethoxyphenylboronic acid (32 mg, 0.17 mmol, 1.2 eq.), Pd(PPh_3_)_4_ (5 mg, 0.004 mmol, 0.03 eq.) and K_2_CO_3_ (39 mg, 0.28 mmol, 2 eq.) using general Suzuki coupling procedure. The crude residue was purified by flash chromatography using a mixture of hexane and ethyl acetate (in a ratio of 85:15) as mobile phase, affording the title compound as a brown solid in 95% yield (67 mg, 0.17 mmol).
^1^H NMR (400 MHz, Chloroform-*d*) δ 9.15 (d, *J* = 2.1 Hz, 1H), 8.83 (s, 1H), 8.49 (d, *J* = 5.1 Hz, 1H), 8.21 (d, *J* = 2.1 Hz, 1H), 7.29 (dd, *J* = 8.3, 2.2 Hz, 1H), 7.24 (d, *J* = 5.0 Hz, 1H), 7.20 (d, *J* = 2.1 Hz, 1H), 7.04 (d, *J* = 8.3 Hz, 1H), 3.99 (s, 3H), 3.97 (s, 3H), 2.61 (s, 3H) ppm.
^13^C NMR (101 MHz, CDCl_3_) δ 155.42, 152.56, 152.50, 150.12, 149.83, 149.70, 149.38, 147.90, 143.63, 136.40, 129.52, 125.43, 124.54, 120.37, 119.72, 111.93, 110.56, 103.23, 83.51, 56.20, 56.21, 20.57 ppm.
HRMS *m*/*z* [M+H]^+^ calcd for C_22_H_17_N_3_O_2_S 388.1114, found 388.1114.
**5-((6-(3,4-Dimethoxyphenyl)isothiazolo[4,3-b]pyridin-3-yl)ethynyl)picolinonitrile** (**7q**)
This compound was obtained from the precursor **6q** (48 mg, 0.14 mmol, 1.0 eq.), 3,4-dimethoxyphenylboronic acid (32 mg, 0.17 mmol, 1.2 eq.), Pd(PPh_3_)_4_ (5 mg, 0.004 mmol, 0.03 eq.) and K_2_CO_3_ (39 mg, 0.28 mmol, 2 eq.) using general Suzuki coupling procedure. The crude residue was purified by flash chromatography using a mixture of hexane and ethyl acetate (in a ratio of 7:3) as mobile phase, affording the title compound as an orange solid in 70% yield (40 mg, 0.10 mmol).
^1^H NMR (400 MHz, Chloroform-*d*) δ 9.17 (d, *J* = 2.1 Hz, 1H), 8.98 (dd, *J* = 2.1, 0.9 Hz, 1H), 8.24 (d, *J* = 2.0 Hz, 1H), 8.10 (dd, *J* = 8.0, 2.1 Hz, 1H), 7.76 (dd, *J* = 8.1, 0.9 Hz, 1H), 7.29 (dd, *J* = 8.3, 2.2 Hz, 1H), 7.20 (d, *J* = 2.1 Hz, 1H), 7.04 (d, *J* = 8.3 Hz, 1H), 4.00 (s, 3H), 3.97 (s, 3H) ppm.
^13^C NMR (101 MHz, CDCl_3_) δ 155.46, 153.29, 153.13, 150.27, 149.90, 148.10, 141.98, 139.47, 136.70, 133.12, 129.23, 127.95, 125.57, 122.91, 120.44, 116.90, 119.97, 110.57, 101.93, 84.10, 56.24, 56.22 ppm.
HRMS *m*/*z* [M+H]^+^ calcd for C_22_H_14_N_4_O_2_S 399.0910, found 399.0912.
**6-(3,4-Dimethoxyphenyl)-3-((5-fluoropyridin-3-yl)ethynyl)isothiazolo[4,3-b]pyridine (7r)**
This compound was obtained from the precursor **6r** (50 mg, 0.15 mmol, 1.0 eq.), 3,4-dimethoxyphenylboronic acid (39 mg, 0.22 mmol, 1.2 eq.), Pd(PPh_3_)_4_ (5.5 mg, 0.005 mmol, 0.03 eq.) and K_2_CO_3_ (50 mg, 0.36 mmol, 2 eq.) using general Suzuki coupling procedure. The crude residue was purified by flash chromatography using a mixture of hexane and ethyl acetate (in a ratio of 4:1) as mobile phase, affording the title compound as a yellow solid in 75% yield (44 mg, 0.11 mmol).
^1^H NMR (400 MHz, Chloroform-*d*) δ 9.16 (d, *J* = 2.1 Hz, 1H), 8.74 (s, 1H), 8.52 (d, *J* = 2.8 Hz, 1H), 8.22 (d, *J* = 2.0 Hz, 1H), 7.70 (ddd, *J* = 8.7, 2.8, 1.7 Hz, 1H), 7.29 (dd, *J* = 8.3, 2.2 Hz, 1H), 7.20 (d, *J* = 2.2 Hz, 1H), 7.04 (d, *J* = 8.3 Hz, 1H), 4.00 (s, 3H), 3.97 (s, 3H) ppm.
^13^C NMR (101 MHz, CDCl_3_) δ 154.14 (d, J = 259 Hz), 152.84, 150.20, 149.88, 148.34 (d, *J* = 4.1 Hz), 147.99, 142.72, 138.89, 138.77(d, J = 23 Hz), 136.56, 129.39, 125.54, 125.34 (d, J= 20 Hz), 120.41, 111.95, 110.58, 102.34 (d, *J* = 2.6 Hz), 81.11, 56.23, 56.21.
HRMS *m*/*z* [M+H]^+^ calcd for C_21_H_14_FN_3_O_2_S 392.0863, found 392.0863.
**6-(3,4-Dimethoxyphenyl)-3-((2-ethoxypyridin-3-yl)ethynyl)isothiazolo[4,3-*b*]pyridine (7s)**
This compound was obtained from the precursor **6s** (90 mg, 0.25 mmol, 1.0 eq.), 3,4-dimethoxyphenylboronic acid (55 mg, 0.3 mmol, 1.2 eq.), Pd(PPh_3_)_4_ (6 mg, 0.005 mmol, 0.02 eq.) and K_2_CO_3_ (69 mg, 0.5 mmol, 2 eq.) using general Suzuki coupling procedure. The crude residue was purified by flash chromatography using a mixture of hexane and ethyl acetate (in a ratio of 9:1) as mobile phase, affording the title compound as a yellow solid in 67% yield (70 mg, 0.17 mmol).
^1^H NMR (400 MHz, Chloroform-*d*) δ 9.13 (d, *J* = 2.0 Hz, 1H), 8.20 (s, 1H), 7.90 (dd, *J* = 7.4, 1.9 Hz, 1H), 7.29 (dd, *J* = 8.3, 2.1 Hz, 1H), 7.20 (d, *J* = 2.1 Hz, 1H), 7.03 (d, *J* = 8.3 Hz, 1H), 6.92 (dd, *J* = 7.4, 5.0 Hz, 1H), 4.52 (q, *J* = 7.0 Hz, 2H), 3.99 (s, 3H), 3.97 (s, 3H), 1.49 (t, *J* = 7.0 Hz, 3H) ppm.
^13^C NMR (101 MHz, CDCl_3_) δ 163.47, 155.34, 152.27, 150.08, 149.83, 148.02, 147.78, 144.37, 142.00, 136.32, 129.66, 125.49, 120.38, 116.33, 111.93, 110.61, 106.47, 103.22, 81.96, 62.79, 56.23, 56.21, 14.72 ppm.
HRMS *m*/*z* [M+H]^+^ calcd for C_23_H_19_N_3_O_3_S 418.1220, found 418.1208.
**6-(3,4-Dimethoxyphenyl)-3-((5-fluoro-2-methoxypyridin-3-yl)ethynyl)isothiazolo[4,3-b]pyridine** (**7t**)
This compound was obtained from the precursor **6s** (98 mg, 0.27 mmol, 1.0 eq.), 3,4-dimethoxyphenylboronic acid (60 mg, 0.32 mmol, 1.2 eq.), Pd(PPh_3_)_4_ (6 mg, 0.005 mmol, 0.02 eq.) and K_2_CO_3_ (75 mg, 0.54 mmol, 2 eq.) using general Suzuki coupling procedure. The crude residue was purified by flash chromatography using a mixture of hexane and ethyl acetate (in a ratio of 85:15) as mobile phase, affording the title compound as a yellow solid in 78% yield (90 mg, 0.21 mmol).
^1^H NMR (400 MHz, CDCl_3_) 9.14 (d, *J* = 2.1 Hz, 1H), 8.20 (d, *J* = 2.1 Hz, 1H), 8.06 (d, *J* = 3.0 Hz, 1H), 7.67 (dd, *J* = 7.8, 3.0 Hz, 1H), 7.29 (dd, *J* = 8.3, 2.2 Hz, 1H), 7.19 (d, *J* = 2.2 Hz, 1H), 7.03 (d, *J* = 8.4 Hz, 1H), 4.05 (s, 3H), 3.99 (s, 3H), 3.97 (s, 3H) ppm.
^13^C NMR (101 MHz, CDCl_3_) 159.88, 155.6, 155.22, 154.36 (d, *J* = 246 Hz), 152.45, 150.02, 149.73, 147.84, 143.31, 136.32, 134.66 (d, *J* = 26 Hz), 129.40, 129.05 (d, *J* = 23 Hz), 125.39, 120.28, 111.81, 110.47, 106.75 (d, *J* = 6 Hz), 101.13, 82.62, 56.10, 56.08, 54.67 ppm.
HRMS *m*/*z* [M+H]^+^ calcd for C_22_H_16_FN_3_O_3_S 422.0969, found 422.0958.
**6-(3,4-Dimethoxyphenyl)-3-((2,6-dimethoxypyridin-3-yl)ethynyl)isothiazolo[4,3-b]pyridine (7u)**
This compound was obtained from the precursor **6u** (100 mg, 0.27 mmol, 1.0 eq.), 3,4-dimethoxyphenylboronic acid (60 mg, 0.32 mmol, 1.2 eq.), Pd(PPh_3_)_4_ (6 mg, 0.005 mmol, 0.02 eq.) and K_2_CO_3_ (75 mg, 0.54 mmol, 2 eq.) using general Suzuki coupling procedure. The crude residue was purified by flash chromatography using a mixture of hexane and ethyl acetate (in a ratio of 9:1) as mobile phase, affording the title compound as a yellow solid in 61% yield (70 mg, 0.16 mmol).
^1^H NMR (400 MHz, CDCl_3_) 9.11 (d, *J* = 2.1 Hz, 1H), 8.18 (d, *J* = 2.1 Hz, 1H), 7.80 (d, *J* = 8.2 Hz, 1H), 7.29 (dd, *J* = 8.3, 2.2 Hz, 1H), 7.19 (d, *J* = 2.1 Hz, 1H), 7.03 (d, *J* = 8.4 Hz, 1H), 6.38 (d, *J* = 8.2 Hz, 1H), 4.07 (s, 3H), 3.99 (s, 3H), 3.98 (s, 3H), 3.96 (s, 3H) ppm.
^13^C NMR (101 MHz, CDCl_3_) 163.75, 163.35, 155.16, 151.80, 149.91, 149.69, 147.46, 144.94, 144.33, 136.09, 129.65, 125.35, 120.23, 111.79, 110.50, 104.02, 101.92, 96.76, 80.55, 56.08, 54.15, 53.86 ppm.
HRMS *m*/*z* [M+H]^+^ calcd for C_23_H_19_N_3_O_4_S 434.1169, found 434.1162.
**6-(3,4-Dimethoxyphenyl)-3-((2-fluoro-5-methylpyridin-3-yl)ethynyl)isothiazolo[4,3-b]pyridine (7v**)
This compound was obtained from the precursor **6v** (101 mg, 0.29 mmol, 1.0 eq.), 3,4-dimethoxyphenylboronic acid (63 mg, 0.35 mmol, 1.2 eq.), Pd(PPh_3_)_4_ (6.7 mg, 0.0058 mmol, 0.02 eq.) and K_2_CO_3_ (80 mg, 0.58 mmol, 2 eq.) using general Suzuki coupling procedure. The crude residue was purified by flash chromatography using a mixture of hexane and ethyl acetate (in a ratio of 9:1) as mobile phase, affording the title compound as an orange solid in 92% yield (107 mg, 0.26 mmol).
^1^H NMR (300 MHz, CDCl_3_). 9.15 (d, *J* = 2.0 Hz, 1H), 8.21 (d, *J* = 2.1 Hz, 1H), 8.06 (s, 1H), 7.90 (d, *J* = 8.7 1H), 7.28 (d, *J* = 8.3 Hz, 1H), 7.19 (d, *J* = 2.2 Hz, 1H), 7.04 (d, *J* = 8.3 Hz, 1H), 3.99 (s, 3H), 3.97 (s, 3H), 2.37 (s, 3H) ppm.
^13^C NMR (101 MHz, CDCl_3_) 160.81 (d, *J* = 240 Hz), 155.22, 152.59, 150.02, 149.72, 147.94 (d, *J* = 14 Hz), 147.85, 143.98, 142.87, 136.33, 130.88 (d, *J* = 5 Hz), 129.33, 125.36, 120.27, 111.81, 110.44, 105.62 (d, *J* = 32 Hz), 99.06 (d, *J* = 6 Hz), 82.74, 56.07, 17.30 ppm
HRMS *m*/*z* [M+H]^+^ calcd for C_22_H_16_FN_3_O_2_S 406.1020, found 406.1014.
**6-(3,4-Dimethoxyphenyl)-3-(quinolin-3-ylethynyl)isothiazolo[4,3-*b*]pyridine (7w)**
This compound was obtained from the precursor **6w** (51 mg, 0.14 mmol, 1.0 eq.), 3,4-dimethoxyphenylboronic acid (32 mg, 0.17 mmol, 1.2 eq.), Pd(PPh_3_)_4_ (5 mg, 0.004 mmol, 0.03 eq.) and K_2_CO_3_ (39 mg, 0.28 mmol, 2 eq.) using general Suzuki coupling procedure. The crude residue was purified by flash chromatography using a mixture of hexane and acetone (in a ratio of 7:3) as mobile phase and a second time using dichloromethane and methanol (in a ratio of 10:0.2), affording the title compound as a yellow solid in 75% yield (43.3 mg, 0.10 mmol).
^1^H NMR (600 MHz, CDCl_3_) *δ*: 3.97 (s, 3H, OCH_3_), 4.00 (s, 3H, OCH_3_), 7.04 (d, *J* = 8.3 Hz, 1H, arom H), 7.21 (d, *J* = 2.1 Hz, 1H, arom H), 7.30 (dd, *J* = 8.3, 2.1 Hz, 1H, arom H), 7.60–7.65 (m, 1H, arom H), 7.77–7.83 (m, 1H, arom H), 7.86 (d, *J* = 7.9 Hz, 1H, arom H), 8.15 (d, *J* = 8.5 Hz, 1H, arom H), 8.23 (d, *J* = 2.1 Hz, 1H, arom H), 8.51 (d, *J* = 1.8 Hz, 1H, arom H), 9.14 (d, *J* = 1.9 Hz, 1H, arom H), 9.17 (d, *J* = 2.0 Hz, 1H, arom H) ppm. ^13^C NMR (150 MHz, CDCl_3_) *δ*: 56.1 (OCH_3_), 56.06 (OCH_3_), 80.05 (C), 104.87 (C), 110.44 (CH), 111.79 (CH), 116.14 (C), 120.25 (CH), 125.41 (CH), 127.01 (C), 127.61 (CH), 127.92 (CH), 129.36 (C), 129.54 (CH), 130.89 (CH), 136.33 (C), 139.24 (CH), 143.42 (C), 147.36 (C), 147.75 (C), 149.74 (C), 149.71 (C), 151.51 (CH), 152.44 (CH), 155.28 (C) ppm.
HRMS *m*/*z* [M+H]^+^ calcd for C_25_H_17_N_3_O_2_S 424.1114, found 424.1113.
**6-(3,4-Dimethoxyphenyl)-3-(pyridin-4-ylethynyl)isothiazolo[4,3-*b*]pyridine (7x)**
This compound was obtained from the precursor **6w** (47 mg, 0.15 mmol, 1.0 eq.), 3,4-dimethoxyphenylboronic acid (39 mg, 0.22 mmol, 1.2 eq.), Pd(PPh_3_)_4_ (5.5 mg, 0.005 mmol, 0.03 eq.) and K_2_CO_3_ (50 mg, 0.36 mmol, 2 eq.) using general Suzuki coupling procedure. The crude residue was purified by flash chromatography using a mixture of hexane and acetone (in a ratio of 3:2) as mobile phase, affording the title compound as a light yellow solid in 71% yield (41.8 mg, 0.11 mmol).
^1^H NMR (600 MHz, CDCl_3_) *δ*: 3.97 (s, 3H, OCH_3_), 4.00 (s, 3H, OCH_3_), 7.04 (d, *J* = 8.3, 1H, arom H), 7.20 (d, *J* = 2.1 Hz, 1H, arom H), 7.29 (dd, *J* = 8.3, 2.2 Hz, 1H, arom H), 7.53–7.55 (m, 2H, arom H), 8.23 (d, *J* = 2.1 Hz, 1H, arom H), 8.70 (d, *J* = 5.8 Hz, 2H, arom H), 9.16 (d, *J* = 2.1 Hz, 1H, arom H) ppm.
^13^C NMR (150 MHz, CDCl_3_) *δ*: 56.08 (OCH_3_), 80.92 (C triple bond), 104.17 (C triple bond), 110.44 (CH), 111.81 (CH), 120.27 (CH), 125.38 (CH), 125.40 (CH), 129.26 (C), 129.97 (C), 136.42 (C), 142.67 (C), 147.84 (C), 149.74 (C), 149.98 (CH), 150.06 (C), 152.73 (CH), 155.31 (C) ppm. HRMS *m*/*z* [M+H]^+^ calcd for C_21_H_15_N_3_O_2_S 374.0958, found 374.0948.
**6-(3,4-Dimethoxyphenyl)-3-(pyridin-2-ylethynyl)isothiazolo[4,3-*b*]pyridine (7y)**
This compound was obtained from the precursor **6y** (50 mg, 0.16 mmol, 1.0 eq.), 3,4-dimethoxyphenylboronic acid (35 mg, 0.19 mmol, 1.2 eq.), Pd(PPh_3_)_4_ (5 mg, 0.004 mmol, 0.025 eq.) and K_2_CO_3_ (44 mg, 0.32 mmol, 2 eq.) using general Suzuki coupling procedure. The crude residue was purified by flash chromatography using a mixture of hexane and acetone (in a ratio of 7:3) as mobile phase, affording the title compound as a light yellow solid in 59% yield (34.6 mg, 0.09 mmol).
^1^H NMR (600 MHz, CDCl_3_) *δ*: 3.97 (s, 3H, OCH_3_), 3.99 (s, 3H, OCH_3_), 7.04 (d, *J* = 8.4 Hz, 1H, arom H), 7.20 (d, *J* = 2.1 Hz, 1H, arom H), 7.29 (dd, *J* = 8.3, 2.2 Hz, 1H, arom H), 7.32–7.35 (m, 1H, arom H), 7.72–7.78 (m, 2H, arom H), 8.22 (d, *J* = 2.1 Hz, 1H, arom H), 8.70–8.72 (m, 1H, arom H), 9.15 (d, *J* = 2.1 Hz, 1H, arom H) ppm.
^13^C NMR (150 MHz, CDCl_3_) *δ*: 56.06 (OCH_3_), 56.07 (OCH_3_), 76.45 (C triple bond), 106.11 (C triple bond), 110.45 (CH), 111.78 (CH), 120.25 (CH), 123.79 (CH), 125.38 (CH), 127.69 (CH), 129.42 (C), 136.30 (C), 136.30 (CH), 142.37 (C), 143.10 (C), 148.04 (C), 149.70 (C), 149.97 (C), 150.39 (CH), 152.46 (CH), 155.19 (C) ppm.
HRMS *m*/*z* [M+H]^+^ calcd for C_21_H_15_N_3_O_2_S 374.0958, found 374.0953.
***tert*-Butyl (3-(6-(3,4-Dimethoxyphenyl)isothiazolo[4,3-*b*]pyridin-3-yl)prop-2-yn-1-yl)carbamate (9)**
This compound was obtained from the precursor **8** (59 mg, 0.16 mmol, 1.0 eq.), 3,4-dimethoxyphenylboronic acid (35 mg, 0.19 mmol, 1.2 eq.), Pd(PPh_3_)_4_ (5 mg, 0.004 mmol, 0.025 eq.) and K_2_CO_3_ (44 mg, 0.32 mmol, 2 eq.) using general Suzuki coupling procedure. The crude residue was purified by flash chromatography using a mixture of hexane and ethyl acetate (in a ratio of 3:2) as mobile phase, affording the title compound as a light yellow solid in 87% yield (57.7 mg, 0.13 mmol).
^1^H NMR (500 MHz, CDCl_3_) *δ*: 1.48 (s, 9H, 3× CH_3_), 3.96 (s, 3H, OCH_3_), 3.98 (s, 3H, OCH_3_), 4.42 (d, *J* = 4.2 Hz, 2H, CH_2_), 4.98 (bs, 1H, NH), 7.02 (d, *J* = 8.4 Hz, 1H, arom H), 7.17 (d, *J* = 2.1 Hz, 1H, arom H), 7.27 (dd, *J* = 8.3, 2.2 Hz, 1H, arom H), 8.18 (d, *J* = 2.1 Hz, 1H, arom H), 9.09 (d, *J* = 2.1 Hz, 1H, arom H) ppm.
^13^C NMR (126 MHz, CDCl_3_) *δ*: 28.3 (CH_3_ ^t^BuO), 31.8 (CH_2_), 56.0 (OCH_3_), 71.0 (C triple bond), 80.4 (C), 104.8 (C triple bond), 110.5 (CH), 111.8 (CH), 120.2 (CH), 125.4 (CH), 129.4 (C), 136.2 (C), 143.7 (C), 147.8 (C), 149.7 (C), 150.0 (C), 152.3 (CH), 155.1 (C) ppm.
HRMS *m*/*z* [M+H]^+^ calcd for C_22_H_23_N_3_O_4_S 426.1482, found 426.1489.
**3-(6-(3,4-Dimethoxyphenyl)isothiazolo[4,3-*b*]pyridin-3-yl)prop-2-yn-1-aminium chloride (10)**
To a solution of the Boc-amino precursor **9** (100 mg, 0.235 mmol, 1.0 eq.) in 1,4-dioxane (5 mL) at 0 °C, hydrogen chloride solution 4.0 M in 1,4-dioxane (10 mL) was added carefully. The resulting mixture was stirred at room temperature for 4–5 h. After completion of the reaction as monitored by TLC, the volatiles were evaporated to dryness and the resulting residue was co-evaporated with dichloromethane and precipitate with a mixture of dichloromethane and diethyl ether (in a ratio 1:4), yielding the title compound as hydrochloric salt (orange solid) in 98% yield (83.3 mg, 0.23 mmol).
^1^H NMR (600 MHz, CDCl_3_) *δ*: 3.90 (s, 2H, CH_2_), 3.96 (s, 3H, OCH_3_), 3.98 (s, 3H, OCH_3_), 7.03 (d, *J* = 8.3 Hz, 1H, arom H), 7.18 (d, *J* = 2.1 Hz, 1H, arom H), 7.27 (dd, *J* = 8.3, 2.2 Hz, 1H, arom H), 8.18 (d, *J* = 2.1 Hz, 1H, arom H), 9.09 (d, *J* = 2.1 Hz, 1H, arom H) ppm.
HRMS *m*/*z* [M+H]^+^ calcd for C_17_H_15_N_3_O_2_S 326.0958, found 326.0962.
**Derivatisation of the propargylamino moiety at position 3 of the isothiazolo[4,3-*b*]pyridine scaffold (11a-c)**
General procedure
To a solution of the amino precursor **10** (1 eq.) in dry dichloromethane (15 mL), dry triethylamine (2 eq.) was added. The reaction mixture was cooled at 0 °C and the appropriated acid chloride, sulfonyl chloride or isocyanate (1.2 eq.) was added. The resulting mixture was stirred at room temperature overnight. After completion of the reaction as monitored by TLC, the volatiles were evaporated to dryness and the resulting residue was purified by silica gel and precipitated with diethyl ether, yielding the corresponding 3-modified isothiazolo[4,3-*b*]pyridine. Compounds **11a-c** were made according to this procedure.
***N*-(3-(6-(3,4-Dimethoxyphenyl)isothiazolo[4,3-*b*]pyridin-3-yl)prop-2-yn-1-yl)benzamide (11a)**
This compound was obtained from amino precursor **10** (50 mg, 0.14 mmol, 1.0 eq.), benzoyl chloride (24 mg, 0.17 mmol, 1.2 eq.) and triethylamine (28 mg, 0.28 mmol, 2 eq.) using above mentioned procedure. The crude residue was purified by flash chromatography using a mixture of hexane and ethyl acetate (in a ratio of 2:3) as mobile phase and a second purification using a mixture of dichloromethane and acetone (in a ratio of 9:1), affording the title compound as a yellow solid in 68% yield (40.3 mg, 0.09 mmol).
^1^H NMR (500 MHz, CDCl_3_) *δ*: 3.96 (s, 3H, OCH_3_), 3.98 (s, 3H, OCH_3_), 4.75 (d, *J* = 5.2 Hz, 2H, CH_2_), 6.80 (bs, 1H, NH), 7.02 (d, *J* = 8.4 Hz, 1H, arom H), 7.17 (d, *J* = 2.1 Hz, 1H, arom H), 7.23–7.29 (m, 1H, arom H), 7.44–7.50 (m, 2H, arom H), 7.50–7.56 (m, 1H, arom H), 7.83–7.89 (m, 2H, arom H), 8.19 (d, *J* = 2.1 Hz, 1H, arom H), 9.08 (d, *J* = 2.1 Hz, 1H, arom H) ppm.
^13^C NMR (126 MHz, CDCl_3_) *δ*: 31.16 (CH_2_), 56.03 (OCH_3_), 56.06 (OCH_3_), 71.49 (C), 104.07 (C), 110.43 (CH), 111.77 (CH), 120.22 (CH), 125.50 (CH), 127.10 (CH), 128.63 (CH), 129.27 (C), 131.87 (CH); 136.25 (C), 133.59 (C), 143.38 (C), 147.86 (C), 149.70 (C), 150.00 (C), 152.25 (CH), 155.08 (C), 167.09 (C) ppm.
HRMS *m*/*z* [M+H]^+^ calcd for C_24_H_19_N_3_O_3_S 430.1220, found 430.1218.
***N*-(3-(6-(3,4-Dimethoxyphenyl)isothiazolo[4,3-*b*]pyridin-3-yl)prop-2-yn-1-yl)benzenesulfonamide (11b)**
This compound was obtained from amino precursor **10** (50 mg, 0.14 mmol, 1.0eq.), benzenesulfonyl chloride (30 mg, 0.17 mmol, 1.2 eq.) and triethylamine (28 mg, 0.28 mmol, 2 eq.) using above mentioned procedure. The crude residue was purified by flash chromatography using a mixture of dichloromethane and acetone (in a ratio of 98:2) as mobile phase, affording the title compound as a yellow solid in 61% yield (39.2 mg, 0.08 mmol).
^1^H NMR (600 MHz, CDCl_3_) *δ*: 3.96 (s, 3H, OCH_3_), 3.98 (s, 3H, OCH_3_), 4.35 (d, *J* = 6.1 Hz, 2H, CH_2_), 5.30 (t, *J* = 6.0 Hz, 1H, NH), 7.01 (d, *J* = 8.3 Hz, 1H, arom H), 7.15 (d, *J* = 2.0 Hz, 1H, arom H), 7.24 (dd, *J* = 8.3, 2.1 Hz, 1H, arom H), 7.47–7.58 (m, 3H, arom H), 7.95–7.99 (m, 2H, arom H), 8.16 (d, *J* = 2.0 Hz, 1H, arom H), 9.05 (d, *J* = 2.0 Hz, 1H, arom H) ppm.
^13^C NMR (150 MHz, CDCl_3_) *δ*: 34.33 (CH_2_), 56.04 (OCH_3_), 56.07 (OCH_3_), 72.54 (C triple bond), 102.13 (C triple bond), 110.38 (CH), 111.75 (CH), 120.21 (CH), 125.38 (CH), 127.43 (CH), 129.16 (CH), 133.08 (CH), 136.31 (C), 139.70 (C), 142.55 (C), 147.71 (C), 149.70 (C), 150.04 (C), 152.40 (CH), 155.03 (C) ppm.
HRMS *m*/*z* [M+H]^+^ calcd for C_23_H_19_N_3_O_4_S_2_ 466.0890, found 466.0888.
**1-(3-(6-(3,4-Dimethoxyphenyl)isothiazolo[4,3-*b*]pyridin-3-yl)prop-2-yn-1-yl)-3-phenylurea (11c)**
This compound was obtained from amino precursor **10** (50 mg, 0.14 mmol, 1.0 eq.), phenyl isocyanate (20 mg, 0.17 mmol, 1.2 eq.) and triethylamine (28 mg, 0.28 mmol, 2 eq.) using above mentioned procedure. The crude residue was purified by flash chromatography using a mixture of dichloromethane and acetone (in a ratio of 95:5) as mobile phase, affording the title compound as a light yellow solid in 54% yield (33.1 mg, 0.07 mmol).
^1^H NMR (600 MHz, DMSO) *δ*: 3.83 (s, 3H, OCH_3_), 3.89 (s, 3H, OCH_3_), 4.41 (d, *J* = 5.8 Hz, 2H, CH_2_), 6.74 (t, *J* = 5.8 Hz, 1H, NH), 6.89–6.94 (m, 1H, arom H), 7.12 (d, *J* = 9.0 Hz, 1H, arom H), 7.22–7.26 (m, 2H, arom H), 7.43 (dd, *J* = 8.6, 1.0 Hz, 2H, arom H), 7.47 (dd, *J* = 6.9, 2.0 Hz, 2H, arom H), 8.50 (d, *J* = 2.1 Hz, 1H, arom H), 8.74 (s, 1H, NH), 9.24 (d, *J* = 2.1 Hz, 1H, arom H) ppm.
^13^C NMR (150 MHz, DMSO) *δ*: 30.31 (CH_2_), 55.72 (OCH_3_), 55.82 (OCH_3_), 69.73 (C triple bond), 107.47 (C triple bond), 111.13 (CH), 112.30 (CH), 117.96 (CH), 120.20 (CH), 121.51 (CH), 124.61 (CH), 128.43 (C), 128.77 (CH), 135.48 (C), 140.22 (C), 143.19 (C), 147.30 (C), 149.45 (C), 149.82 (C), 152.31 (CH), 154.89 (C), 155.14 (C) ppm.
HRMS *m*/*z* [M+H]^+^ calcd for C_24_H_20_N_4_O_3_S 445.1329, found 445.1322.
**2-Methoxy-4-(3-(pyridin-3-ylethynyl)isothiazolo[4,3-*b*]pyridin-6-yl)benzoic acid (4)**
To a solution of the precursor **3j** (50 mg, 0.13 mmol, 1.0 eq.) in a mixture of THF/H_2_O (in a ratio of 4:2) (6 mL), LiOH (6.2 mg, 0.26 mmol, 2 eq.) was added and the reaction mixture was stirred at 50 °C overnight. After disappearance of the starting material as monitored by TLC, the mixture was cooled down to room temperature and a solution of HCl (1 M) was added slowly until pH acid was reached (pH = 2–4). The volatiles were evaporated to dryness and the crude residue was precipitated with methanol and subsequently, with diethyl ether, affording the title compound as a light yellow solid in 94% yield (45.4 mg, 0.12 mmol).
^1^H NMR (600 MHz, DMSO) *δ*: 3.98 (s, 3H, OCH_3_), 7.55 (dd, *J* = 7.9, 1.3 Hz, 1H), 7.60–7.63 (m, 2H, arom H), 7.80 (d, *J* = 7.9 Hz, 1H, arom H), 8.19–8.23 (m, 1H, arom H), 8.70–8.74 (m, 2H, arom H), 8.94 (d, *J* = 1.4 Hz, 1H, arom H), 9.34 (d, *J* = 2.0 Hz, 1H, arom H) ppm.
^13^C NMR (150 MHz, DMSO) *δ*: 56.18 (OCH_3_), 80.47 (C), 103.69 (C), 111.91 (CH), 118.64 (C), 119.37 (CH), 121.54 (C), 124.24 (CH), 126.74 (CH), 131.53 (CH), 134.96 (C), 139.61 (CH), 140.60 (C), 142.43 (C), 148.01 (C), 149.70 (CH), 151.15 (CH), 152.46 (CH), 154.74 (C), 158.75 (C), 167.05 (C) ppm.
HRMS *m*/*z* [M+H]^+^ calcd for C_21_H_13_N_3_O_3_S 388.0750, found 388.0746.
**4-(2-(4-(3-(Pyridin-3-ylethynyl)isothiazolo[4,3-*b*]pyridin-6-yl)phenoxy)ethyl)morpholine (5a)**
To a solution of the precursor **3k** (99 mg, 0.30 mmol, 1.0 eq.) in acetone, K_2_CO_3_ (62 mg, 0.45 mmol, 1.5 eq.) was added and the mixture was stirred at 50 °C for 20 min. Separately, to a second solution of 4-(2-chloroethyl)morpholine hydrochloride (67 mg, 0.36 mmol, 1.2 eq.) in acetone, K_2_CO_3_ (62 mg, 0.45 mmol, 1.5 eq.) and KI (100 mg, 0.6 mmol, 2 eq.) was added and the mixture was stirred at 50 °C for 20 min. Then, the second solution was added to the one contained the precursor **3k** and the final mixture was stirred at reflux overnight. After disappearance of the starting material as monitored by TLC, the mixture was cooled down to room temperature and the volatiles were evaporated in *vacuo*. The crude residue was purified by silica gel flash chromatography using a mixture of hexane and acetone (in a ratio of 7:3) as mobile phase (adding 6 drops of ammonia 25% in 100 mL of solvent), affording the title compound as a light yellow solid in 48% yield (64.5 mg, 0.14 mmol).
^1^H NMR (600 MHz, CDCl_3_) *δ*: 2.58–2.64 (m, 4H, 2 x CH_2_), 2.86 (t, *J* = 5.7 Hz, 2H, CH_2_), 3.74–3.79 (m, 4H, 2 x CH_2_), 4.20 (t, *J* = 5.7 Hz, 2H, CH_2_), 7.06–7.10 (m, 2H, arom H), 7.37 (ddd, *J* = 7.8, 4.9, 0.8 Hz, 1H, arom H), 7.62–7.67 (m, 2H, arom H), 7.98 (dt, *J* = 7.9, 1.9 Hz, 1H, arom H), 8.20 (d, *J* = 2.1 Hz, 1H, arom H), 8.64 (dd, *J* = 4.9, 1.6 Hz, 1H, arom H), 8.92 (dd, *J* = 2.0, 0.7 Hz, 1H, arom H), 9.14 (d, *J* = 2.1 Hz, 1H, arom H) ppm.
^13^C NMR (150 MHz, CDCl_3_) *δ*: 54.10 (CH_2_), 57.54 (CH_2_), 66.01 (CH_2_), 66.91 (CH_2_), 80.07 (C), 104.00 (C) 115.49 (CH), 119.25 (C), 123.12 (CH), 125.27 (CH), 128.76 (CH), 129.16 (C), 136.07 (C), 138.68 (CH), 143.23 (C), 147.63 (C), 149.78 (CH), 152.32 (CH), 152.43 (CH), 155.30 (C), 159.53 (C) ppm.
HRMS *m*/*z* [M+H]^+^ calcd for C_25_H_22_N_4_O_2_S 443.1536, found 443.1531.
**3-(Pyridin-3-ylethynyl)-6-(4-(2-(pyrrolidin-1-yl)ethoxy)phenyl)isothiazolo[4,3-*b*]pyridine (5b)**
To a solution of the precursor **3k** (250 mg, 0.76 mmol, 1.0 eq.) in DMF (10 mL), K_2_CO_3_ (157 mg, 1.14 mmol, 1.5 eq.) was added and the mixture was stirred at 50 °C for 20 min. Separately, to a second solution of 1-(2-chloroethyl)pyrrolidine hydrochloride (170 mg, 0.91 mmol, 1.2 eq.) in DMF (10 mL), K_2_CO_3_ (157 mg, 1.14 mmol, 1.5 eq.) and KI (252 mg, 1.52 mmol, 2 eq.) was added and the mixture was stirred at 50 °C for 20 min. After this time, the second solution was added to the one contained the precursor **3k** and the final mixture was stirred at 100 °C for 5 h. After disappearance of the starting material as monitored by TLC, the mixture was cooled down to room temperature and the volatiles were evaporated in *vacuo*. The crude residue was purified by silica gel flash chromatography using a mixture of hexane and acetone (in a ratio of 7:3) as mobile phase (adding 6 drops of ammonia 25% in 100 mL of solvent), affording the title compound as a light yellow solid in 25% yield (80.9 mg, 0.19 mmol).
^1^H NMR (600 MHz, CDCl_3_) *δ*: 1.82–1.87 (m, 4H, 2 x CH_2_), 2.64–2.69 (m, 4H, 2 × CH_2_), 2.96 (t, *J* = 5.9 Hz, 2H, CH_2_), 4.20 (t, *J* = 5.9 Hz, 2H, CH_2_), 7.09 (d, *J* = 8.7 Hz, 2H, arom H), 7.37 (dd, *J* = 7.6, 5.0 Hz, 1H, arom H), 7.65 (d, *J* = 8.7 Hz, 2H, arom H), 7.98 (dt, *J* = 7.9, 1.8 Hz, 1H, arom H), 8.20 (d, *J* = 2.0 Hz, 1H, arom H), 8.64 (dd, *J* = 4.8, 1.5 Hz, 1H, arom H), 8.92 (d, *J* = 1.4 Hz, 1H, arom H), 9.14 (d, *J* = 2.0 Hz, 1H, arom H) ppm.
^13^C NMR (150 MHz, CDCl_3_) *δ*: 23.48 (CH_2_), 54.74 (CH_2_), 54.98 (CH_2_), 67.28 (CH_2_), 80.09 (C), 103.96 (C), 115.49 (CH), 119.26 (C), 123.11 (CH), 125.20 (CH), 128.71 (CH), 128.96 (C), 136.13 (C), 138.68 (CH), 143.17 (C), 147.60 (C), 149.76 (CH), 152.31 (CH), 152.49 (CH), 155.33 (C), 159.71 (C) ppm.
HRMS *m*/*z* [M+H]^+^ calcd for C_25_H_22_N_4_OS 427.1587, found 427.1582.
***N*-(Propargyl)aniline**
To a suspension of aniline (94 mg, 1.0 mmol, 1.2 eq.) and K_2_CO_3_ (174 mg, 1.26mmol, 1.5 eq.) in DMF at 0 °C, propargyl bromide 80% w/w in toluene (125 mg, 0.84 mmol, 1.0 eq.) was added. The reaction mixture was stirred at room temperature overnight. The volatiles were evaporated in *vacuo* and the crude residue was purified by silica gel flash chromatography using a mixture of hexane and ethyl acetate (in a ratio of 9:1) as mobile phase, affording the title compound as colourless oil in 87% yield (95.9 mg, 0.73 mmol).
^1^H NMR (300 MHz, CDCl_3_) *δ*: 2.21 (t, *J* = 2.4 Hz, 1H, CH), 3.92 (d, *J* = 2.4 Hz, 2H, CH_2_), 6.65–6.72 (m, 2H, arom H), 6.79 (t, *J* = 7.3 Hz, 1H, arom H), 7.15–7.29 (m, 2H, arom H) ppm.
HRMS *m*/*z* [M+H]^+^ calcd for C_9_H_9_N 132.0808, found 132.0802.
***N*-(3-(6-Bromoisothiazolo[4,3-*b*]pyridin-3-yl)prop-2-yn-1-yl)aniline (12)**
A solution of 3,6-dibromoisothiazolo[4,3-*b*]pyridine **1b** (100 mg, 0.34 mmol, 1 eq.) in dry THF was degassed with a flow of argon for 5 min. Subsequently, Pd(PPh_3_)_2_Cl_2_ (4.8 mg, 0.0068 mmol, 0.02 eq.), CuI (0.65 mg, 0.0034 mmol, 0.01 eq.) and *N*-(propargyl)aniline (45 mg, 0.34 mmol, 1.0 eq.) were added and the reaction mixture was filled with argon and stirred at room temperature for 5 h. After disappearance of the starting material as monitored by TLC, the volatiles were evaporated in *vacuo* and the crude residue was purified by silica gel flash chromatography using a mixture of hexane and ethyl acetate (in a ratio of 8:2) as mobile phase, affording the title compound as dark oil in 78% yield (91.3 mg, 0.26 mmol).
^1^H NMR (300 MHz, CDCl_3_) *δ*: 4.11 (bs, 1H, NH), 4.40 (s, 2H, CH_2_), 6.72–6.87 (m, 3H, arom H), 7.19–7.31 (m, 2H, arom H), 8.31 (d, *J* = 2.1 Hz, 1H, arom H), 8.79 (d, *J* = 2.1 Hz, 1H, arom H) ppm.
HRMS *m*/*z* [M+H]^+^ calcd for C_15_H_10_BrN_3_S 343.9852, found 343.9842.
***N*-(3-(6-(3,4-Dimethoxyphenyl)isothiazolo[4,3-*b*]pyridin-3-yl)prop-2-yn-1-yl)aniline (13)**
This compound was obtained from the precursor **12** (52 mg, 0.15 mmol, 1.0 eq.), 3,4-dimethoxyphenylboronic acid (33 mg, 0.18 mmol, 1.2 eq.), Pd(PPh_3_)_4_ (5 mg, 0.004 mmol, 0.03 eq.) and K_2_CO_3_ (50 mg, 0.36 mmol, 2 eq.) using general Suzuki coupling procedure.
The crude residue was purified by flash chromatography using a mixture of hexane and acetone (in a ratio of 8:2) as mobile phase., affording the title compound as a yellow solid in 81% yield (47.2 mg, 0.12 mmol).
^1^H NMR (600 MHz, CDCl_3_) *δ*: 3.96 (s, 3H, OCH_3_), 3.98 (s, 3H, OCH_3_), 4.14 (bs, 1H, NH), 4.42 (s, 2H, CH_2_), 6.76–6.80 (m, 2H, arom H), 6.82 (tt, *J* = 7.4, 1.0 Hz, 1H, arom H), 7.02 (d, *J* = 8.3 Hz, 1H, arom H), 7.16 (d, *J* = 2.1 Hz, 1H, arom H), 7.23–7.28 (m, 3H, arom H), 8.16 (d, *J* = 2.1 Hz, 1H, arom H), 9.09 (d, *J* = 2.1 Hz, 1H, arom H) ppm.
^13^C NMR (150 MHz, CDCl_3_) *δ*: 35.18 (CH_2_), 56.03 (OCH_3_), 56.05 (OCH_3_), 71.05 (C triple bond), 105.98 (C triple bond), 110.42 (CH), 111.74 (CH), 113.59 (CH), 118.80 (CH), 120.20 (CH), 125.38 (CH), 129.32 (CH), 129.32 (C), 136.15 (C), 143.85 (C), 146.66 (C), 147.76 (C), 149.66 (C), 149.93 (C), 152.16 (CH), 155.07 (C) ppm.
HRMS *m*/*z* [M+H]^+^ calcd for C_23_H_19_N_3_O_2_S 402.1271, found 402.1263.
**6-Bromo-N-(pyridin-3-ylmethyl)isothiazolo[4,3-*b*]pyridin-3-amine (14)**
The mixture of 3,6-dibromoisothiazolo[4,3-*b*]pyridine (147 mg, 0.5 mmol, 1.0 eq.), pyridin-3-ylmethanamine (216 mg, 2.0 mmol, 4.0 eq.) and K_2_CO_3_ (276 mg, 2.0 mmol, 4 eq.) in acetonitrile (5 mL) was heated under reflux till the starting material was almost disappeared on TLC. After cooling down to room temoerature, the mixture was concentrated under reduced pressure. The residue was purified with silica gel column (acetone in dichloromethane from 0 to 20% in 15 min.) to yield the title compounds 109 mg (0.34 mmol, 68%) as yellowish solid.
^1^H NMR (300 MHz, CDCl_3_) δ: 8.70 (s, 1H), 8.62 (d, *J* = 4.6 Hz, 1H), 8.31 (s, 1H), 7.98 (s, 1H), 7.75 (d, *J* = 7.7 Hz, 1H), 7.32 (dd, *J* = 7.7 & 5.0 Hz, 1H), 6.69 (br., 1H), 4.59 (d, *J* = 5.5 Hz, 2H), ppm.
^13^C NMR (75 MHz, CDCl_3_) δ: 154.2, 150.0, 149.5, 145.8, 135.6, 133.1, 131.1, 130.1, 123.8, 121.4, 49.3 ppm.
**6-(3,4-Dimethoxyphenyl)-N-(pyridin-3-ylmethyl)isothiazolo[4,3-b]pyridin-3-amine (15)**
This compound was obtained from the precursor **14** (55 mg, 0.17 mmol, 1.0 eq.), 3,4-dimethoxyphenylboronic acid (36 mg, 0.20 mmol, 1.2 eq.), Pd(PPh_3_)_4_ (5 mg, 0.004 mmol, 0.027 eq.) and K_2_CO_3_ (45 mg, 0.32 mmol, 2 eq.) using general Suzuki coupling procedure. The crude residue was purified by silica gel flash chromatography (from 0 to 20% acetone in dichloromethane over 15 min) to yield the title compounds 50 mg (0.12 mmol, 70%) as yellowish solid.
^1^H NMR (300 MHz, CDCl_3_) δ: 8.72 (s, 1H), 8.59 (m, 2H), 7.88 (s, 1H), 7.76 (d, *J* = 7.7 Hz, 1H), 7.30 (m, 1H), 7.20 (d, *J* = 8.2 Hz, 1H), 7.14 (s, 1H), 6.98 (d, *J* = 8.2 Hz, 1H), 6.86 (t, *J* = 5.5 Hz, 1H), 4.61 (d, *J* = 5.5 Hz, 2H), 3.96 (s, 3H), 3.94 (s, 3H) ppm.
^13^C NMR (75 MHz, CDCl_3_) δ: 171.01, 154.50, 149.88, 149.61, 149.51, 145.21, 136.82, 135.52, 133.77, 131.49, 130.35, 124.67, 123.75, 119.99, 111.68, 110.48, 56.04, 49.35 ppm.
HRMS *m*/*z* [M+H]^+^ calcd for C_20_H_18_N_4_O_2_S 379.1223, found 379.1224.
**6-Bromo-3-(pyridin-3-yl)isothiazolo[4,3-b]pyridine (16)**
The title compound was synthesized from 3,6-dibromoisothiazolo[4,3-*b*]pyridine **1b** (294mg, 1.0 mmol, 1.0 eq.) and pyridin-3-ylboronic acid (148 mg, 1.2 mmol, 1.2 eq.) Pd(PPh_3_)_4_ (14 mg, 0.02 mmol, 0.02 eq.) and K_2_CO_3_ (414 mg, 3.0 mmol, 3 eq.) using general Suzuki coupling procedure (80 °C, 3 h). The crude residue was purified by silica gel flash chromatography (from 0 to 20% acetone in dichloromethane over 15 min) to yield the title compounds 248 mg (0.85 mmol, 85%) as yellowish solid.
^1^H NMR (300 MHz, CDCl_3_) δ: 9.28 (s, 1H), 8.82 (s, 1H), 8.72 (d, *J* = 4.4 Hz, 1H), 8.55 (d, *J* = 7.6 Hz, 1H), 8.34 (s, 1H), 7.50 (dd, *J* = 7.6 & 5.2 Hz, 1H) ppm.
**6-(3,4-Dimethoxyphenyl)-3-(pyridin-3-yl)isothiazolo[4,3-b]pyridine (17)**
The title compound was synthesized from intemediate **16** (246 mg, 0.5 mmol, 1.0 eq.) and 3,4-dimethoxyphenylboronic acid (109 mg, 0.6 mmol, 1.2 eq.), Pd(PPh_3_)_4_ (7 mg, 0.01 mmol, 0.02 eq.) and K_2_CO_3_ (212 mg, 1.5 mmol, 3 eq.) using general Suzuki coupling procedure (80 °C, 4 h). The crude residue was purified by silica gel flash chromatography (from 0 to 20% acetone in dichloromethane over 15 min) to yield the title compounds 140 mg (0.40 mmol, 80%) as yellowish solid.
^1^H NMR (300 MHz, CDCl_3_) δ: 9.33 (s, 1H), 9.13 (s, 1H), 8.71 (d, *J* = 3.2 Hz, 1H), 8.62 (d, *J* = 7.6 Hz, 1H), 8.20 (s, 1H), 7.51 (t, *J* = 5.3 Hz, 1H), 7.30 (d, *J* =8.2 Hz, 1H), 7.22 (s, 1H), 7.04 (d, *J* = 8.2 Hz, 1H), 4.00 (s, 3H), 3.97 (s, 3H) ppm.
^13^C NMR (75 MHz, CDCl_3_) δ: 158.24, 156.72, 151.94, 150.60, 149.92, 149.67, 148.86, 143.90, 135.84, 135.34, 129.51, 126.82, 125.05, 123.97, 120.15, 111.79, 110.39, 56.06 ppm.
HRMS *m*/*z* [M+H]^+^ calcd for C_19_H_15_N_3_O_2_S 350.0958, found 350.0951.
***N*-(6-Bromoisothiazolo[4,3-b]pyridin-3-yl)nicotinamide (19)**
To a mixture of 6-bromoisothiazolo[4,3-b]pyridin-3-amine (115 mg, 0.5 mmol, 1.0 eq.) and nicotinoyl chloride hydrochloride (115 mg, 0.6 mmol, 1.2 eq.) in dichloromethane (5 mL), trimethylamine (0.5 mL) was added. The mixture was stirred at room temperature for 2 h. After concentration under reduced pressure, the residue was purified by silica gel flash chromatography (from 0 to 20% acetone in dichloromethane over 15 min) to yield the title compounds 110 mg (0.33 mmol, 66%) as yellowish solid.
^1^H NMR (300 MHz, CDCl_3_) δ: 9.18 (d, *J* = 1.8 Hz, 1H), 9.08 (d, *J* = 1.8 Hz, 1H), 8.88 (br., 1H), 8.83 (d, *J* = 4.8 Hz, 1H), 8.53 (d, *J* = 1.8 Hz, 1H), 8.24 (d, *J* = 8.0 Hz, 1H), 7.49 (dd, *J* = 8.0 & 4.8 Hz, 1H) ppm.
^13^C NMR (75 MHz, CDCl_3_) δ: 164.4, 163.7, 148.6, 147.9, 138.7, 135.3, 132.0, 128.7, 125.7, 123.9, 121.9, 114.6 ppm.
**N-(6-(3,4-Dimethoxyphenyl)isothiazolo[4,3-b]pyridin-3-yl)nicotinamide (20)**
The title compound was synthesized from intemediate **19** (100 mg, 0.3 mmol, 1.0 eq.) and 3,4-dimethoxyphenylboronic acid (66 mg, 0.36 mmol, 1.2 eq.), Pd(PPh_3_)_4_ (4.2 mg, 0.005 mmol, 0.02 eq.) and K_2_CO_3_ (124 mg, 0.9 mmol, 3 eq.) using general Suzuki coupling procedure (80 °C, 4 h). The crude residue was purified by silica gel column (acetone in dichloromethane from 0 to 20% in 15 min) to yield the title compounds 82 mg (0.21 mmol, 71%) as yellowish solid.
^1^H NMR (300 MHz, CDCl_3_+CD_3_OD) δ: 9.21 (d, *J* = 2.0 Hz, 1H), 8.79 (s, 2H), 8.43 (m, 2H), 7.61 (dd, *J* = 8.1 Hz, 5.0 Hz, 1H), 7.31 (dd, *J* = 8.3 Hz, 1.8 Hz, 1H), 7.23 (s, 1H), 7.08 (d, *J* = 8.3 Hz, 1H), 3.98 (s, 3H), 3.96 (s, 3H) ppm. ^13^C NMR (75 MHz, CDCl_3_+CD_3_OD) δ: 165.38, 152.34, 150.55, 149.70, 148.53, 145.77, 140.57, 138.00, 136.38, 130.79, 129.65, 127.94, 125.72, 123.98, 120.69, 115.36, 111.92, 110.33, 55.81, 55.77 ppm. HRMS *m*/*z* [M+H]^+^ calcd for C_20_H_16_N_4_O_3_S 393.1016, found 393.1019.
**6-(3,4-Dimethoxyphenyl)-3-(2-(pyridin-3-yl)ethyl)isothiazolo[4,3-*b*]pyridine (22)**
To a solution of compound **21** (200 mg, 0.54 mmol, 1.0 eq.) in a mixture of EtOH/THF (10 mL, in a ratio of 9:1), palladium hydroxide on carbon 20 wt. % was (100 mg) was added. The air from the flask was evacuated and filled with hydrogen using a balloon. This procedure was repeated 3 times and the reaction mixture was stirred under hydrogen at 60 °C for 3 days. After disappearance of the starting material as monitored by TLC, the mixture was cooled down to room temperature and the volatiles were evaporated in *vacuo*. The crude residue was purified by silica gel flash chromatography using a mixture of dichloromethane and acetone (in a ratio of 9:1) as mobile phase and a second time using hexane and acetone (7:3), affording the title compound as a beige solid in 20% yield (40.4 mg, 0.11 mmol).
^1^H NMR (500 MHz, CDCl_3_) *δ*: 3.27 (t, *J* = 7.6 Hz, 2H, CH_2_), 3.79 (t, *J* = 7.7 Hz, 2H, CH_2_), 3.96 (s, 3H, OCH_3_), 3.99 (s, 3H, OCH_3_), 7.03 (d, *J* = 8.3 Hz, 1H, arom H), 7.19 (d, *J* = 2.1 Hz, 1H, arom H), 7.23 (dd, *J* = 7.7, 4.8 Hz, 1H, arom H), 7.28 (dd, *J* = 8.3, 2.3 Hz, 1H, arom H), 7.52–7.57 (m, 1H, arom H), 8.13 (d, *J* = 2.0 Hz, 1H, arom H), 8.54–8.46 (m, 2H, arom H), 9.02 (d, *J* = 2.0 Hz, 1H, arom H) ppm. ^13^C NMR (126 MHz, CDCl_3_) *δ*: 27.54 (CH_2_), 33.68 (CH_2_), 56.05 (OCH_3_), 110.50 (CH), 111.78 (CH), 120.13 (CH), 123.44 (CH), 125.08 (CH), 129.92 (C), 135.46 (C), 135.83 (C), 135.99 (CH), 145.14 (C), 148.07 (CH), 149.64 (C), 149.79 (C), 149.97 (CH), 150.53 (CH), 155.74 (C), 163.66 (C) ppm. HRMS m/z [M+H]^+^ calcd for C_21_H_19_N_3_O_2_S 378.1273, found 378.1271.
**(Z)-6-(3,4-Dimethoxyphenyl)-3-(2-methoxy-2-(pyridin-3-yl)vinyl)isothiazolo[4,3-*b*]pyridine (23a)**
To the solution of 6-(3,4-dimethoxyphenyl)-3-(pyridin-3-ylethynyl)isothiazolo[4,3-b]pyridine **21** (187 mg, 0.5 mmol, 1.0 eq.) in methanol (5 mL) at room temperature, potassium *tert*-butoxide (112 mg, 1.0 mmol, 2.0 eq.) was added. The resulting mixture was stirred under reflux for 2 h. After cooling down to room temperature, the mixture was concentrated under reduced pressure. The residue was purified by silica gel flash chromatography (from 0 to 20% acetone in dichloromethane over 15 min) to yield the title compound as a yellowish solid (150 mg, 0.37 mmol, 74%).
^1^H NMR (300 MHz,) δ: 8.99 (s, 1H), 8.95 (s, 1H), 8.69 (d, *J* = 4.5 Hz, 1H), 8.16 (s, 1H), 7.96 (d, *J* = 7.8 Hz, 1H), 7.42 (dd, *J* = 7.8 & 4.5 Hz, 1H), 7.36 (s, 1H), 7.28 (d, *J* = 8.3 Hz, 1H), 7.21 (s, 1H), 7.02 (d, *J* = 8.3 Hz, 1H), 3.99 (s, 3H), 3.96 (s, 3H), 3.90 (s, 3H) ppm.
^13^C NMR (150 MHz, CDCl_3_ + CD_3_OD) δ: 157.26, 156.32, 154.34, 150.53, 149.63, 149.63, 148.42, 144.29, 135.96, 134.48, 130.12, 129.42, 124.94, 123.59, 120.11, 111.77, 110.52, 104.07, 58.09, 56.07 ppm. HRMS *m*/*z* [M+H]^+^ calcd for C_22_H_19_N_3_O_3_S 406.1220, found 406.1208.
**((Z)-2-((2-(6-(3,4-Dimethoxyphenyl)isothiazolo[4,3-*b*]pyridin-3-yl)-1-(pyridin-3-yl)vinyl)oxy)-*N*,*N*-dimethylethan-1-amine (23b)**
To a solution of 6-(3,4-dimethoxyphenyl)-3-(pyridin-3-ylethynyl)isothiazolo[4,3-*b*]pyridine (187 mg, 0.5 mmol, 1.0 eq.) in 2-(dimethylamino)ethan-1-ol (4 mL) at room temperature, potassium *tert*-butoxide (112 g, 1.0 mmol, 2.0 eq.) was added. The resulting mixture was stirred at 80 °C for 2 h. The mixture was cooled down to room temperature and concentrated under reduced pressure. The resulted residue was purified by silica gel flash chromatography (from 0 to 20% acetone in dichloromethane over 15 min) to yield the title compound as a yellowish solid (167 mg, 0.36 mmol, 72%).
^1^H NMR (300 MHz, CDCl_3_) δ: 8.99 (s, 1H), 8.97 (d, *J* = 1.8 Hz, 1H), 8.68 (d, *J* = 4.0 Hz, 1H), 8.15 (d, *J* = 1.8 Hz, 1H), 7.99 (d, *J* = 8.0 Hz, 1H), 7.42 (dd, *J* = 7.8 & 4.8 Hz, 1H), 7.31 (m, 2H), 7.20 (d, *J* = 1.8 Hz, 1H), 7.02 (d, *J* = 8.3 Hz, 1H), 4.10 (t, *J* = 6.0 Hz, 2H), 3.99 (s, 3H), 3.96 (s, 3H), 2.89 (t, *J* = 6.0 Hz, 2H), 2.32 (s, 6H) ppm.
^13^C NMR (75 MHz, CDCl_3_) δ: 156.47, 156.33, 154.25, 150.45, 149.71, 149.6, 148.48, 144.40, 135.94, 134.58, 130.13, 129.92, 124.91, 123.52, 120.10, 111.76, 110.51, 104.11, 68.99, 59.14, 56.06, 45.94 ppm.
HRMS *m*/*z* [M+H]^+^ calcd for C_25_H_26_N_4_O_3_S 463.1798, found 463.1795.
**(Z)-4-(2-(6-(3,4-Dimethoxyphenyl)isothiazolo[4,3-*b*]pyridin-3-yl)-1-(pyridin-3-yl)vinyl)morpholine (23c)**
A suspension of 6-(3,4-dimethoxyphenyl)-3-(pyridin-3-ylethynyl)isothiazolo[4,3-*b*]pyridine (187 mg, 0.5 mmol, 1.0 eq.) in morpholine (5 mL) was heated at 80 °C for 3h. The mixture was cooled down to room temperature and concentrated under reduced pressure to dryness. The resulted residue was purified by silica gel flash chromatography (from 0 to 20% acetone in dichloromethane over 15 min) to yield the title compound as a yellowish solid (140 mg, 0.30 mmol, 60%).
^1^H NMR (300 MHz, CDCl_3_) δ: 8.86 (m, 2H), 8.66 (s, 1H), 7.95 (s, 1H), 7.73 (d, *J* = 7.8 Hz, 1H), 7.53 (dd, *J* = 7.7 & 5.0 Hz, 1H), 7.23 (d, *J* = 8.3 Hz, 1H), 7.16 (s, 1H), 7.00 (d, *J* = 8.3 Hz, 1H), 6.79 (s, 1H), 3.96 (s, 3H), 3.94 (s, 3H), 3.79 (m, 4H), 3.23 (m, 4H) ppm. ^13^C NMR (75 MHz, CDCl_3_) δ: 162.4, 154.36, 151.80, 151.42, 150.35, 149.60, 148.00, 144.05, 137.50, 135.94, 131.71, 130.27, 125.10, 124.71, 119.94, 111.71, 110.42, 94.86, 66.62, 66.45, 56.04, 56.02, 48.38 ppm. HRMS *m*/*z* [M+H]^+^ calcd for C_25_H_24_N_4_O_3_S 461.1642, found 461.1637.
**(Z)-2-((2-(6-(3,4-Dimethoxyphenyl)isothiazolo[4,3-*b*]pyridin-3-yl)-1-(pyridin-3-yl)vinyl)thio)ethan-1-ol (23d)**
The mixture of 6-(3,4-dimethoxyphenyl)-3-(pyridin-3-ylethynyl)isothiazolo[4,3-*b*]pyridine 187 mg, 0.5 mmol, 1.0 eq.), 2-mercaptoethan-1-ol (156 mg, 2.0 mmol, 4.0 eq.) and K_2_CO_3_ (280 mg, 2.0 mmol, 4.0 eq.) in THF (5 mL) was heated under reflux stirred for 2 h. The mixture was cooled down to room temperature and concentrated under reduced pressure to dryness. The resulted residue was purified by silica gel flash chromatography (from 0 to 20% acetone in dichloromethane over 15 min) to yield the title compound as a yellowish solid (167 mg, 0.37 mmol, 74%).
^1^H NMR (300 MHz, CDCl_3_) δ: 8.93 (s, 1H), 8.80 (d, *J* = 4.6 Hz, 1H), 8.66 (s, 1H), 7.99 (s, 1H), 7.76 (d, *J* = 7.8 Hz, 1H), 7.64 (s, 1H), 7.49 (dd, *J* = 7.7 & 5.0 Hz, 1H), 7.20 (d, *J* = 8.3 Hz, 1H), 7.13 (s, 1H), 6.98 (d, *J* = 8.3 Hz, 1H), 3.96 (s, 3H), 3.95 (m, 2H), 3.94 (s, 3H), 3.16 (m, 3H) ppm.
^13^C NMR (75 MHz, CDCl_3_) δ: 157.65, 154.50, 151.05, 150.04, 149.83, 149.62, 149.54, 143.95, 141.95, 136.94, 136.21, 133.84, 129.62, 124.86, 120.05, 114.41, 111.73, 110.35, 60.51, 56.05, 35.41 ppm.
HRMS *m*/*z* [M+H]^+^ calcd for C_23_H_21_N_3_O_3_S_2_ 452.1097, found 452.1092.
**Kinase assays**

Compounds were screened for PIKfyve activity using a mobility shift assay (Nanosyn, Santa Clara, CA, USA). Data are expressed as compound concentration that causes 50% inhibition of the enzymatic activity (IC_50_ values). Various compounds were also tested for their binding affinity to PIP4K2C, using the DiscoverX/Kinome platform, in which the results are expressed as the dissociation constant value (*K*_D_ values).

**Antiviral screening**
*Cell lines for antiviral and viability assays*

Human astrocytes (U-87 MG, ATCC, Manassas, VA, USA) and Vero E6/TMPRSS2 (JCRB cell bank, Osaka, Japan #cat JCRB1819) cells were grown in Dulbecco’s Modified Eagle’s medium DMEM (Gibco, Waltham, MA, USA) supplemented with 10% FCS (Biowest, Bradenton, FL, USA), 1% L-glutamine (Gibco, Waltham, MA, USA), and 1% penicillin-streptomycin (Pen-strep, Gibco, Waltham, MA, USA). Vero E6 (ATCC, Manassas, VA, USA) cells were maintained in DMEM supplemented with 10% FCS, 1% L-glutamine, 1% Pen-strep, 1% nonessential amino acids (Corning, New York, USA), 1% HEPES (Gibco, Waltham, MA, USA), 1% Sodium pyruvate (Thermo Fisher Scientific, Waltham, MA, USA). All cell lines were maintained in a humidified incubator with 5% CO_2_ at 37 °C and tested negative for mycoplasma by MycoAlert (Lonza, Morristown, NJ, USA). Cells from passages 14–15 were used for this study.

*Virus constructs*

The plasmid encoding infectious VEEV (TC-83) with a nanoluciferase reporter (VEEV-TC-83-Cap-nLuc-Tav) used for virus production (VEEV-TC-83-nLuc) was a gift from Dr. William B. Klimstra (Department of Immunology, University of Pittsburgh, Pittsburgh) [32]. Plasmids used to produce SARS-CoV-2 (SARS-CoV-2 expressing nLuc-reporter gene) were a gift from Dr. Luis Martinez-Sobrido [33].

*Virus production*

The VEEV-TC-83-nLuc virus was harvested from culture supernatant 24 h post-electroporation. Viral stock for SARS-CoV-2-nLuc was generated as previously described [34]. Virus produced in Vero E6/TMPRSS2 cells and passaged 3–4 times was used for the experiments.

Supernatants were collected, clarified and stored at −80 °C. Viral titers were measured using plaque assays on Vero (VEEV) or Vero E6 cells (SARS-CoV-2).

*Measuring antiviral activity*

U-87 MG and Vero E6/TMPRSS2 cells were pretreated with the compounds or DMSO for 1 h prior to infection with VEEV-TC-83-nLuc or SARS-CoV-2-nLuc at a multiplicity of infection (MOI) of 0.01 (VEEV) and 0.5 (SARS-CoV-2) respectively (*n* = 4). The inhibitors were maintained for the duration of the experiment. Viral infection was measured at 18 h post-infection (VEEV) and 24 h post-infection using a nanoluciferase assay. The relative light units (RLUs) were normalized to DMSO-treated cells (set as 100%).

*Cell viability assays*

Cell viability was measured in virus-infected cells using the AlamarBlue^®^ reagent (Invitrogen, Waltham, MA, USA) according to the manufacturer’s protocol. Fluorescence was detected at 560 nm on GloMax Discover Microplate Reader (Promega, Madison, WI, USA). The raw fluorescence values were normalized to DMSO-treated cells (set as 100%).

**Antitumoral assays**
*Cell culture*

Cancer cell lines Capan-1, HCT-116, NCI-H460, LN-229, HL-60, K-562 and Z-138 were obtained from the American Type Culture Collection (ATCC, Manassas, VA, USA), and the DND-41 cell line was purchased from the Deutsche Sammlung von Mikroorganismen und Zellkulturen (DSMZ Leibniz-Institut, Braunschweig, Germany). All cell lines were cultured according to the suppliers’ recommendations. Culture media were acquired from Gibco Life Technologies (Waltham, MA, USA) and supplemented with 10% fetal bovine serum (HyClone, GE Healthcare Life Sciences, Boston, MA, USA).

*Cell proliferation assays*

Adherent cell lines Capan-1, HCT-116, NCI-H460 and LN-229 were seeded at densities ranging from 500 to 1500 cells per well in 384-well culture plates (Greiner, Vilvoorde, Belgium). After overnight incubation, cells were treated with various concentrations of the test compounds. Suspension cell lines DND-41, HL-60, K-562 and Z-138 were seeded at densities between 2500 to 5500 cells per well in 384-well culture plates containing the test compounds at identical concentration points. After 72 h of incubation with the compounds, cell viability was assessed using the CellTiter 96^®^ AQueous One Solution Cell Proliferation Assay (MTS) reagent according to the manufacturer’s instructions (Promega, Madison, WI, USA). Absorbance was measured at 490 nm using a SpectraMax Plus 384 (Molecular Devices, San Jose, CA, USA) and the optical density values were utilized to determine the 50% cytotoxic concentration (CC_50_). Each compound underwent testing in two independent experiments.

## 4. Conclusions

**Figure 5:**
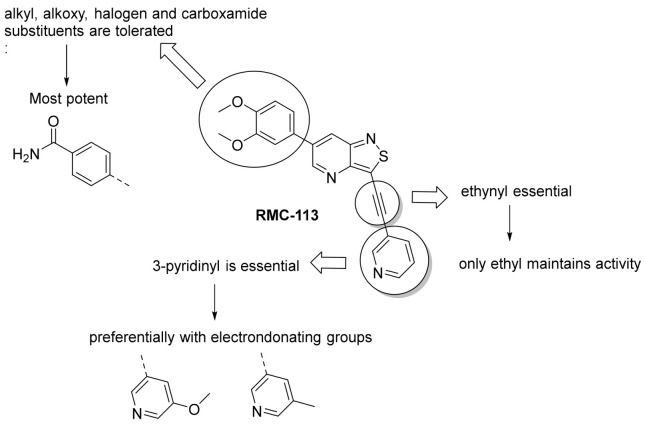
Summary of the SAR study versus PIKfyve.

A previously discovered dual inhibitor of the lipid kinases PIKfyve and PIP4K2C (RMC-113) was subjected to a systematic SAR study by introducing structural modifications at positions 3 and 6 of the central isothiazolo[4,3-*b*]pyridine scaffold (Figure 5). The SAR study was guided by a biochemical PIKfyve assay. PIKfyve inhibition tolerated a wide variety of substituents (alkyl, alkoxy, halogens and carboxamides) on the aryl ring at position 6, with the 4-carboxamide analogue emerging as the most potent PIKfyve inhibitor (IC_50_ = 1 nM). In contrast, the SAR at position 3 was more restricted. Replacement of the pyridinyl moiety by (substituted) phenyl rings reduced the enzymatic activity. The introduction of small electron-donating substituents (methyl and methoxy) on the pyridinyl yielded potent PIKfyve inhibitors, with IC_50_ values in the low nM range. The acetylenic moiety proved to be essential for PIKfyve inhibition, and only the saturated congener containing an ethyl linker displayed potent PIKfyve inhibition, albeit less active than the acetylene counterpart. In general, the SAR against PIKfyve and PIP4K2C ran in parallel, although the compounds were 2- to 5-fold less potent on PIP4K2C relative to PIKfyve. The majority of the dual PIKfyve/PIP4K2C inhibitors displayed low µM antiviral activity against both VEEV and SARS-CoV-2. An antitumoral screening against a variety of cancer cell lines revealed that some of the potent PIKfyve/PIP5K2C inhibitors lack antitumoral activity. Overall, the correlation between enzymatic potency and cellular efficacy is not very clear, which will need further investigation. The most promising antiviral and/or antitumoral compounds will need to be evaluated for their pharmacokinetic properties. Given the fact that some of the isothiazolo[4,3-*b*]pyridines show antiviral as well as antitumoral activity (e.g., compounds **3m** and **7l**), priority might be given to antitumoral evaluation.

## Figures and Tables

**Table 1:** Initial SAR of RMC-113.

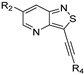			
**Cmpd#**	**R_2_**	**R_4_**	**IC_50_ (µM)**
**21** (RMC-113)	3,4-dimethoxyphenyl	3-pyridyl	0.0080
**2a**	H	3-pyridyl	1.15
**7a**	3,4-dimethoxyphenyl	*n*-propyl	0.85

**Table 2:** PIKfyve inhibition of 6-aryl-3-(pyridin-3-yl)ethynyl)isothiazolo[4,3-*b*]pyridines.

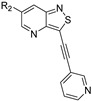		
**Cmpd#**	**R_2_**	**IC_50_ (µM)**
**21** (RMC-113)	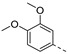	0.0080
**3a**	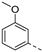	0.0068
**3b**	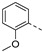	0.0068
**3c**	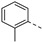	0.0082
**3d**	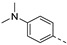	0.0022
**3e**	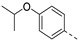	0.020
**3f**	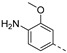	0.0028
**3g**	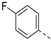	0.010
**3h**	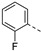	0.0088
**3i**	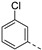	0.021
**3j**	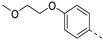	0.0021
**4**	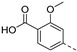	0.049
**5a**	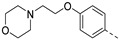	0.0071
**5b**	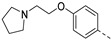	0.0055
**3m**	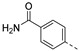	0.0016
**3n**	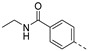	0.0035
**3o**	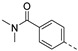	0.025
**3p**	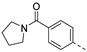	0.020
**3q**	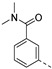	0.018
**3r**	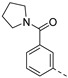	0.0061
**3s**	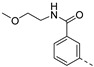	0.0019

**Table 3:** SAR of the pyridinyl moiety.

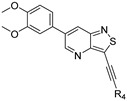		
**Cmpd#**	**R_4_**	**IC_50_ (µM)**
**7b**	Ph	0.059
**7c**	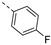	0.25
**7d**	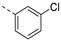	0.039
**7e**	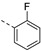	0.062
**7f**	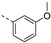	0.021
**7g**	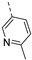	0.36
**7h**	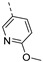	0.59
**7i**	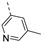	0.002
**7j**	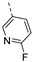	0.12
**7k**	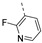	0.029
**7l**	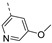	0.0033
**7m**	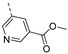	0.12
**7n**	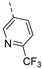	0.68
**7o**	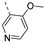	0.019
**7p**	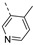	0.033
**7q**	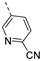	>10
**7r**	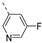	0.098
**7s**	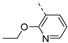	0.11
**7t**	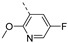	>10
**7u**	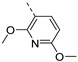	5.67
**7v**	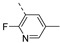	0.042
**7w**	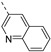	5.69
**7x**	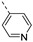	0.075
**7y**	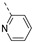	0.030

**Table 4:** SAR of the alkynyl linker.

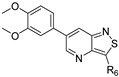		
**Cmpd#**	**R_6_**	**IC_50_ (µM)**
**17**	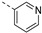	0.602
**15**	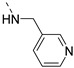	>10
**20**	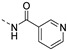	4.29
**22**	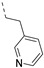	0.033
**11a**	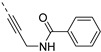	2.04
**11b**	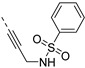	1.84
**11c**	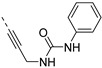	>10
**13**	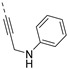	0.184
**23a**	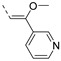	0.525
**23b**	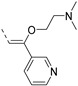	0.309
**23c**	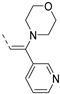	0.613
**23d**	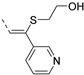	1.54

**Table 5:** PIP4K2C affinity and PIKfyve inhibition of selected isothiazolo[4,3-*b*]pyridines.

Cmpd#	PIP4K2C *K*_D_ (µM)	PIKfyve IC_50_ (µM)
**21** (RMC-113)	0.046	0.008
**3a**	0.190	0.0068
**3i**	0.180	0.021
**3f**	0.044	0.0028
**3m**	0.041	0.0016
**4**	0.068	0.049
**3s**	0.030	0.0019
**7e**	0.280	0.062
**7k**	0.066	0.029
**7l**	0.039	0.0033
**7q**	>30	>100
**7w**	2	5.69
**7o**	0.230	0.019
**7r**	0.110	0.098

**Table 6:** Antiviral activity of selected isothiazolo[4,3-*b*]pyridines.

	VEEV (TC-83)		SARS-CoV2	
Cmpd#	EC_50_ (µM) ^a^	CC_50_ (µM) ^b^	EC_50_ (µM) ^a^	CC_50_ (µM) ^b^
**21** (RMC-113)	0.75 ± 0.02	>10	1.01 ± 0.04	>20
**3a**	6.88 ± 0.33	>10	<10	<10
**3h**	6.93 ± 0.13	>20	1.34 ± 0.5	>20
**3f**	0.26 ± 0.08	>20	2.74 ± 0.04	>20
**3m**	0.19 ± 0.001	>20	1.82 ± 0.94	>20
**4**	8.7 ± 1.7	>10	0.36 ± 0.19	>20
**5a**	0.31 ± 0.01	>10	5.63 ± 0.97	>20
**5b**	0.71 ± 0.09	>10	1.42 ± 0.14	>20
**3s**	0.4 ± 0.08	>10	5.65 ± 1.49	>20
**7e**	16.84 ± 1.25	>20	0.55 ± 0.15	15.69 ± 1.34
**7f**	4.79 ± 0.24	>10	<10	<10
**7k**	3.4 ± 0.08	>10	1.83 ± 0.30	>20
**7l**	1.64 ± 0.05	>10	4.92 ± 0.93	>20
**7m**	1.16 ± 0.001	>10	2.56 ± 0.86	>20
**7w**	2.08 ± 0.1	>10	<10	<10
**7o**	0.92 ± 0.08	7.69 ± 0.001	<10	<10
**7p**	4.01 ± 0.13	>10	0.83 ± 0.06	>20
**7r**	1.85 ± 0.12	>10	1.92 ± 0.52	>20
**Apilimod**	1.35 ± 0.49	>10	0.19 ± 0.00	18.35 ± 0.42

^a^ EC_50_= Half-maximal effective concentrations. ^b^ CC_50_ = Half-maximal cytotoxic concentrations.

**Table 7:** Antiproliferative activity of selected isothiazolo[4,3-*b*]pyridines.

	CC_50_ (µM) ^a^							
	Capan-1	HCT-116	LN-229	NCI-H460	DND-41	HL-60	K-562	Z-138
**21** (RMC-113)	0.81 ± 0.11	0.82 ± 0.37	1.62 ± 0.50	2.52 ± 1.09	1.75 ± 1.02	1.47 ± 0.45	4.24 ± 0.11	0.77 ± 0.06
**3g**	0.73 ± 0.23	0.81 ± 0.16	4.39 ± 0.94	1.46 ± 0.36	3.06 ± 1.71	1.51 ± 0.71	5.18 ± 0.48	1.03 ± 0.20
**3b**	1.02 ± 0.09	1.02 ± 1.24	3.28 ± 1.24	2.97 ± 0.75	3.92 ± 0.72	1.48 ± 1.13	5.27 ± 0.59	1.81 ± 0.69
**3m**	2.41 ± 0.94	0.34 ± 0.78	3.23 ± 0.78	2.9 ± 1.72	2.25 ± 0.19	1.0 ± 0.57	6.53 ± 0.46	0.96 ± 0.30
**7i**	14.6 ± 2.97	≥74.0	>100	>100	>100	>100	>100	70.2 ± 2.55
**7l**	1.79 ± 1.20	2.45 ± 0.85	5.15 ± 2.05	5.38 ± 0.56	5.25 ± 0.64	1.80 ± 0.00	5.70 ± 1.27	1.85 ± 0.07
**7p**	>100	>100	>100	>100	>100	>100	>100	12.3 ± 1.77
**Apilimod**	<0.02	<0.02	0.08 ± 0.09	<0.02	0.08 ± 0.02	0.17 ± 0.02	0.33 ± 0.34	0.06 ± 0.00
**Vacuolin-1**	0.90 ± 0.13	1.87 ± 0.75	6.35 ± 1.80	1.46 ± 0.16	24.13 ± 1.21	26.43 ± 1.48	17.21 ± 3.08	16.6 ± 1.31
**YM-201636**	0.17 ± 0.05	1.08 ± 1.23	0.16 ± 0.03	0.81 ± 0.09	0.38 ± 0.09	0.31 ± 0.11	1.24 ± 0.28	0.17 ± 0.04

^a^ CC_50_ = concentration required for 50% inhibition of tumor growth; CC_50_ values are the average of two independent experiments ± S.D.

## Data Availability

The data are contained within the article or Supplementary Materials.

## Supplementary Materials

The following supporting information can be downloaded at: https://www.mdpi.com/article/10.3390/ph18091341/s1, Copies of ^1^H- and ^13^C-NMR spectra.
