# Supplementary material for: Structure–Activity Relationship Study of 3-Alkynyl-6-aryl-isothiazolo[4,3-b]pyridines as Dual Inhibitors of the Lipid Kinases PIKfyve and PIP4K2C
Source: Pharmaceuticals (Basel). 2025 Sep 6;18(9):1341. doi: 10.3390/ph18091341 (PMC12472322; doi:10.3390/ph18091341)

Structure–Activity Relationship Study of 3-Alkynyl-6-aryl-isothiazolo[4,3-*b*]pyridines as Dual Inhibitors of the Lipid Kinases PIKfyve and PIP4K2C

Demian Kalebic [1], Ling-Jie Gao [2], Belén Martinez-Gualda [2], Marwah Karim [3], Sirle Saul [3], Do Hoang Nhu Tran [3], Jef Rozenski [2], Leentje Persoons [4], Dominique Schols [5], Wim Dehaen [1], Shirit Einav [3,6,7] and Steven De Jonghe [5,*]

[1] Sustainable Chemistry for Metals and Molecules, Department of Chemistry, KU Leuven, Celestijnenlaan 200F, B-3001 Leuven, Belgium; demian.kalebic@gmail.com (D.K.); wim.dehaen@kuleuven.be (W.D.)

[2] Laboratory of Medicinal Chemistry, Department of Pharmaceutical and Pharmacological Sciences, Rega Institute for Medical Research, KU Leuven, Herestraat 49, Box 1030, 3000 Leuven, Belgium; ling-jie.gao@kuleuven.be (L.-J.G.); belen.mar.gual@gmail.com (B.M.-G.); jef.rozenski@kuleuven.be (J.R.)

[3] Division of Infectious Diseases and Geographic Medicine, Department of Medicine, Stanford University School of Medicine, Stanford, CA 94305, USA; mkarim@stanford.edu (M.K.); sirlesaul@gmail.com (S.S.); dotran@stanford.edu (D.H.N.T.); seinav@stanford.edu (S.E.)

[4] Molecular Genetics and Therapeutics in Virology and Oncology Research Group, Department of Microbiology, Immunology and Transplantation, Rega Institute for Medical Research, KU Leuven, Herestraat 49, Box 1043, 3000 Leuven, Belgium; leentje.persoons@kuleuven.be

[5] Molecular, Structural and Translational Virology Research Group, Department of Microbiology, Immunology and Transplantation, Rega Institute for Medical Research, KU Leuven, Herestraat 49, Box 1043, 3000 Leuven, Belgium; dominique.schols@kuleuven.be

[6] Department of Microbiology and Immunology, Stanford University School of Medicine, Stanford, CA 94305, USA

[7] Chan Zuckerberg Biohub, San Francisco, CA 94305, USA

* Correspondence: steven.dejonghe@kuleuven.be

^{1}H NMR spectrum of **2a**

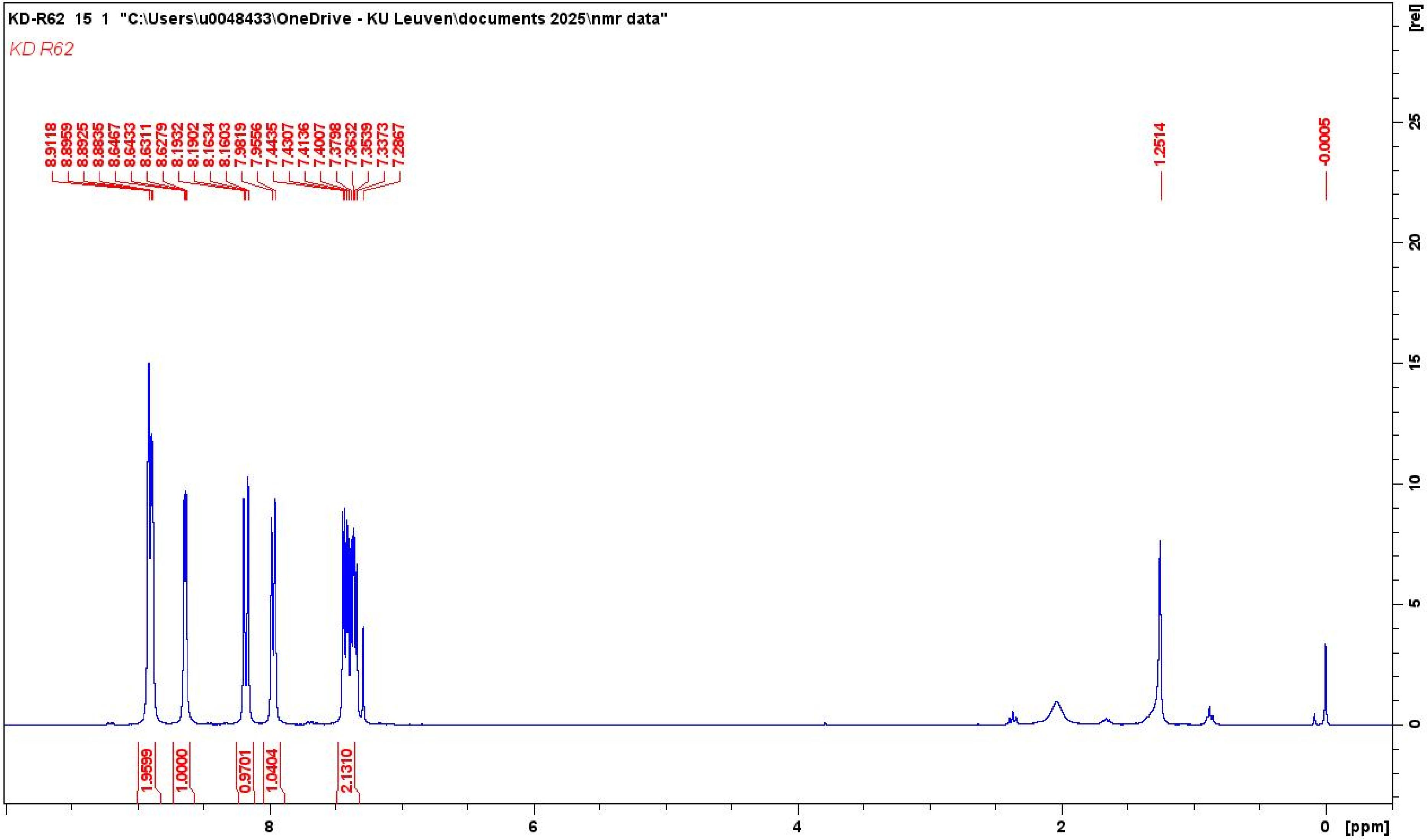

^{13}C NMR spectrum of **2a**

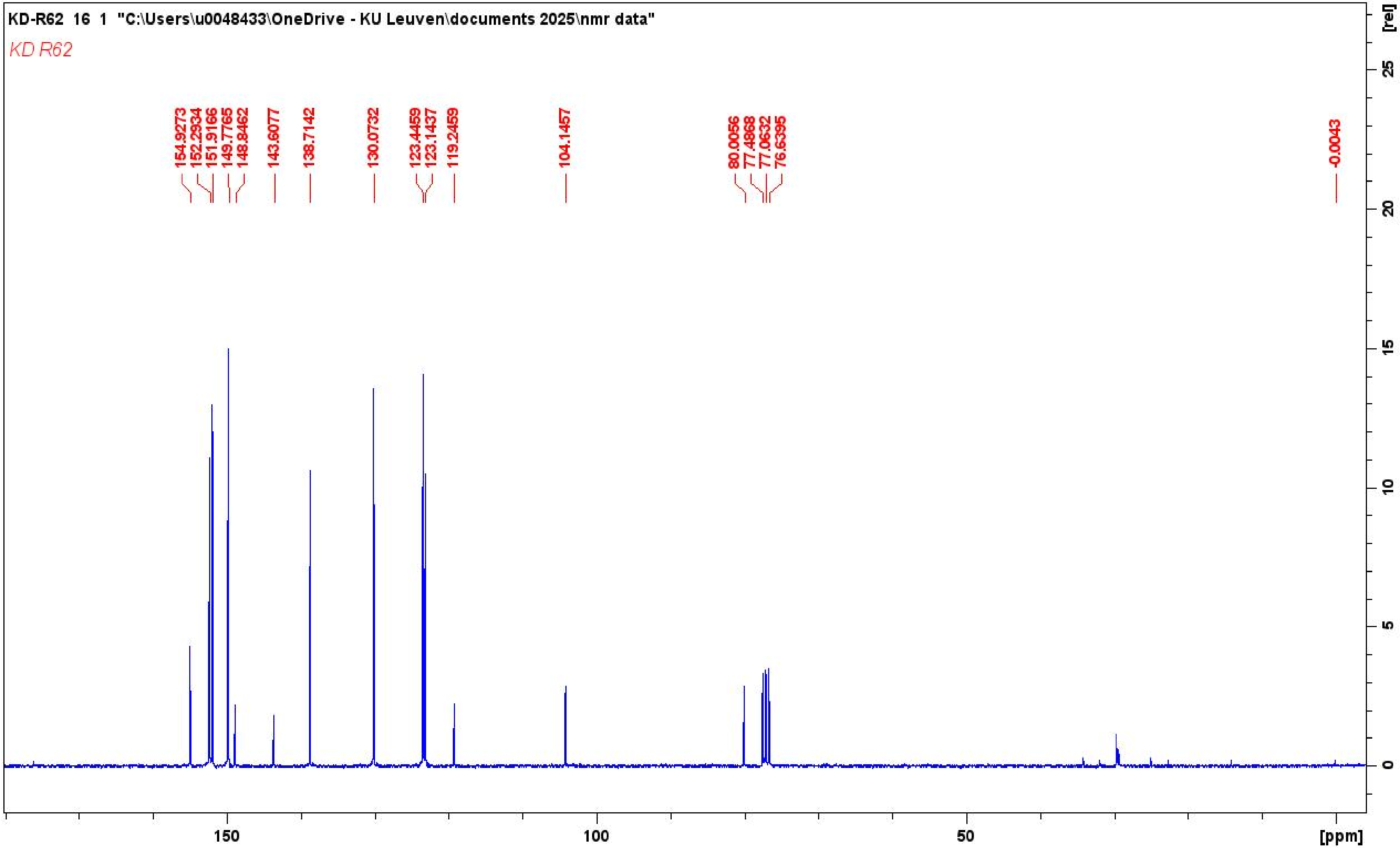

^{1}H NMR spectrum of **3a**

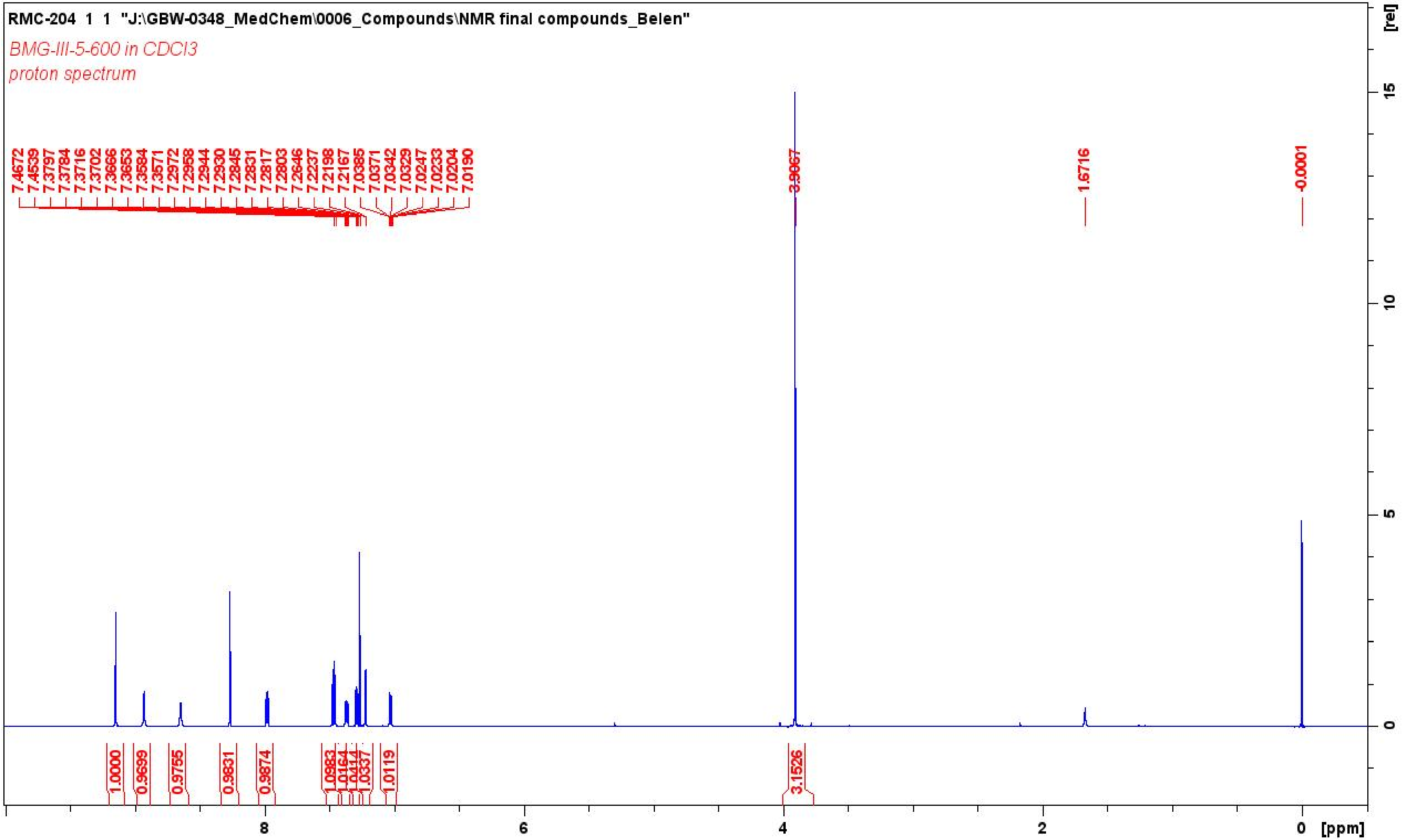

^{13}C NMR spectrum of **3a**

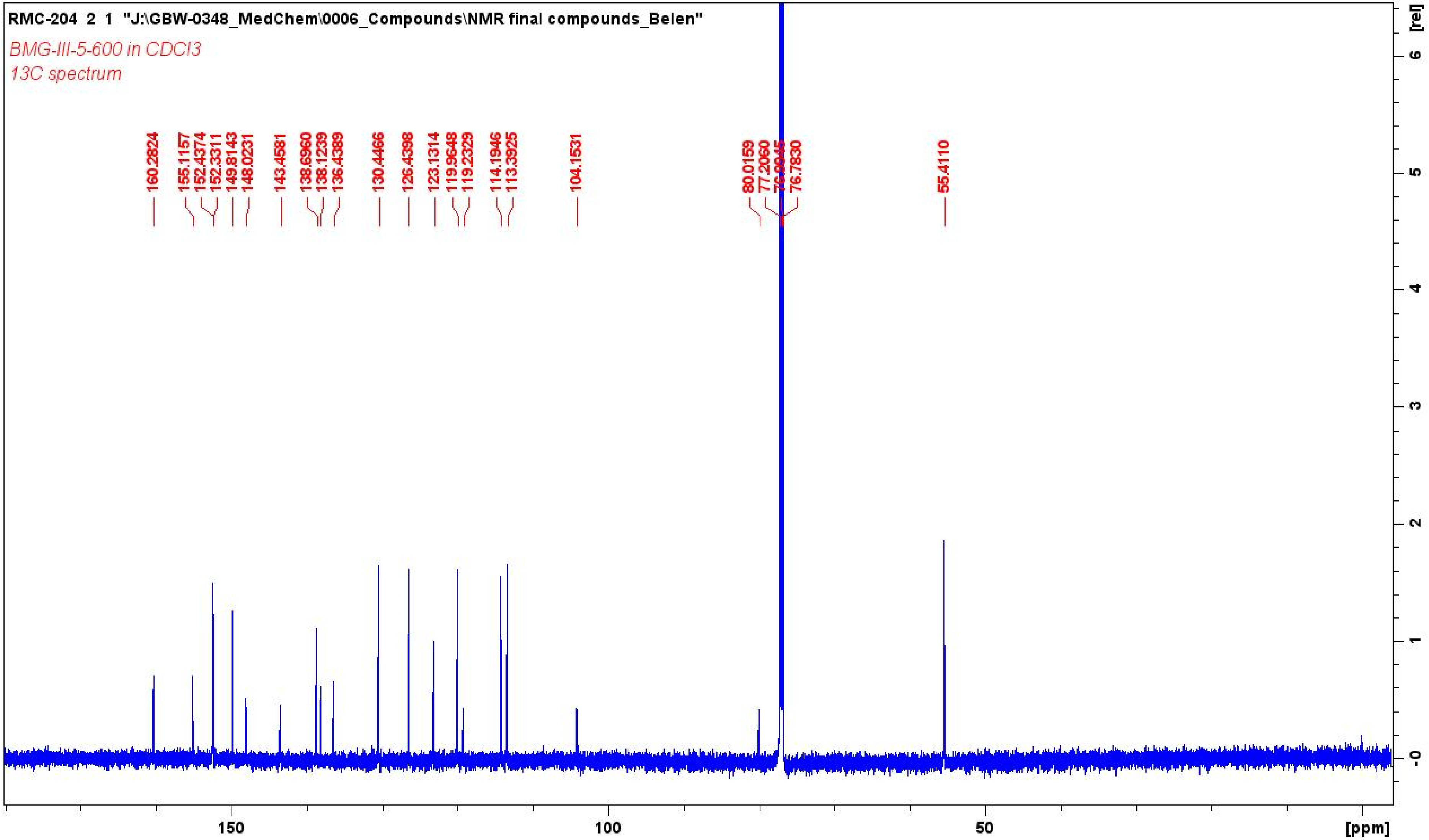

^{1}H NMR spectrum of **3b**

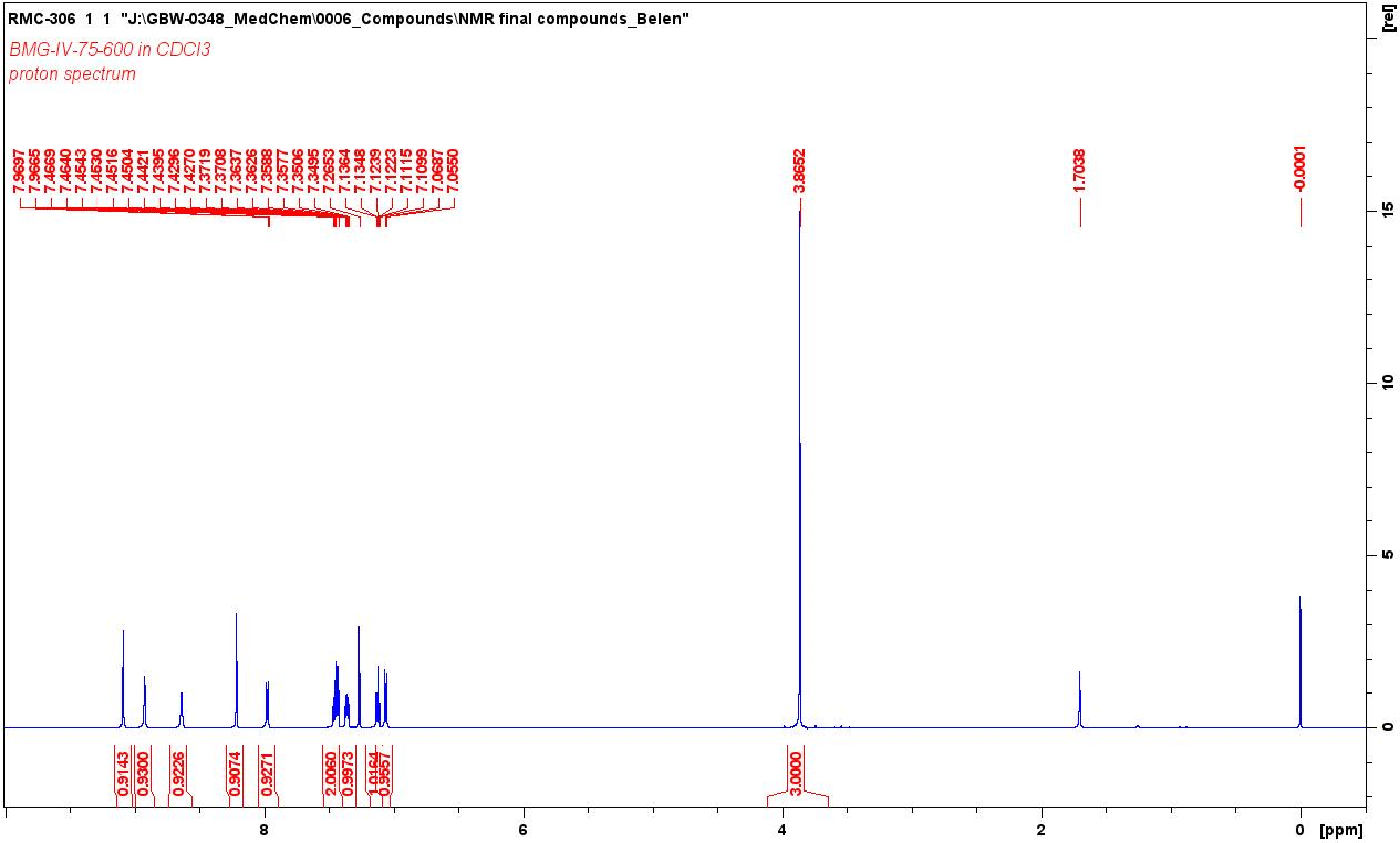

^{13}C NMR spectrum of **3b**

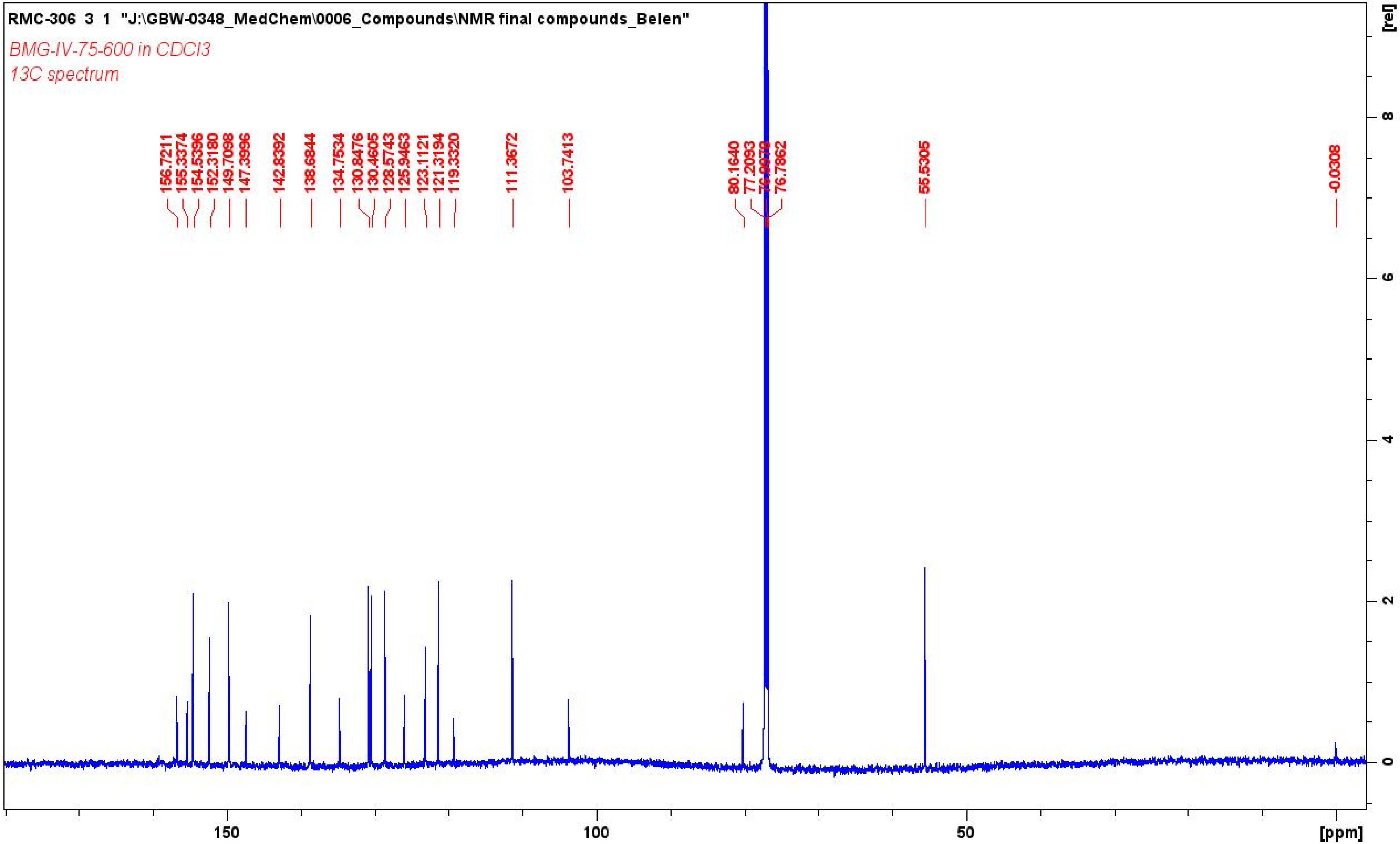

^1H NMR spectrum of **3c**

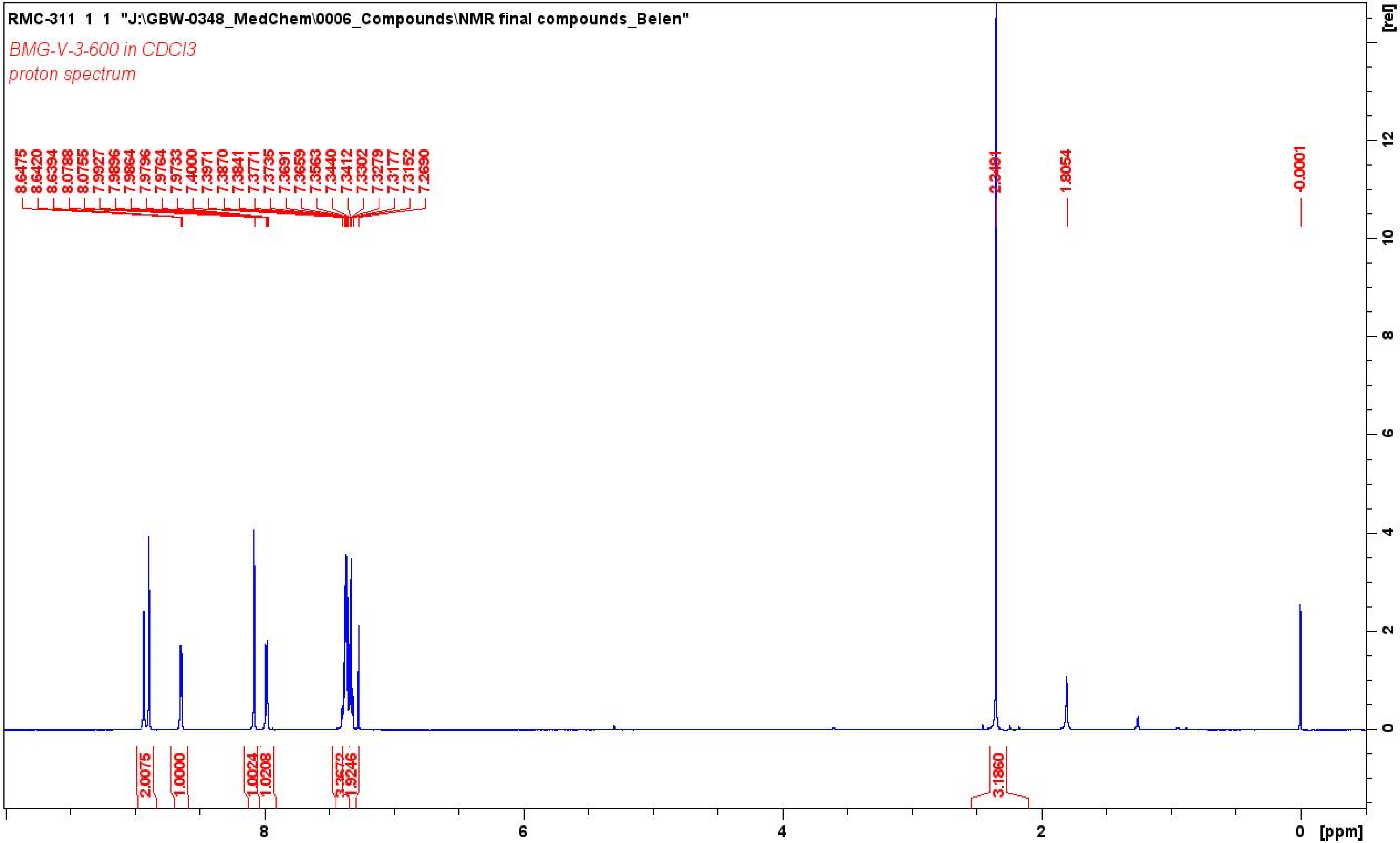

^{13}C NMR spectrum of **3c**

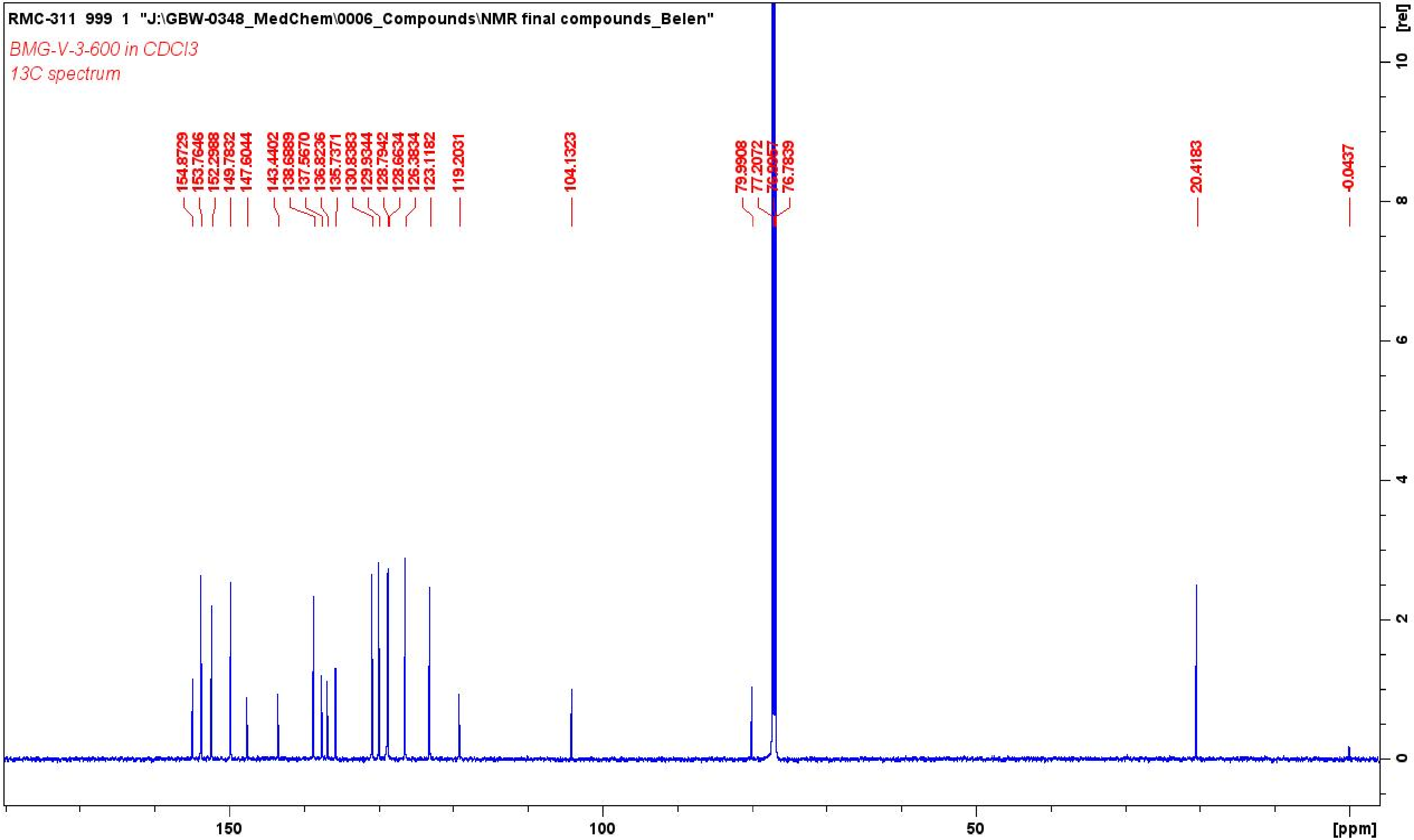

^{1}H NMR spectrum of **3d**

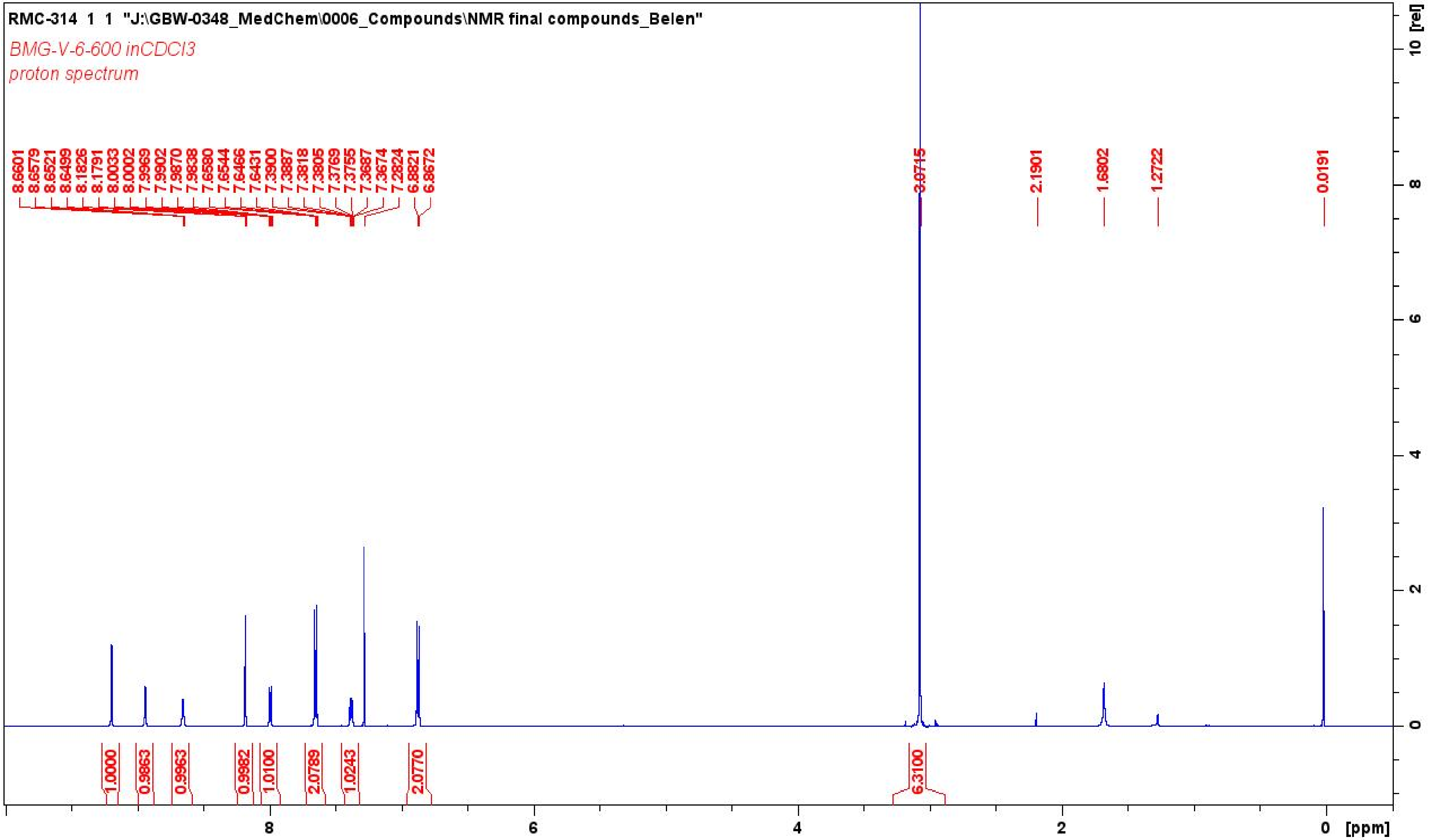

^{13}C NMR spectrum of **3d**

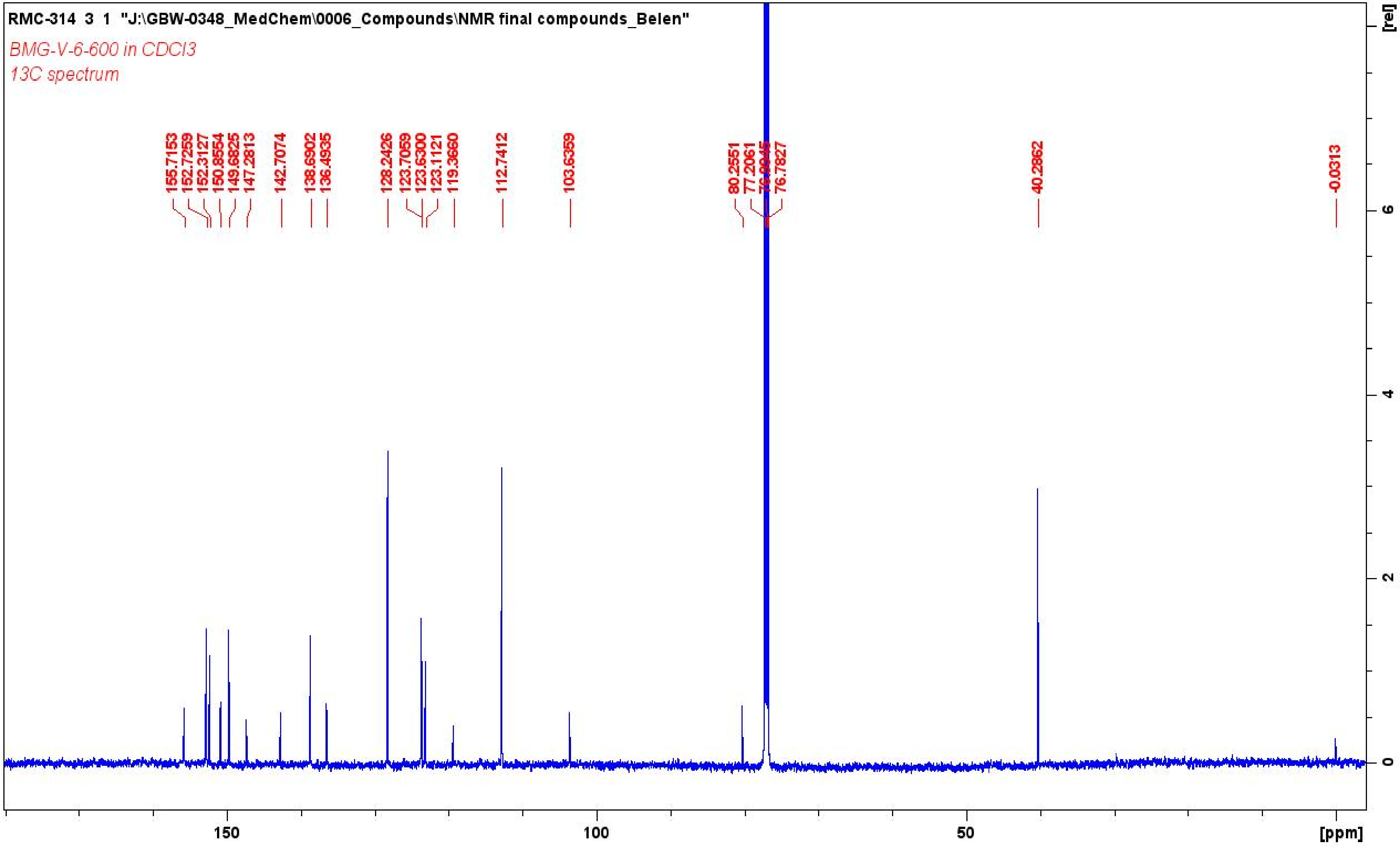

^{1}H NMR spectrum of **3e**

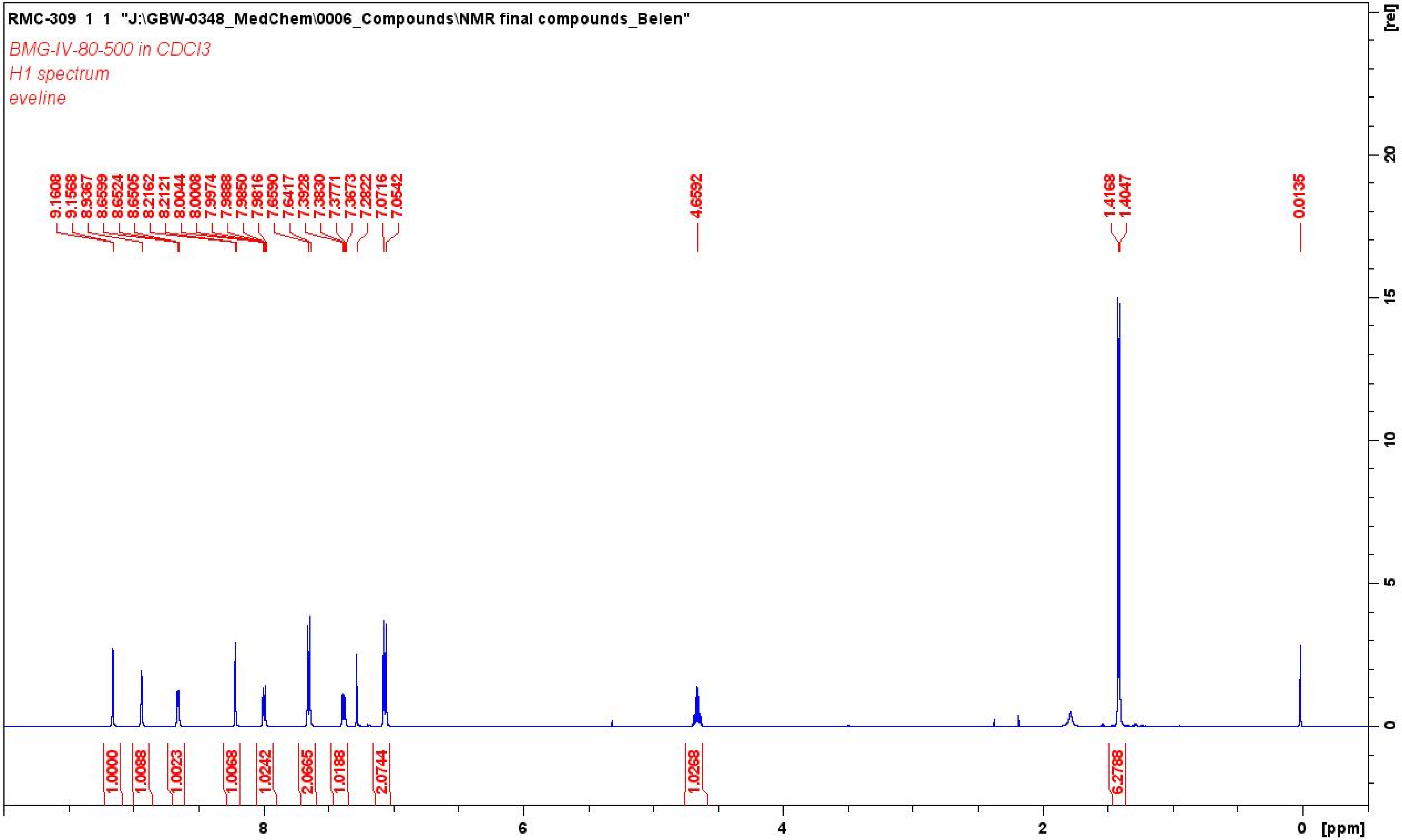

^{13}C NMR spectrum of **3e**

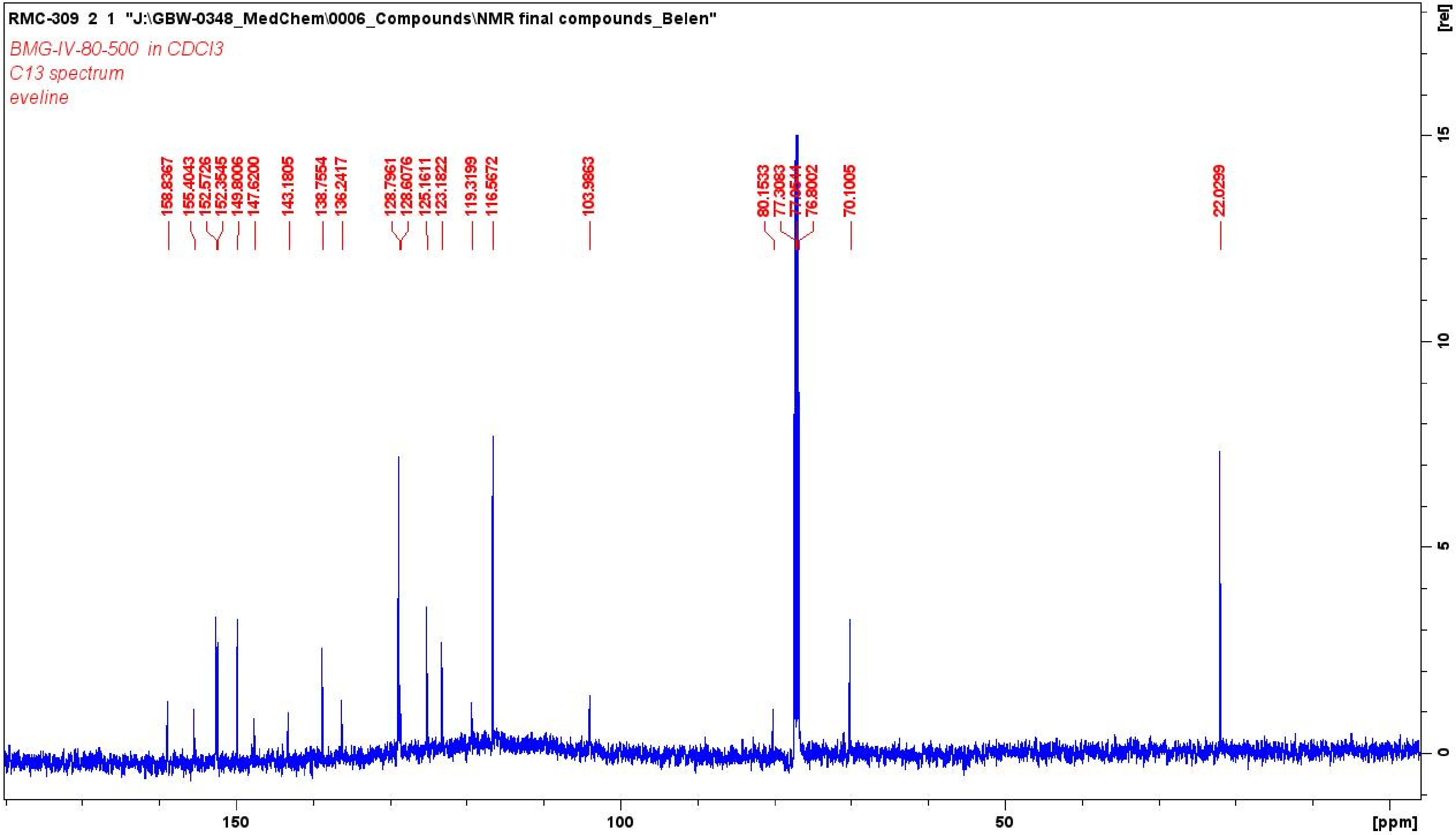

^{1}H NMR spectrum of **3f**

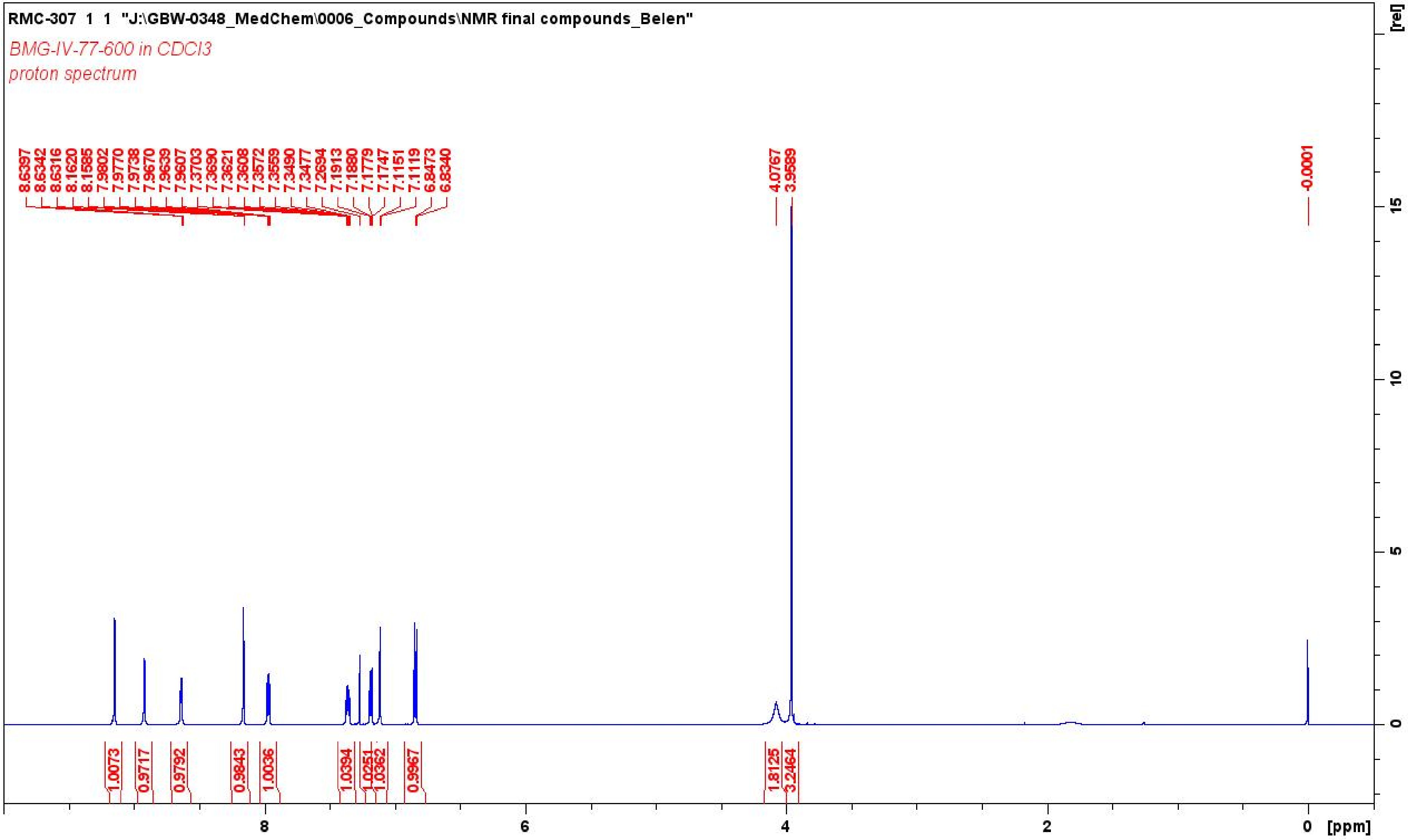

^{13}C NMR spectrum of **3f**

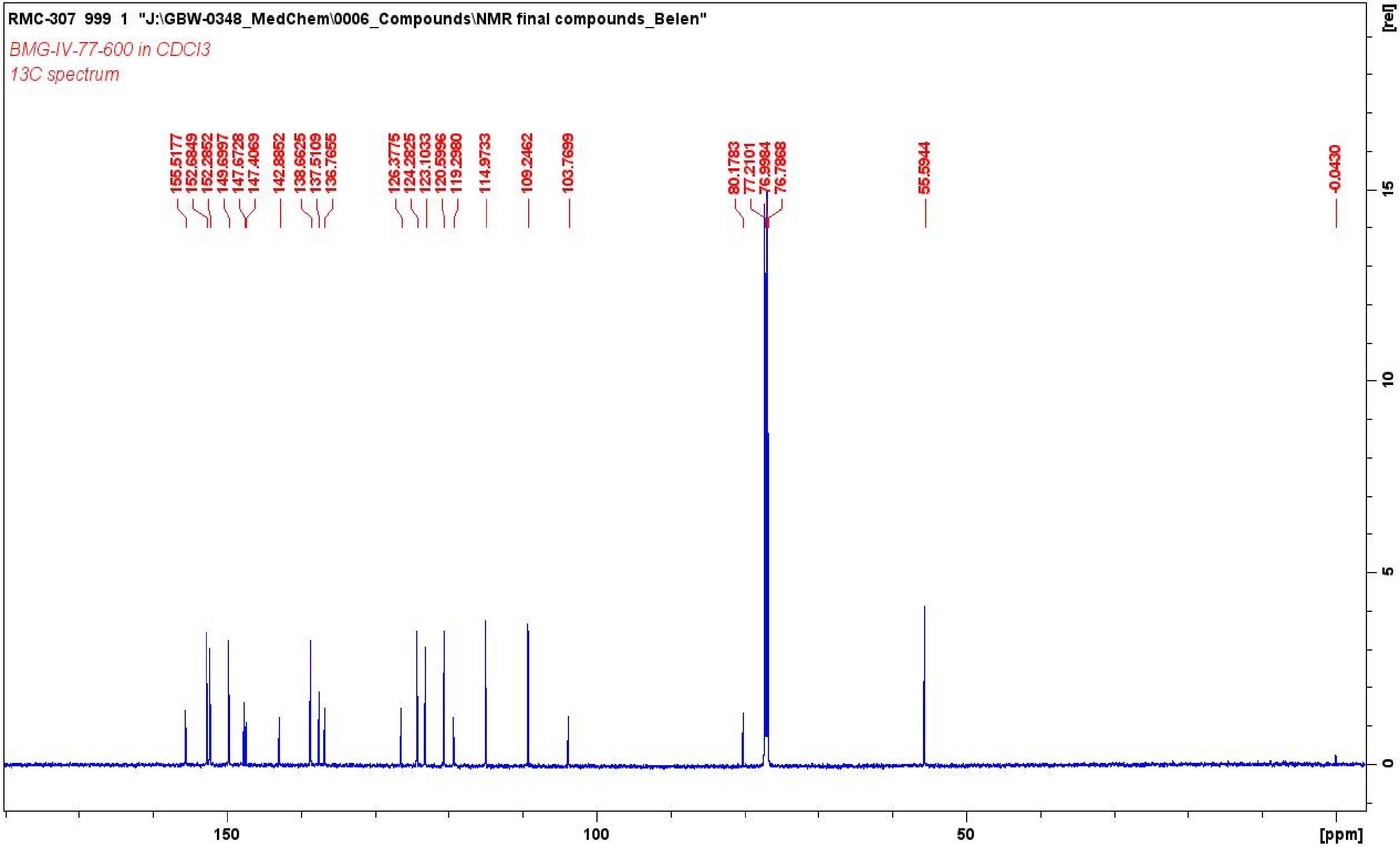

^{1}H NMR spectrum of **3g**

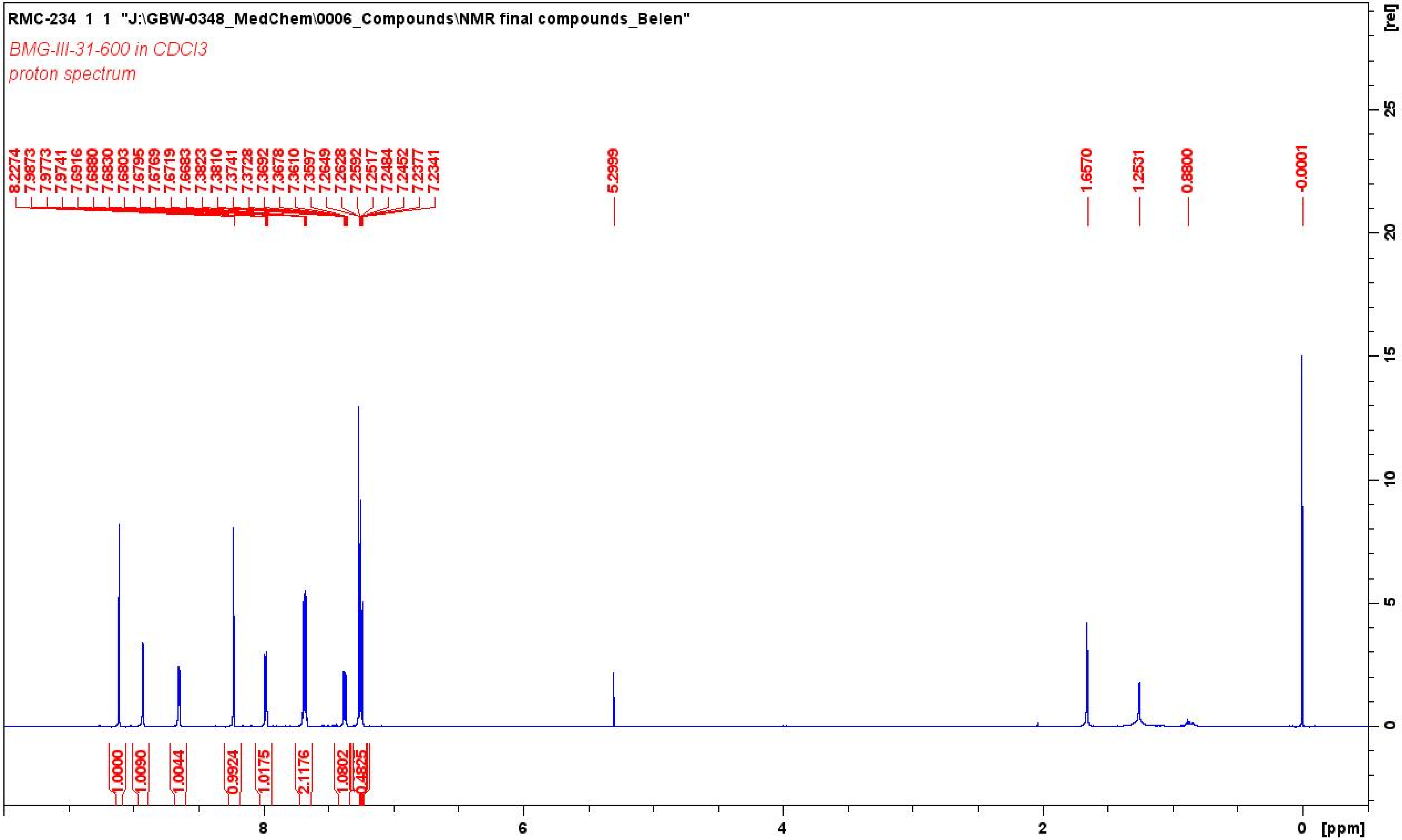

^{13}C NMR spectrum of **3g**

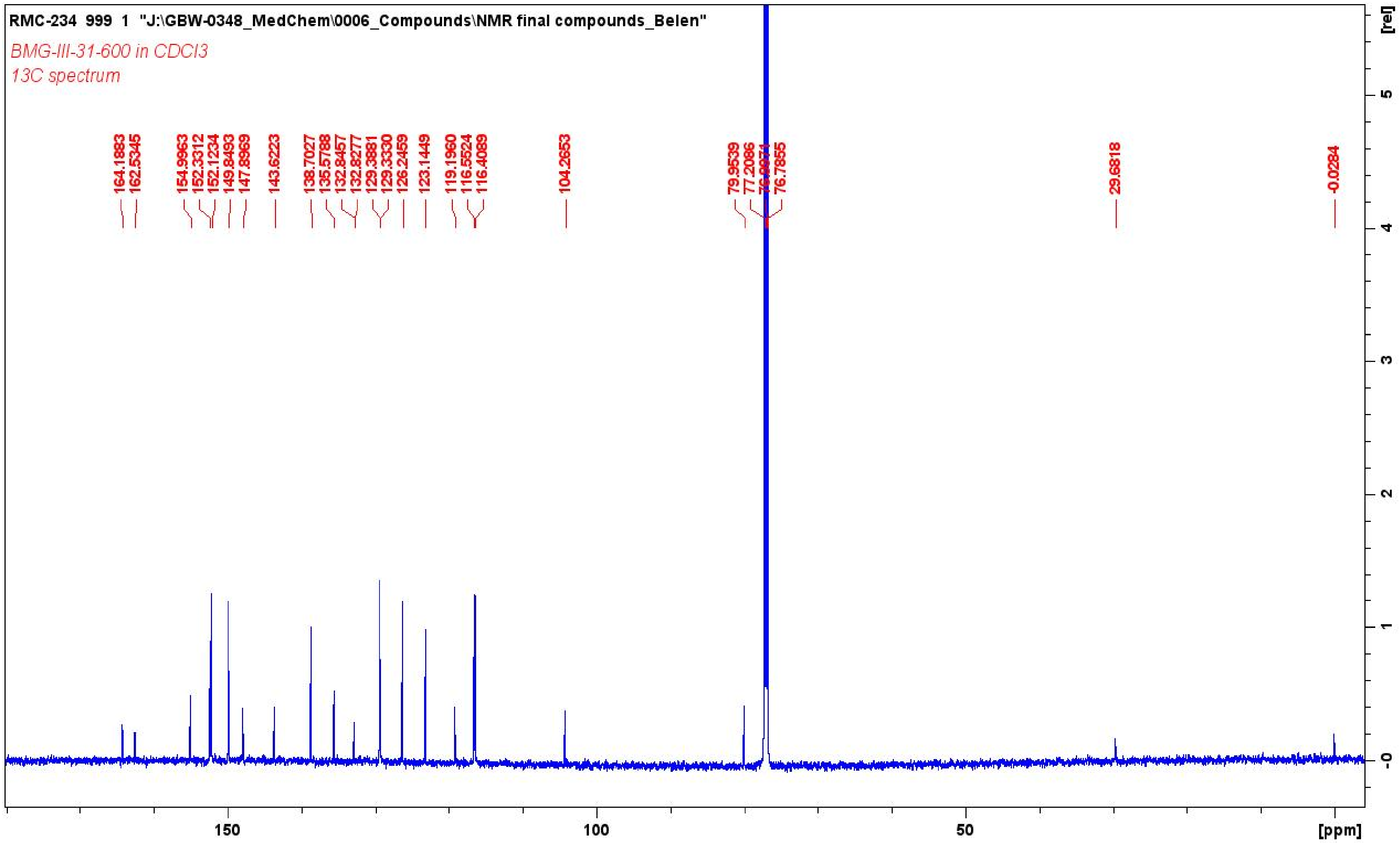

^{1}H NMR spectrum of **3h**

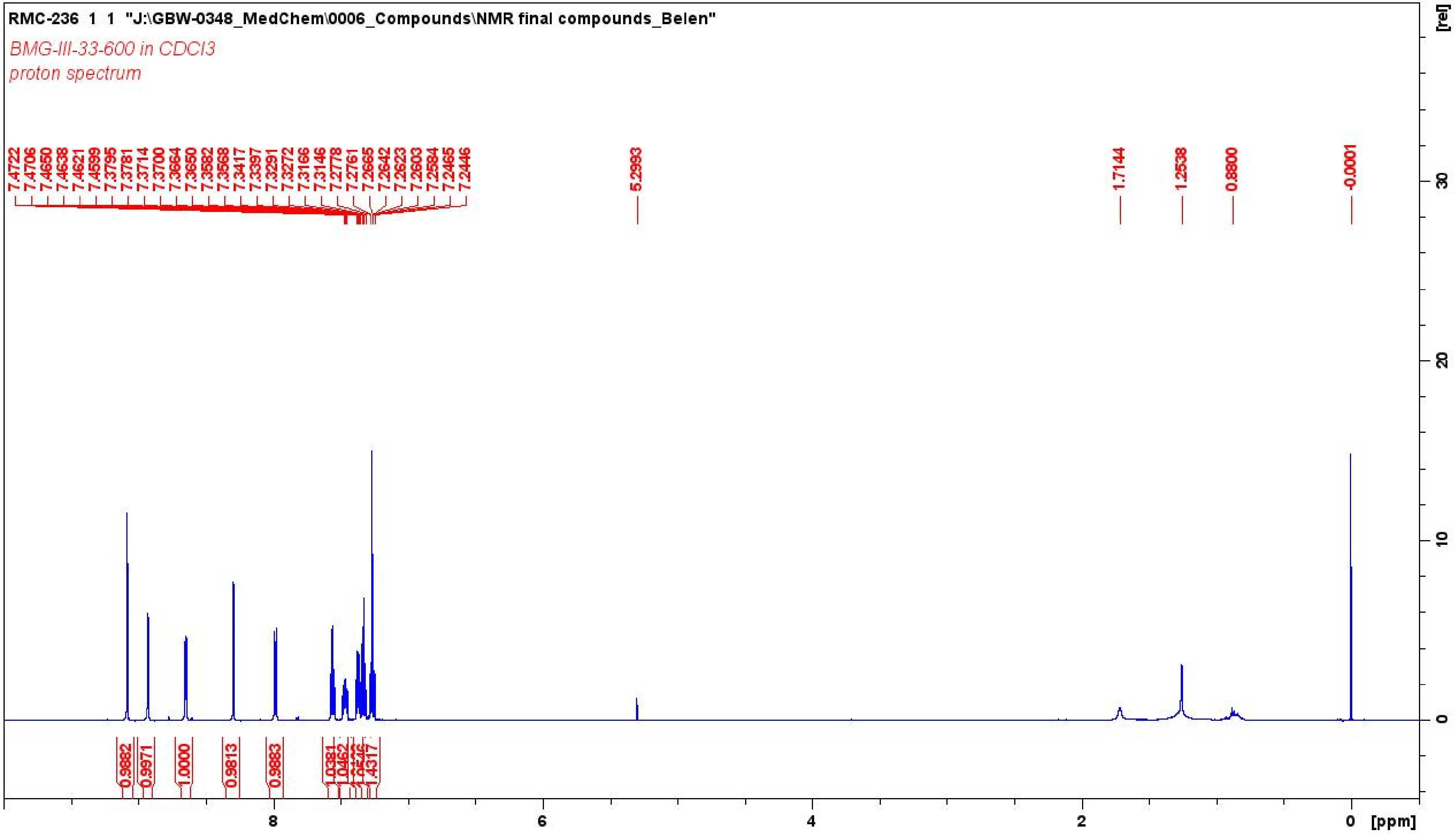

^{13}C NMR spectrum of **3h**

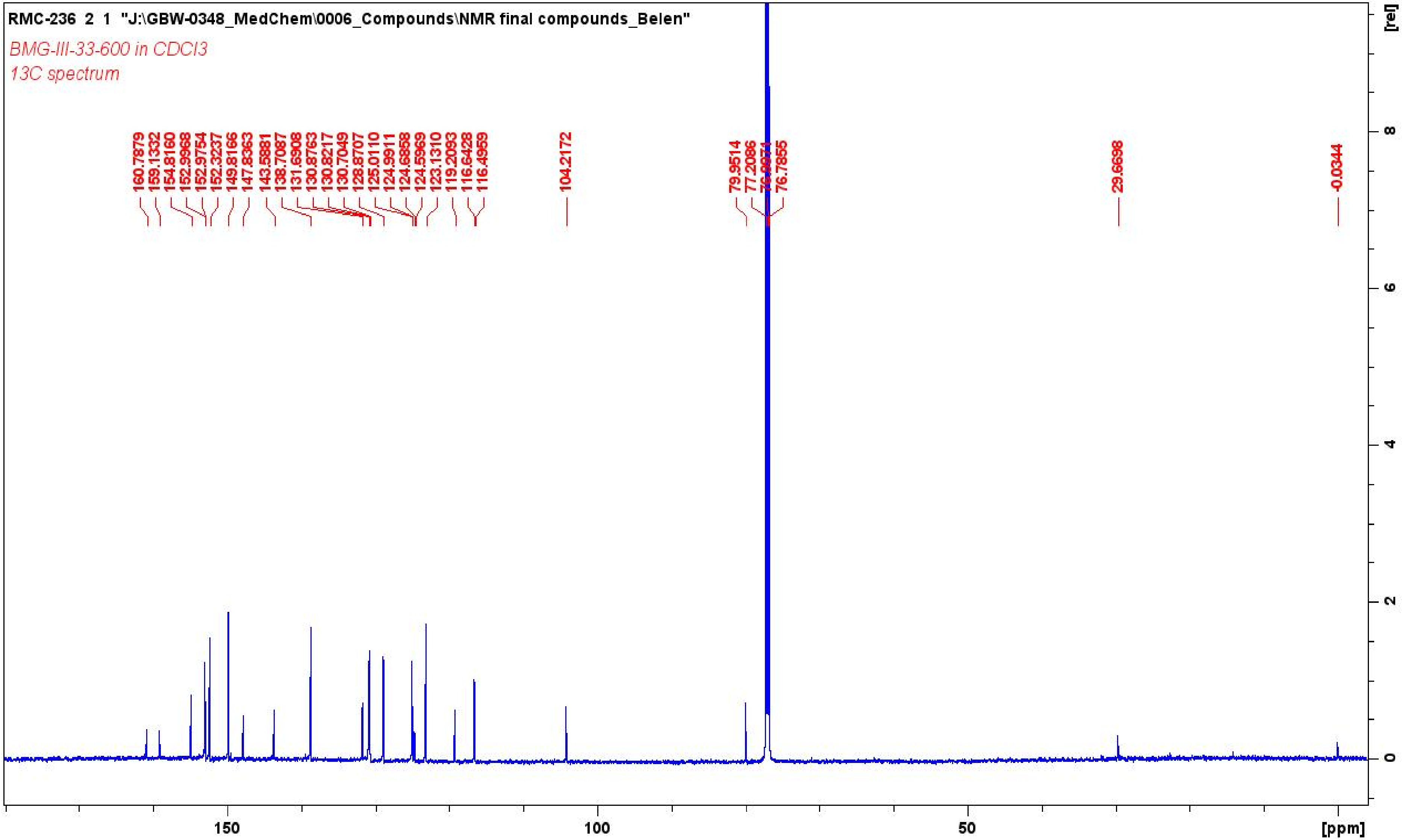

^{1}H NMR spectrum of **3i**

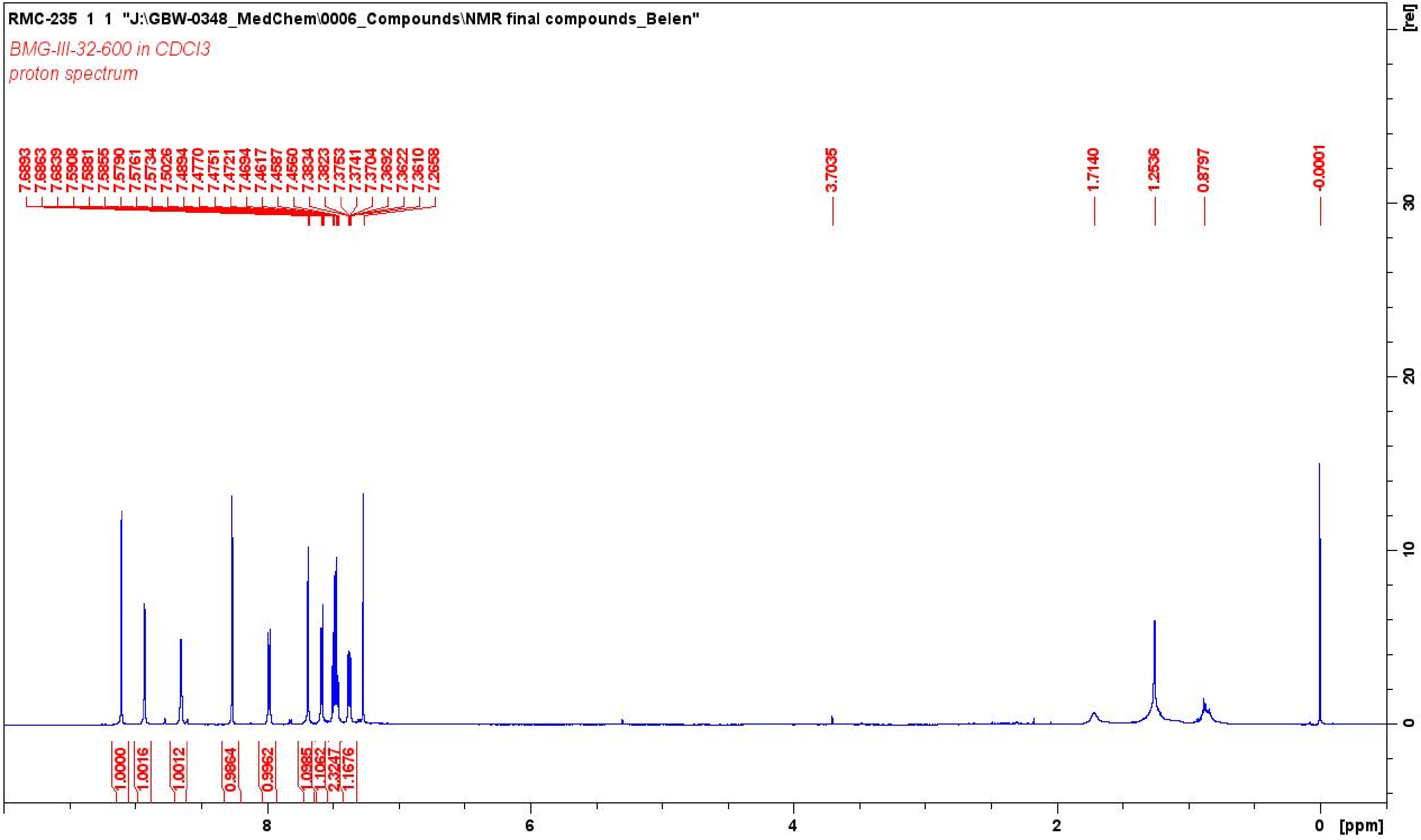

^{13}C NMR spectrum of **3i**

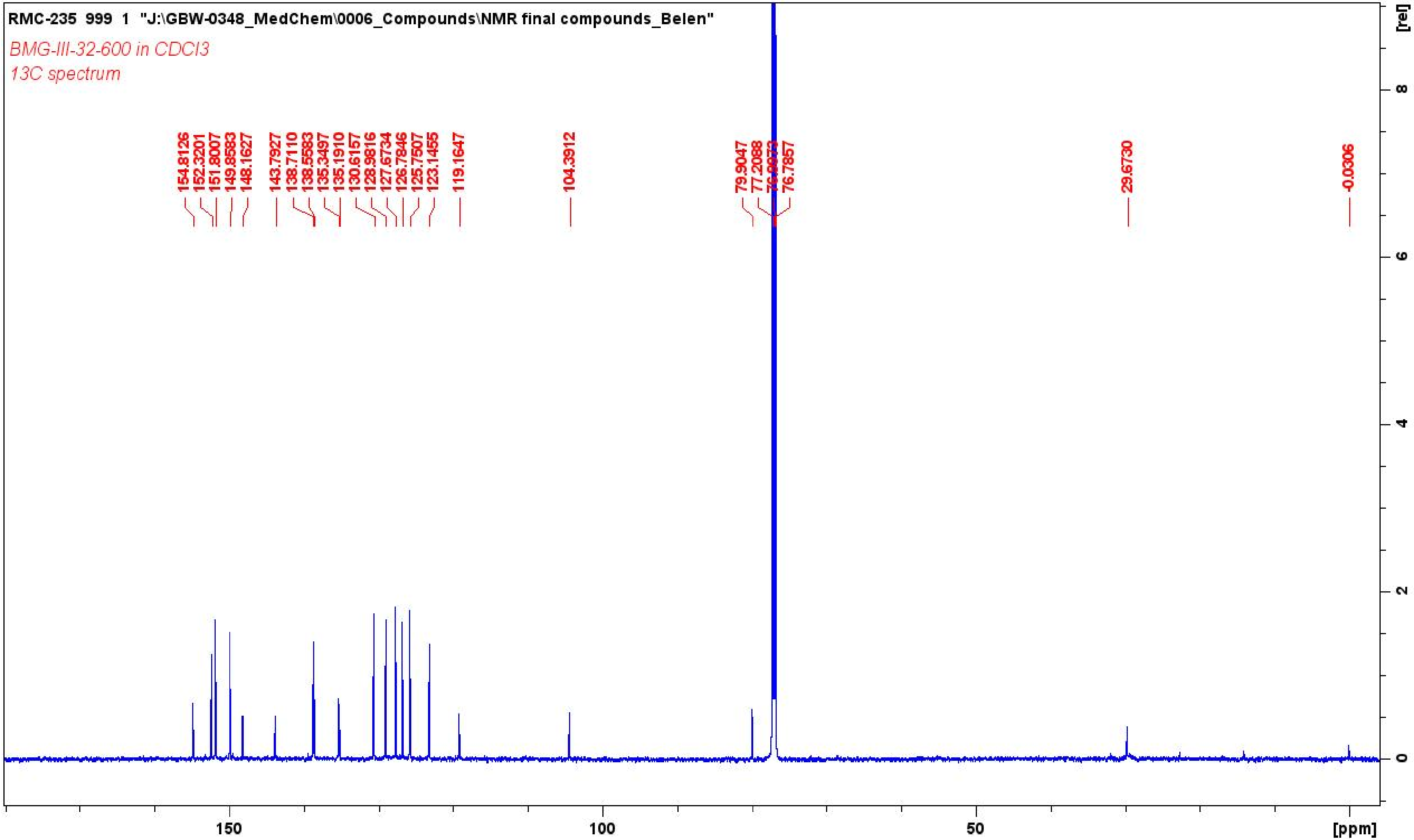

^{1}H NMR spectrum of **3j**

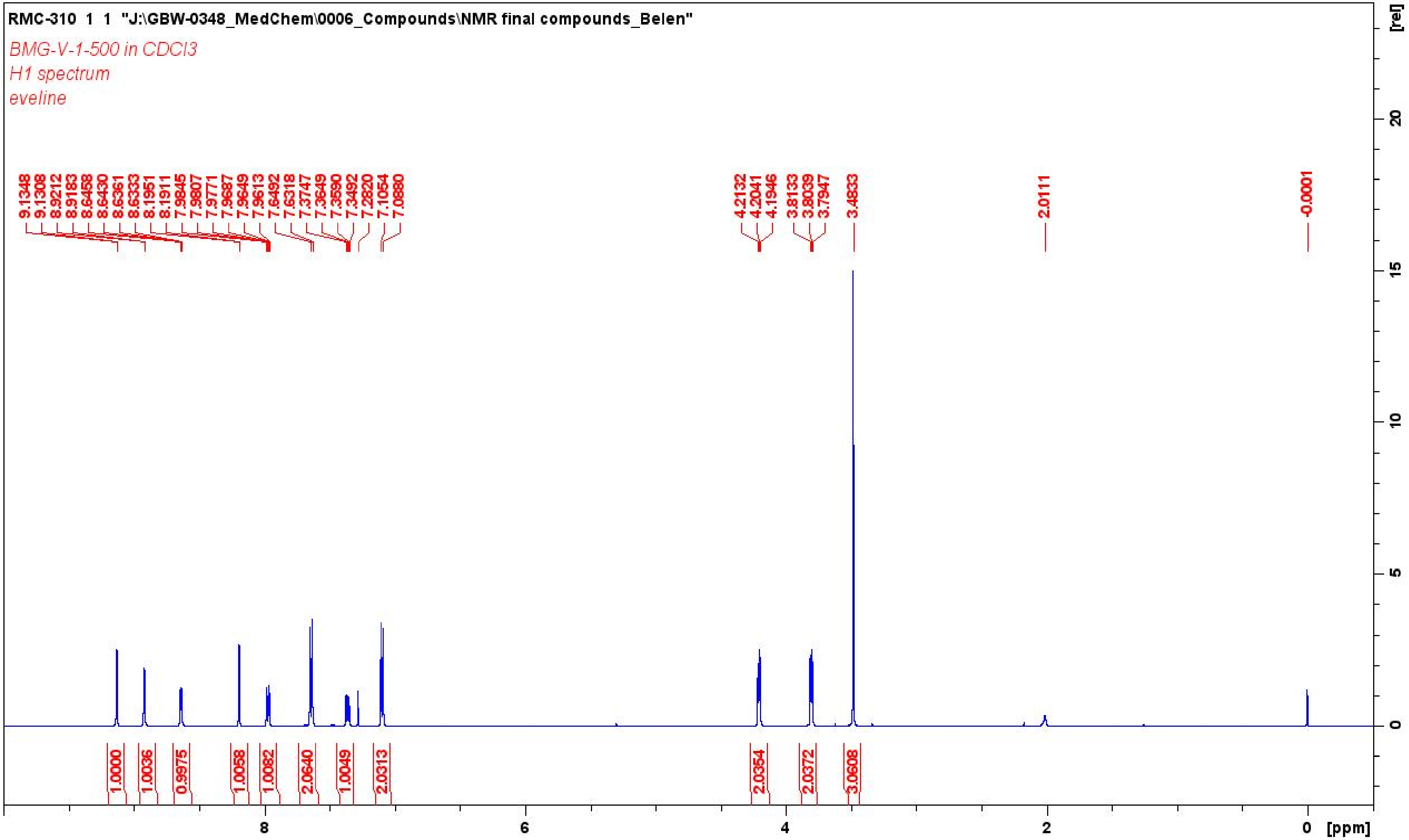

^{13}C NMR spectrum of **3j**

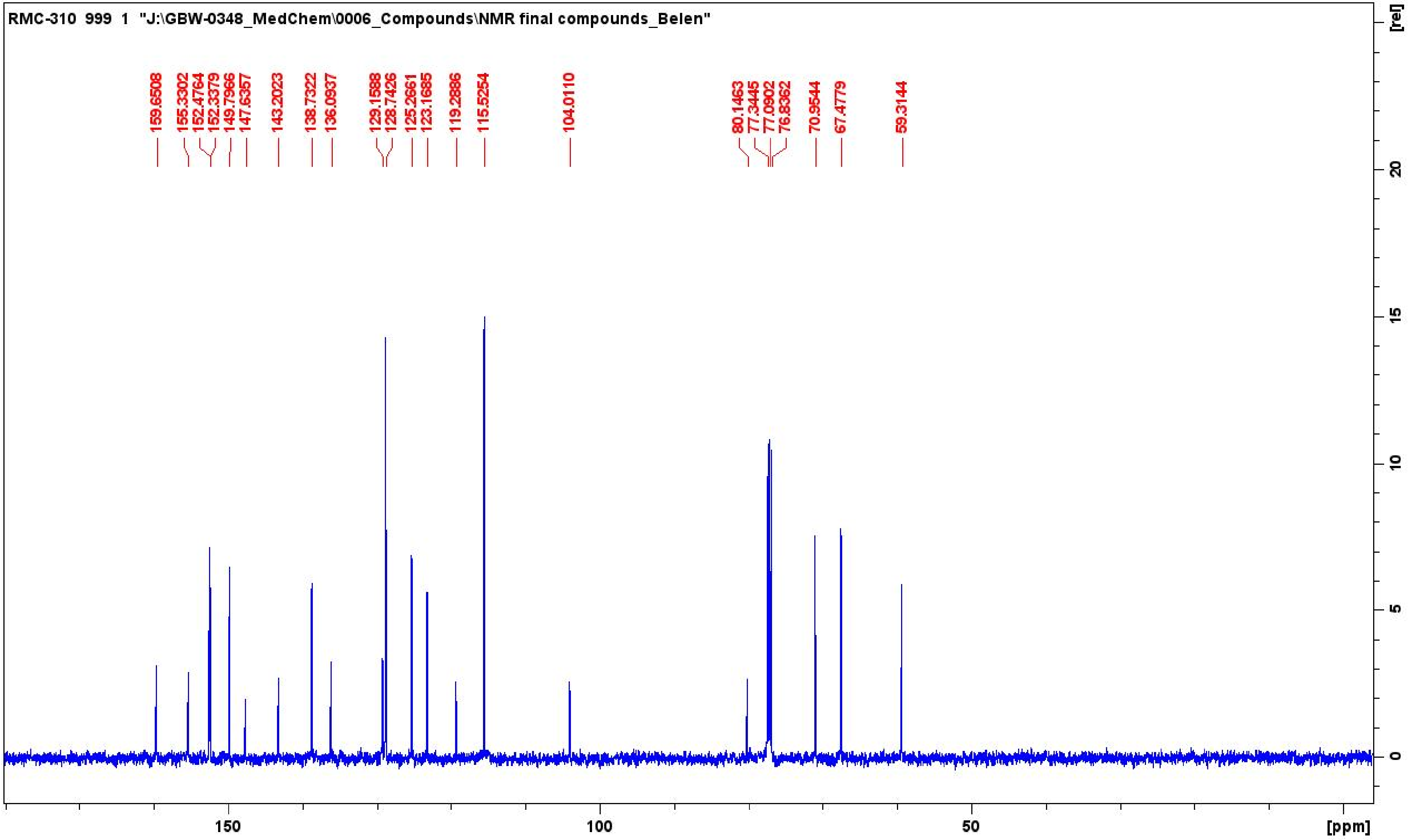

^{1}H NMR spectrum of **3l**

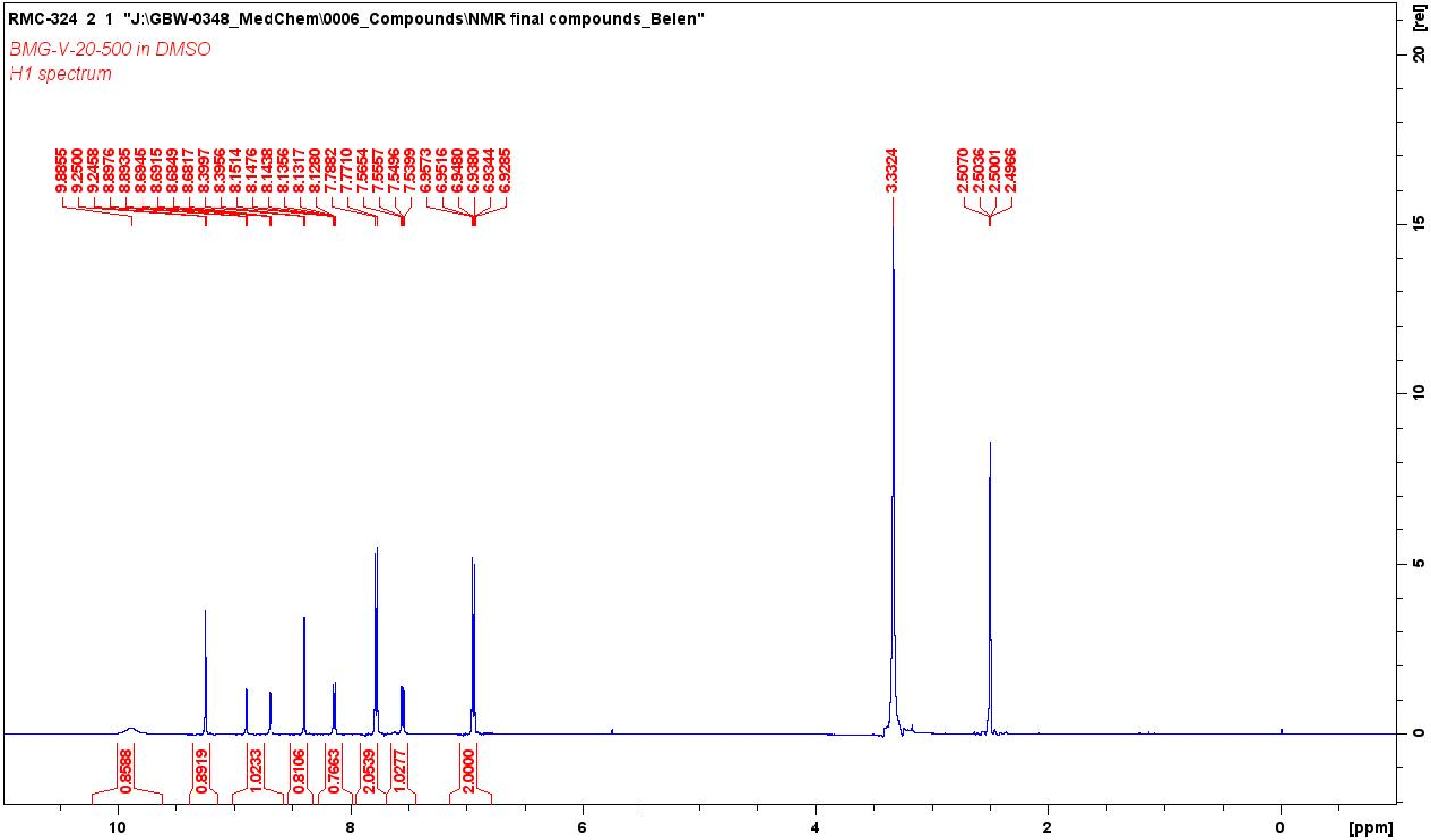

^{13}C NMR spectrum of **3l**

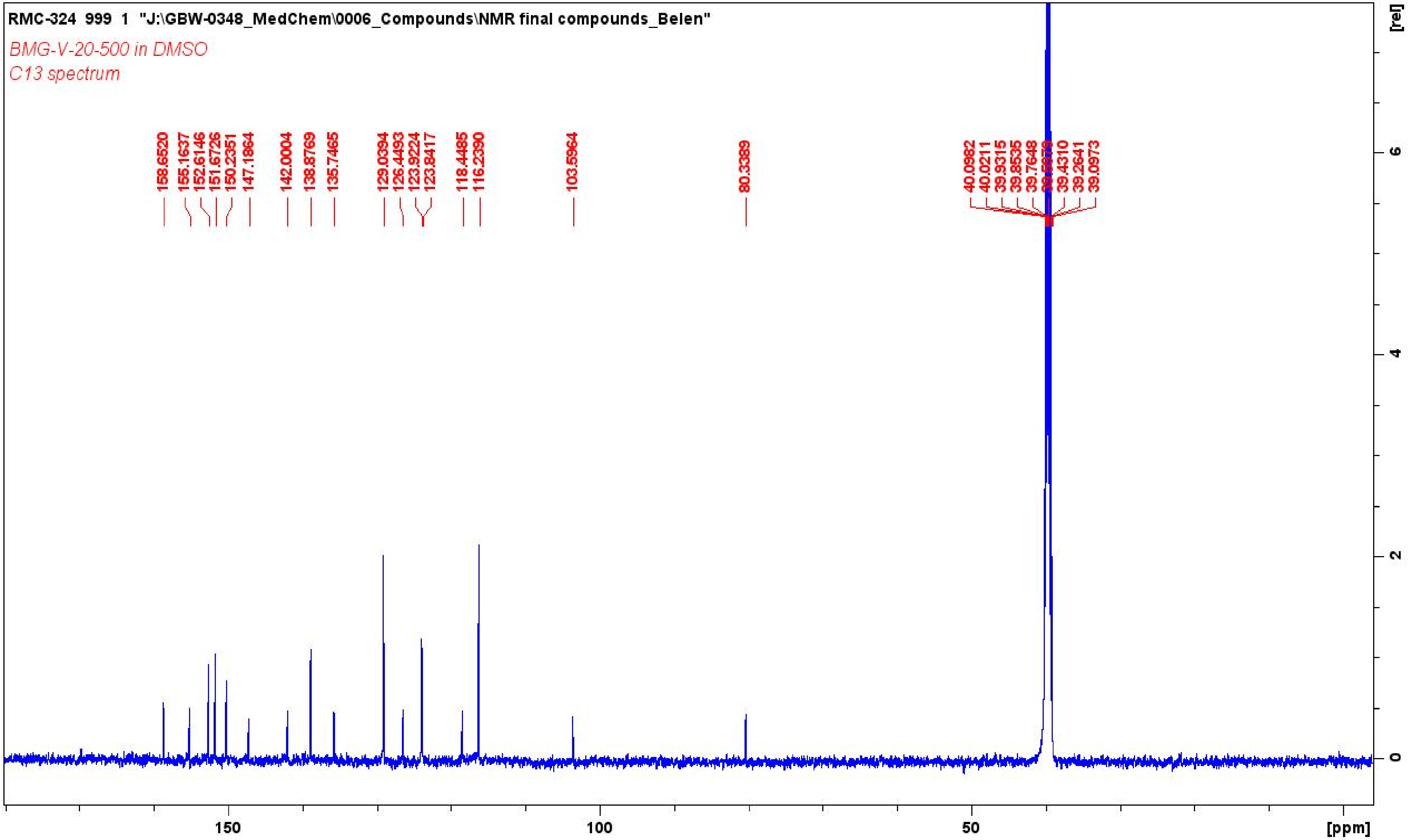

^{1}H NMR spectrum of **3m**

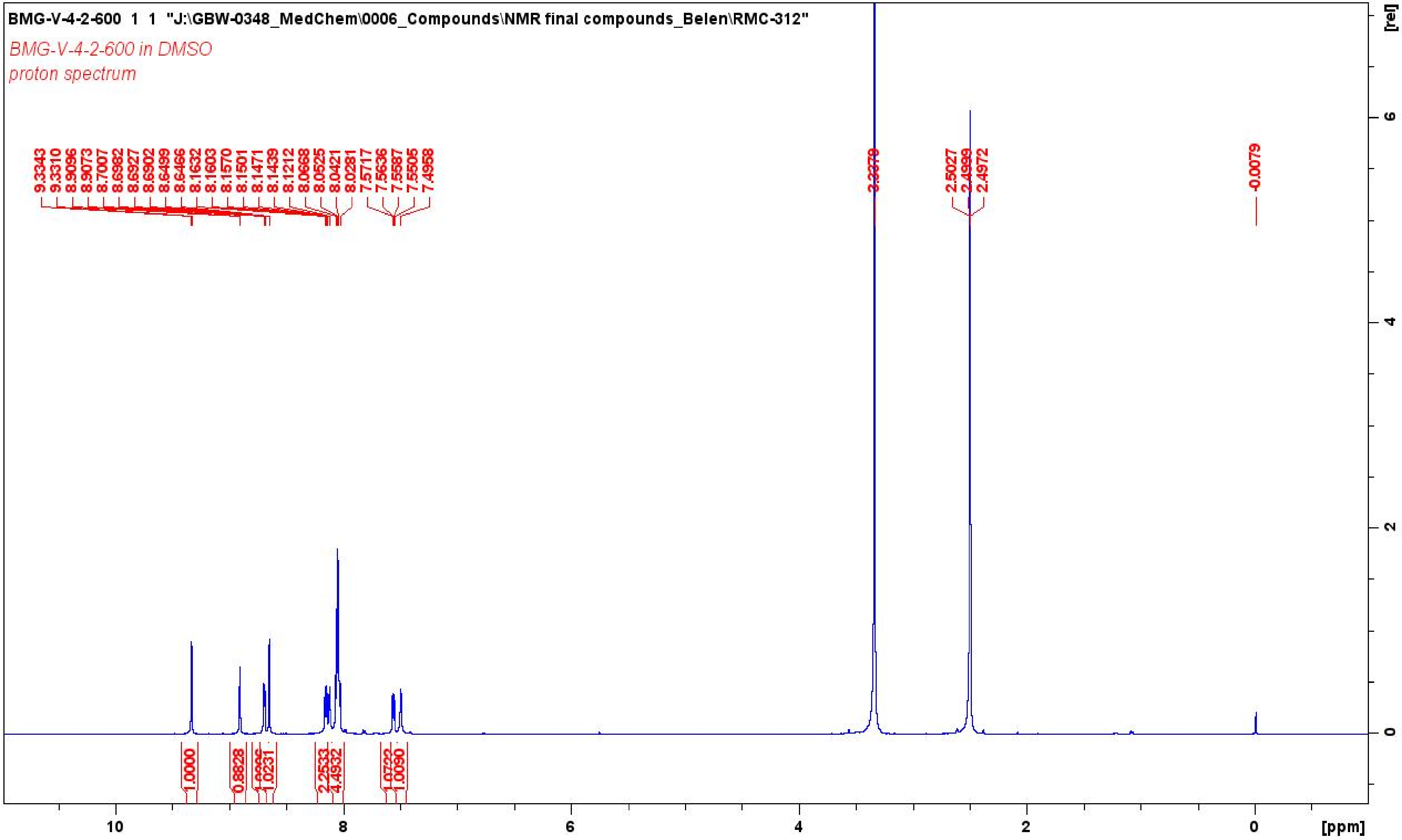

^{13}C NMR spectrum of **3m**

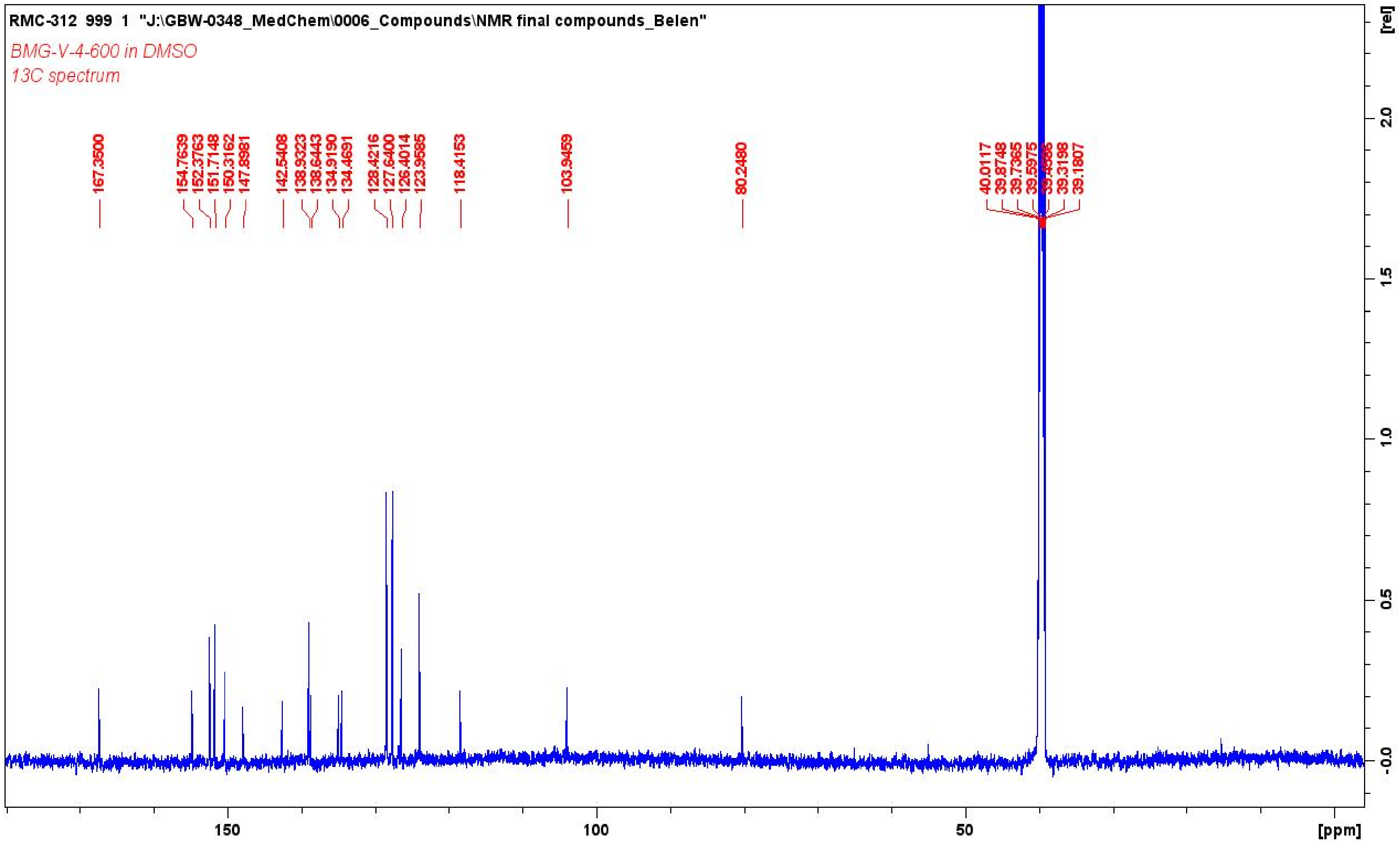

^{1}H NMR spectrum of **3n**

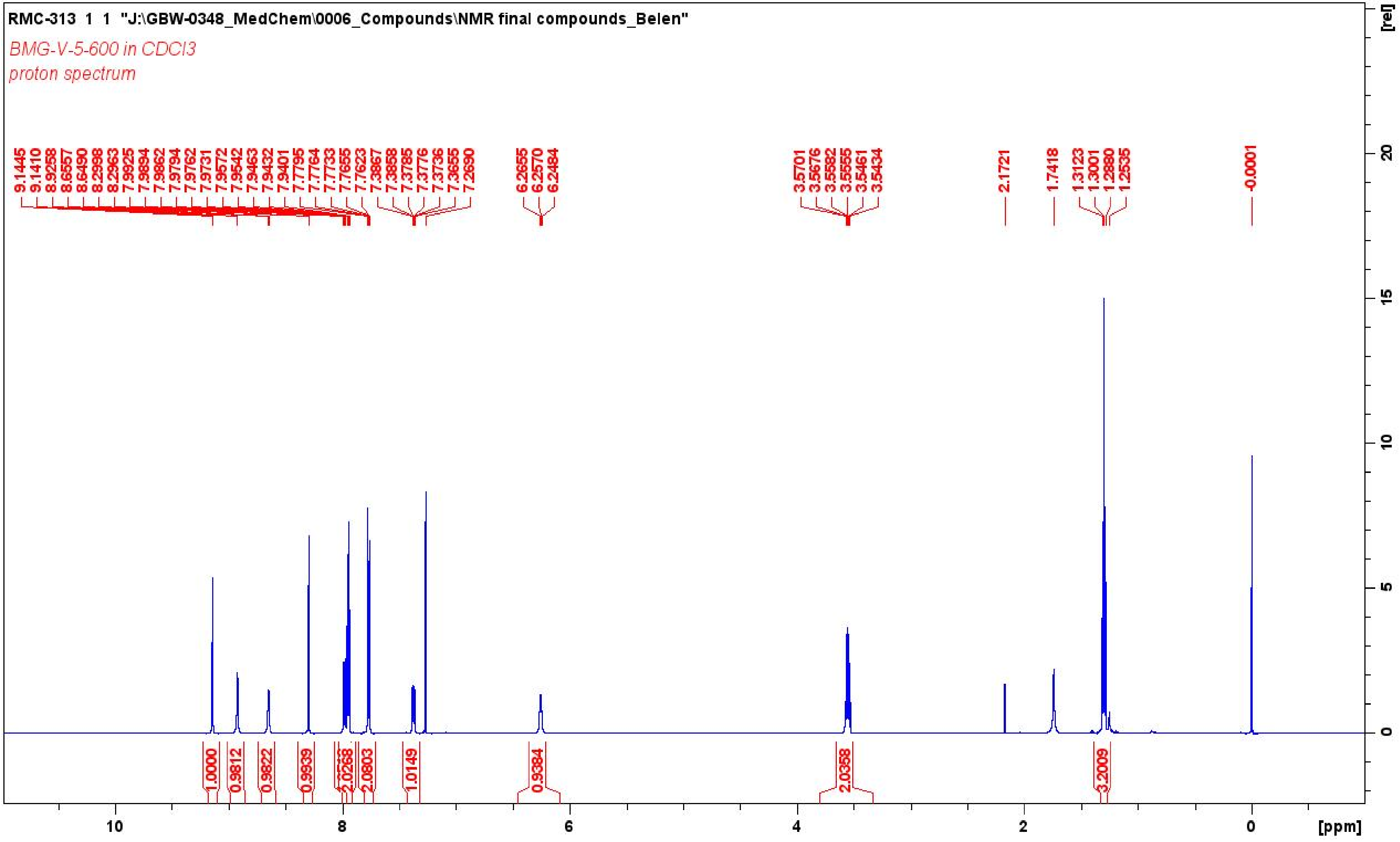

^{13}C NMR spectrum of **3n**

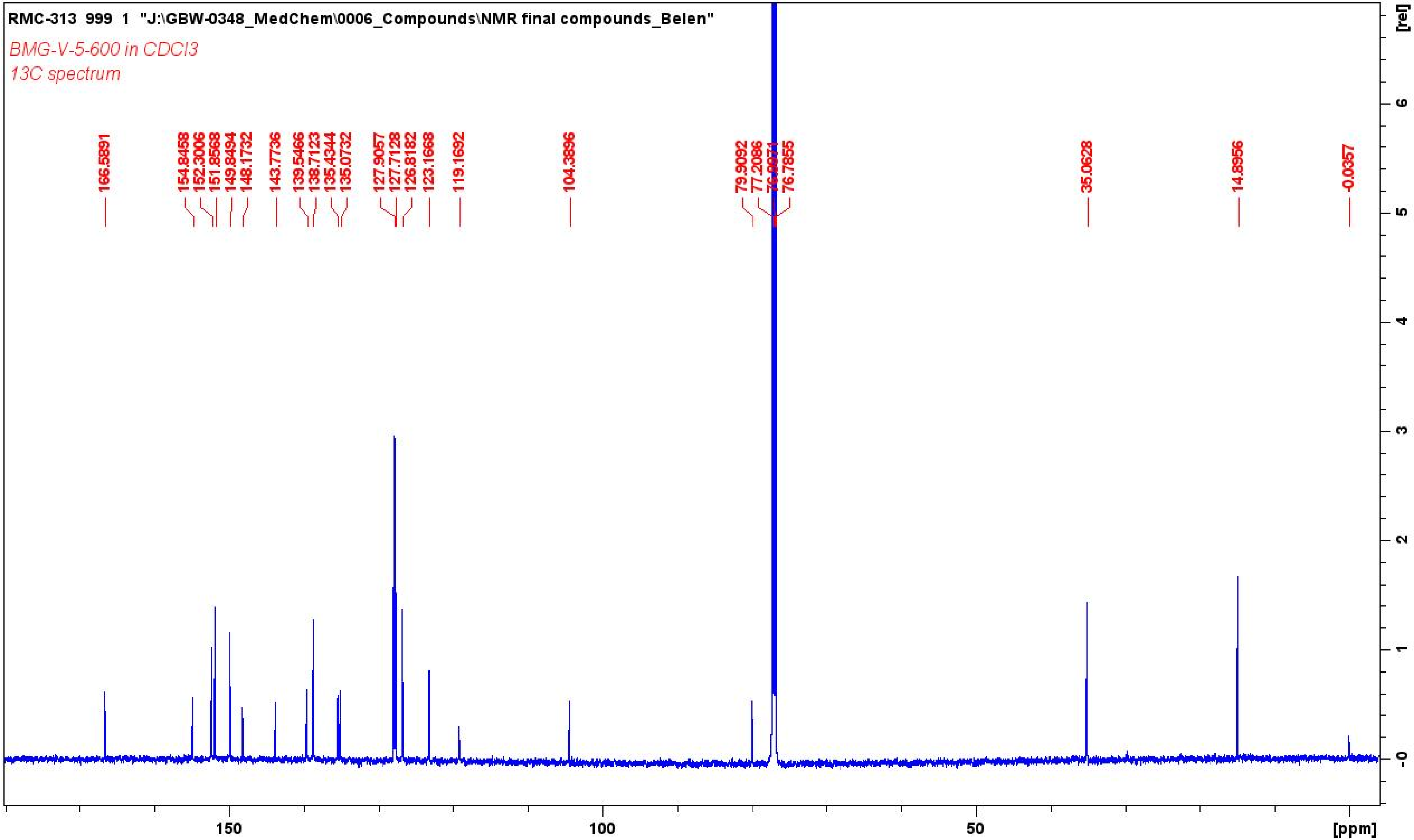

^{1}H NMR spectrum of **3o**

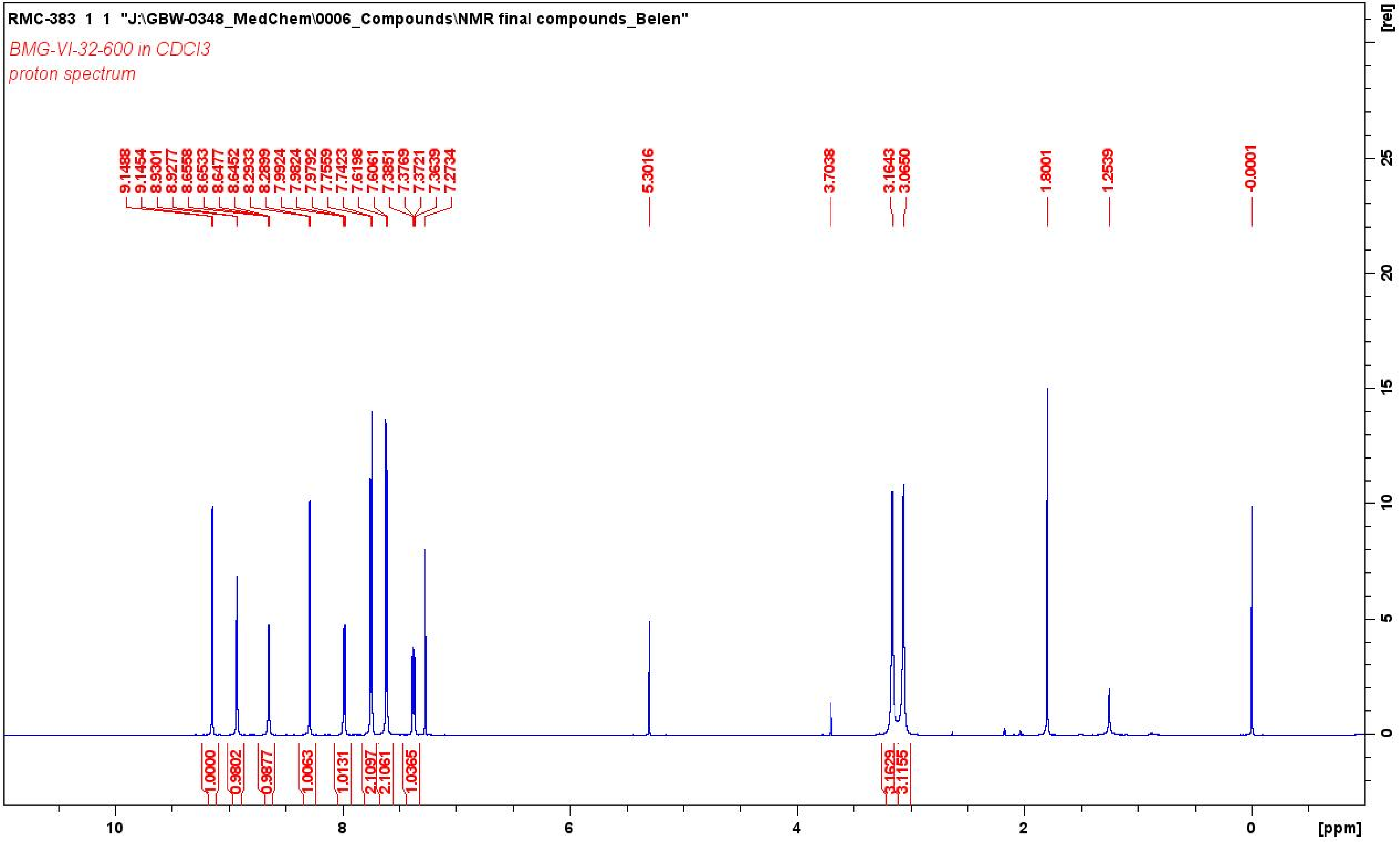

^{13}C NMR spectrum of **3o**

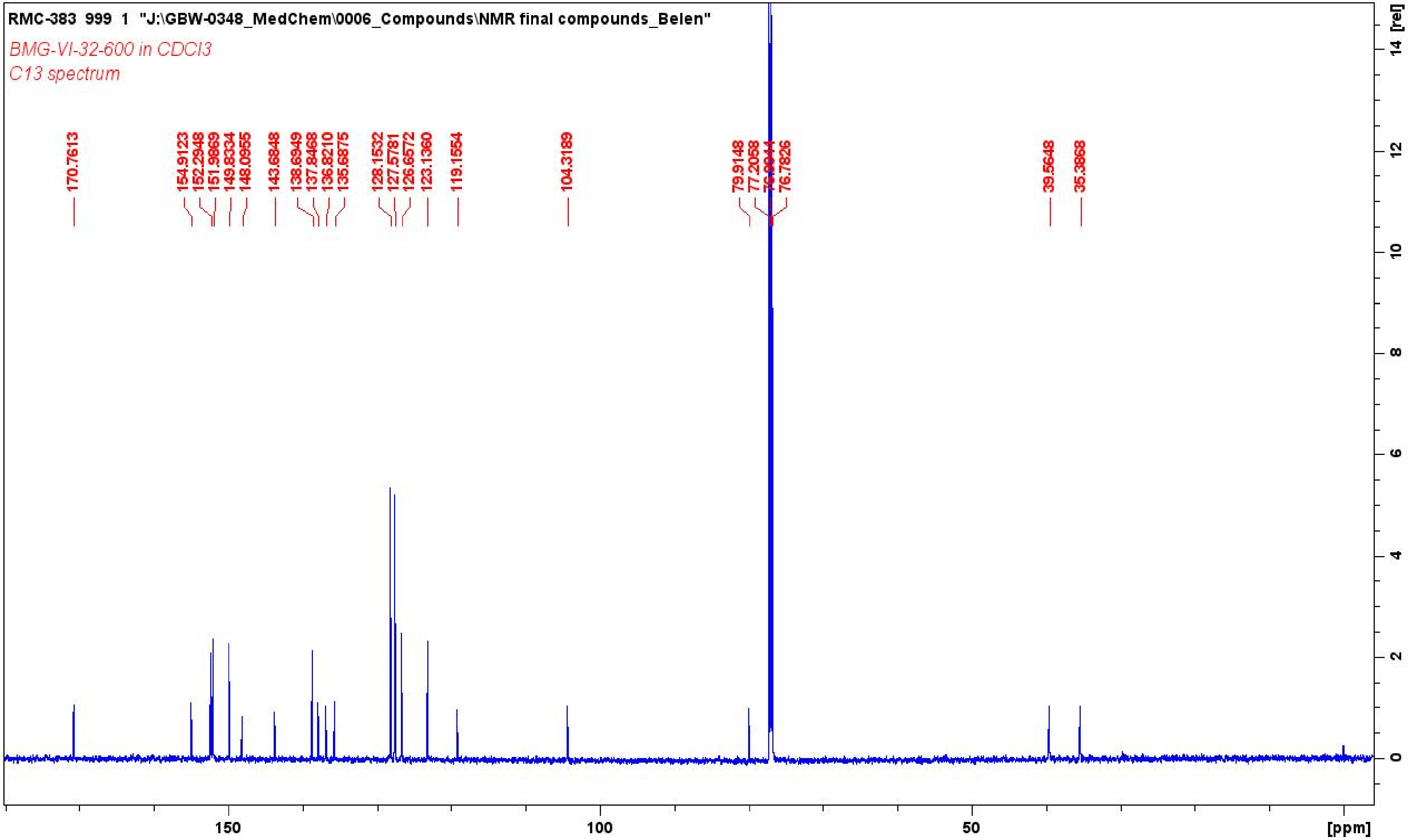

^{1}H NMR spectrum of **3p**

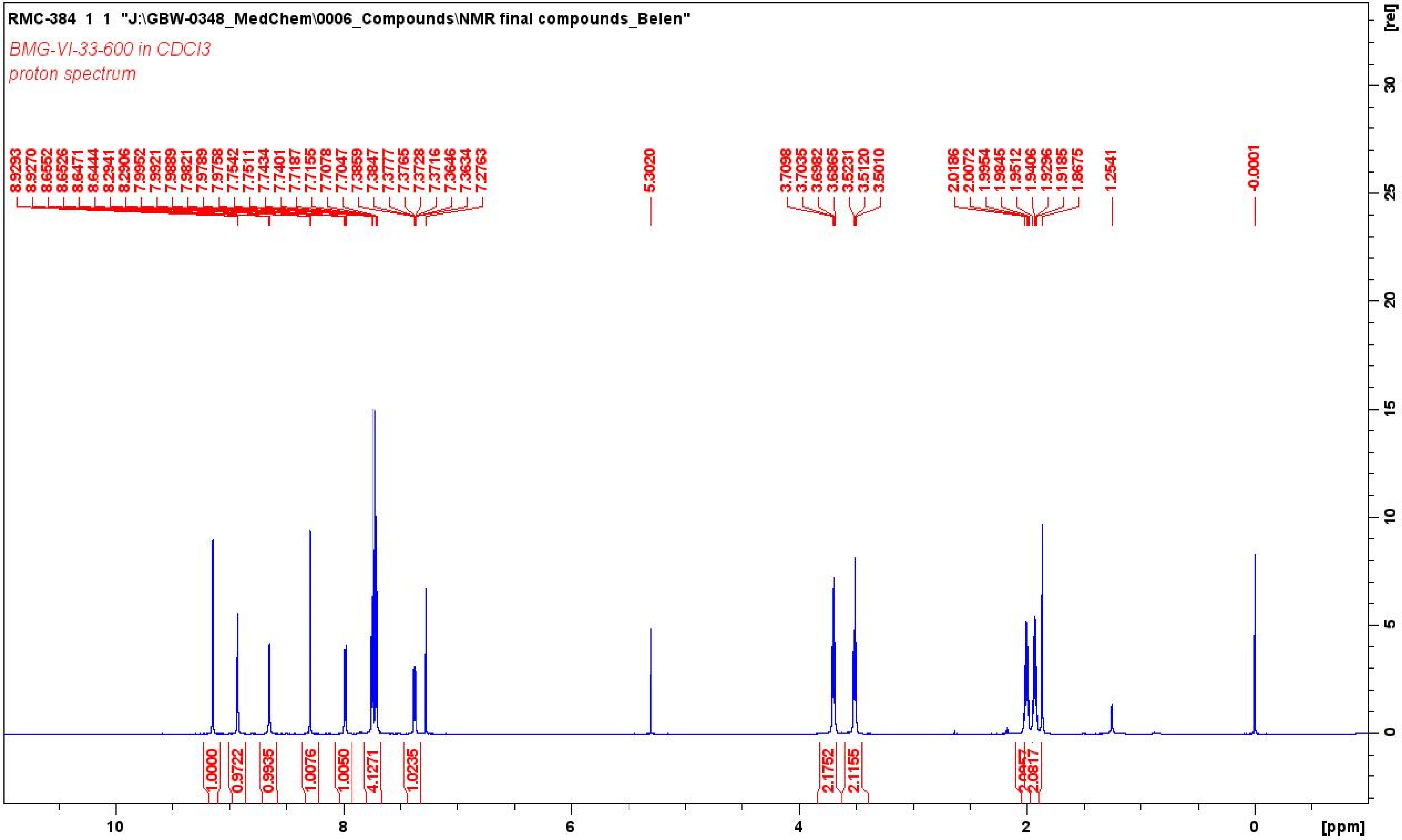

^{13}C NMR spectrum of **3p**

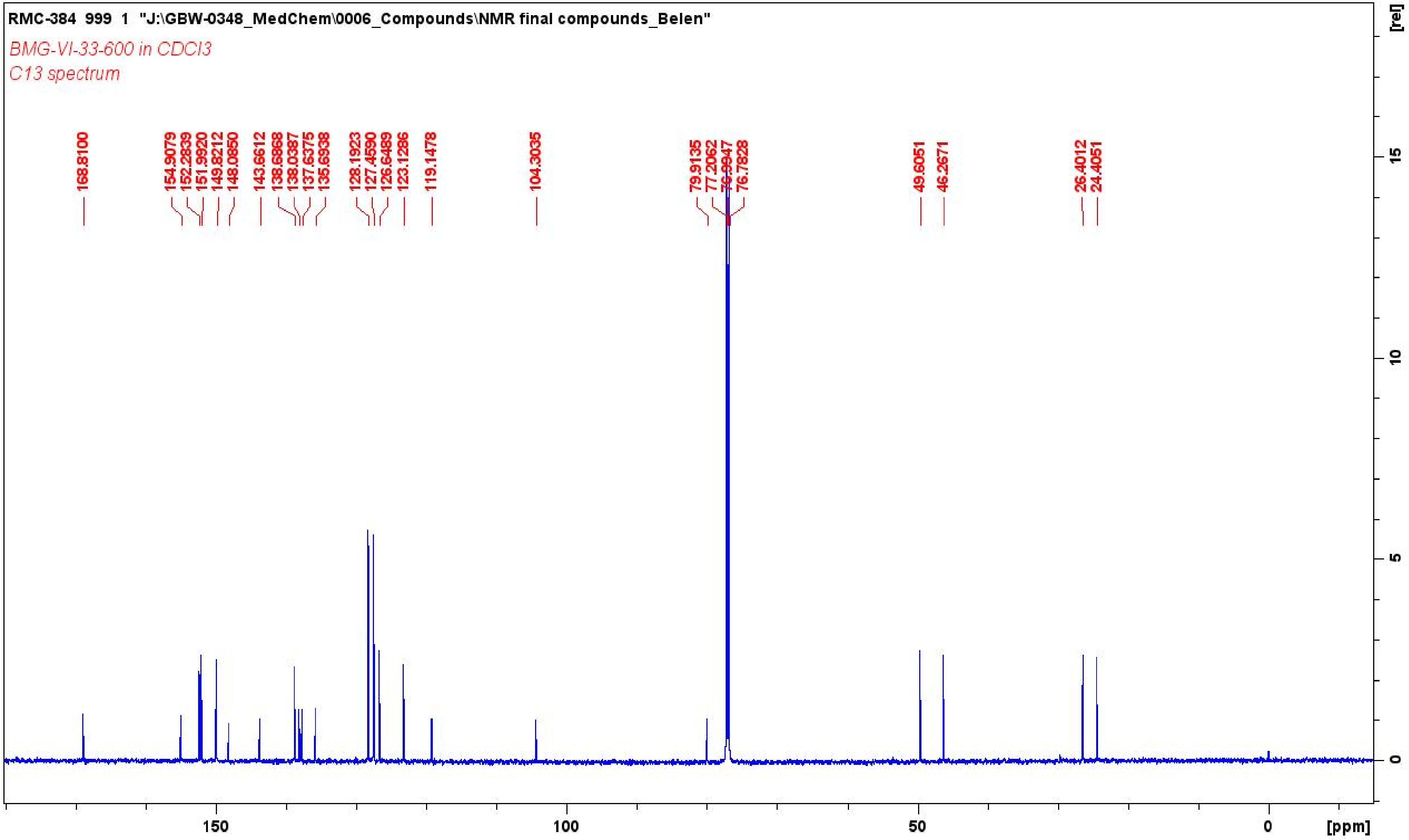

^{1}H NMR spectrum of **3q**

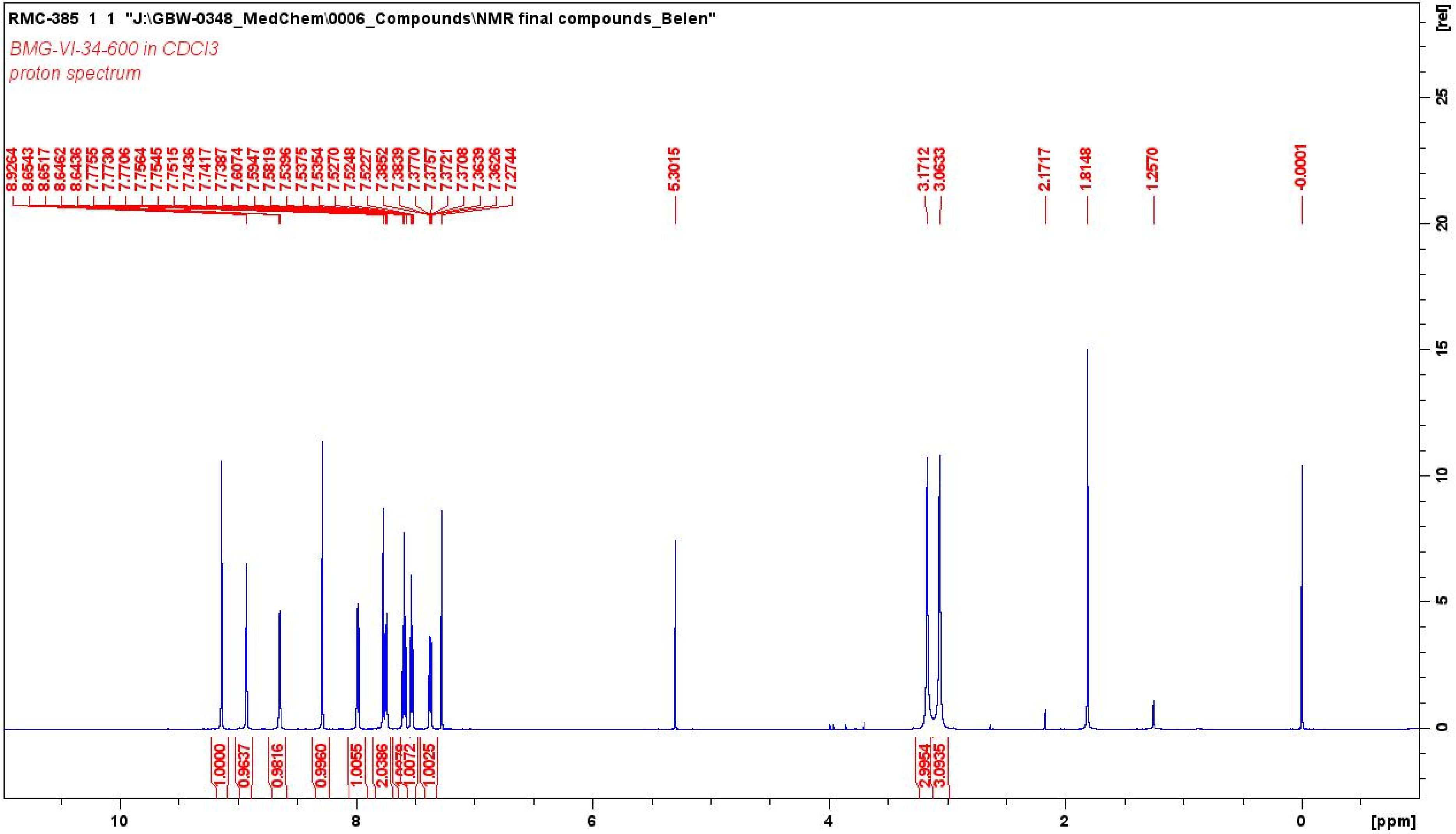

^{13}C NMR spectrum of **3q**

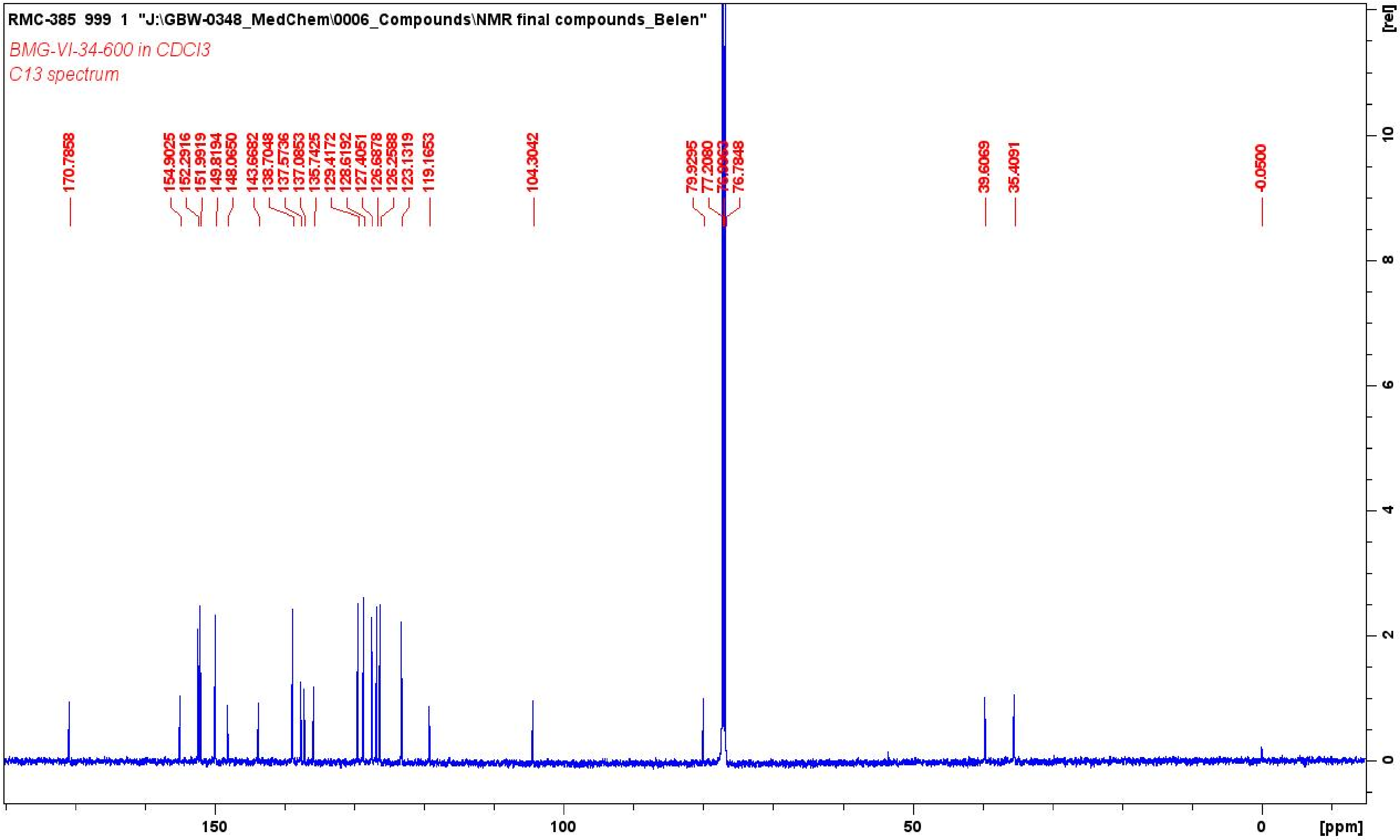

^{1}H NMR spectrum of **3r**

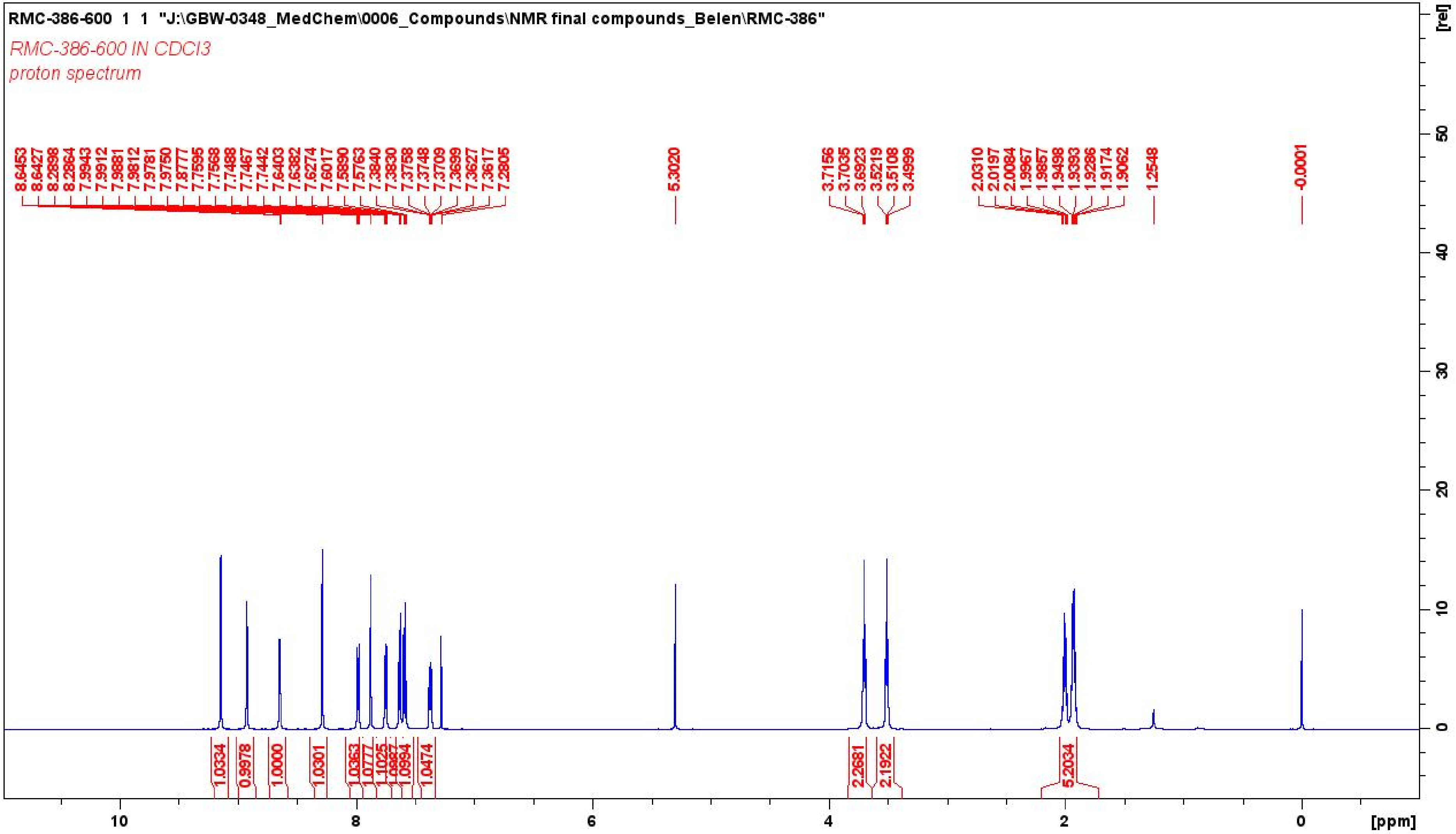

^{13}C NMR spectrum of **3r**

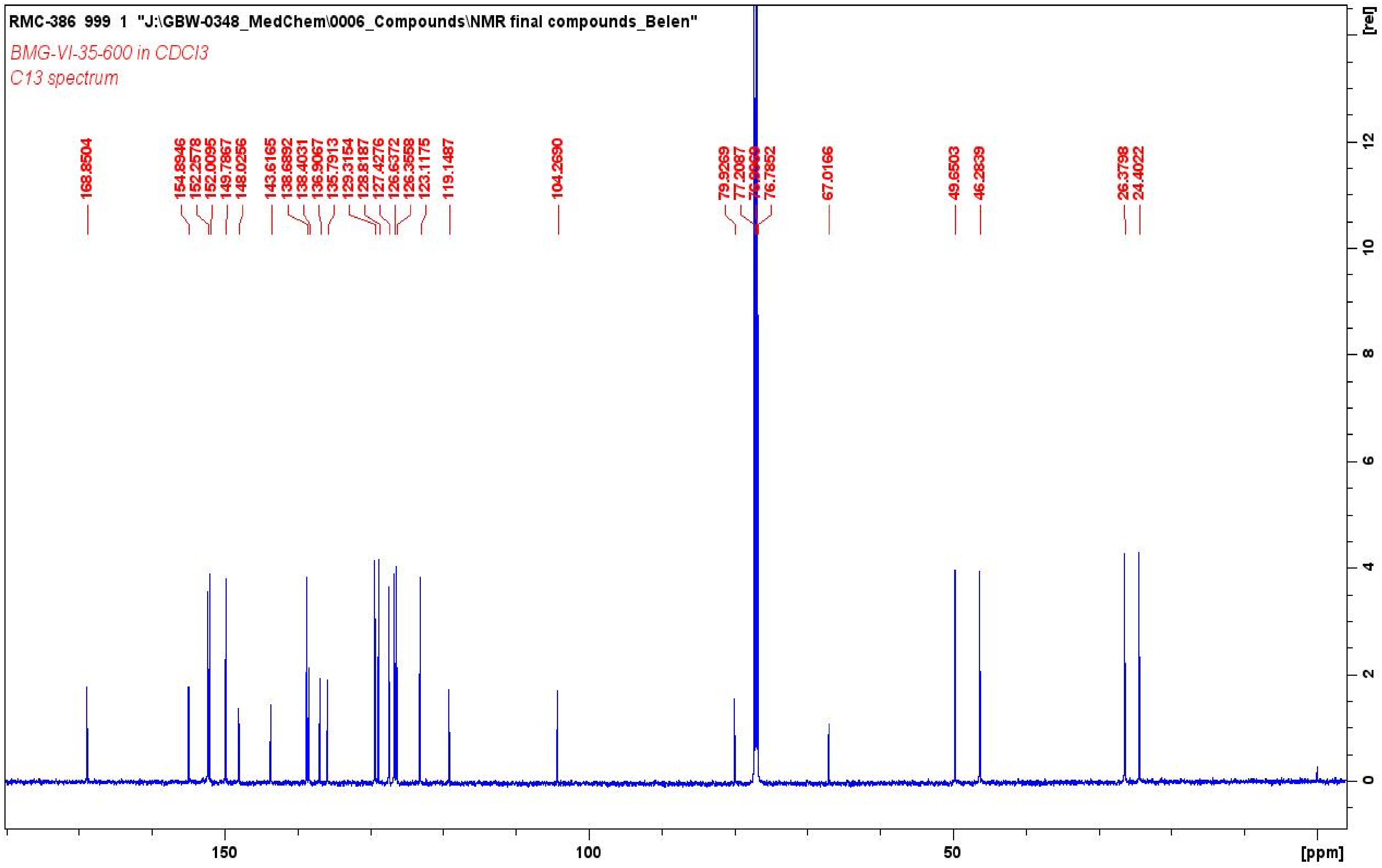

RMC-386 999 1 "J:\GBW-0348_MedChem\0006_Compounds\NMR final compounds_Belen"
BMG-VI-35-600 in CDCl3
C13 spectrum
168.8504
154.8946
152.2578
152.0095
149.7867
148.0256
143.6165
138.6892
138.4031
136.9067
135.7913
129.3154
128.8187
127.4276
126.6372
126.3558
123.1175
119.1487
104.2690
79.9269
77.2087
76.7852
67.0166
49.6503
46.2839
26.3798
24.4022
[rel]
[ppm]

^{1}H NMR spectrum of **3s**

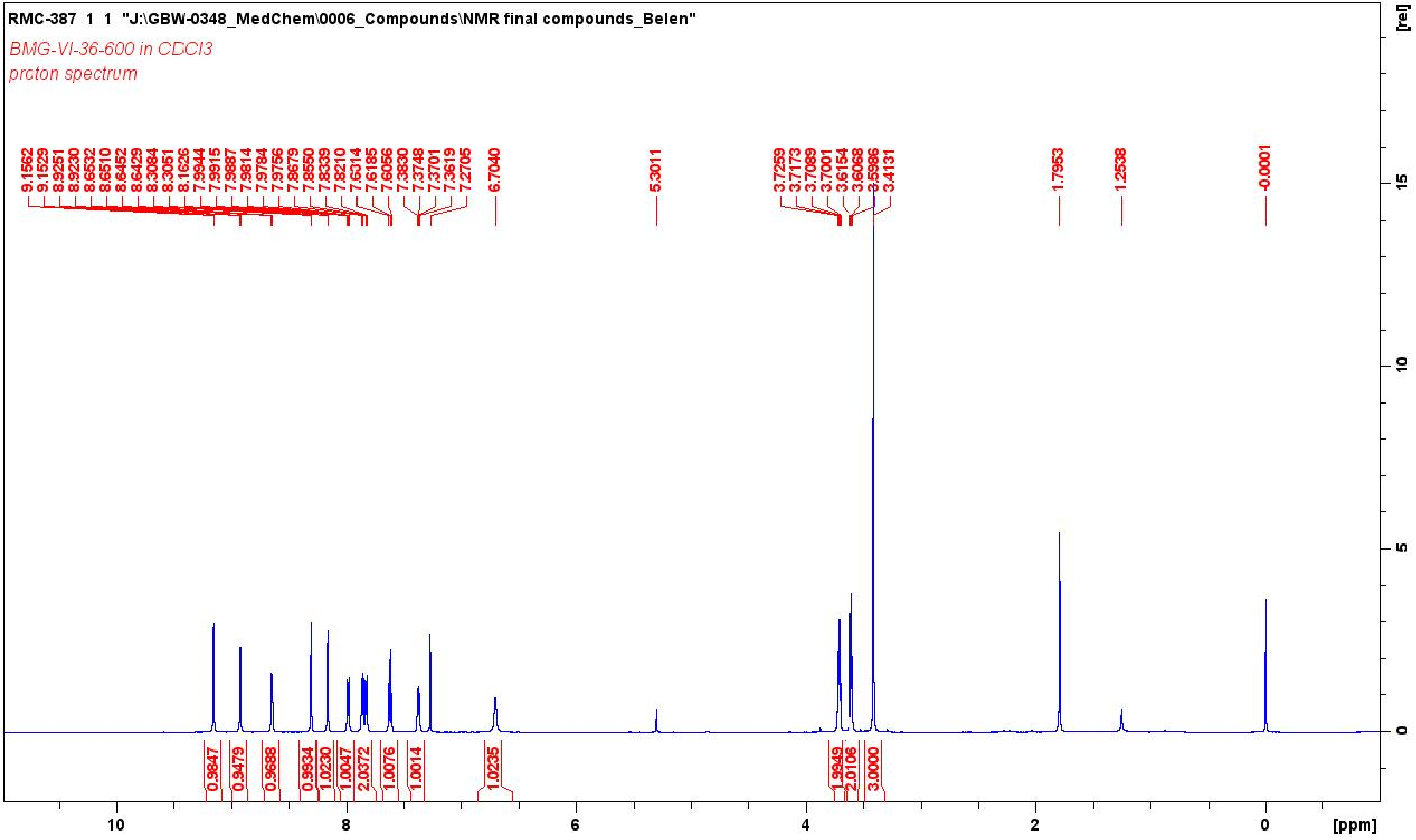

^{13}C NMR spectrum of **3s**

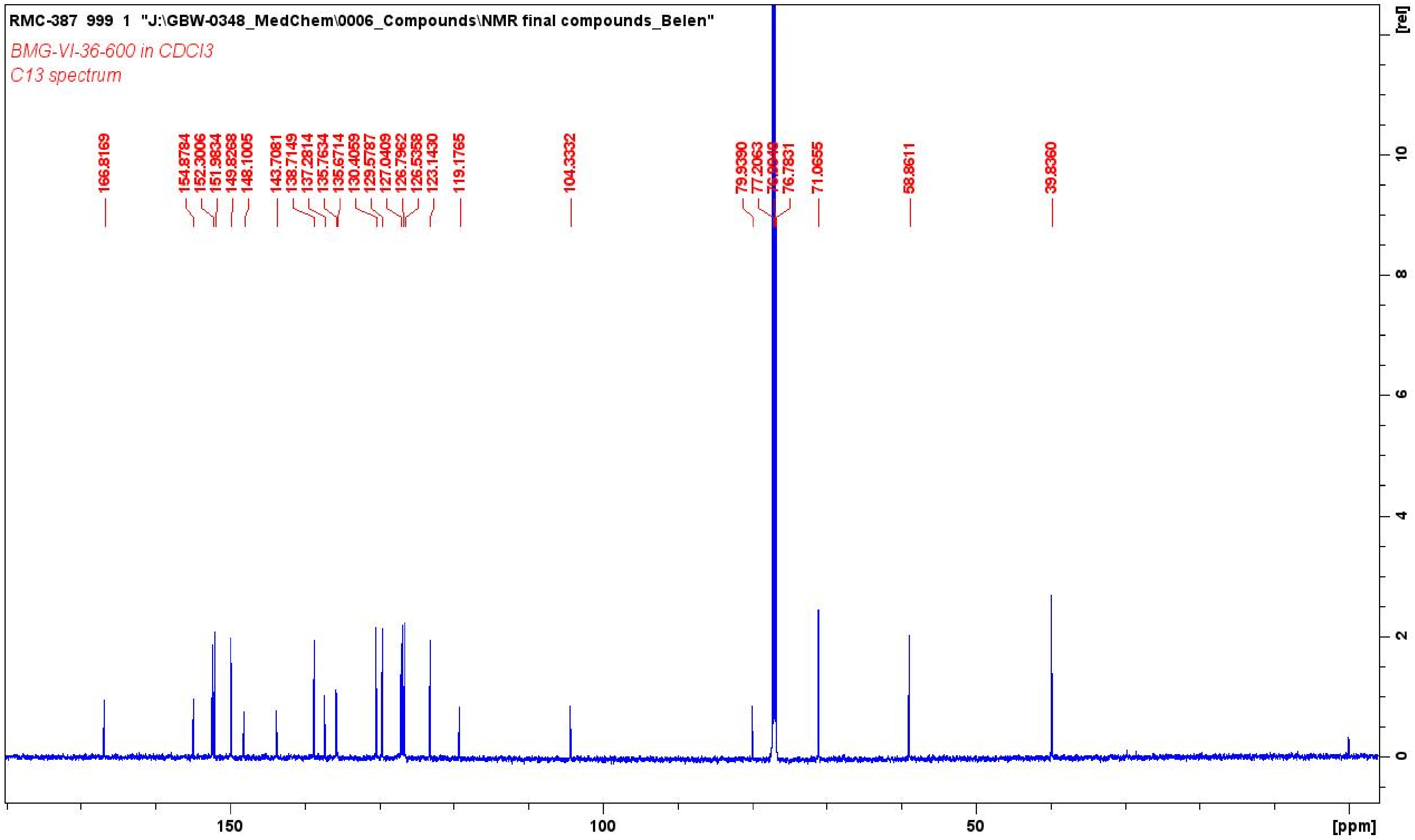

^{1}H NMR spectrum of **4**

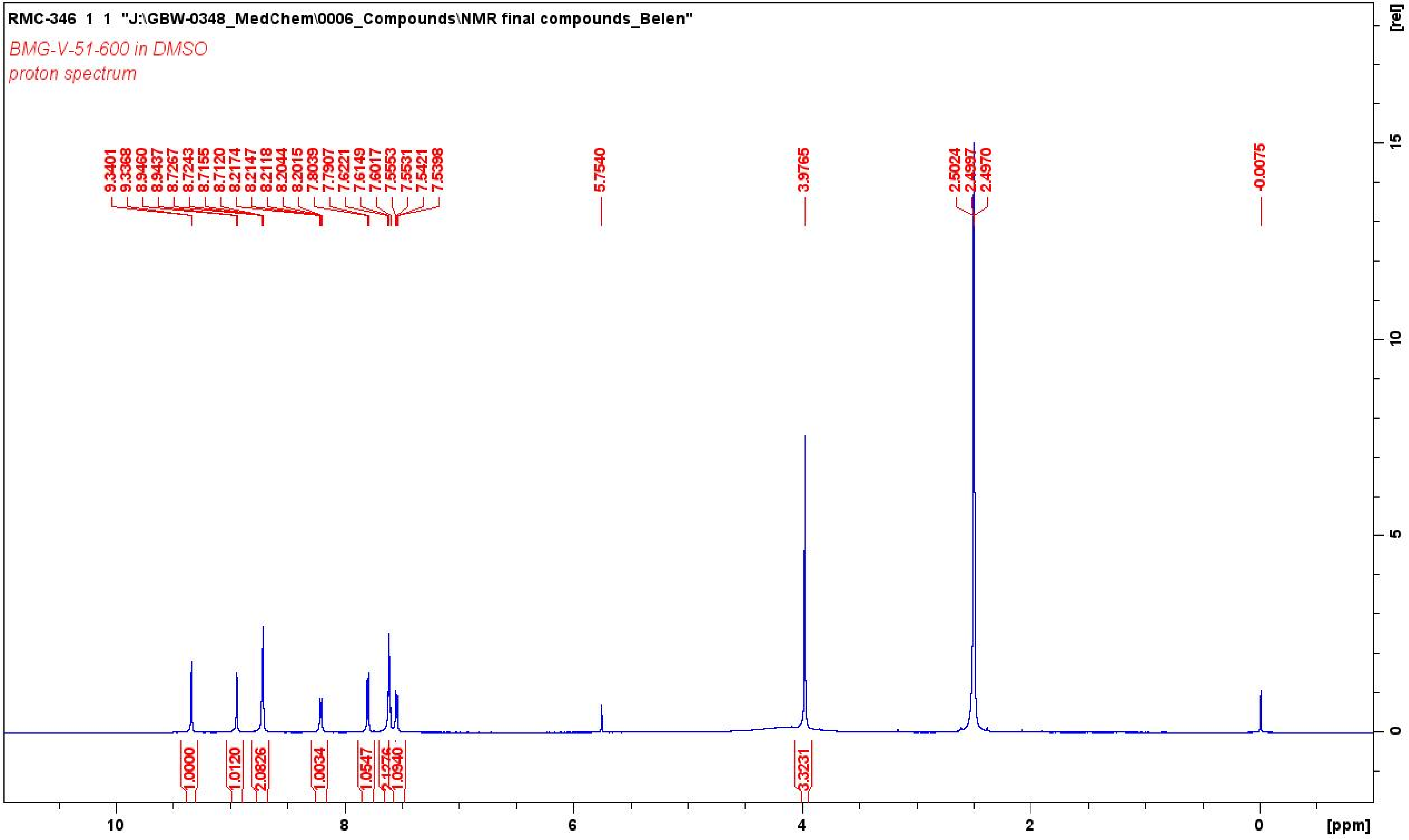

^{13}C NMR spectrum of **4**

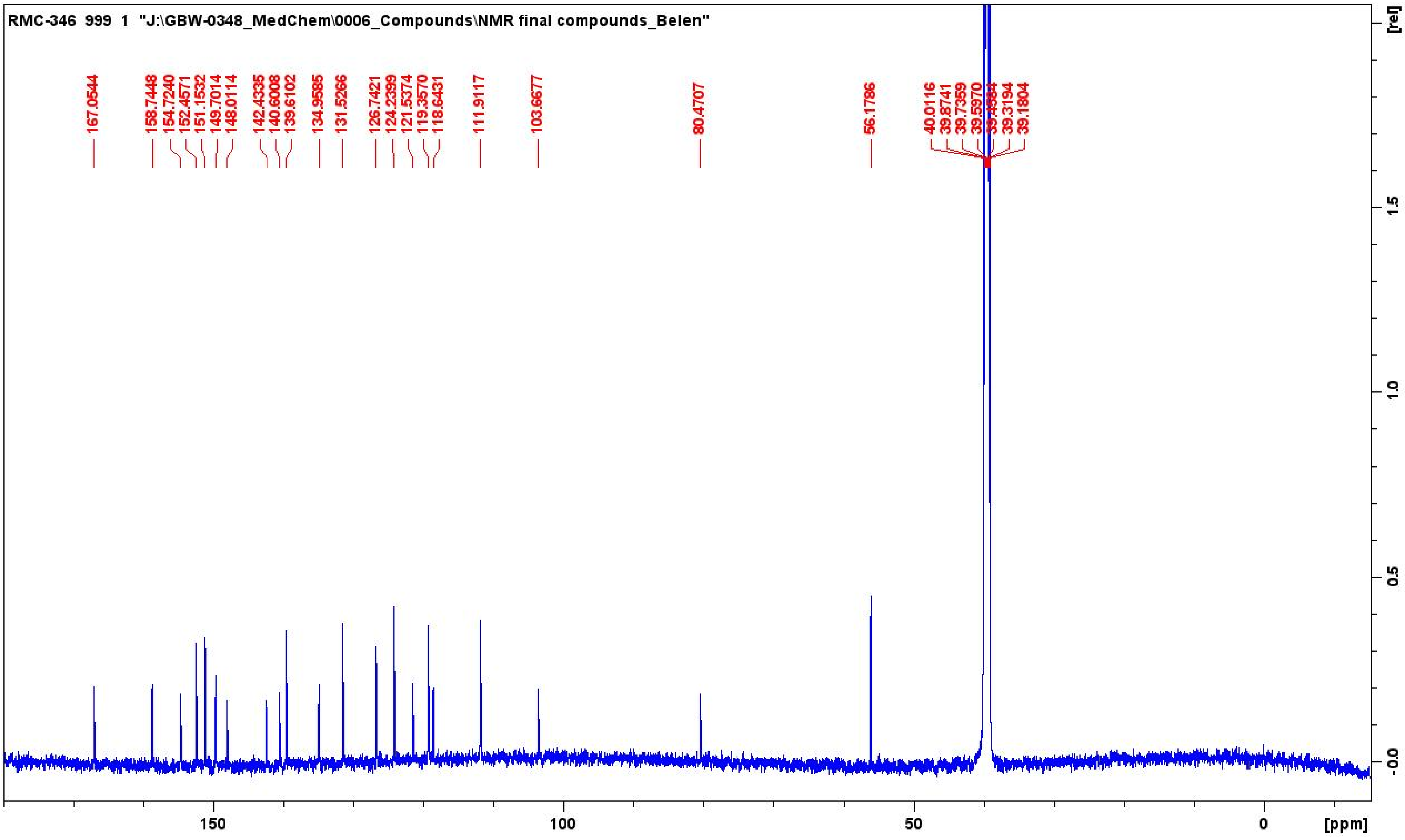

^{1}H NMR spectrum of **5a**

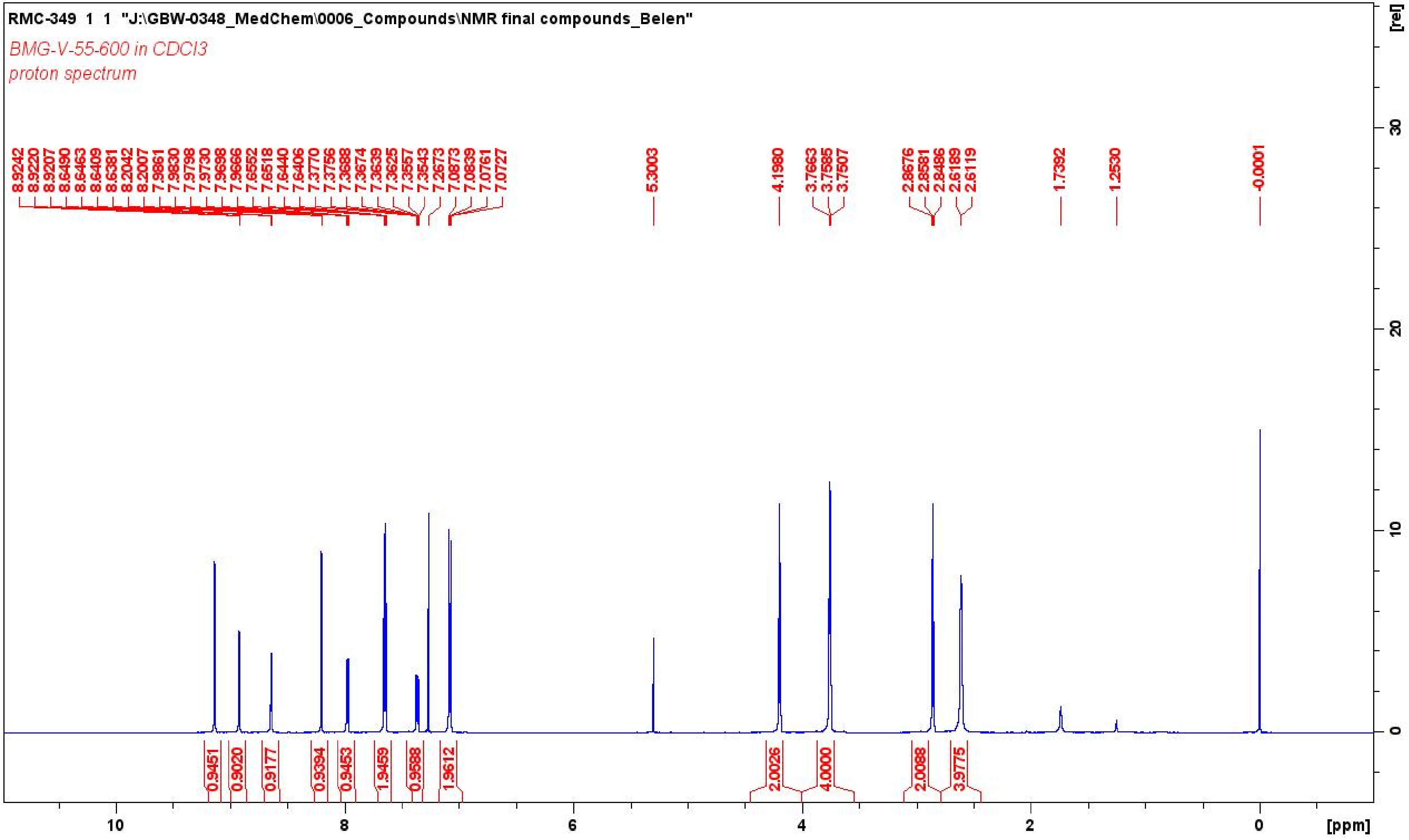

^{13}C NMR spectrum of **5a**

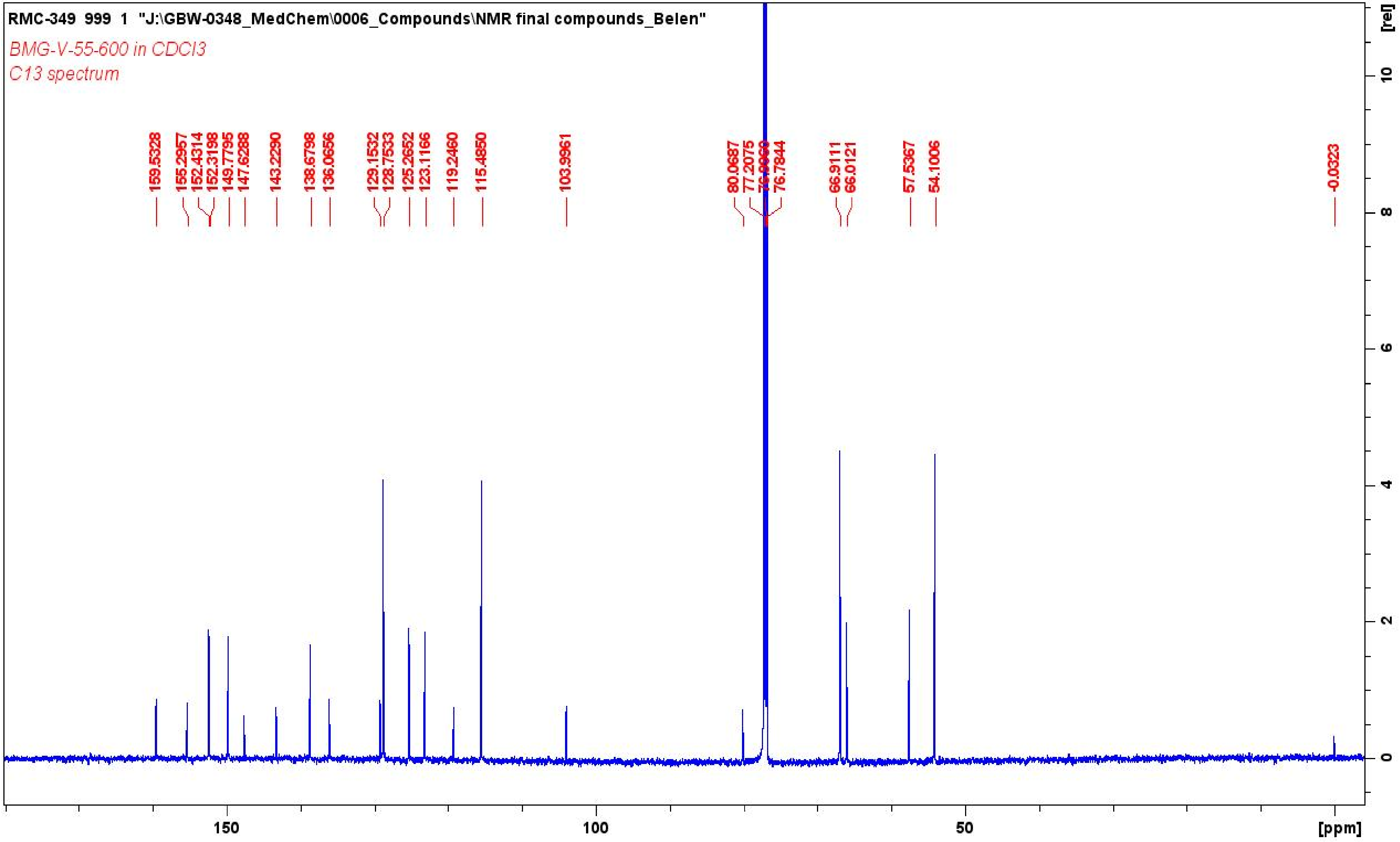

^{1}H NMR spectrum of **5b**

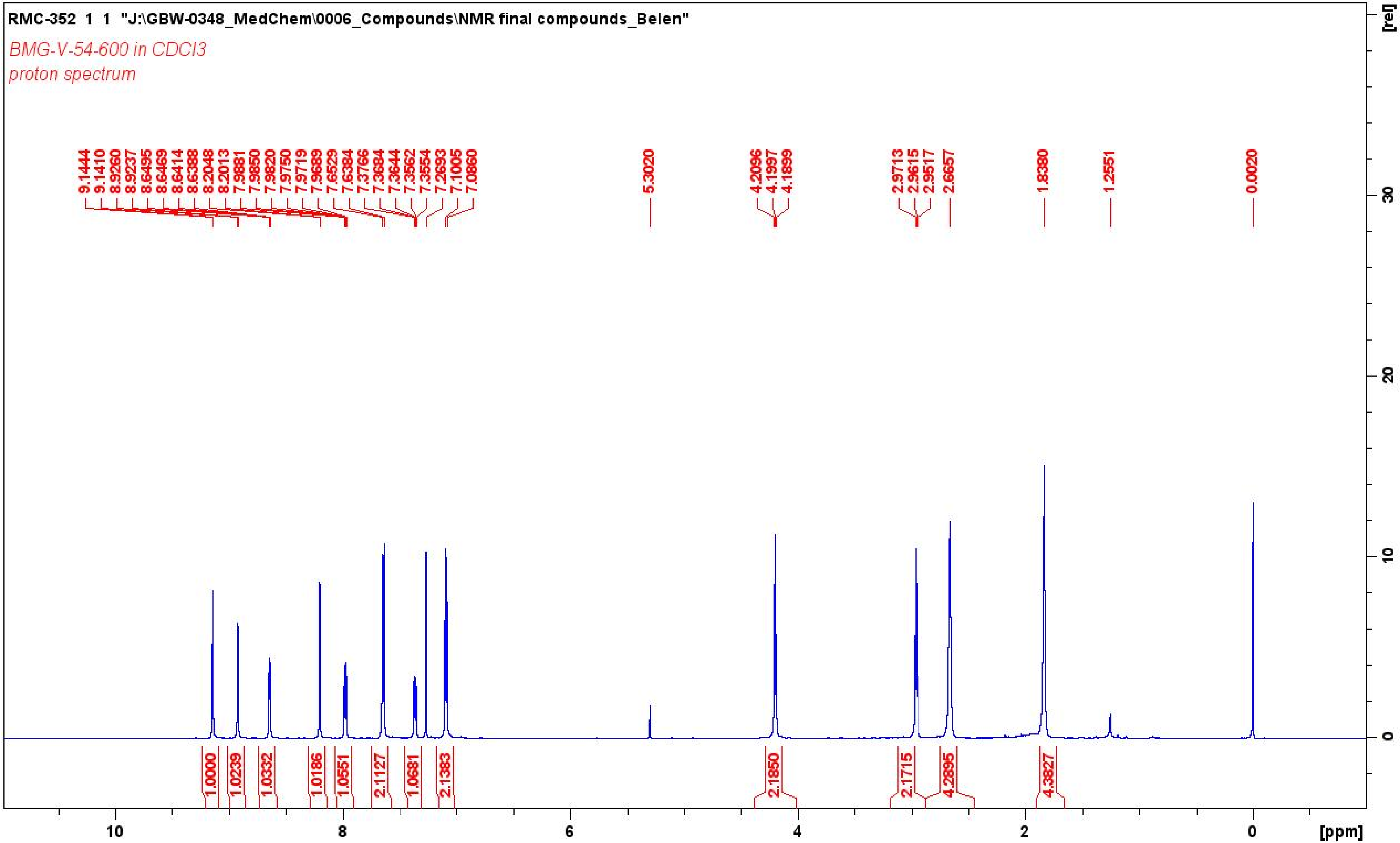

^{13}C NMR spectrum of **5b**

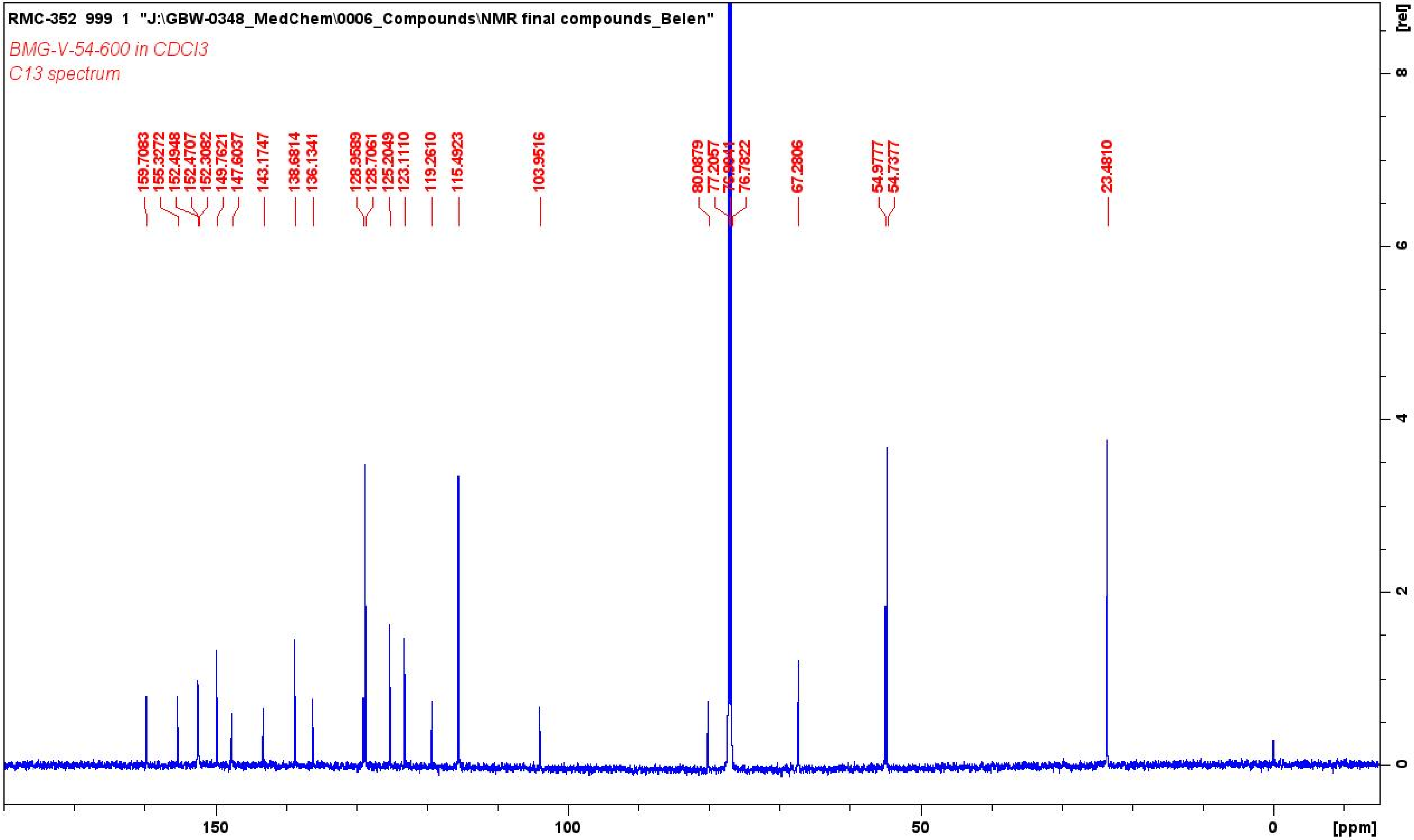

^{1}H NMR spectrum of **7a**

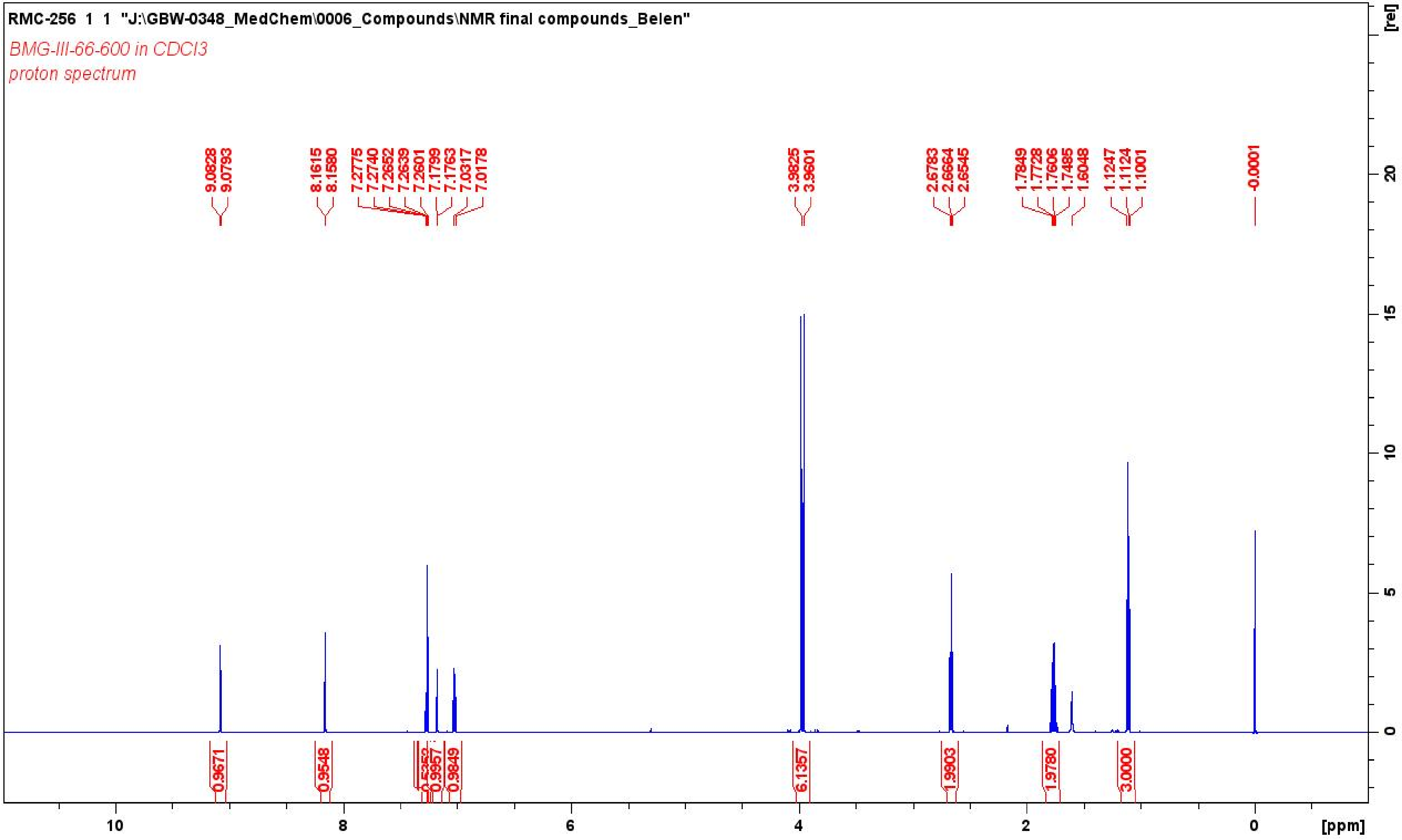

^{13}C NMR spectrum of **7a**

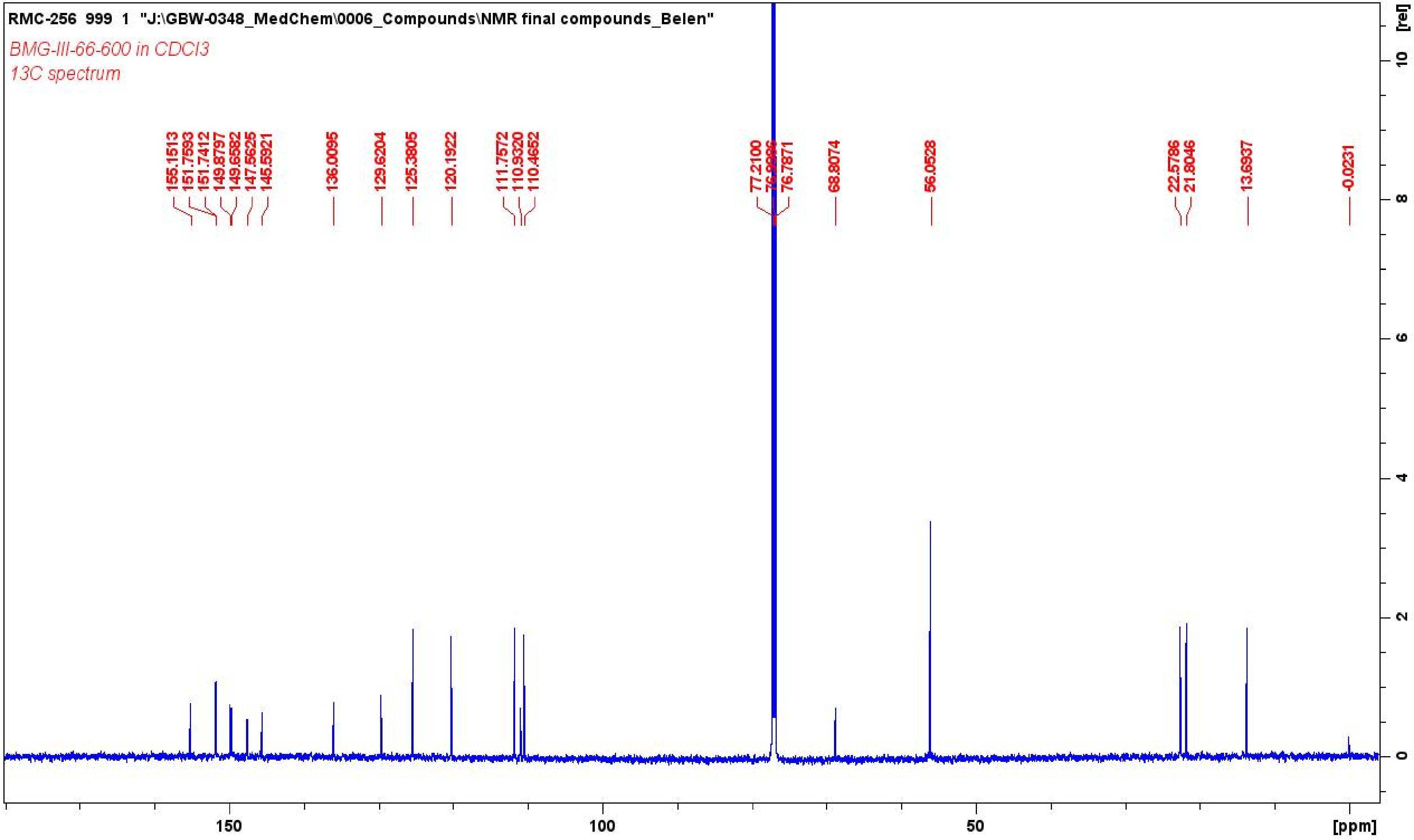

^{1}H NMR spectrum of **7b**

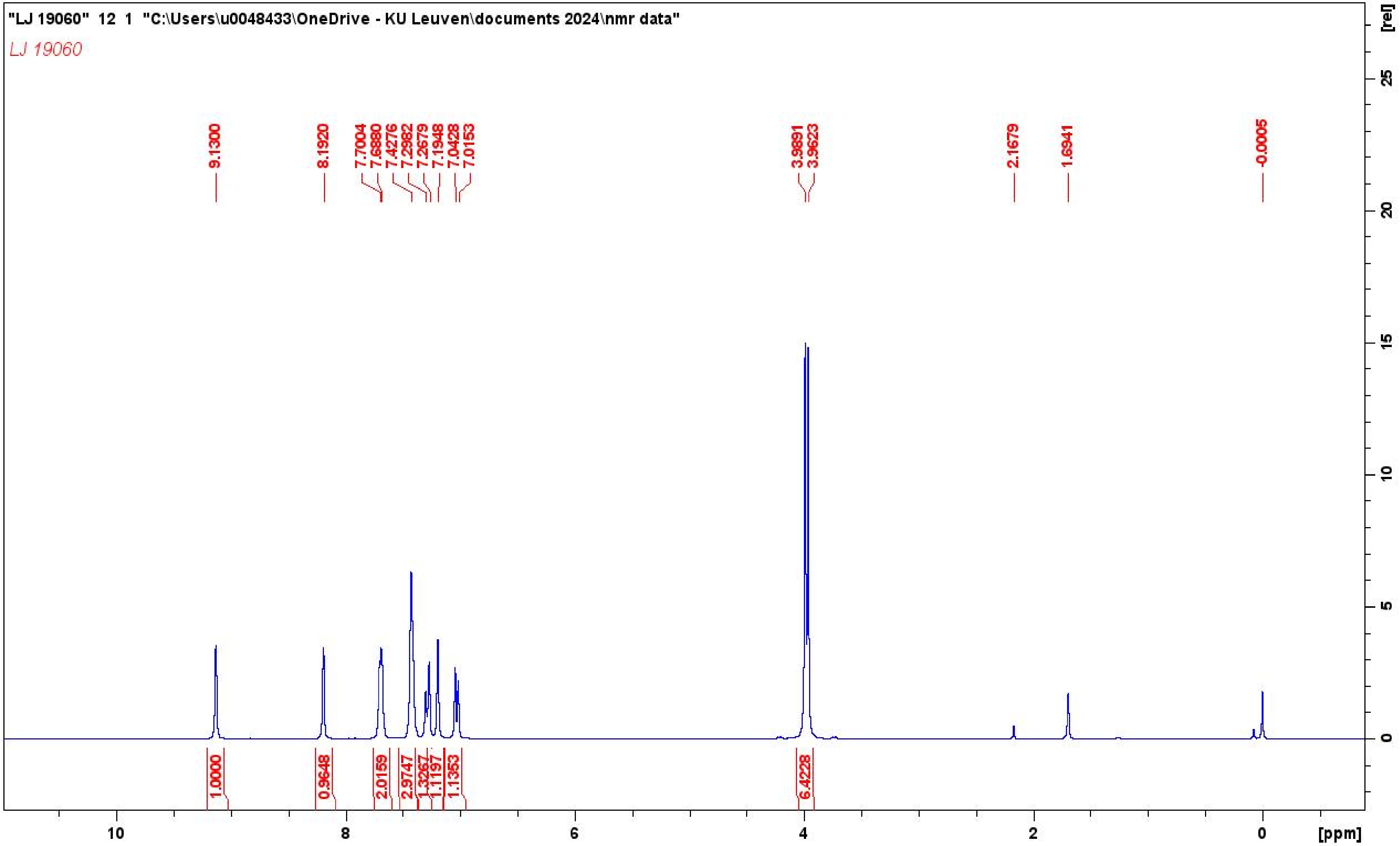

^{13}C NMR spectrum of **7b**

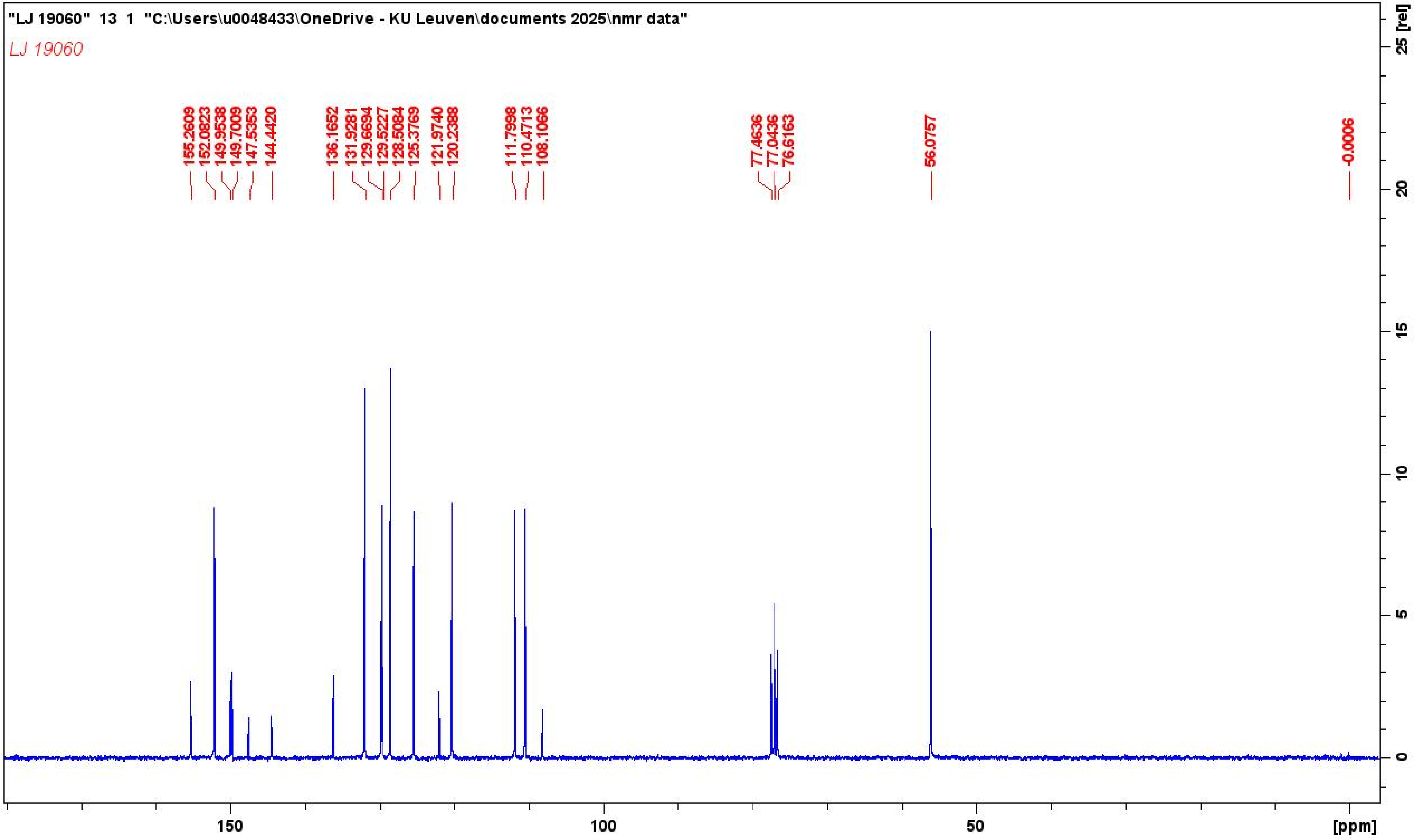

^{1}H NMR spectrum of **7c**

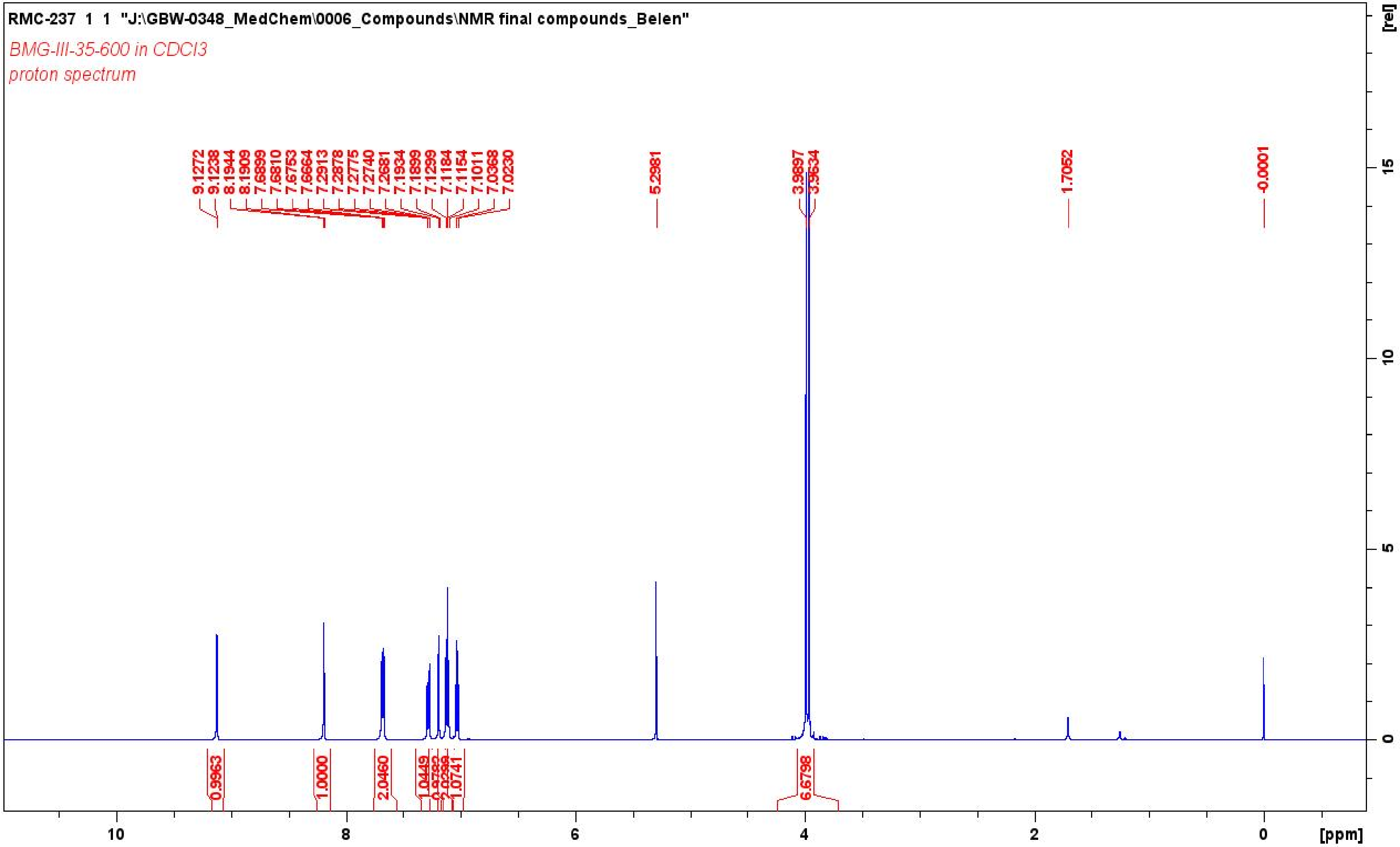

^{13}C NMR spectrum of **7c**

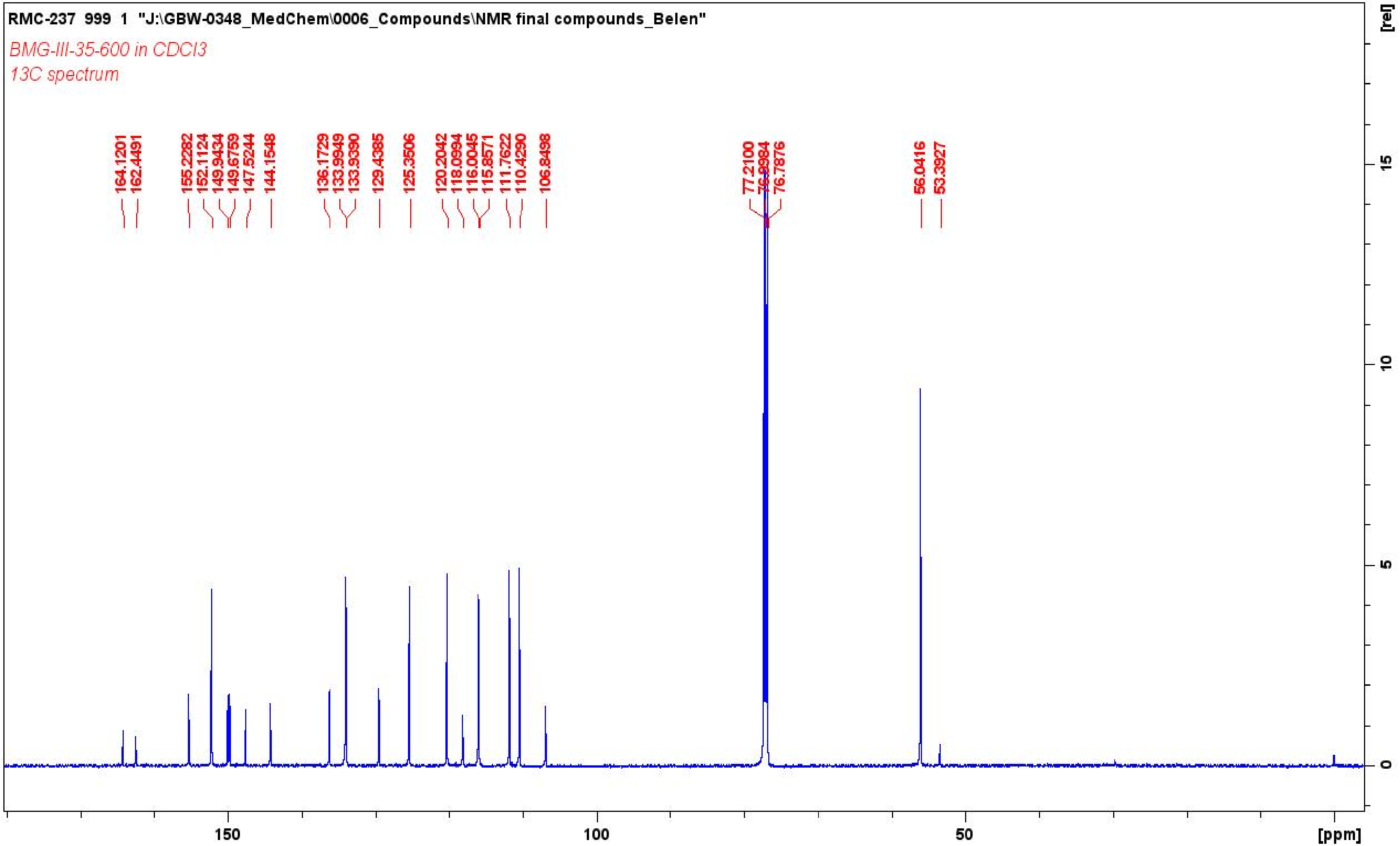

^{1}H NMR spectrum of **7d**

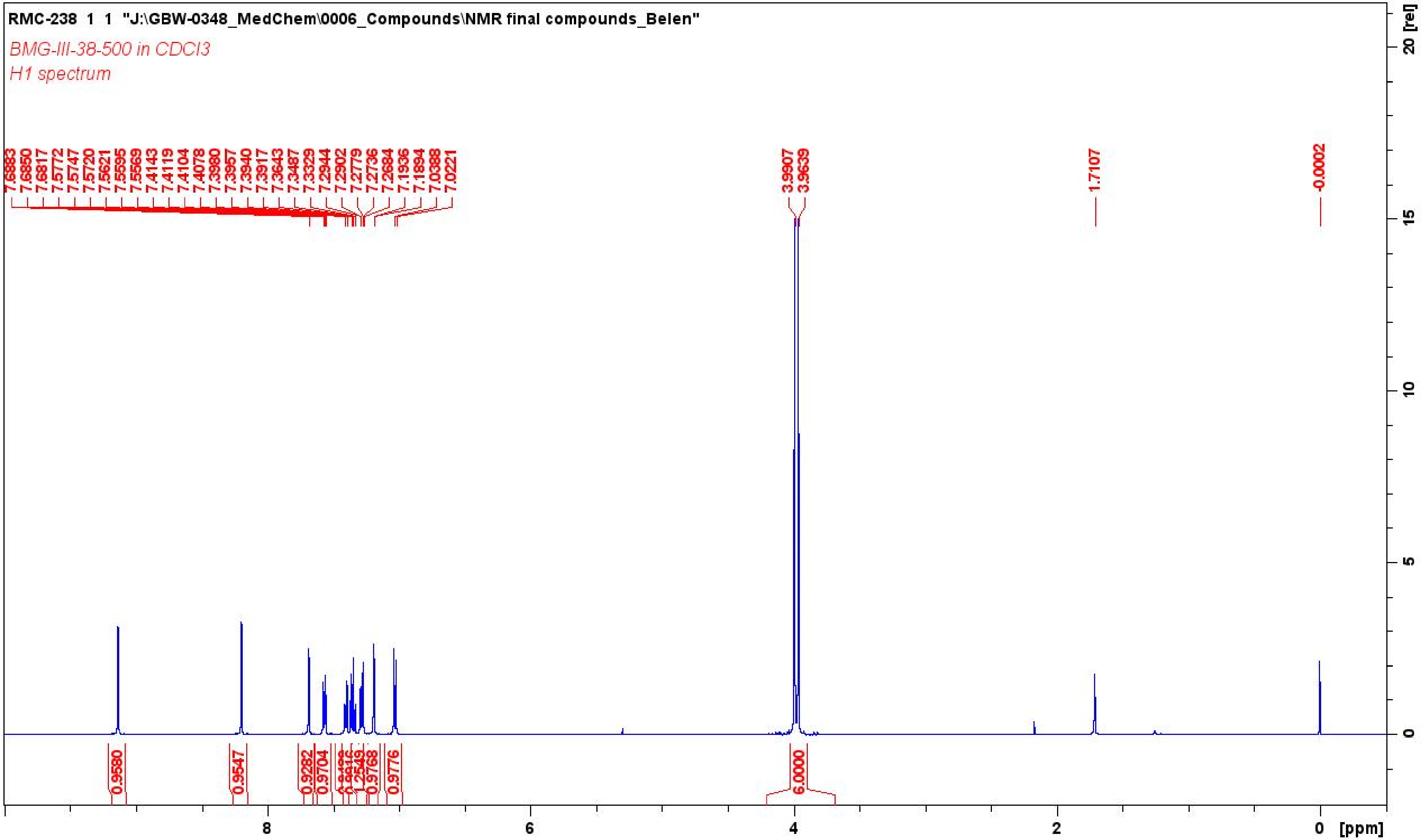

^{13}C NMR spectrum of **7d**

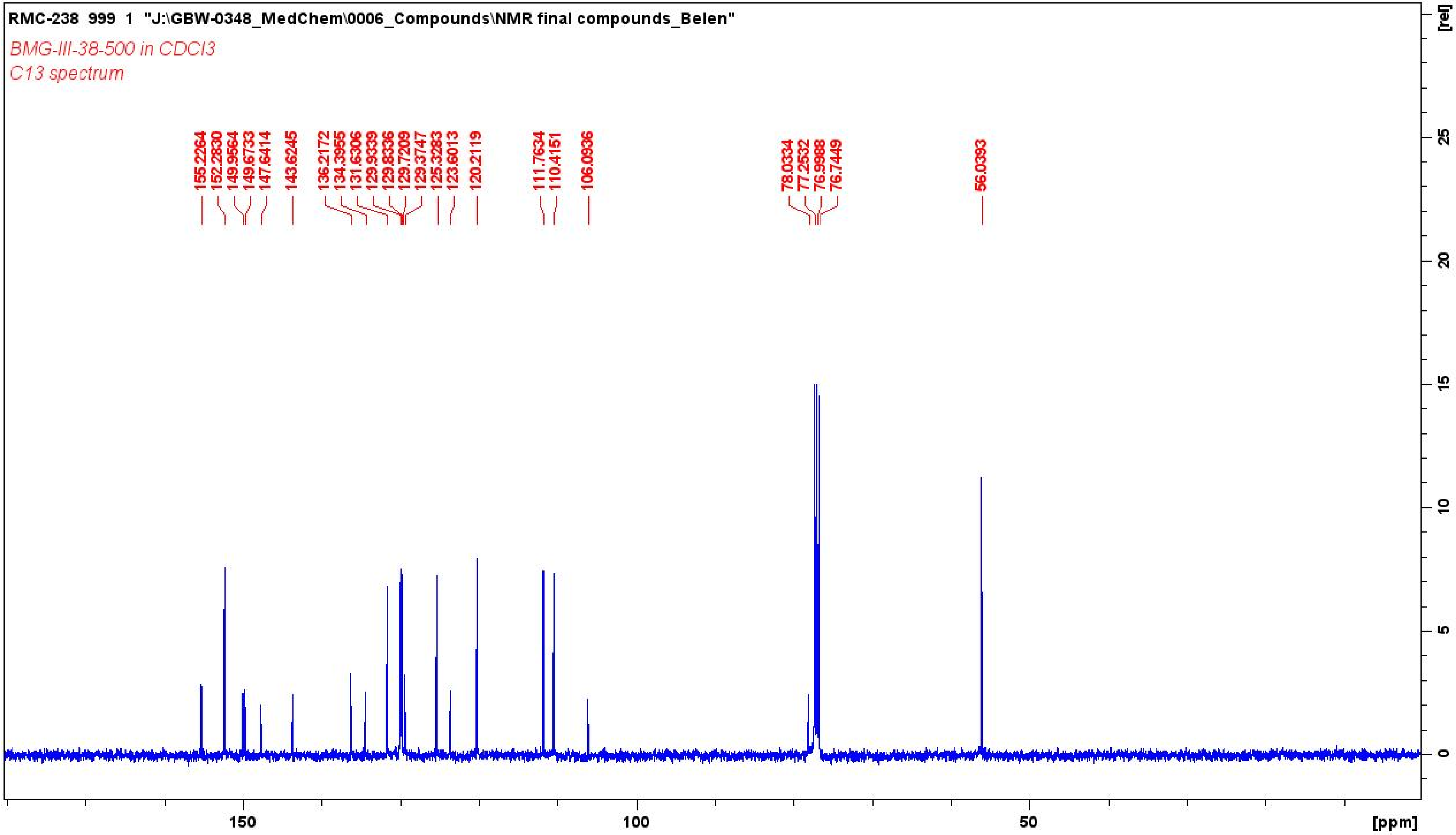

^{1}H NMR spectrum of **7e**

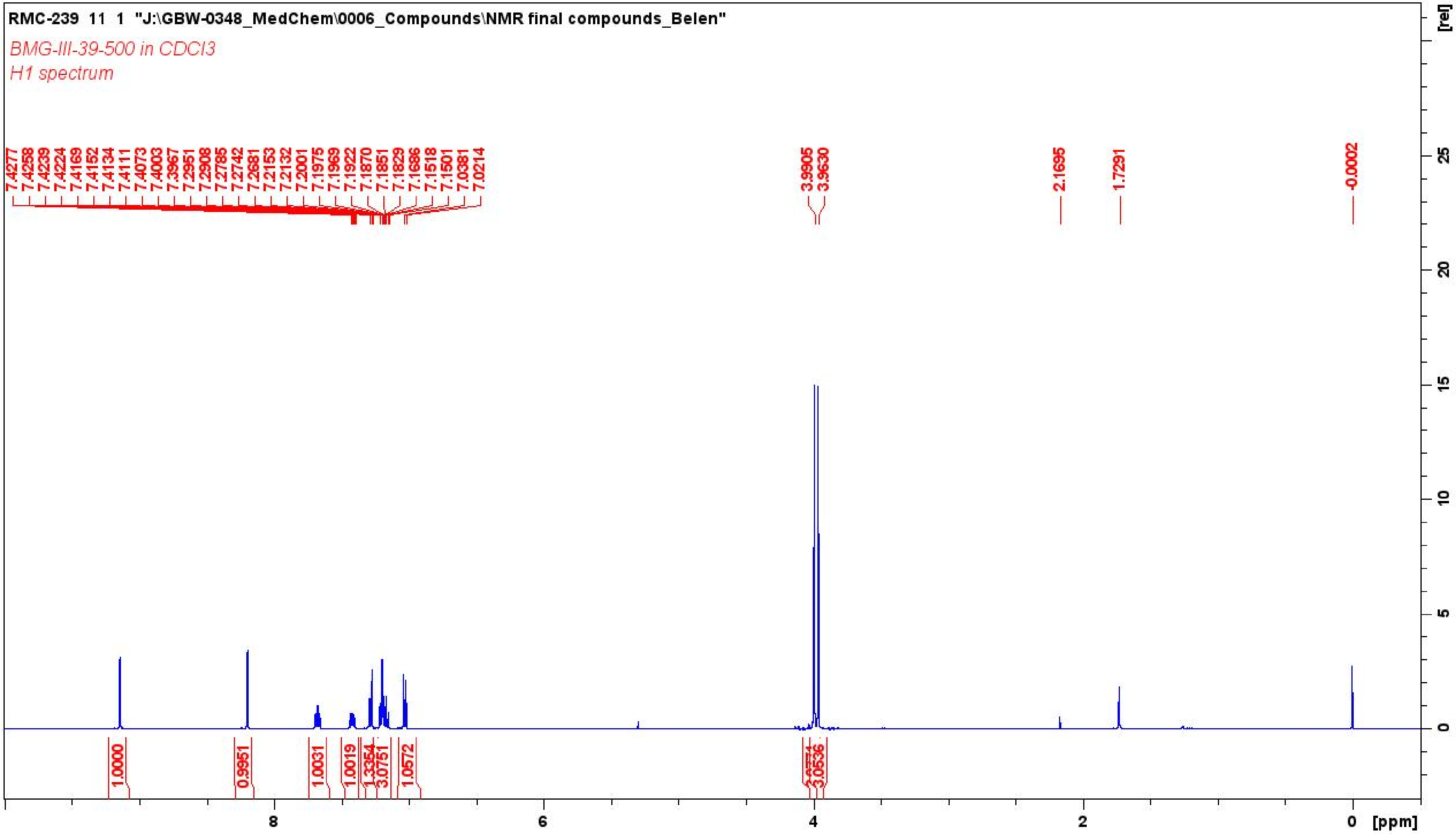

^{13}C NMR spectrum of **7e**

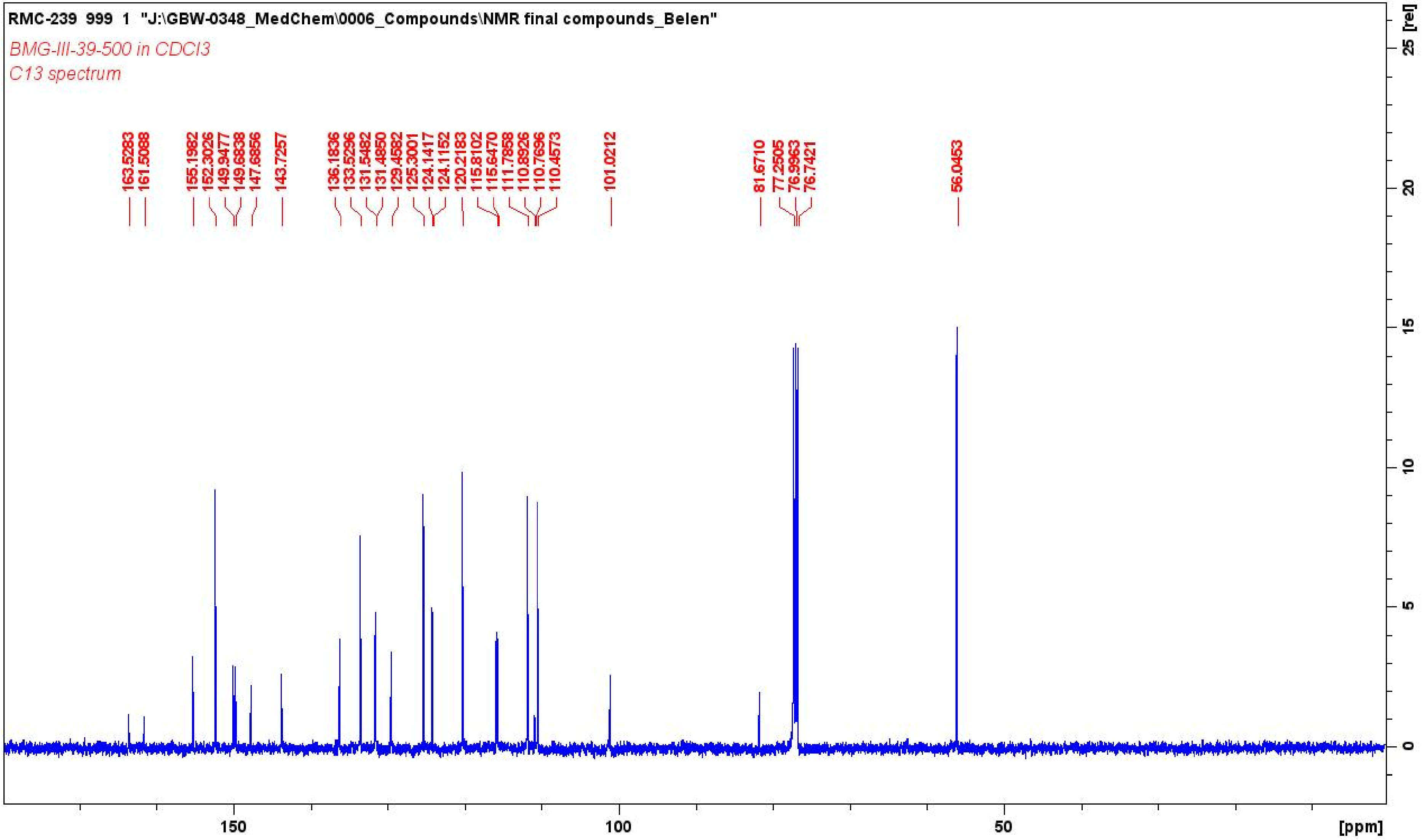

^{1}H NMR spectrum of **7f**

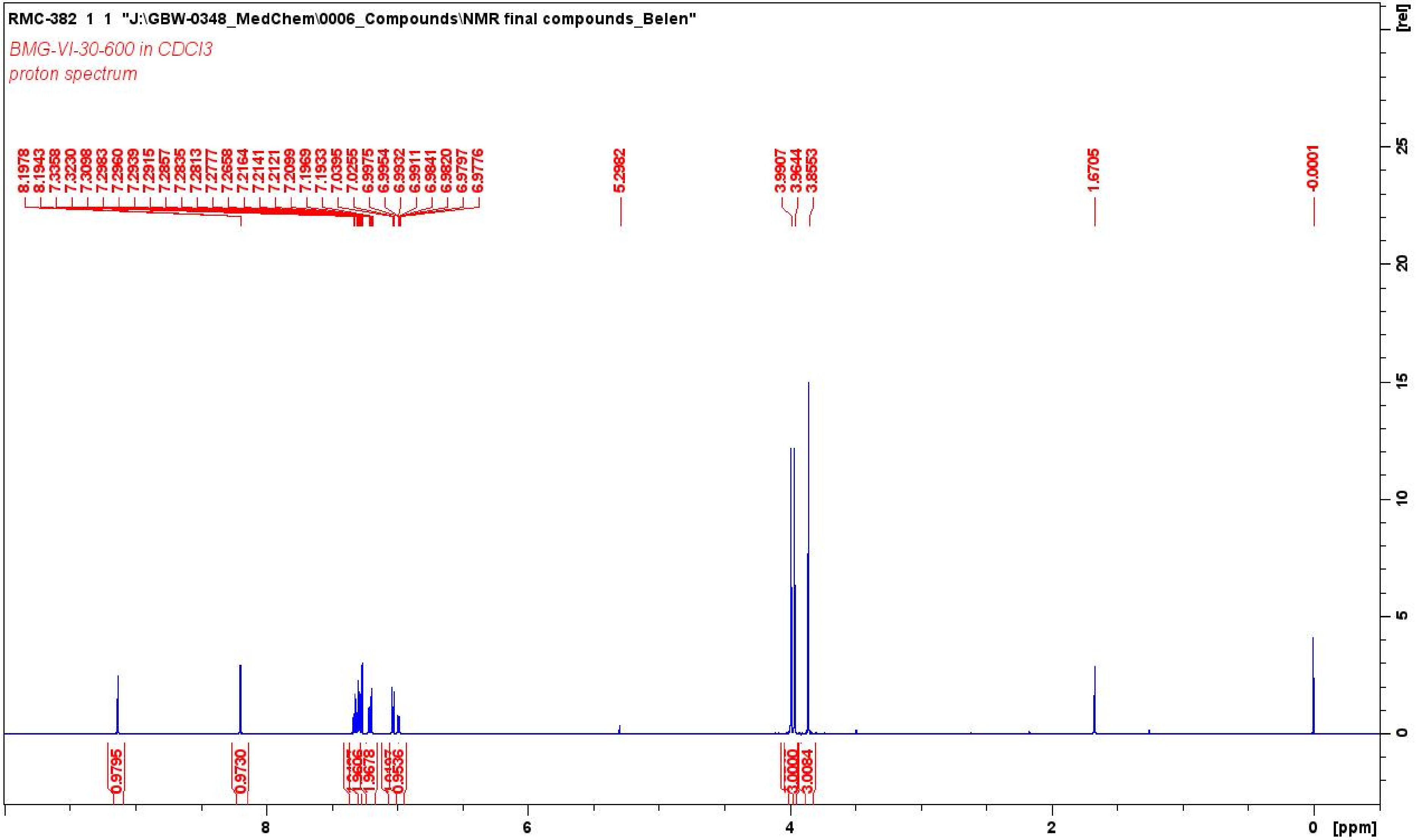

^{13}C NMR spectrum of **7f**

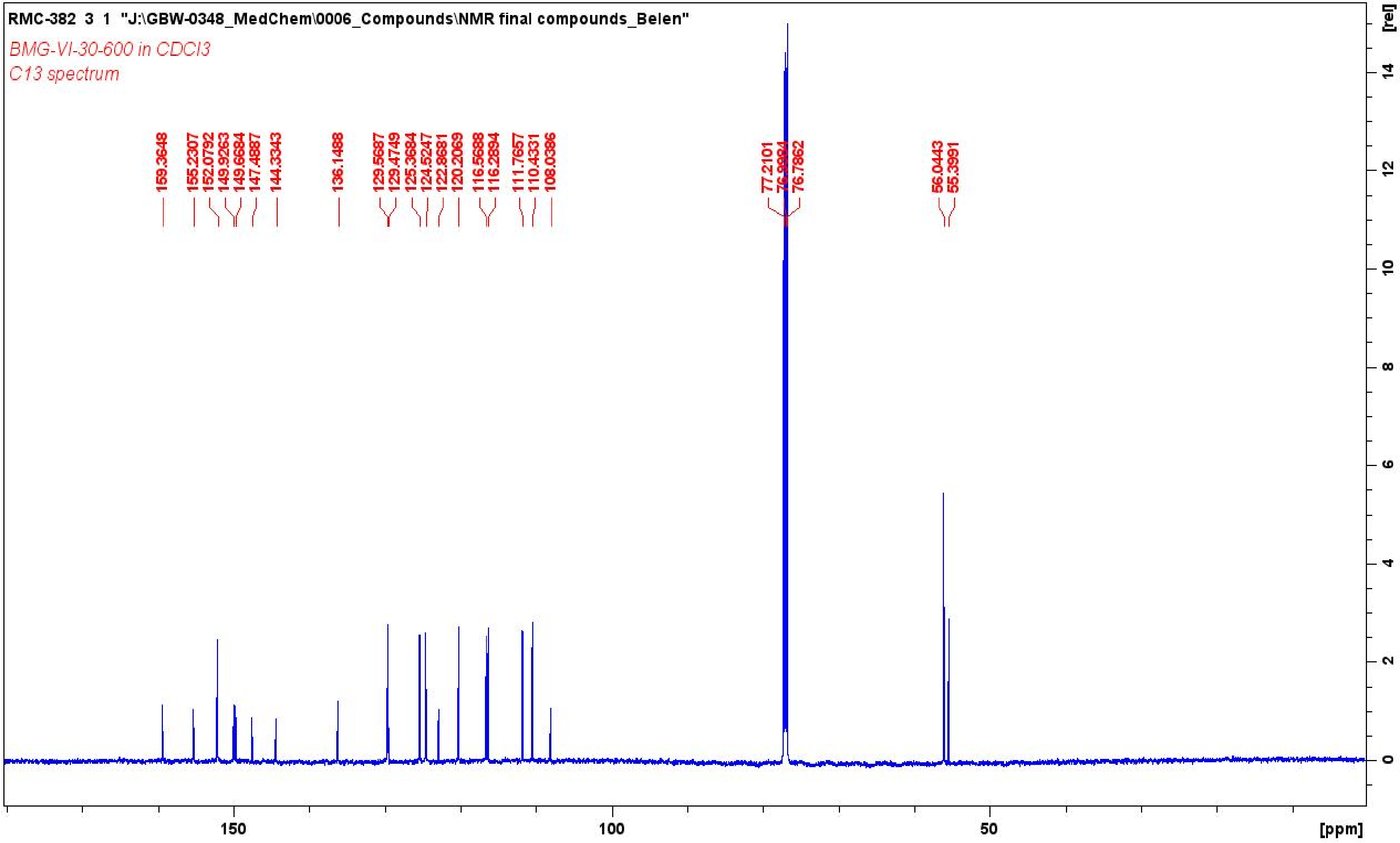

^{1}H NMR spectrum of **7g**

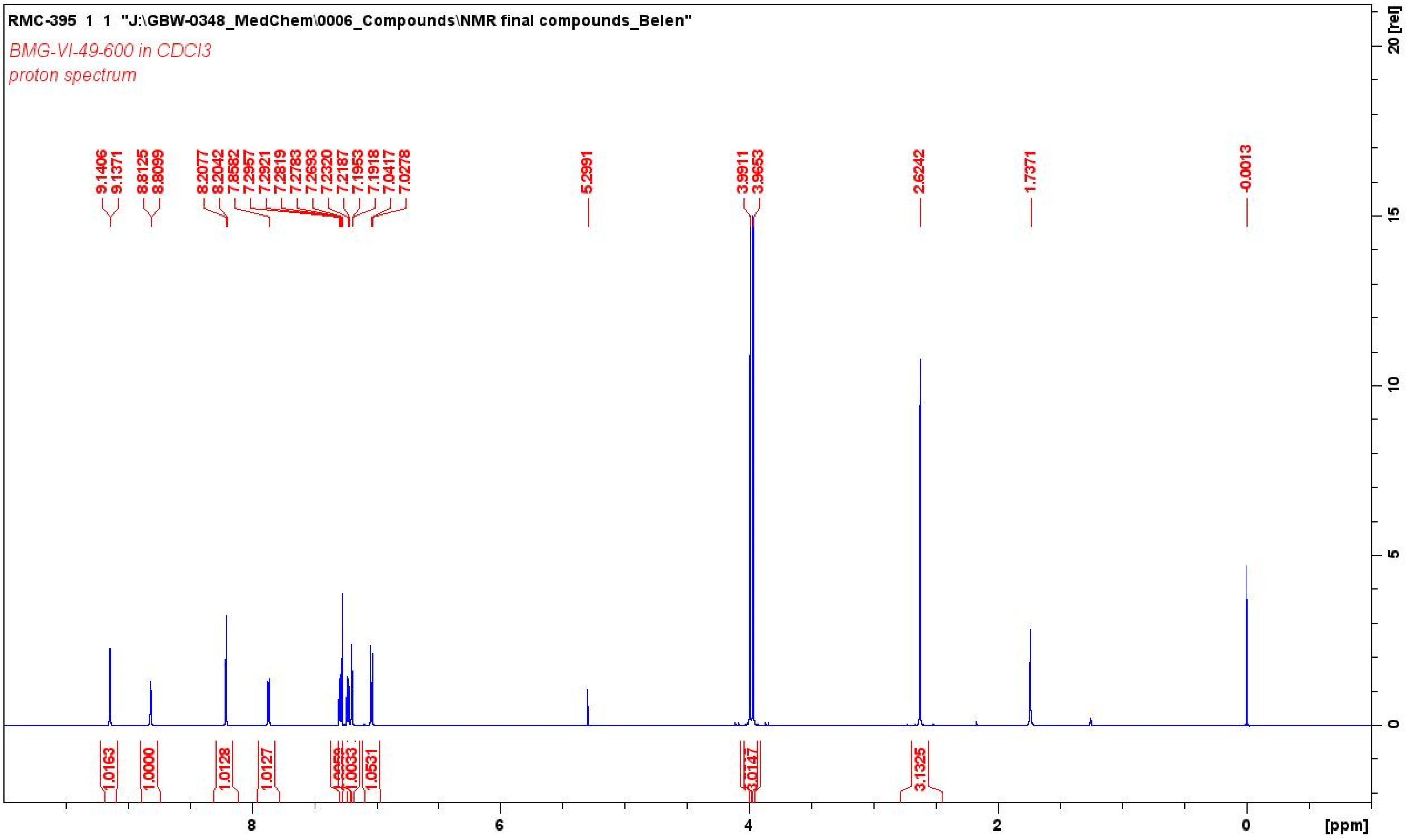

^{13}C NMR spectrum of **7g**

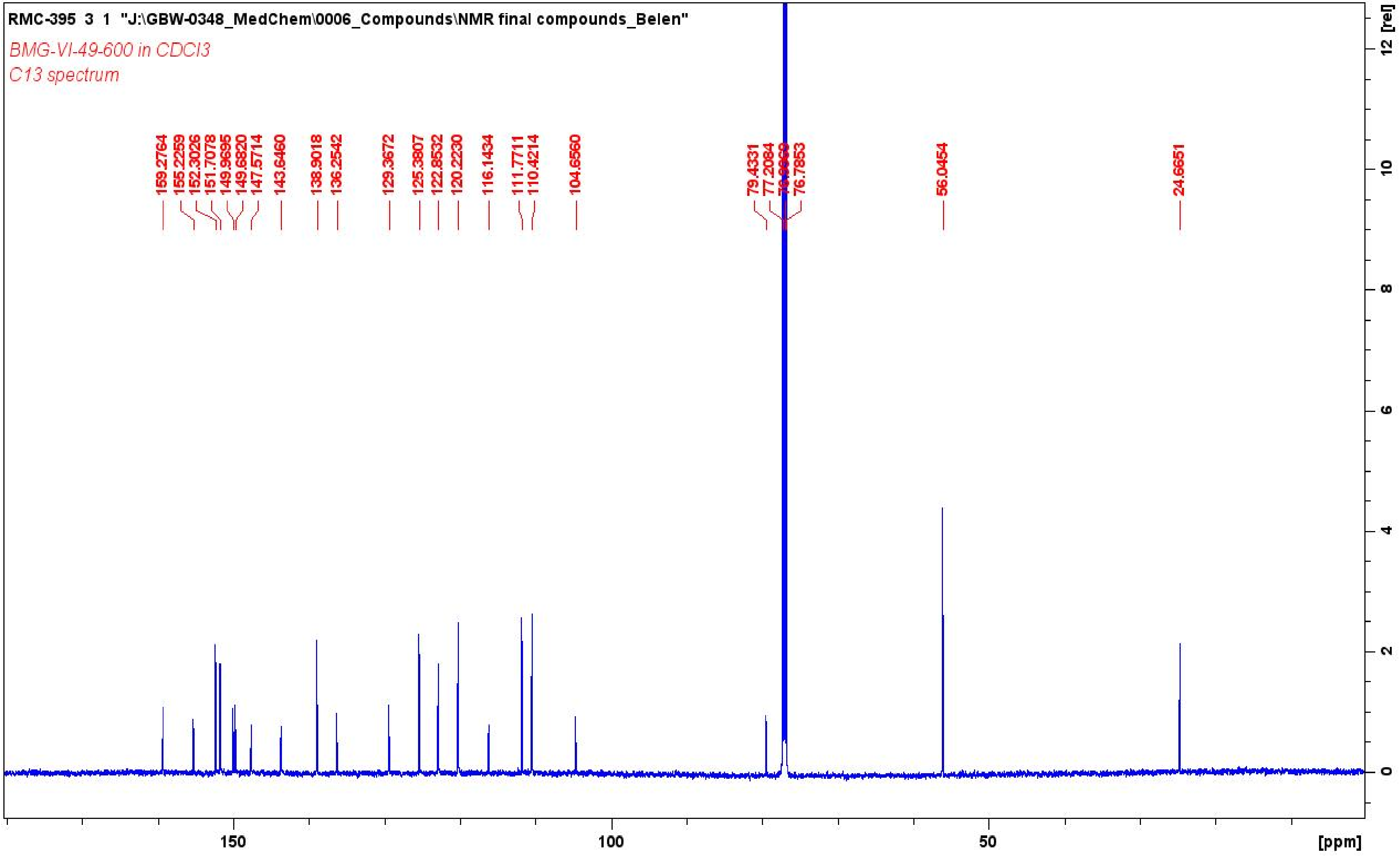

^{1}H NMR spectrum of **7h**

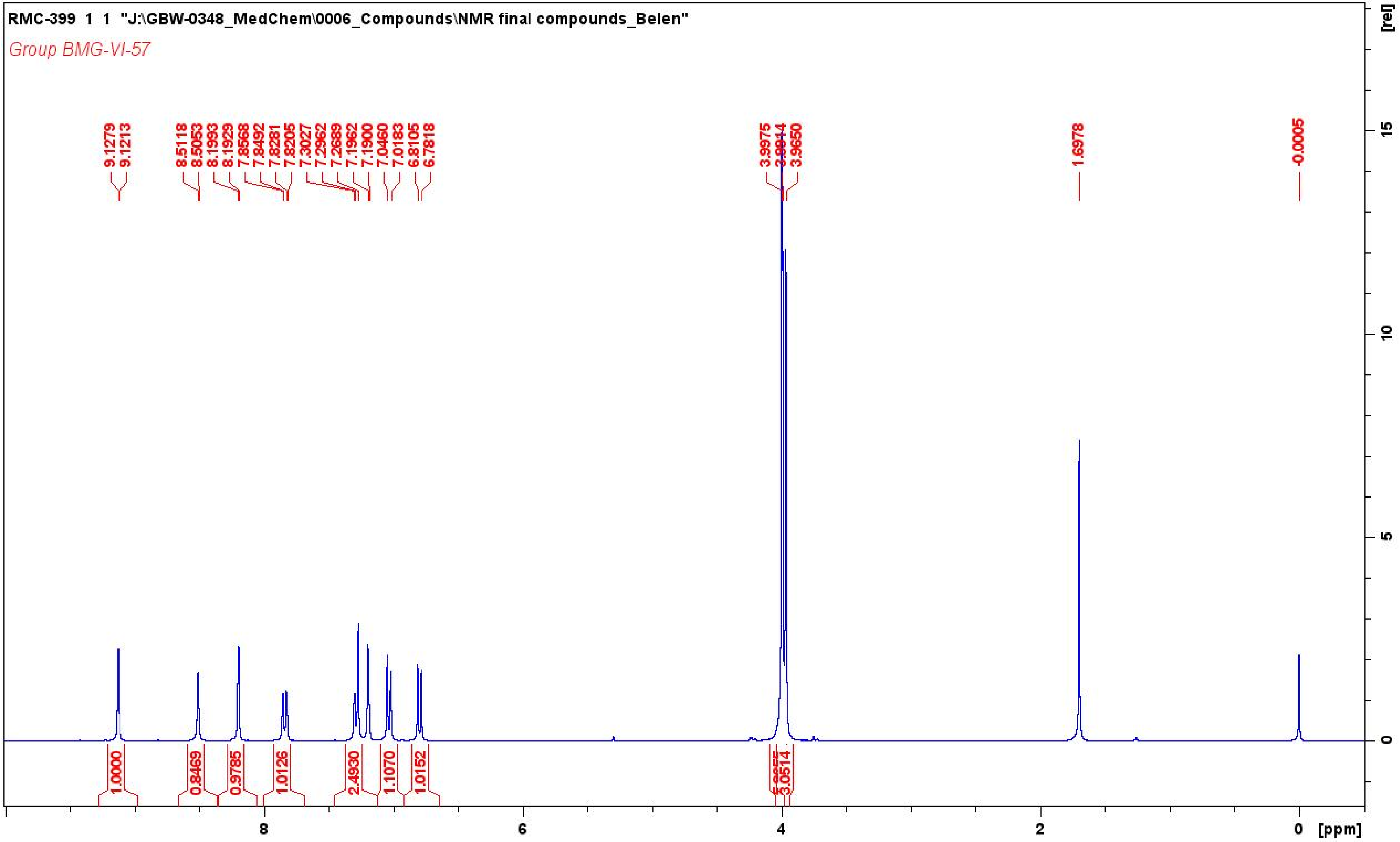

^{13}C NMR spectrum of **7h**

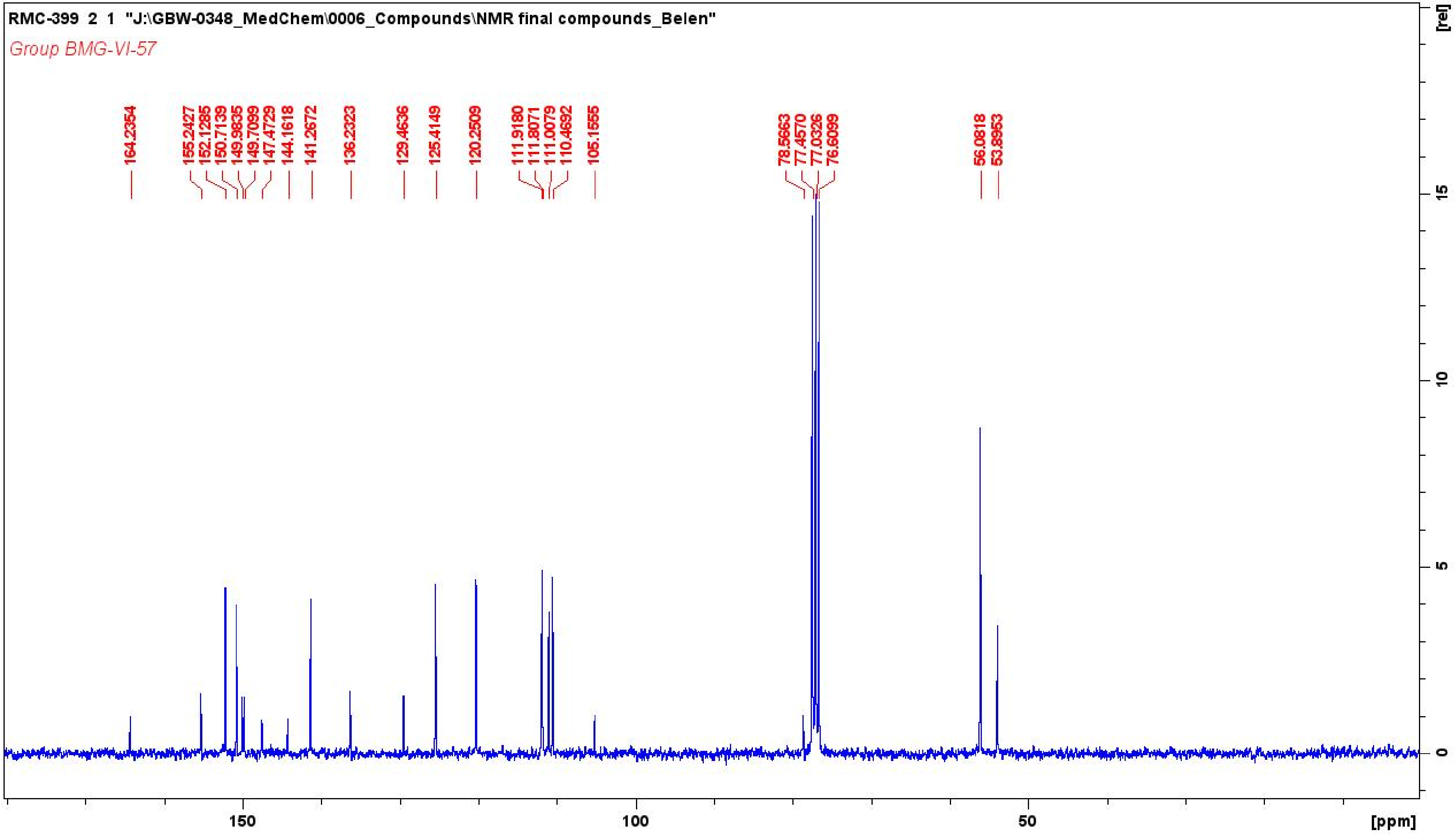

^{1}H NMR spectrum of **7i**

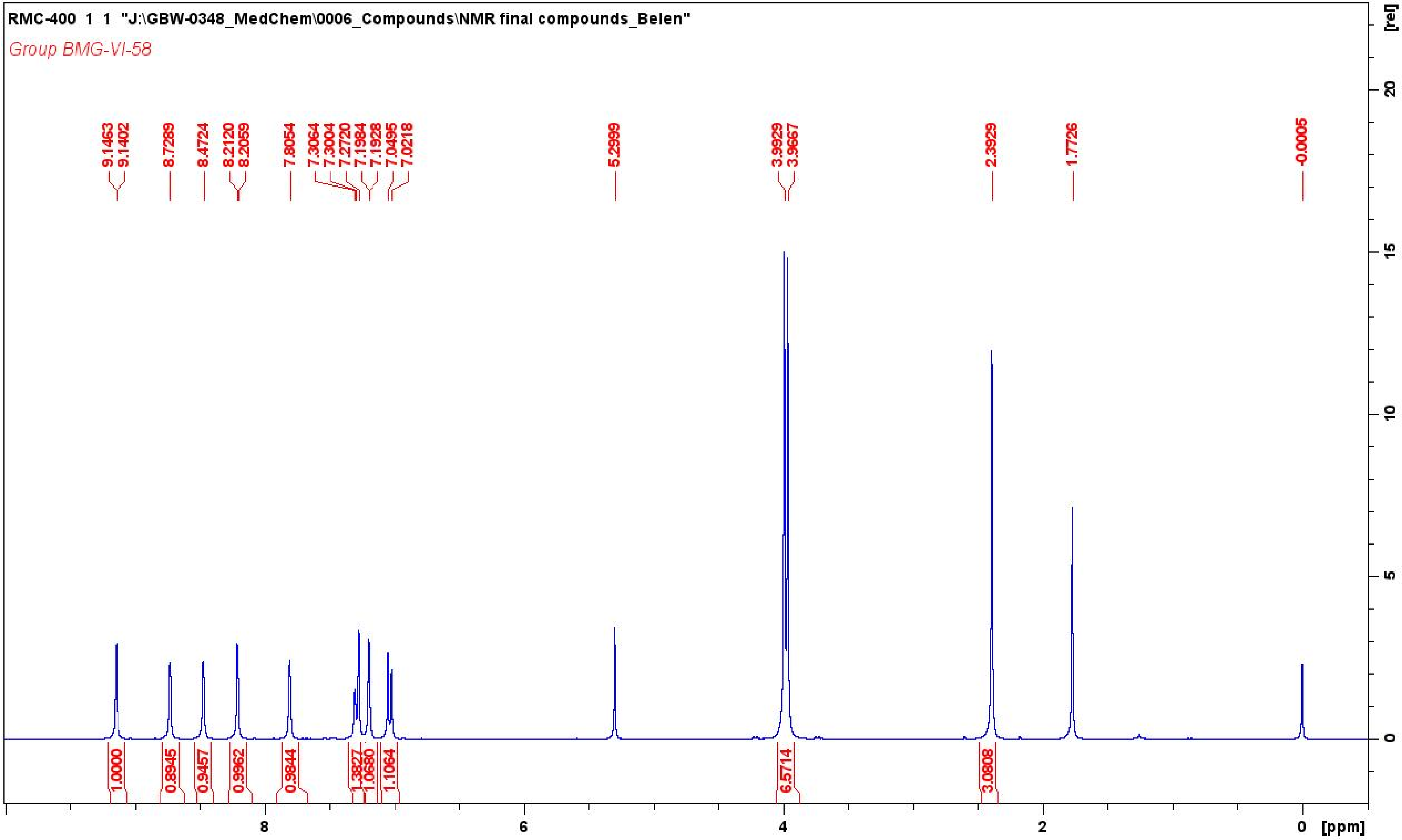

^{13}C NMR spectrum of **7i**

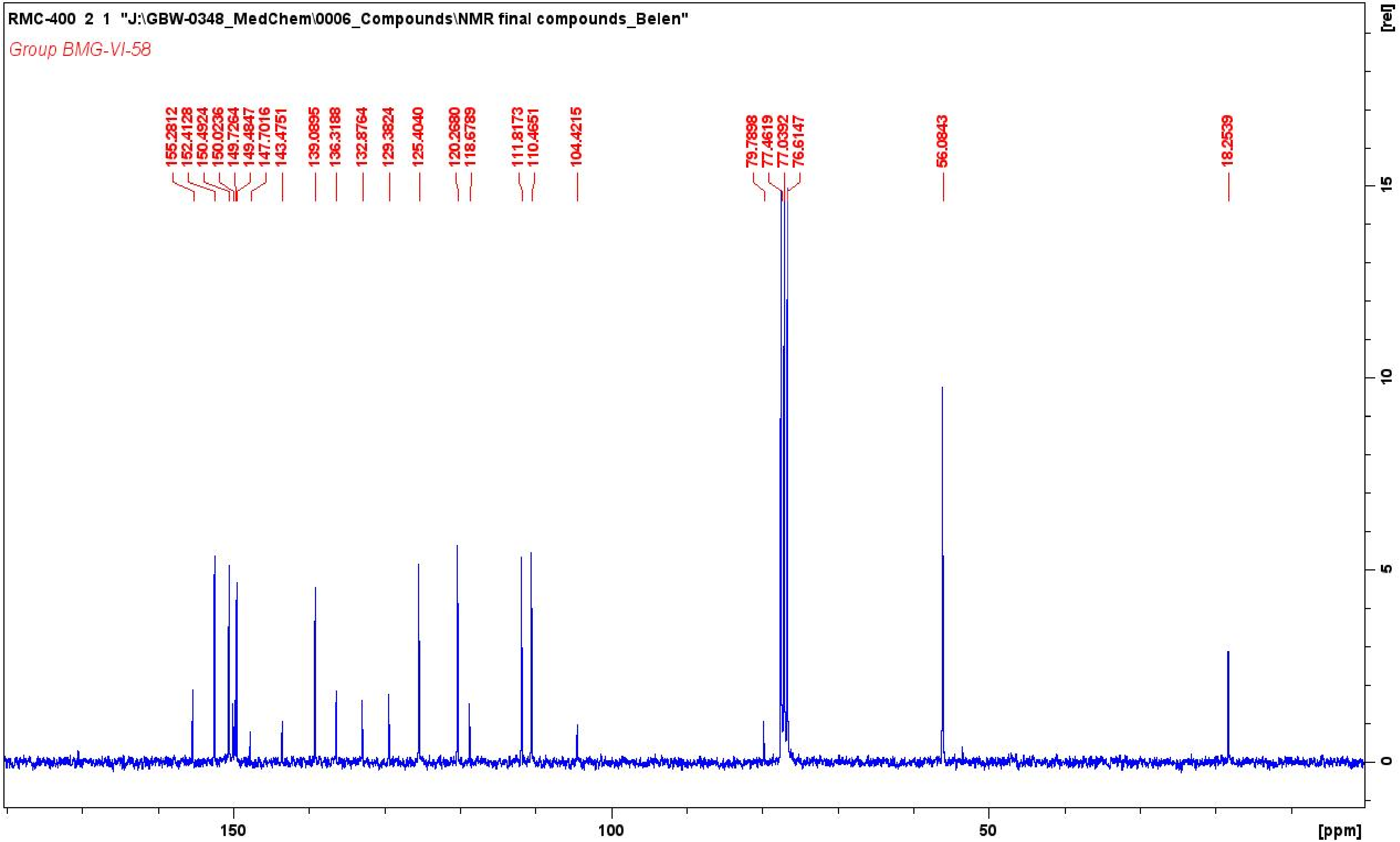

^{1}H NMR spectrum of **7j**

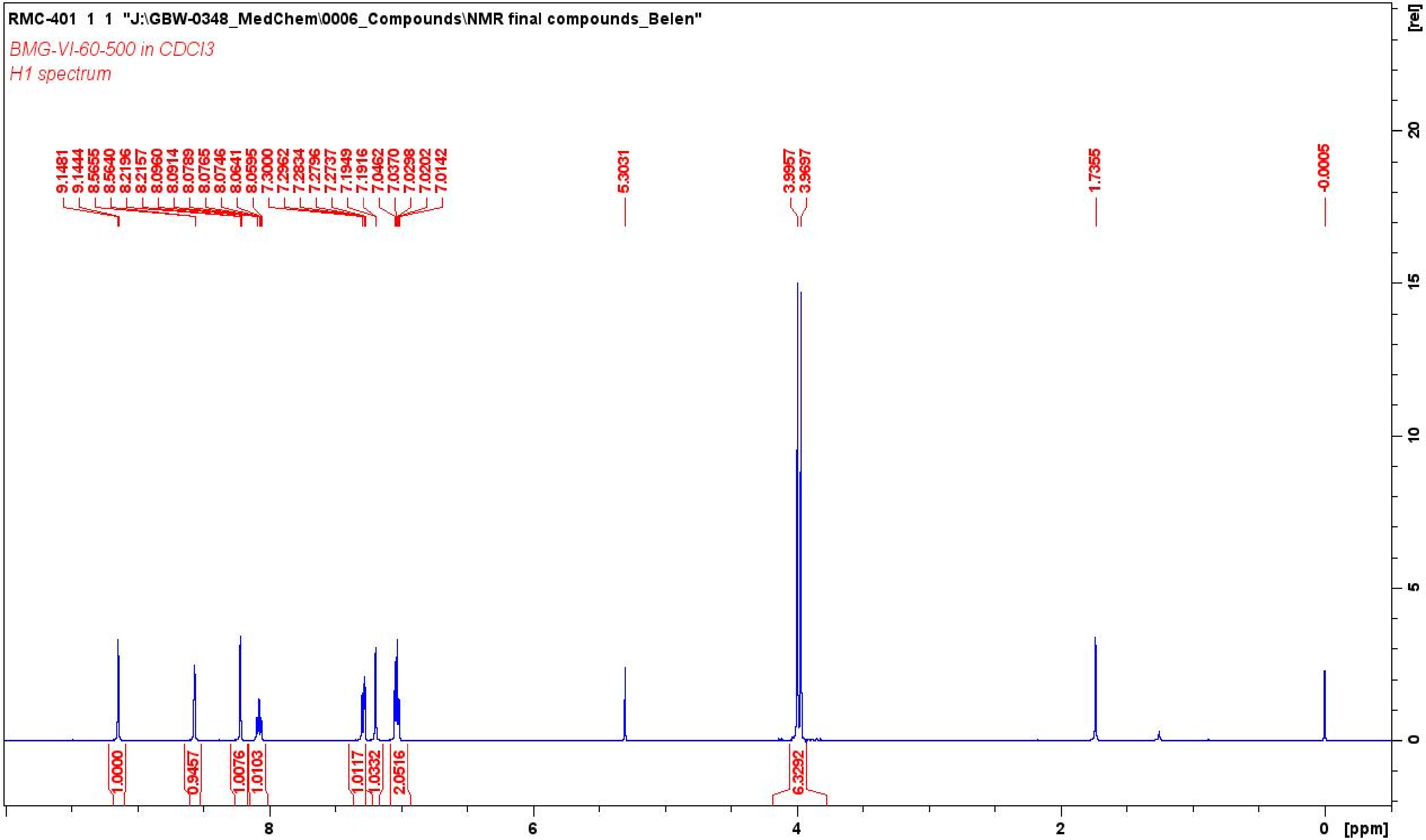

^{13}C NMR spectrum of **7j**

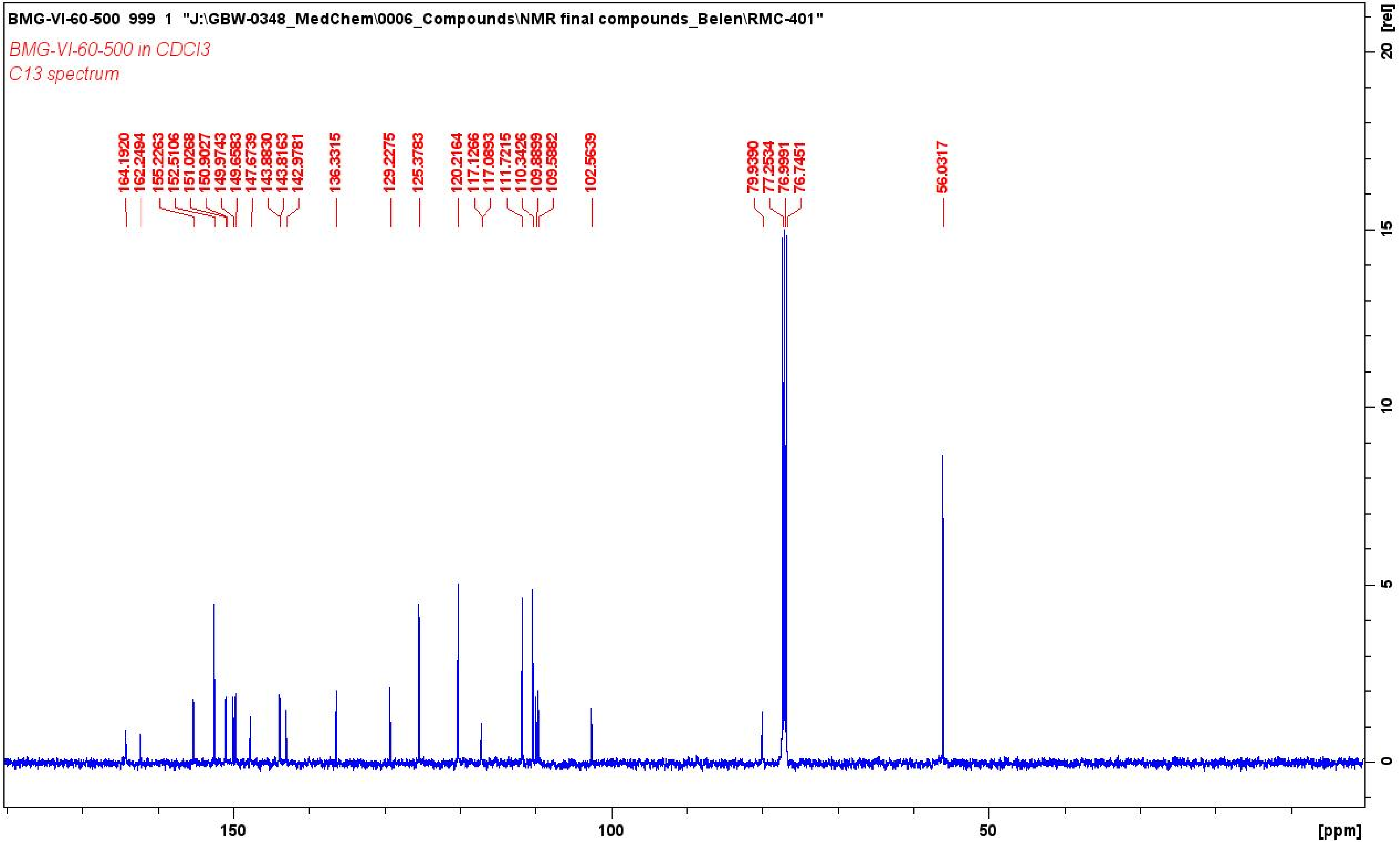

^{1}H NMR spectrum of **7k**

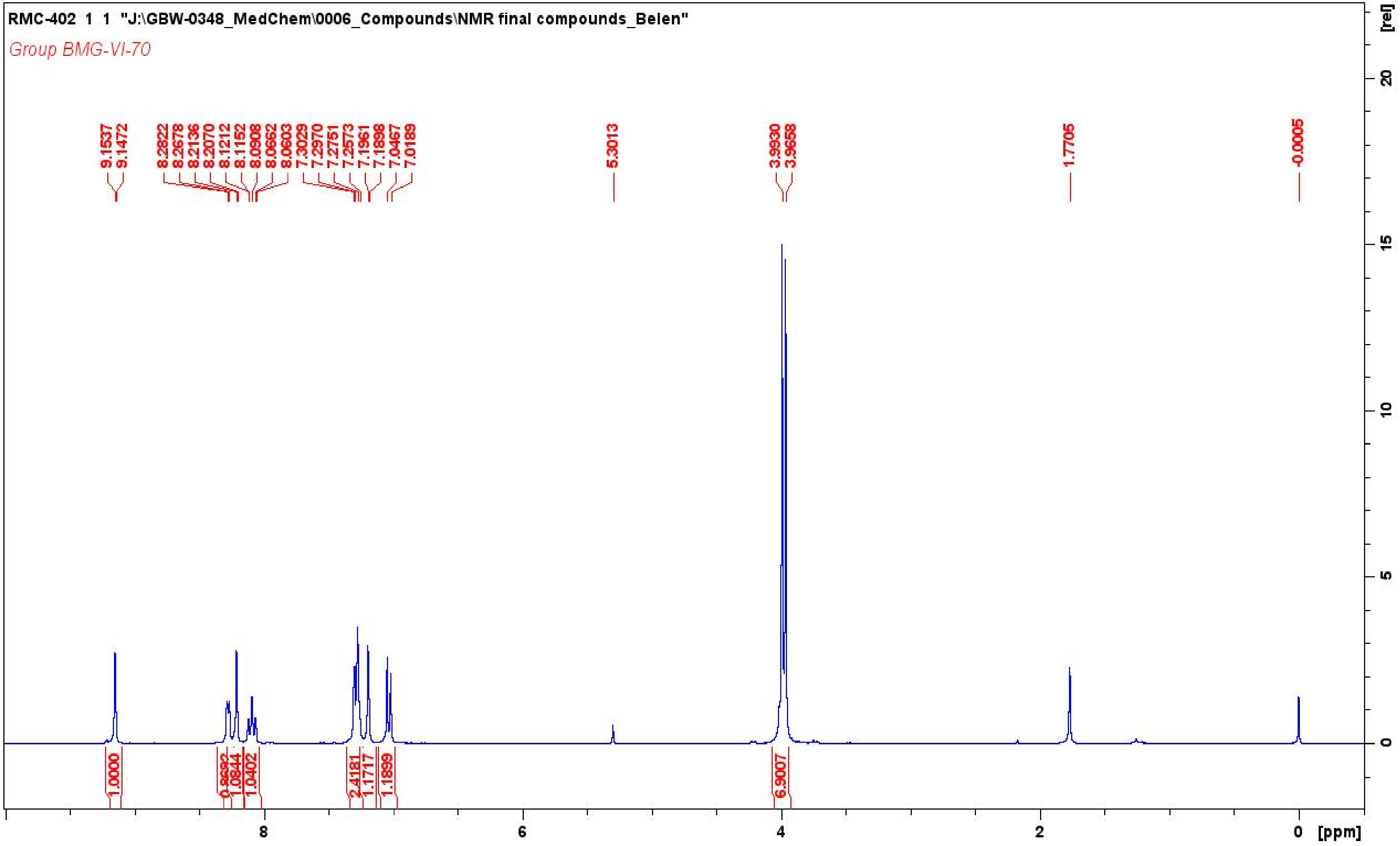

^{13}C NMR spectrum of **7k**

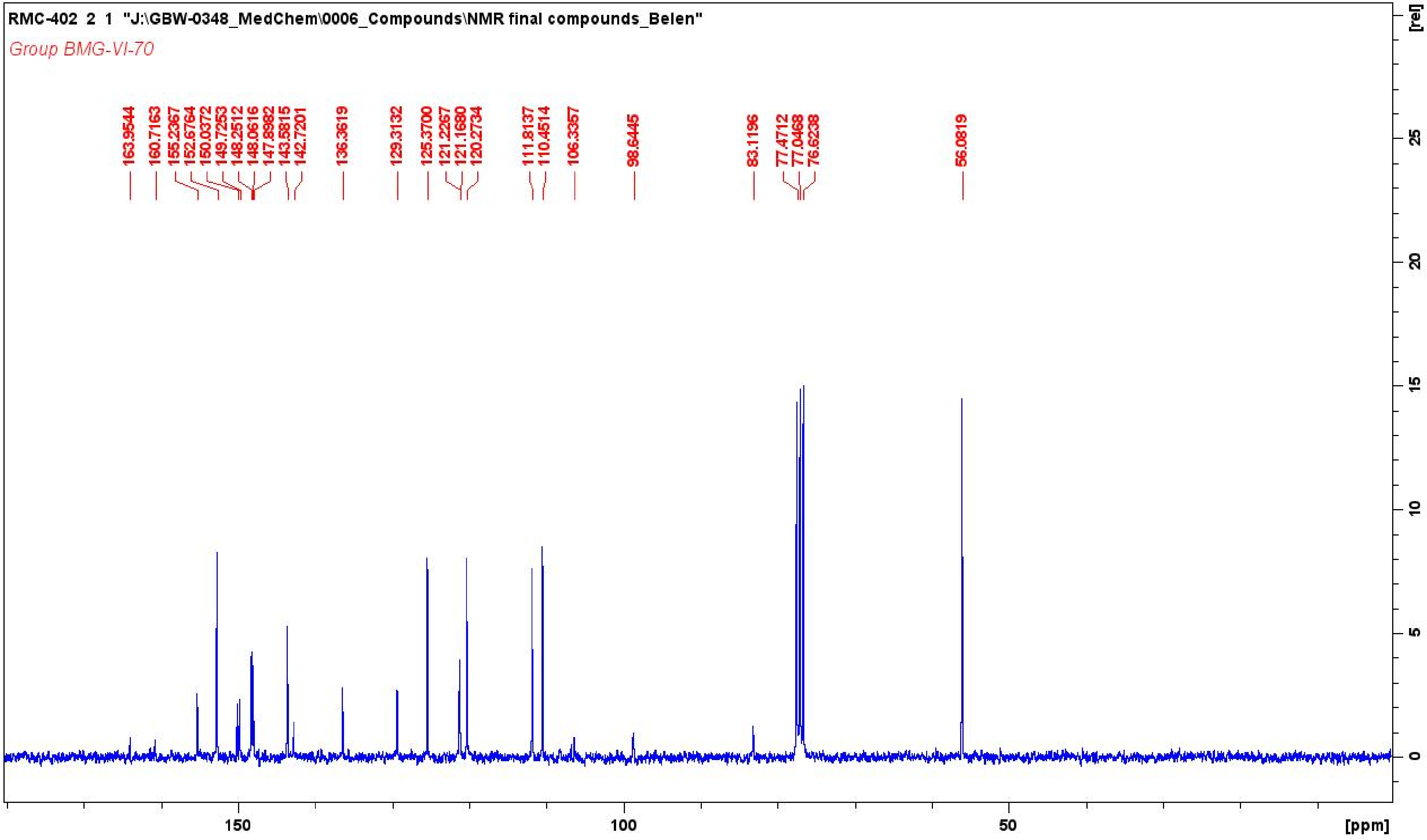

^{1}H NMR spectrum of **7l**

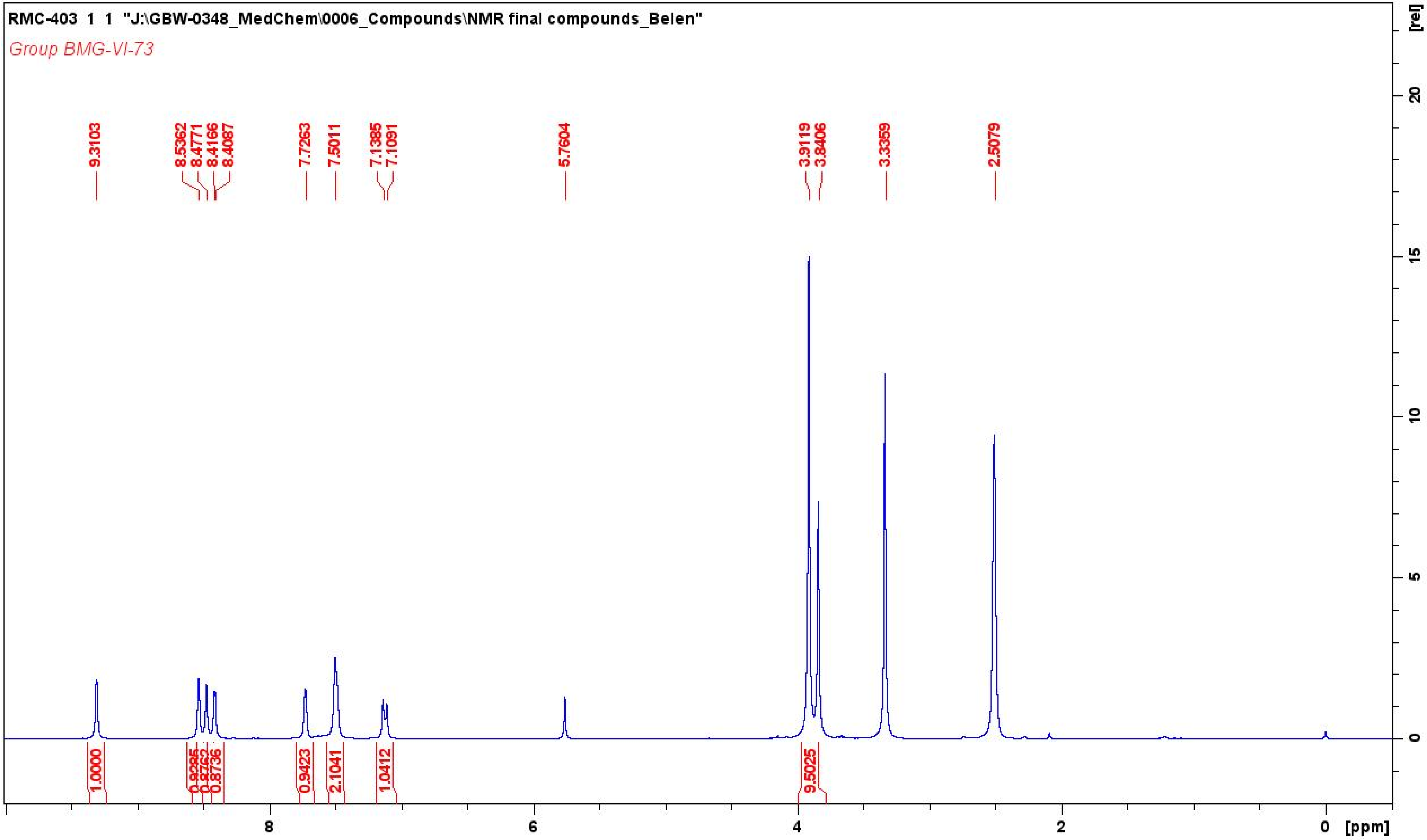

^{13}C NMR spectrum of **7l**

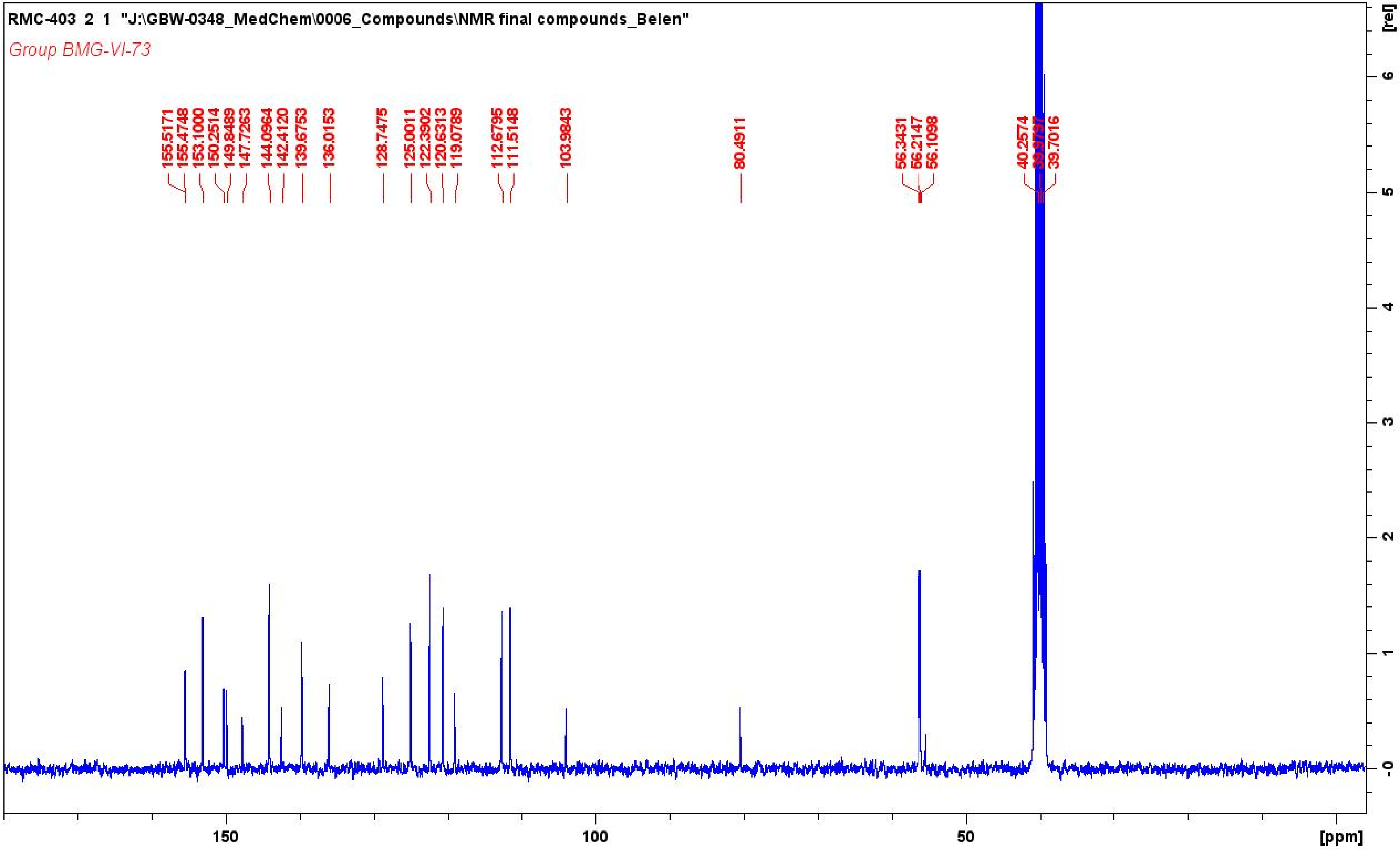

^{1}H NMR spectrum of **7m**

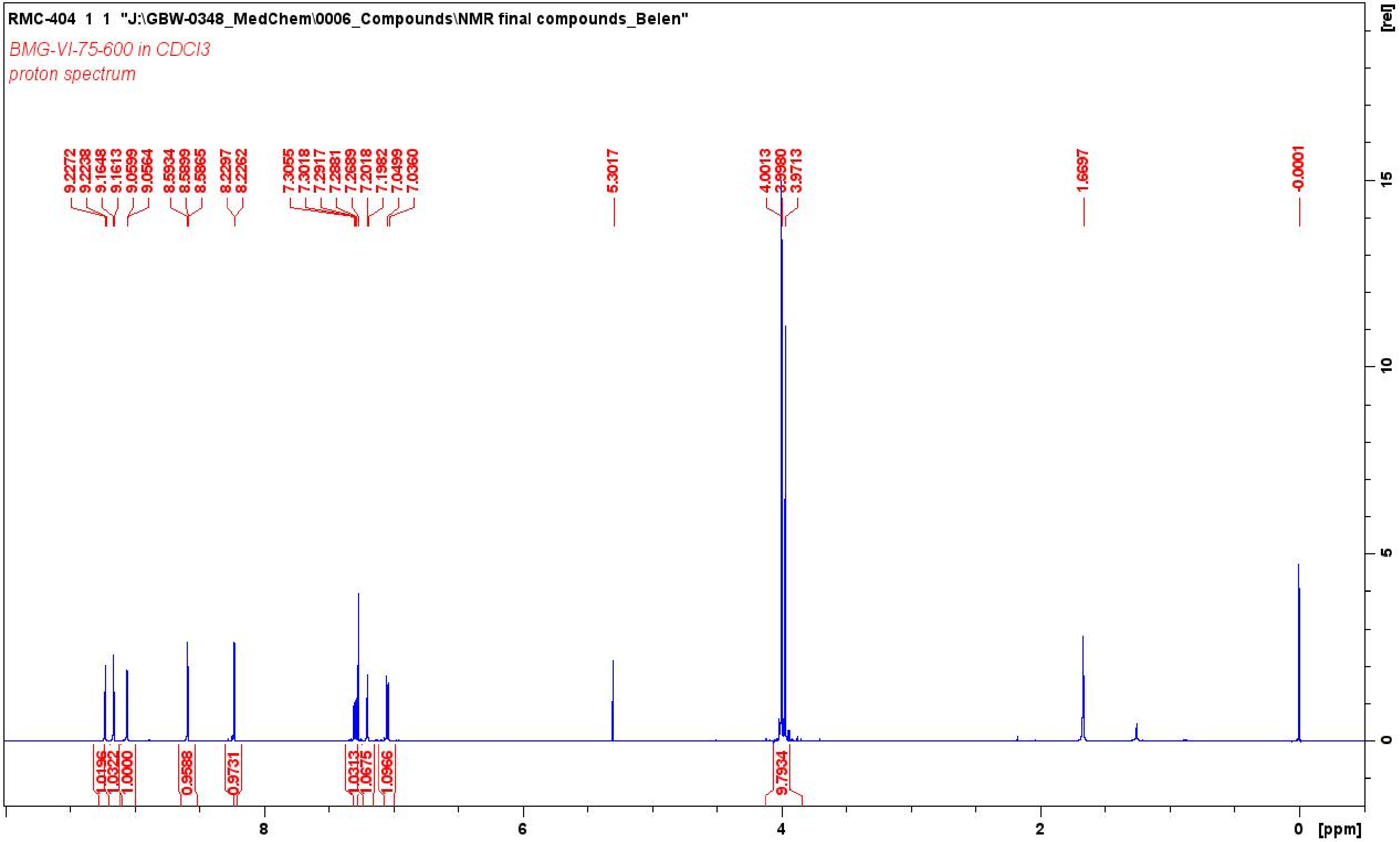

^{13}C NMR spectrum of **7m**

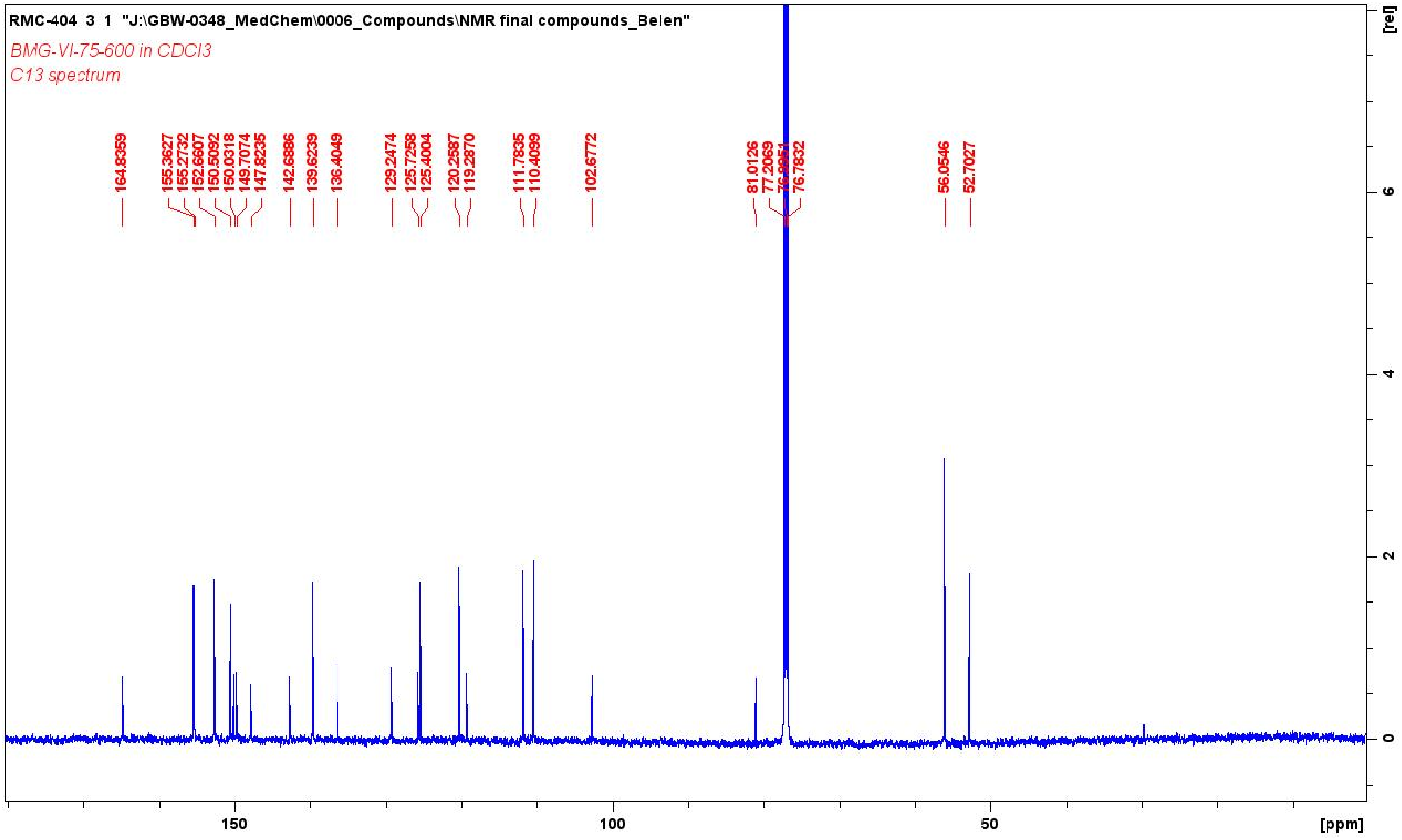

^{1}H NMR spectrum of **7n**

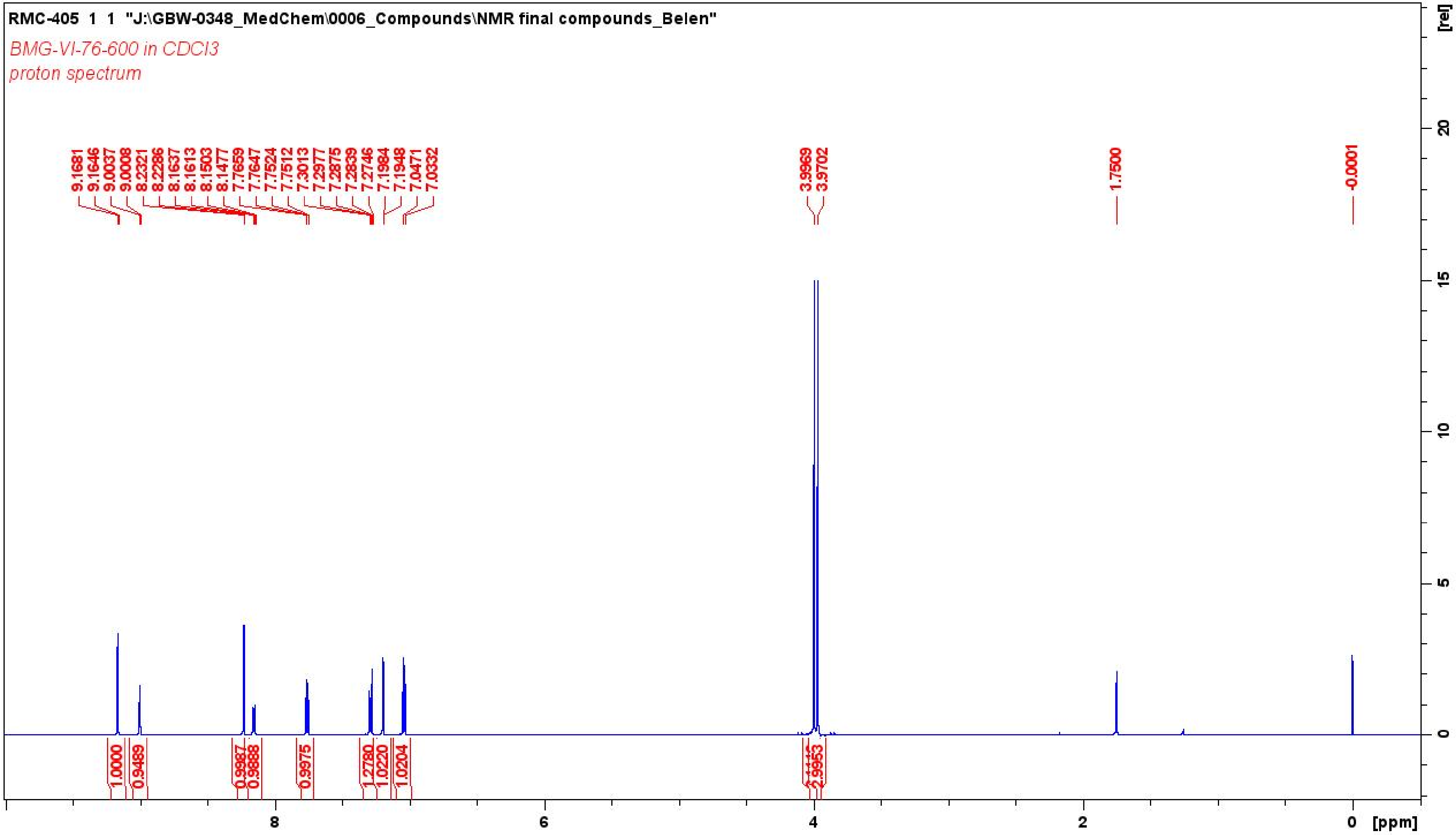

^{13}C NMR spectrum of **7n**

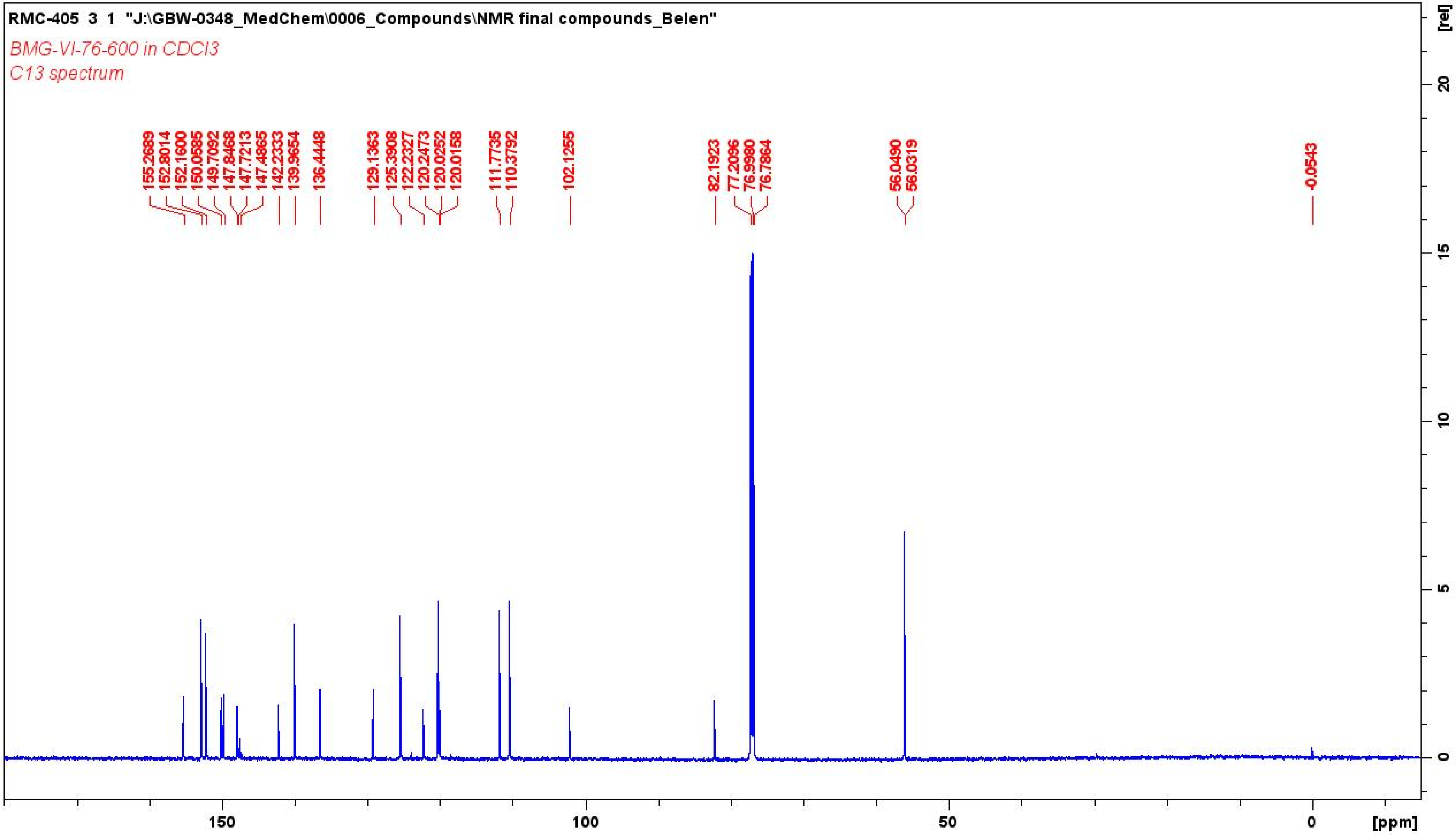

^{1}H NMR spectrum of **7o**

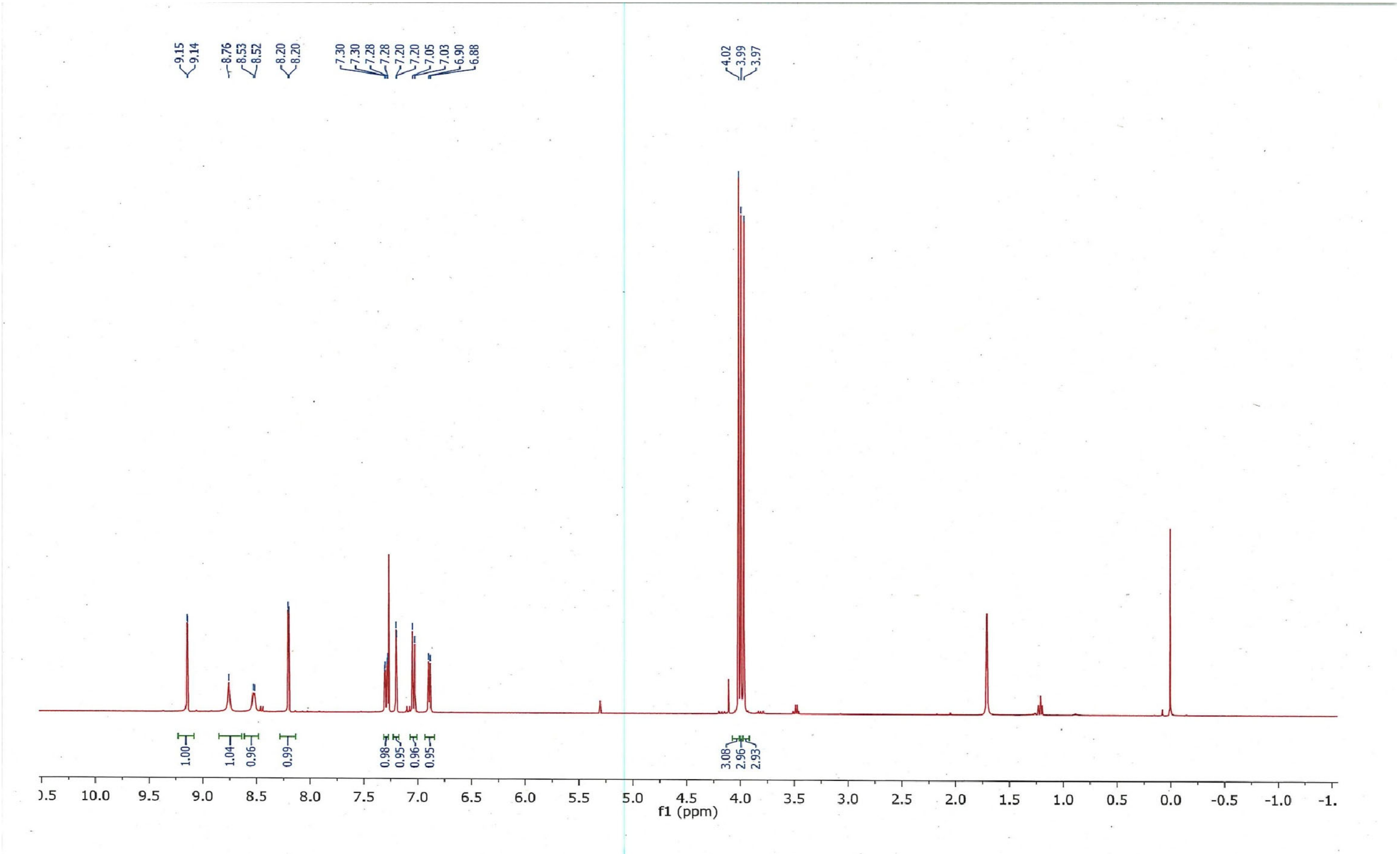

^{13}C NMR spectrum of **7o**

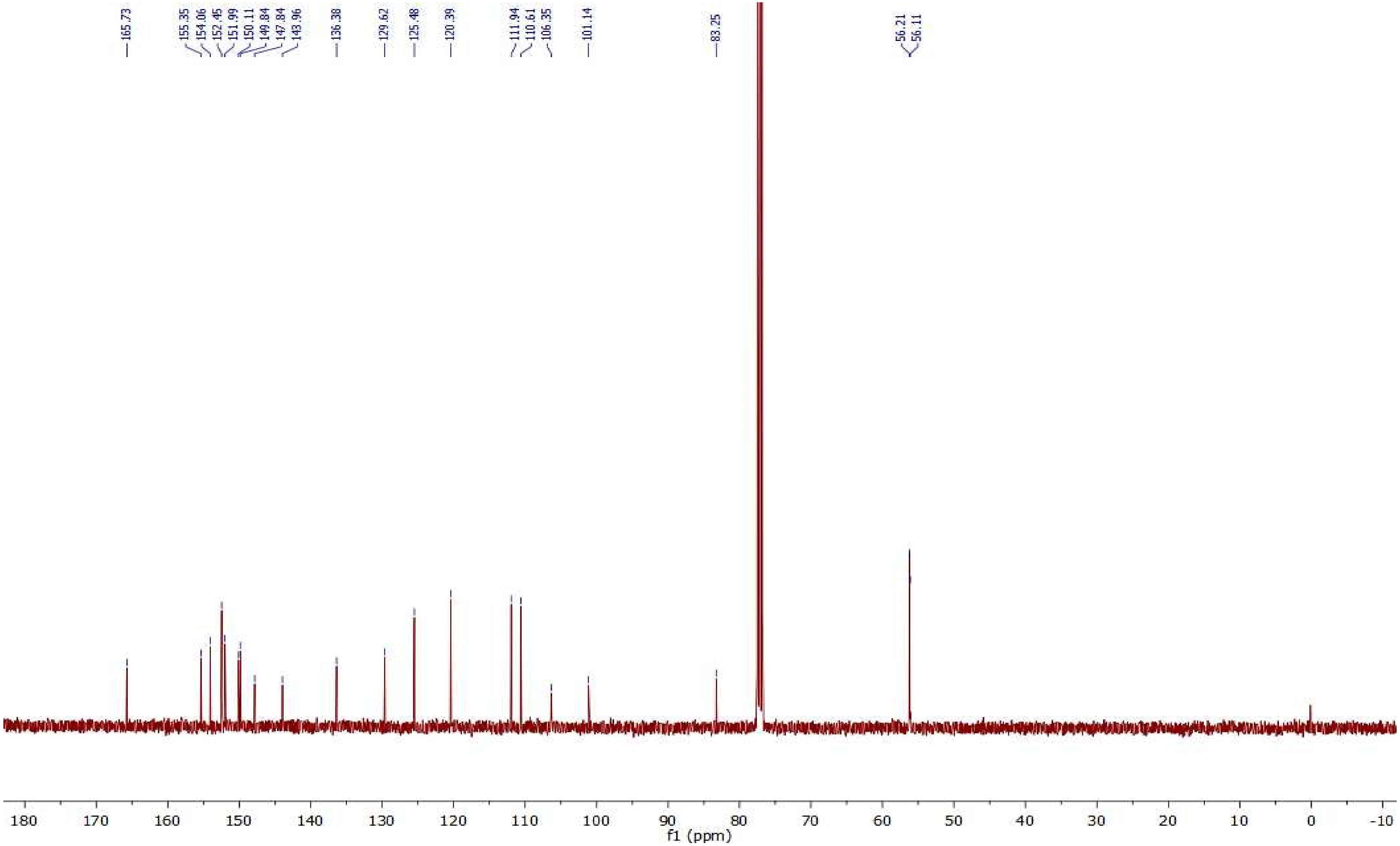

^{1}H NMR spectrum of **7p**

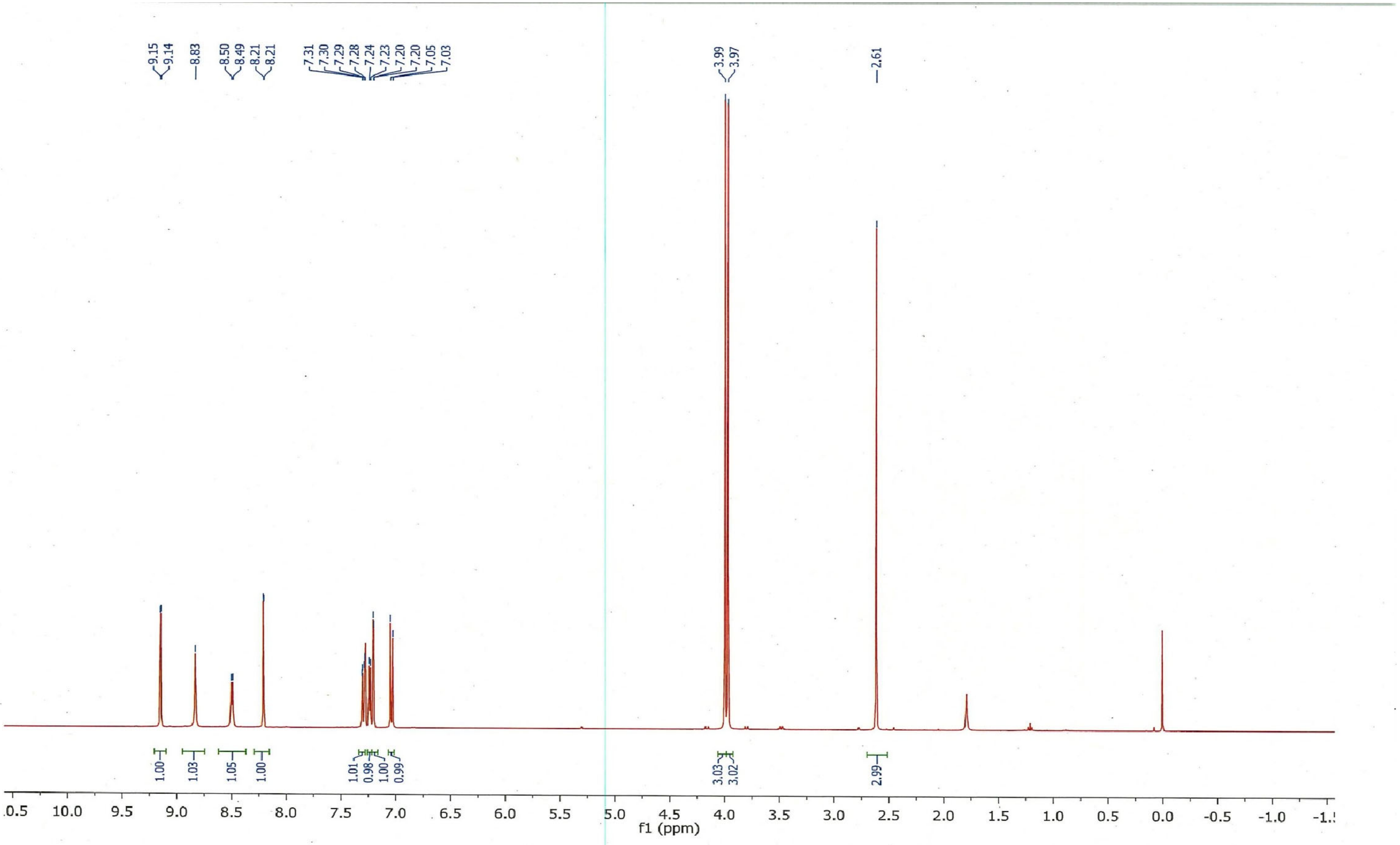

^{13}C NMR spectrum of **7p**

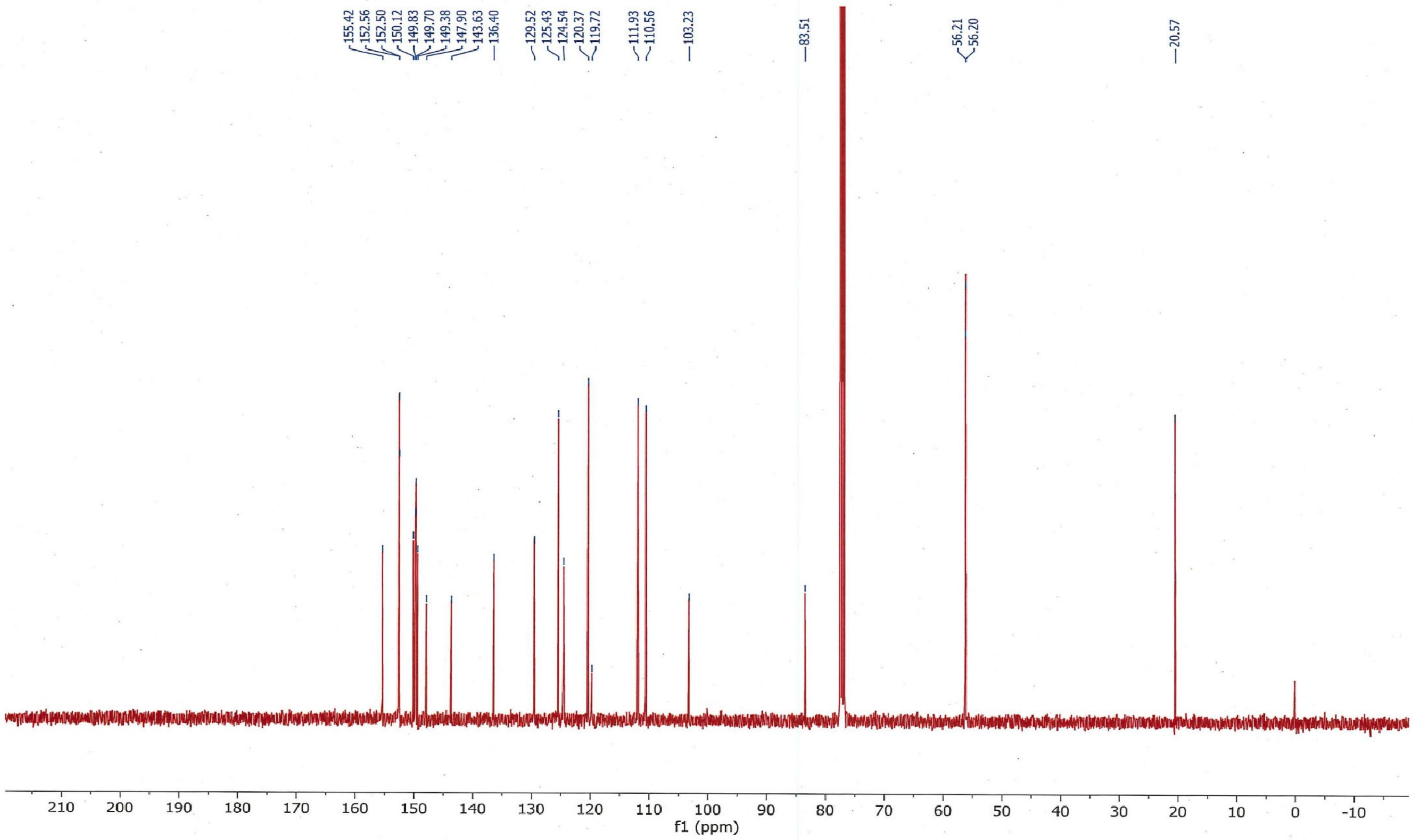

^{1}H NMR spectrum of **7q**

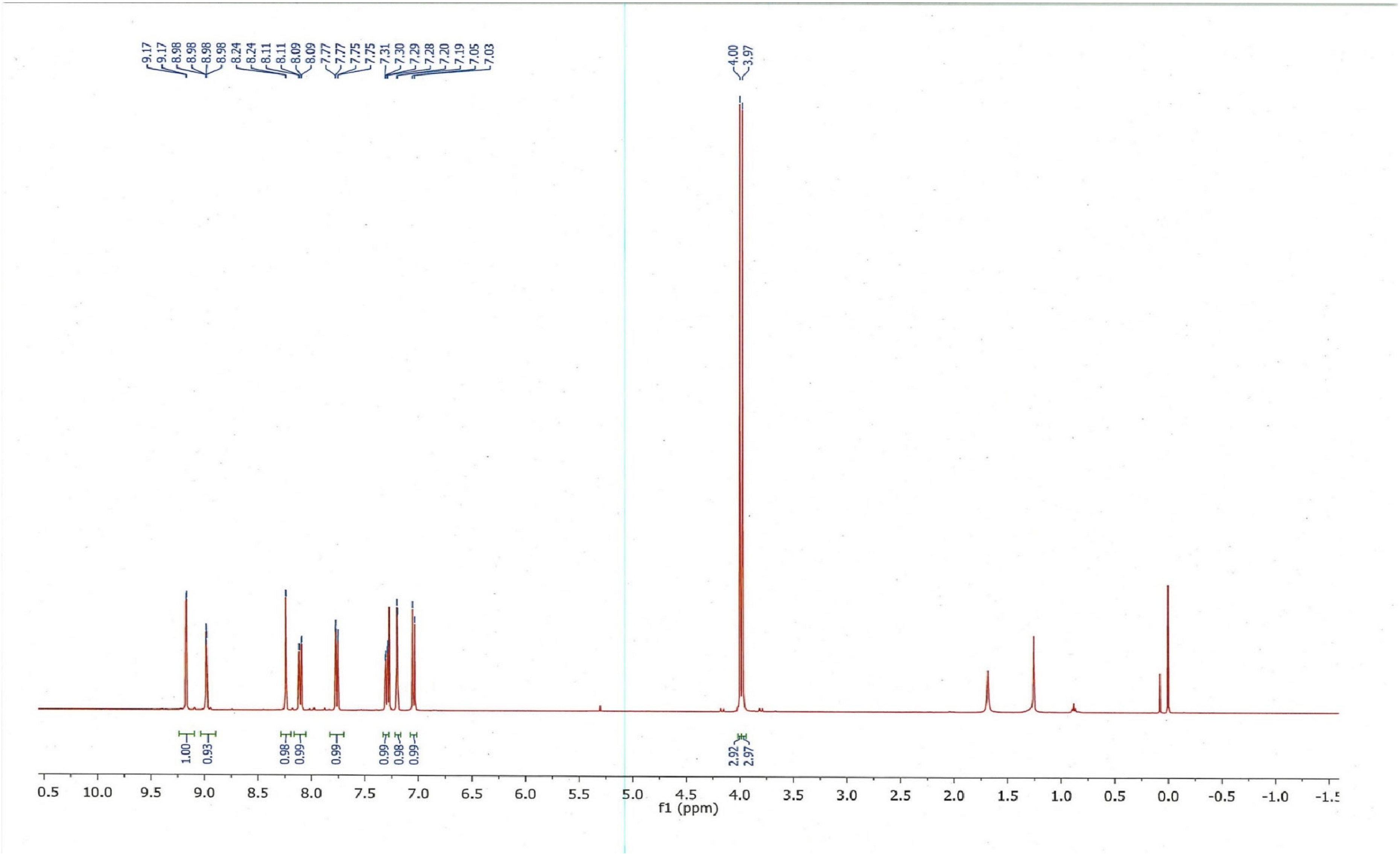

^{13}C NMR spectrum of **7q**

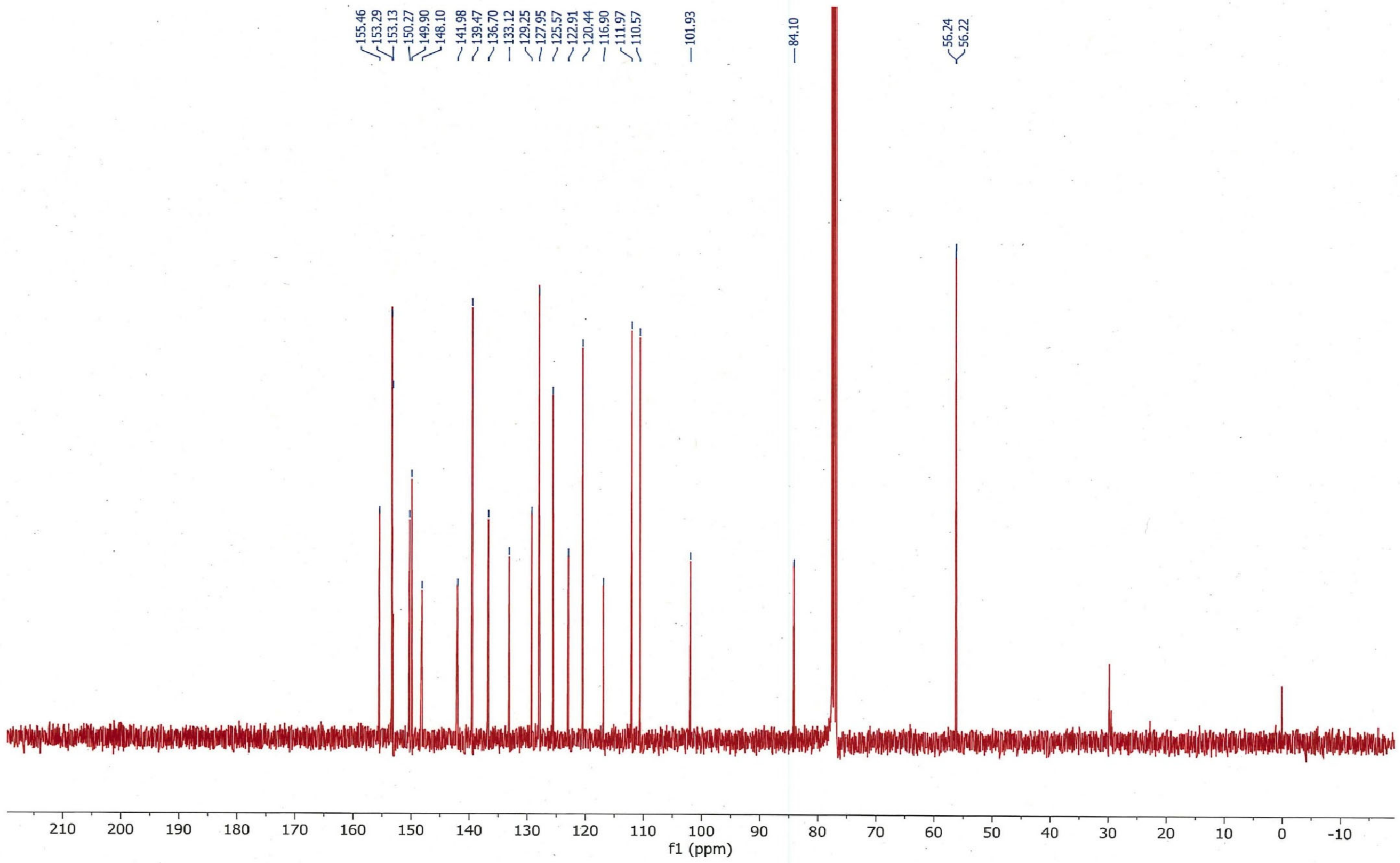

^{1}H NMR spectrum of **7r**

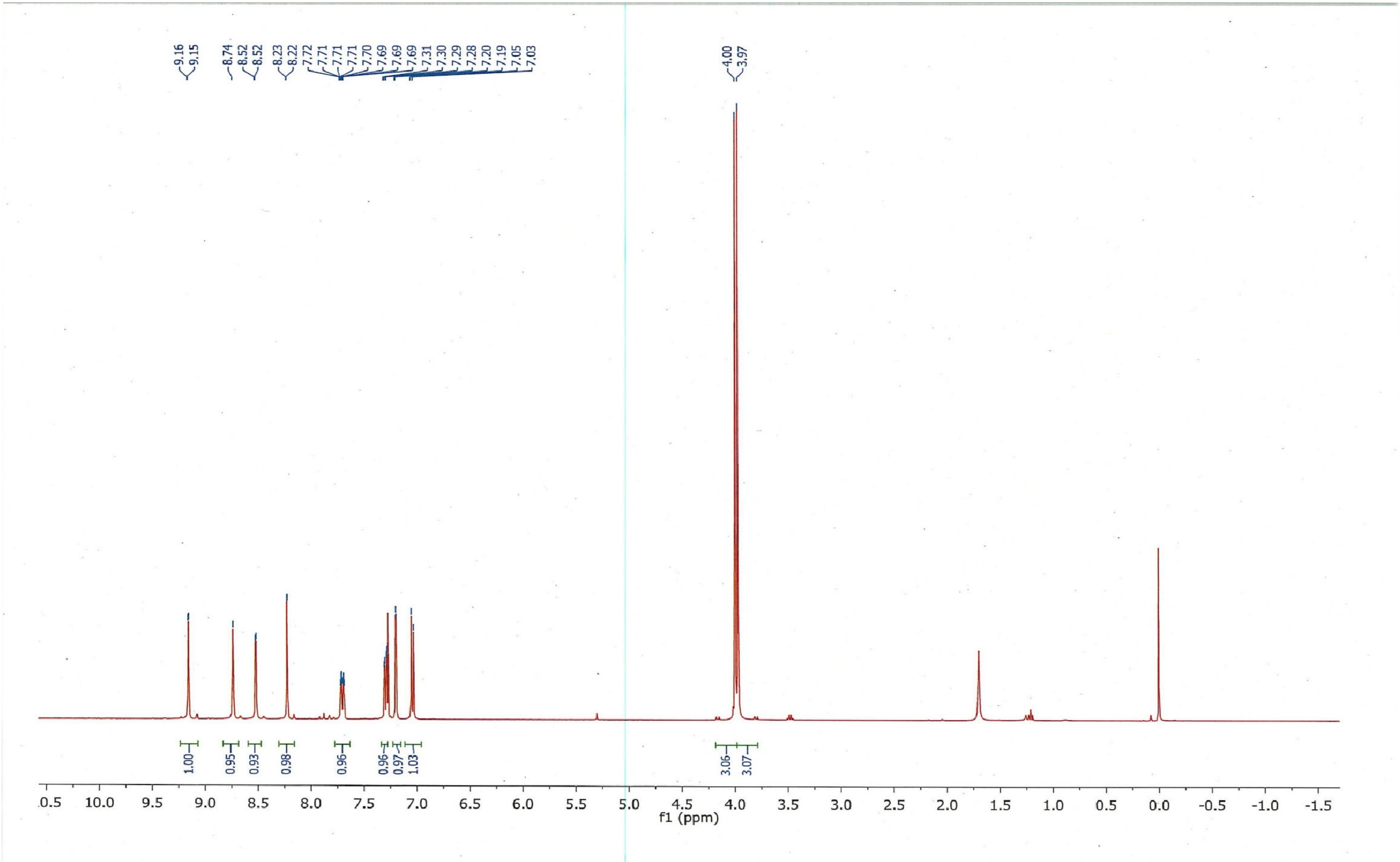

^{13}C NMR spectrum of **7r**

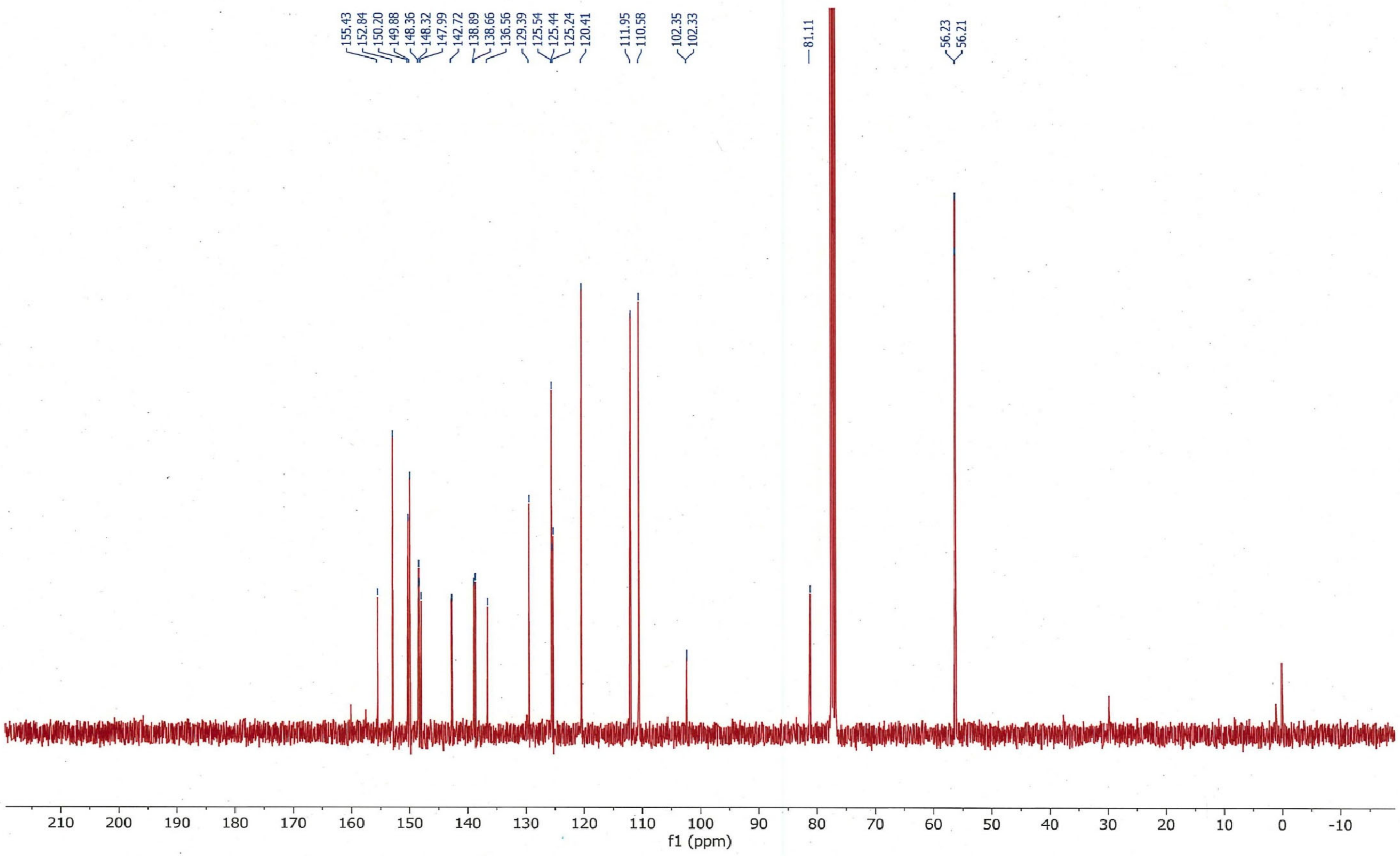

^{1}H NMR spectrum of **7s**

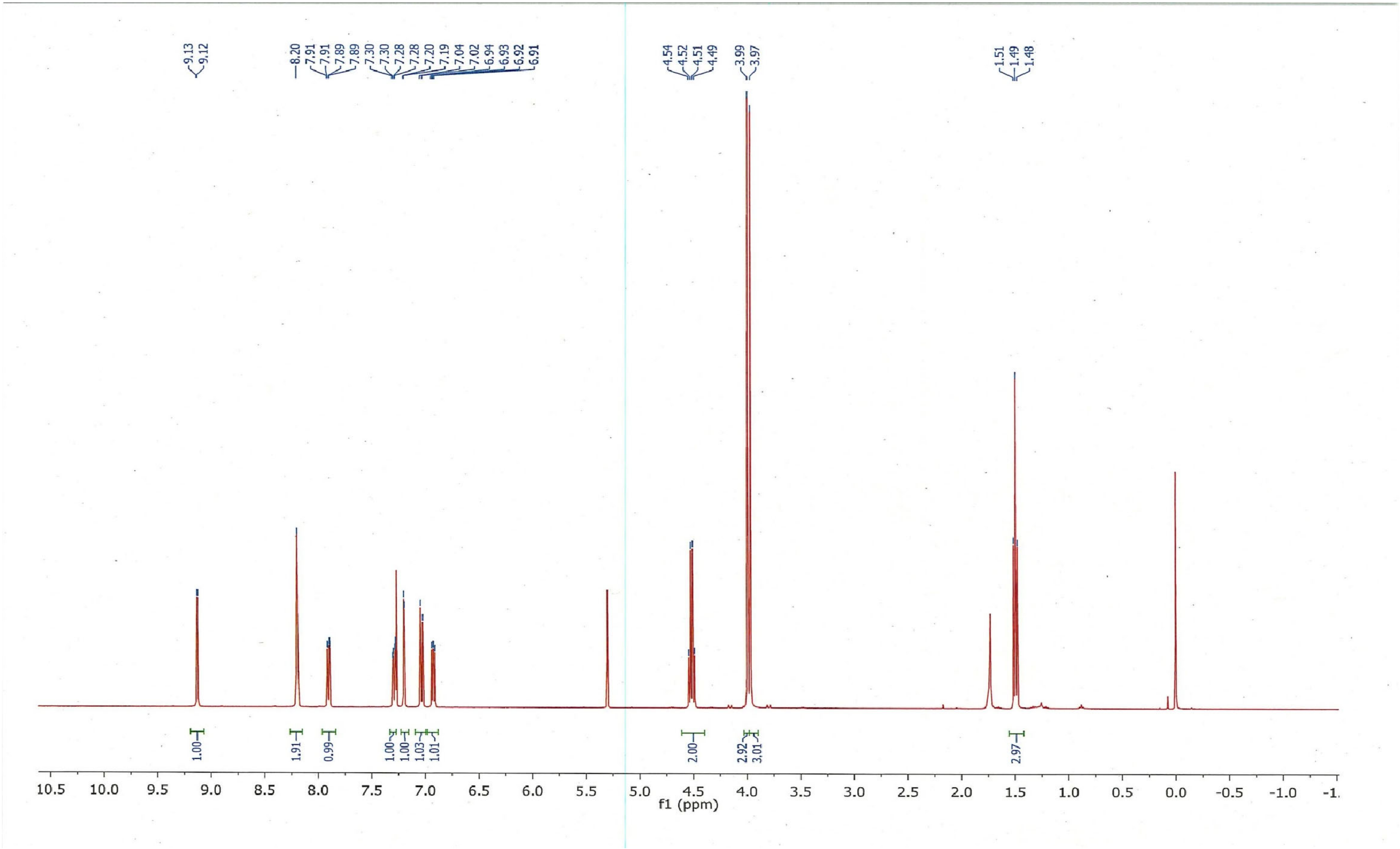

^{13}C NMR spectrum of **7s**

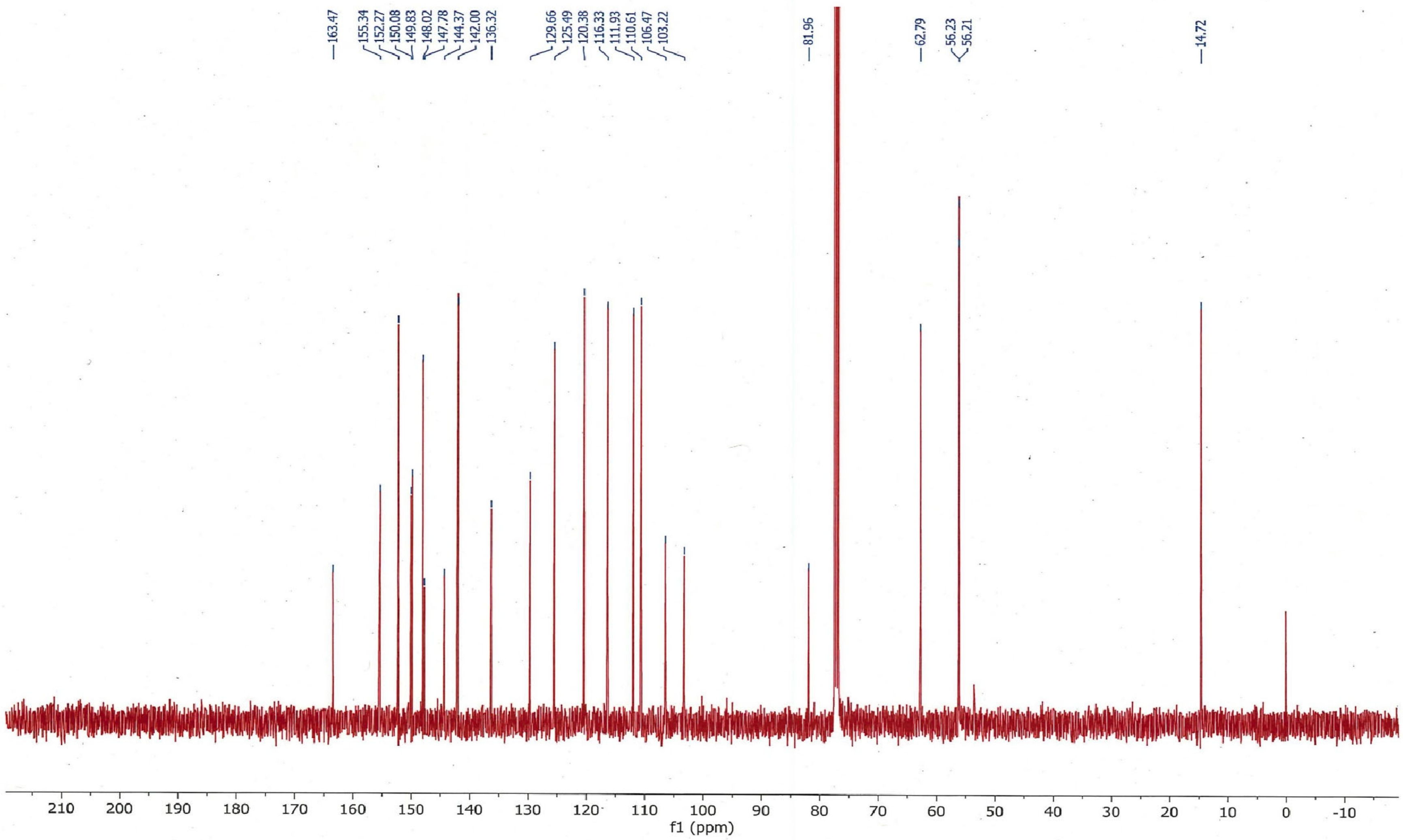

^{1}H NMR spectrum of **7t**

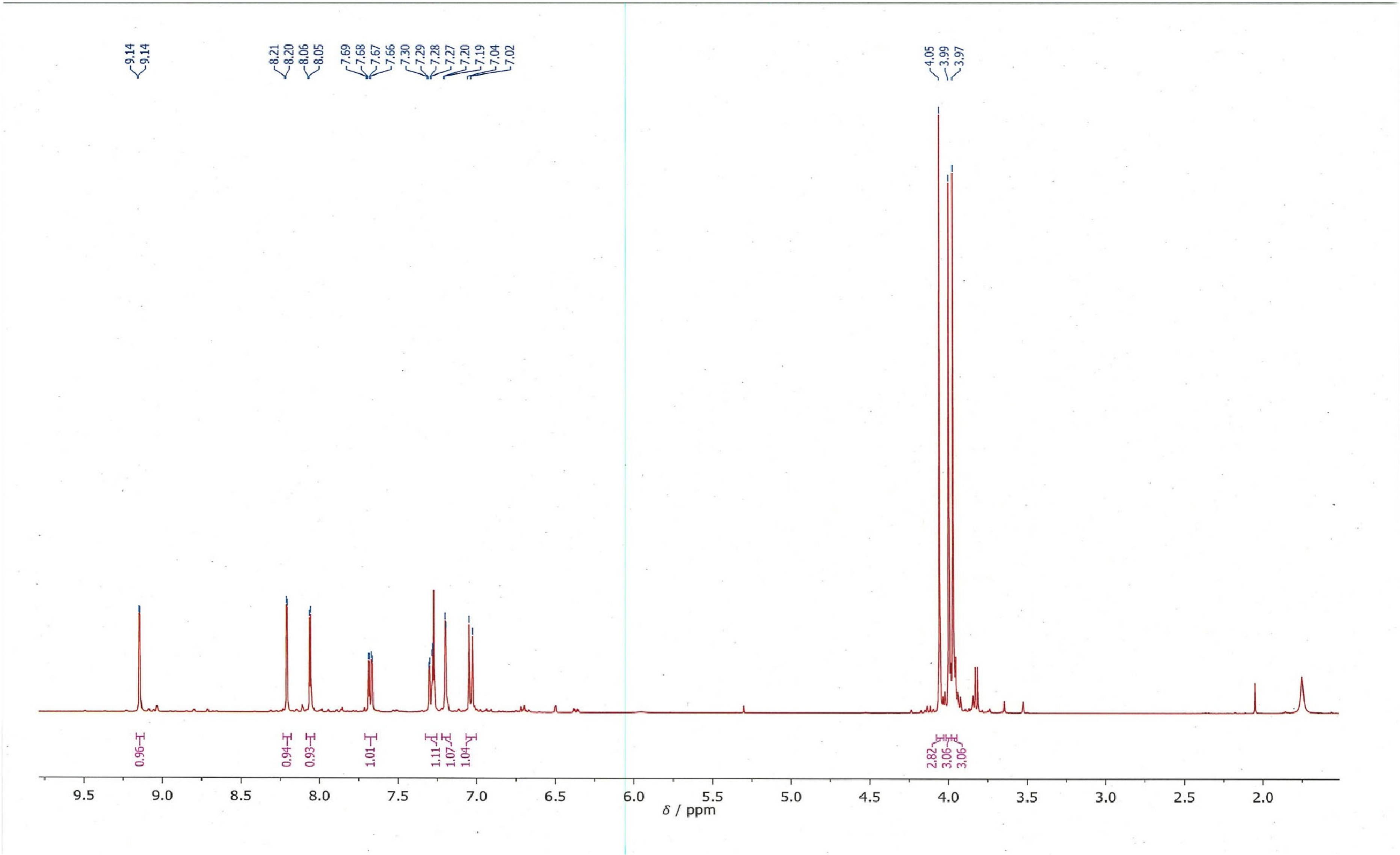

^{13}C NMR spectrum of **7t**

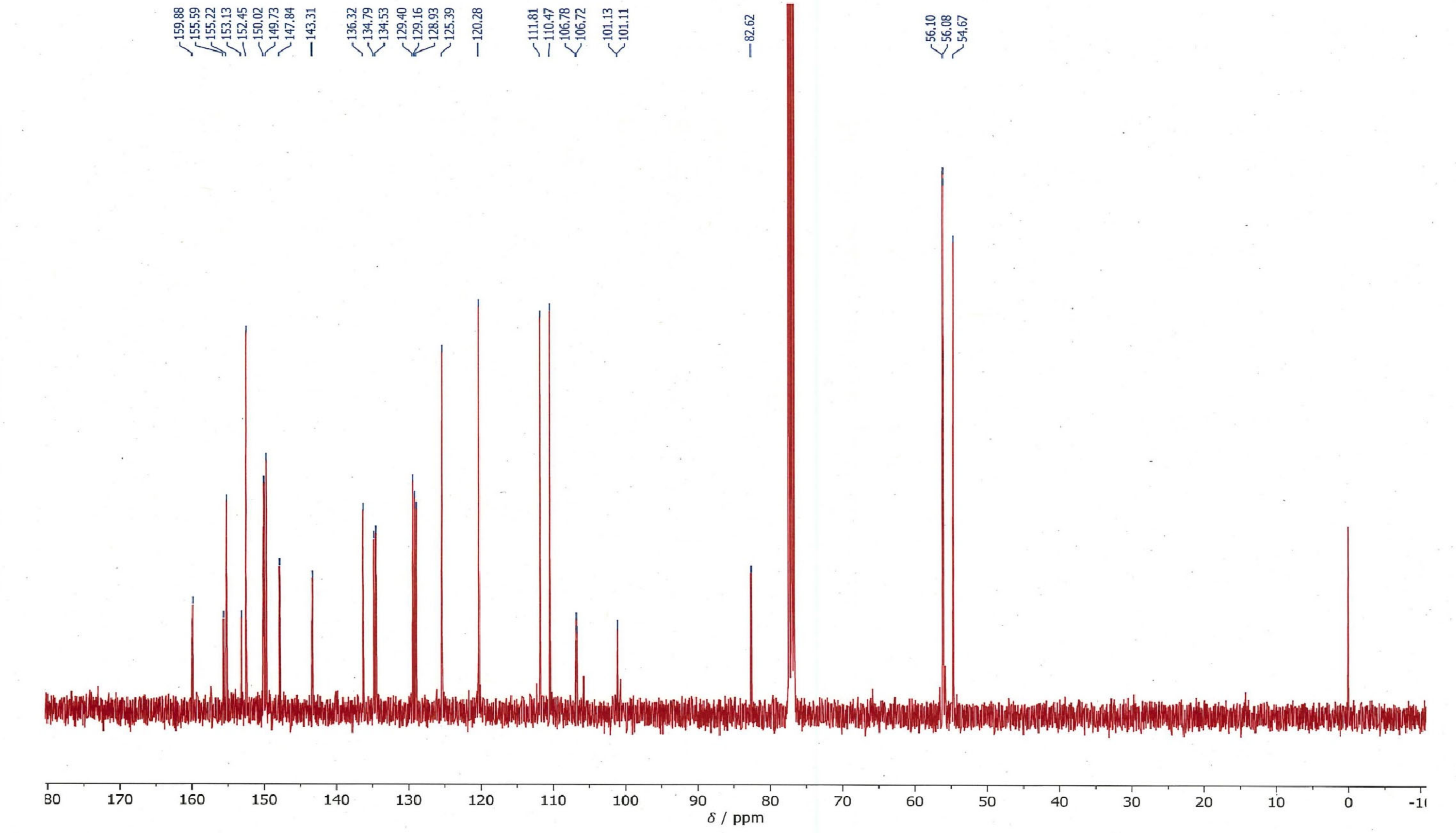

^{1}H NMR spectrum of **7u**

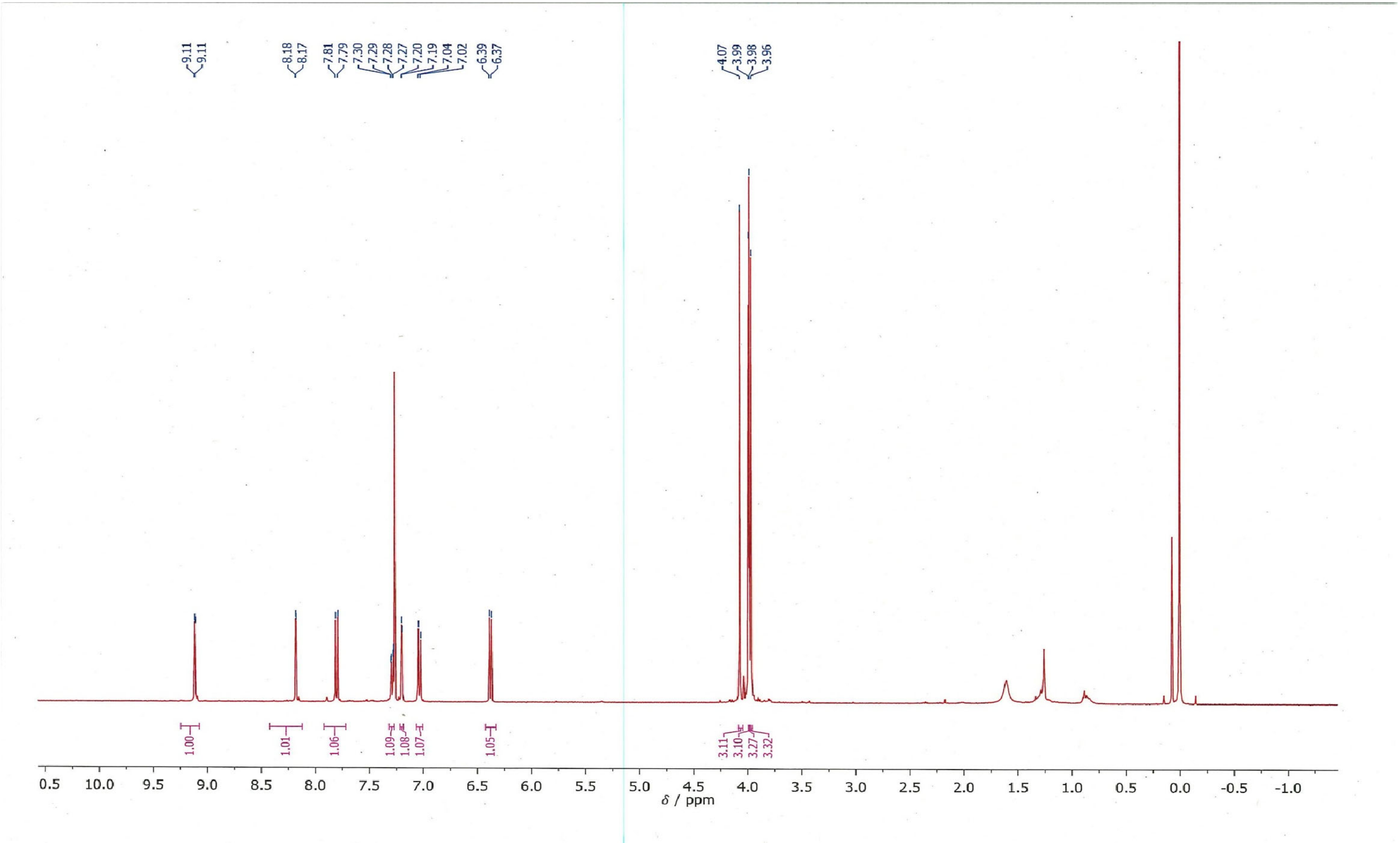

^{13}C NMR spectrum of **7u**

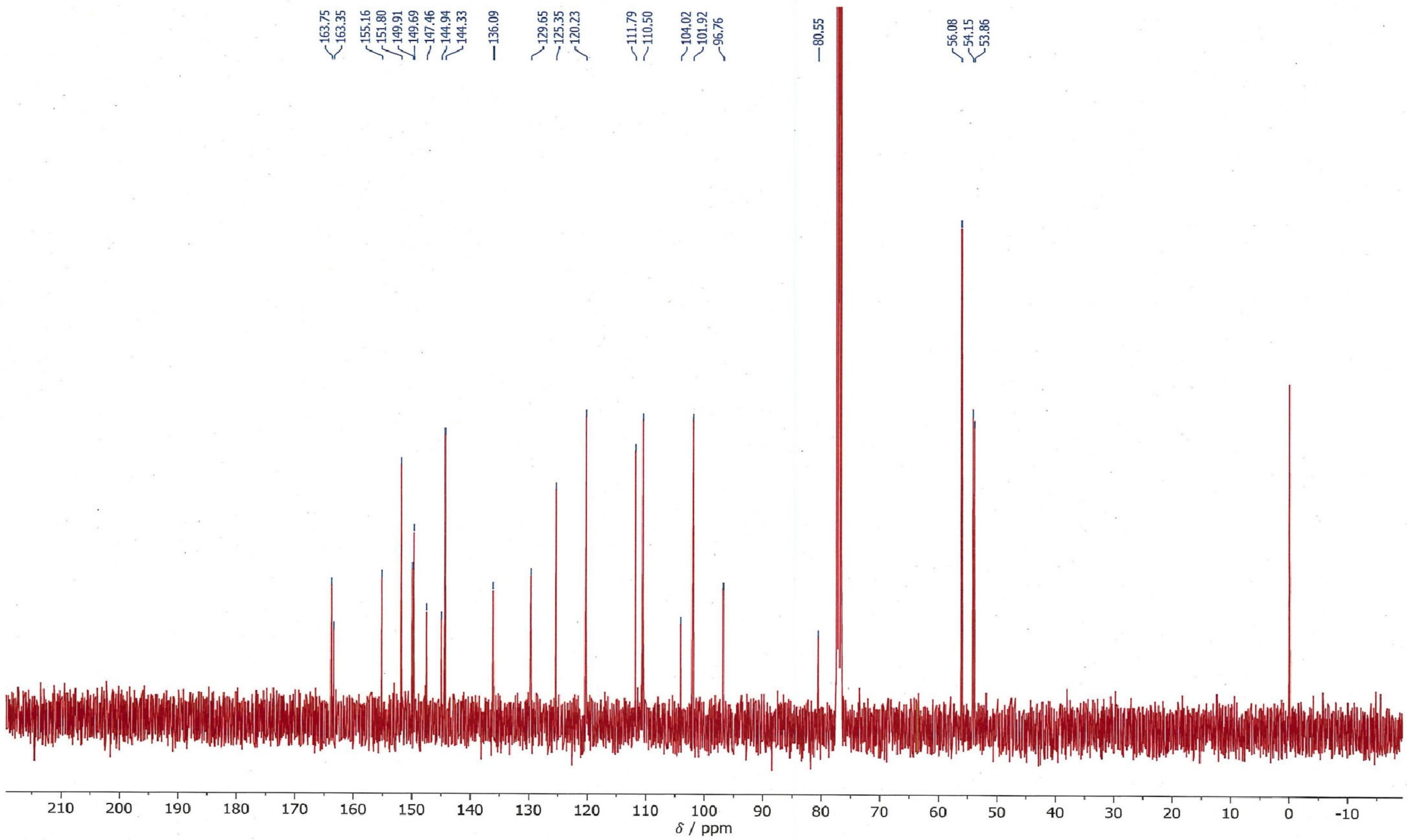

^{1}H NMR spectrum of **7v**

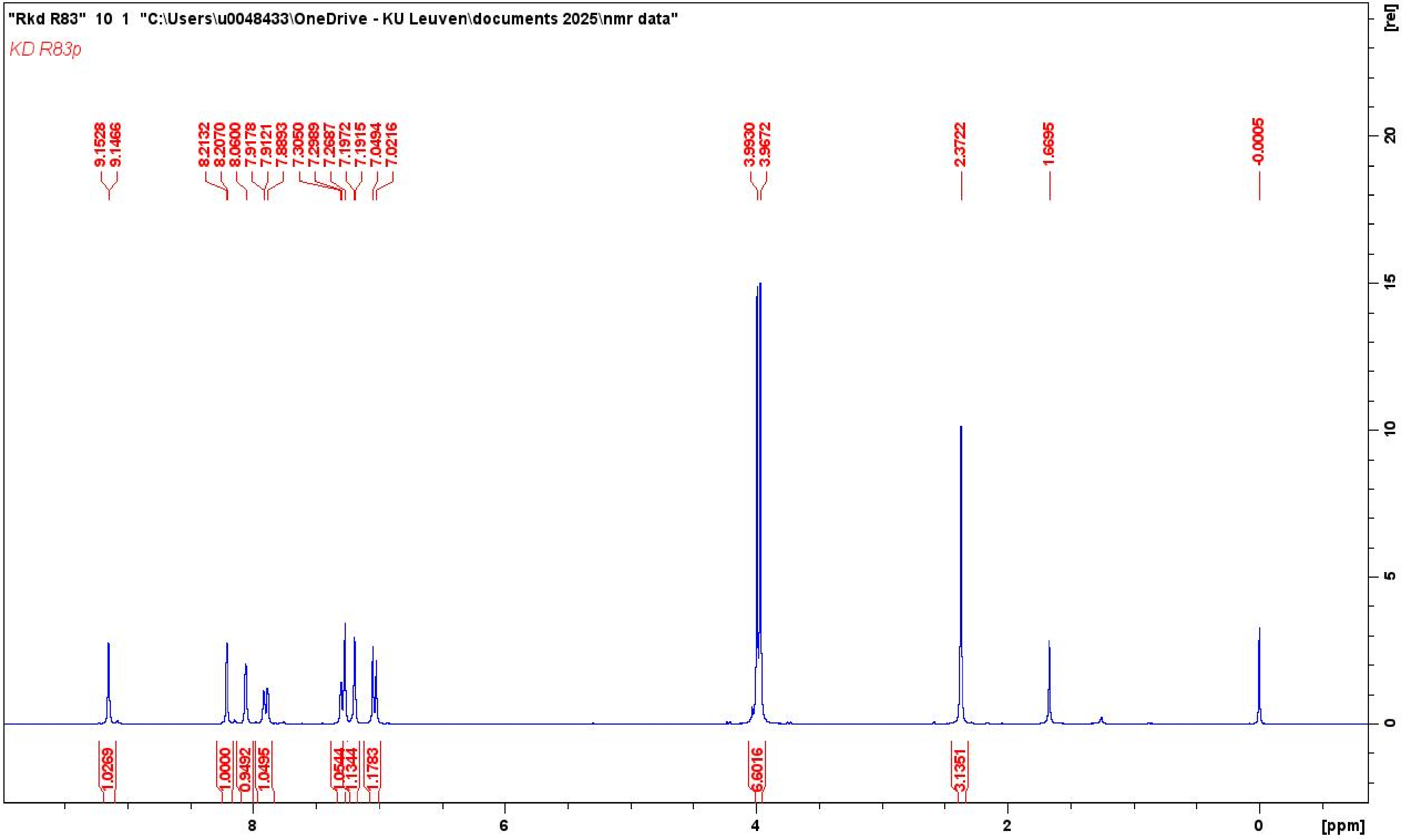

^{13}C NMR spectrum of **7v**

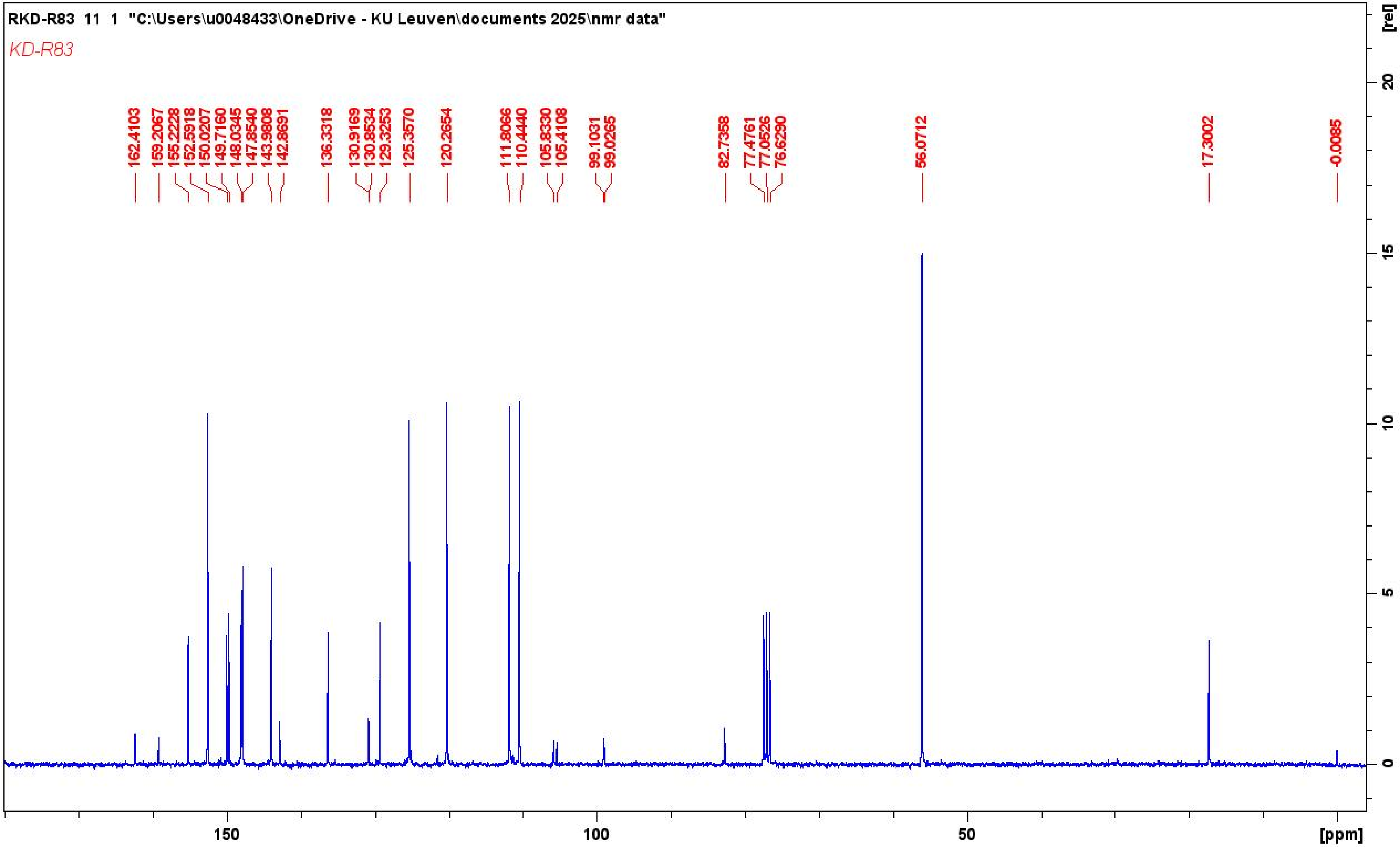

^{1}H NMR spectrum of 7w

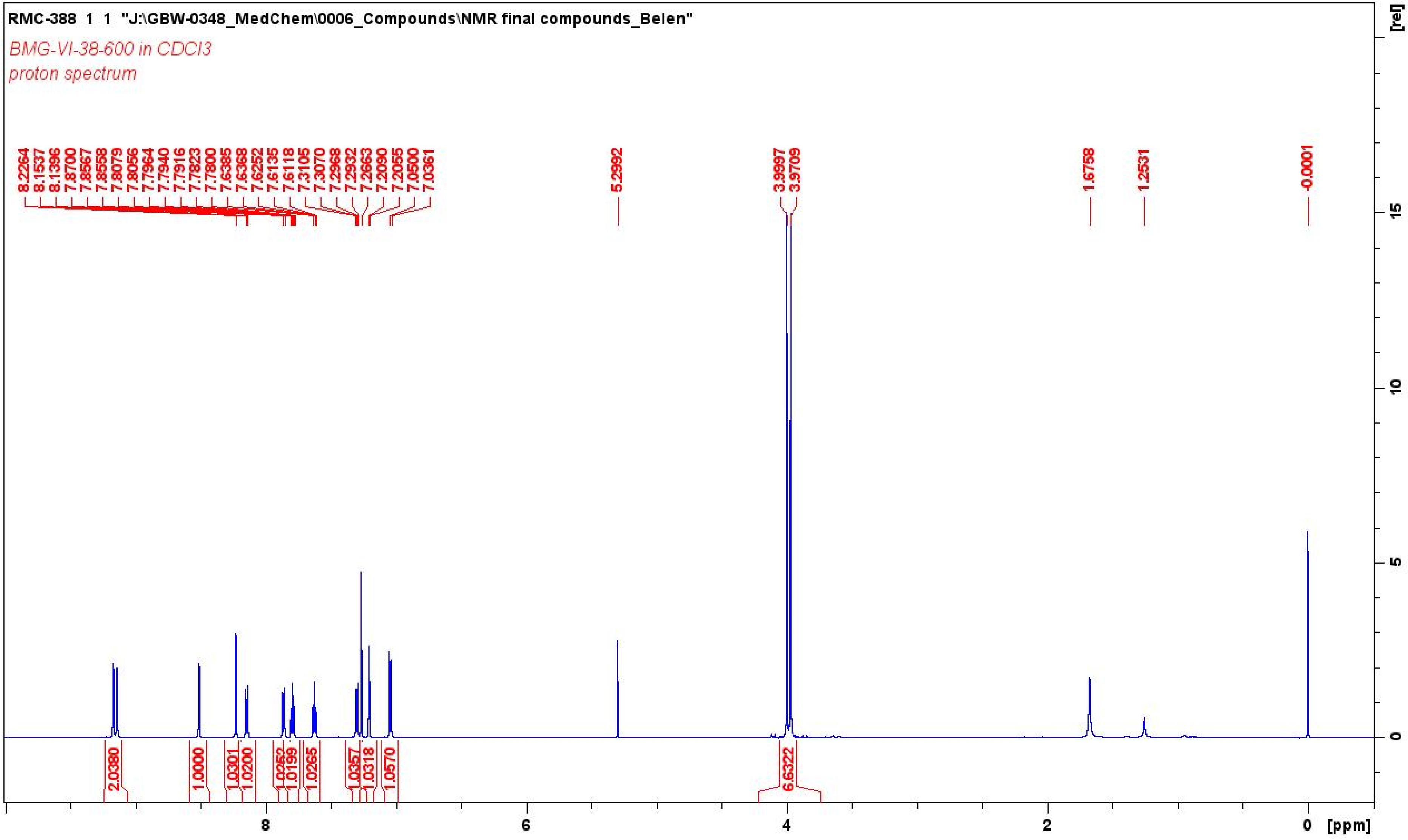

^{13}C NMR spectrum of **7w**

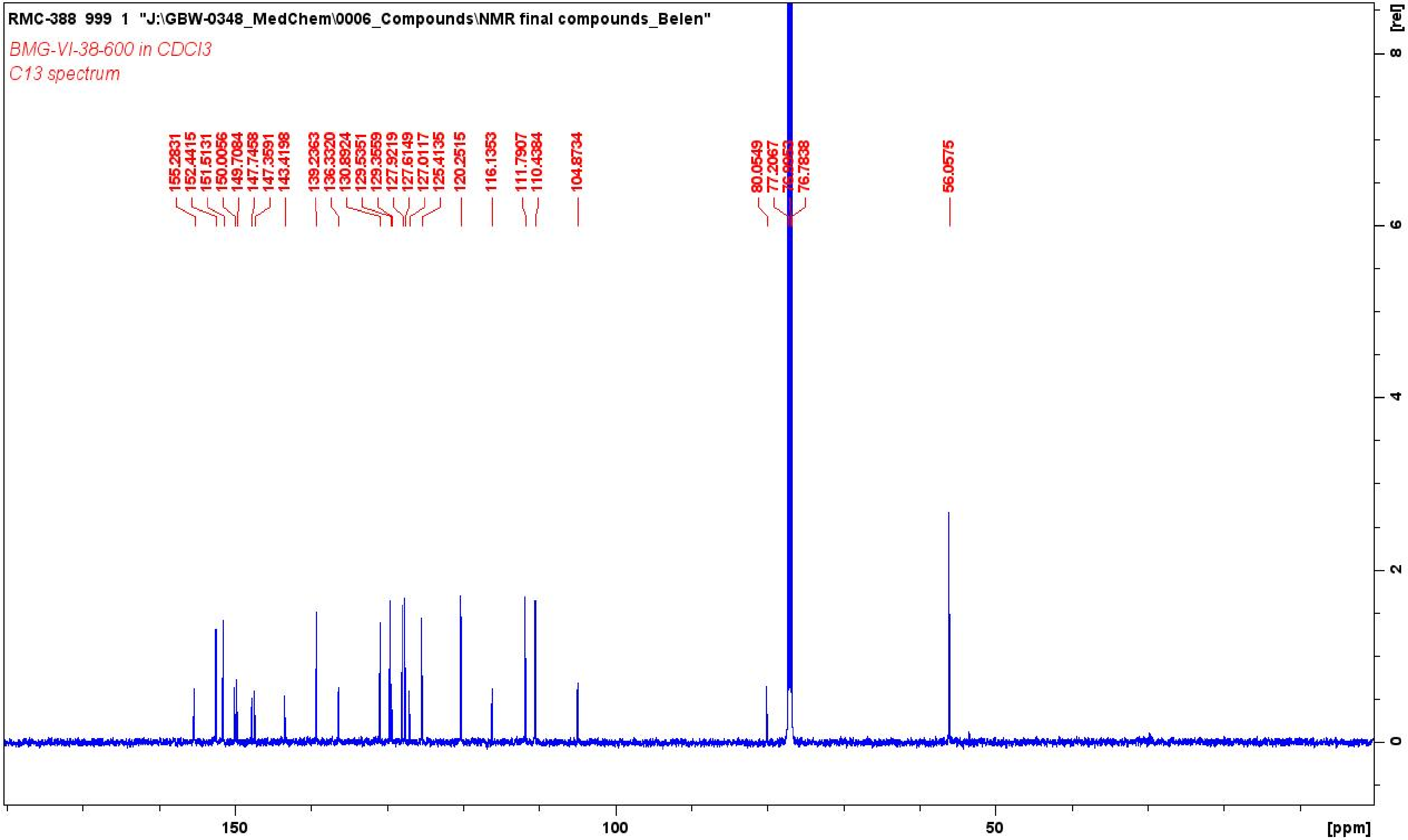

^{1}H NMR spectrum of **7x**

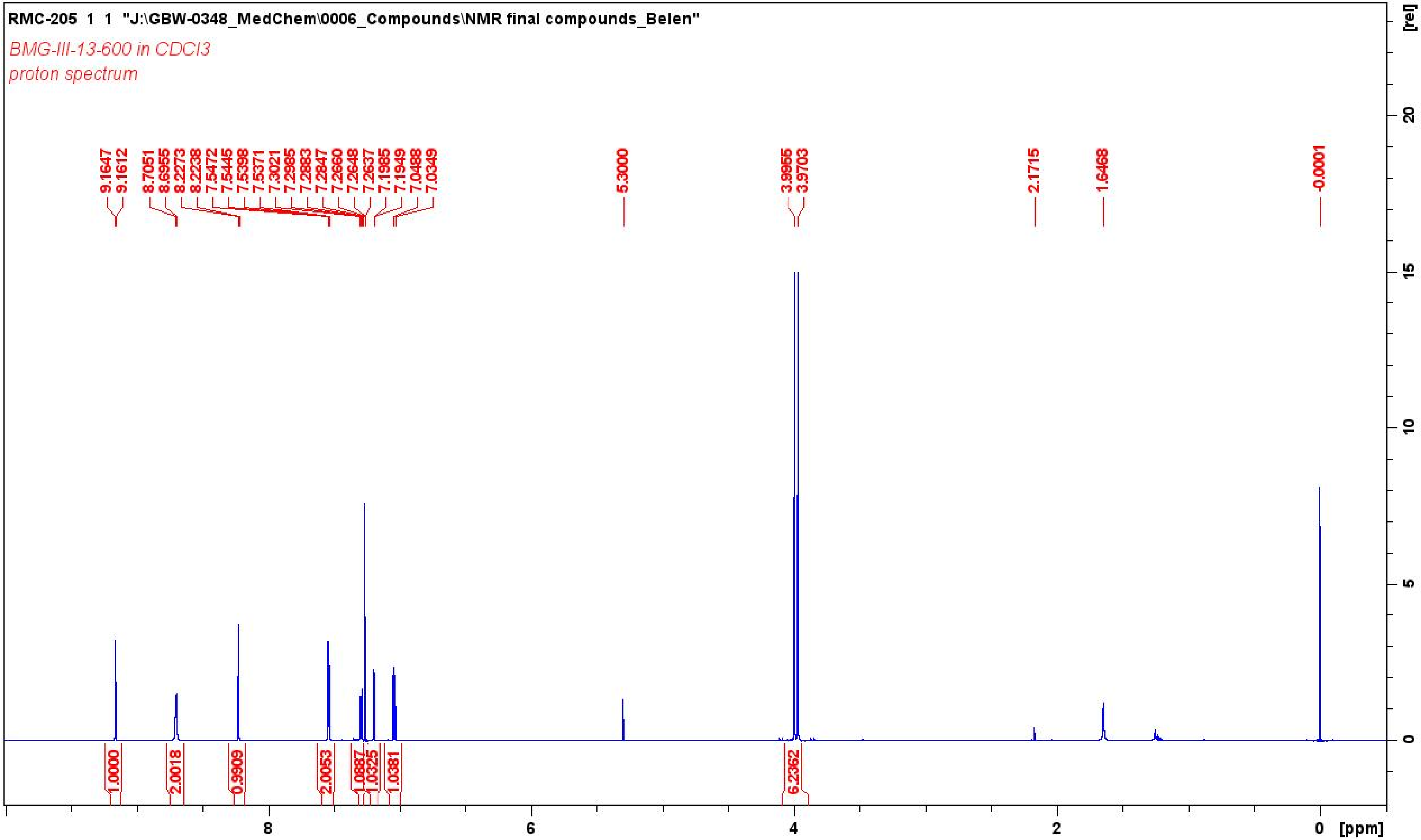

^{13}C NMR spectrum of **7x**

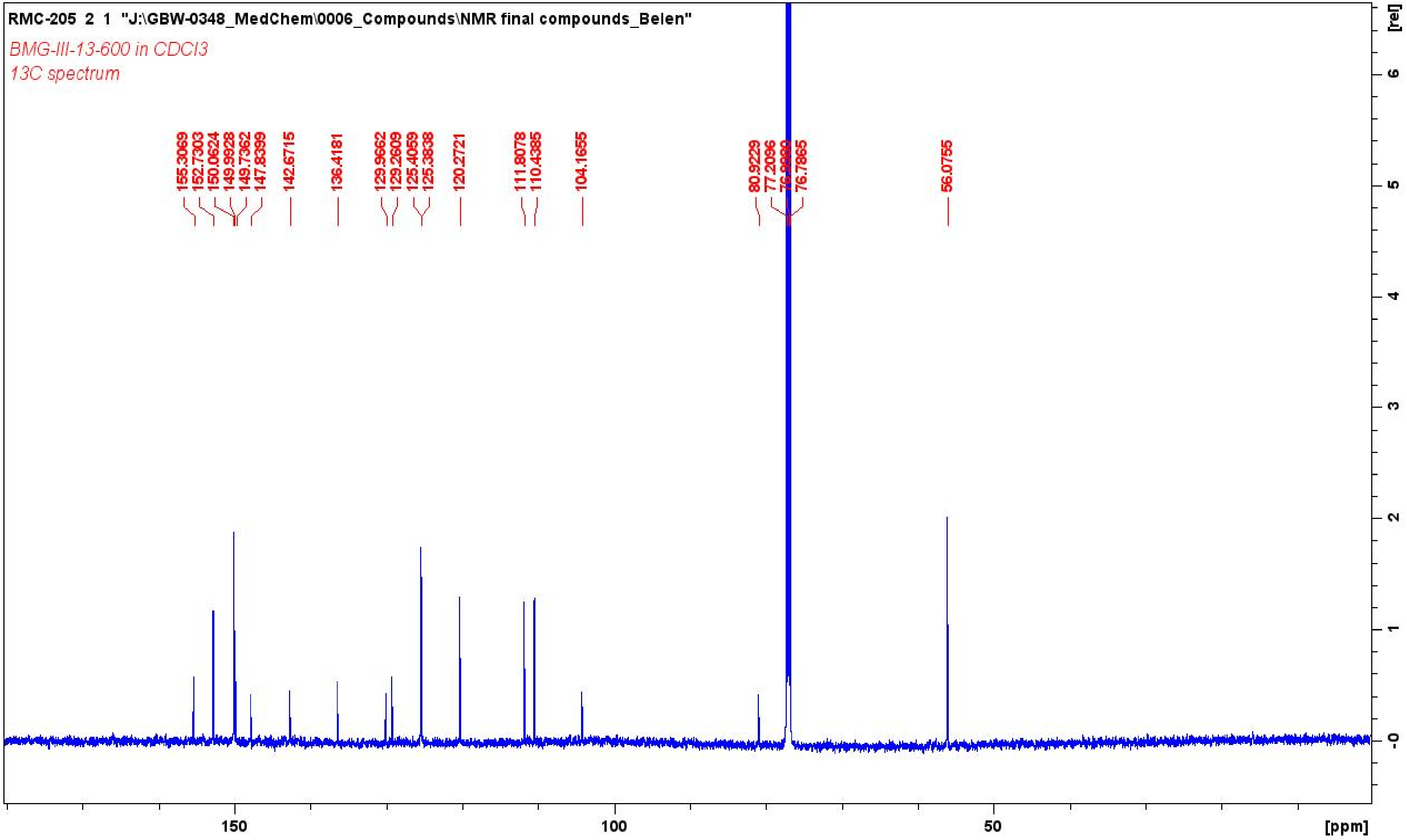

^{1}H NMR spectrum of **7y**

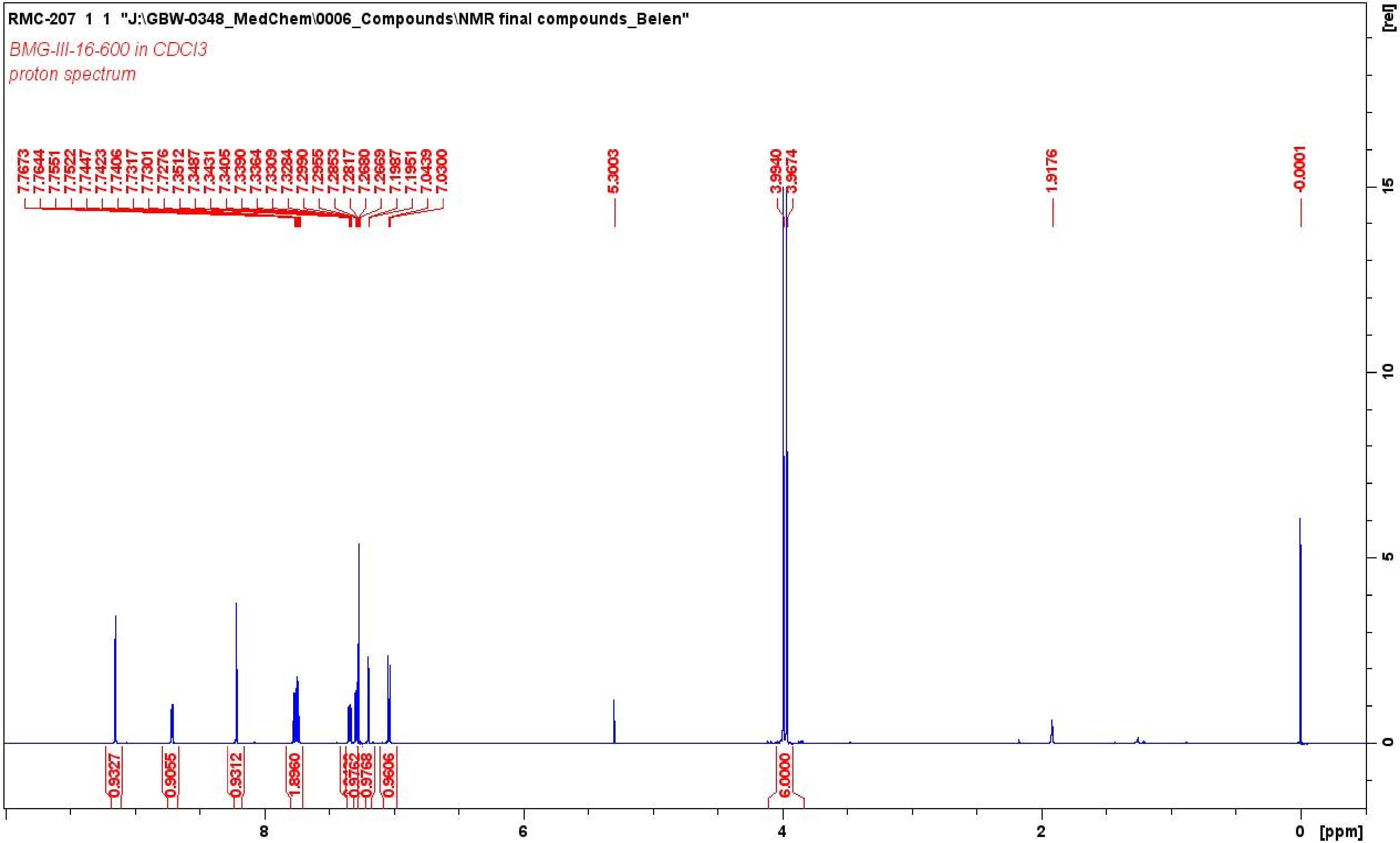

^{13}C NMR spectrum of **7y**

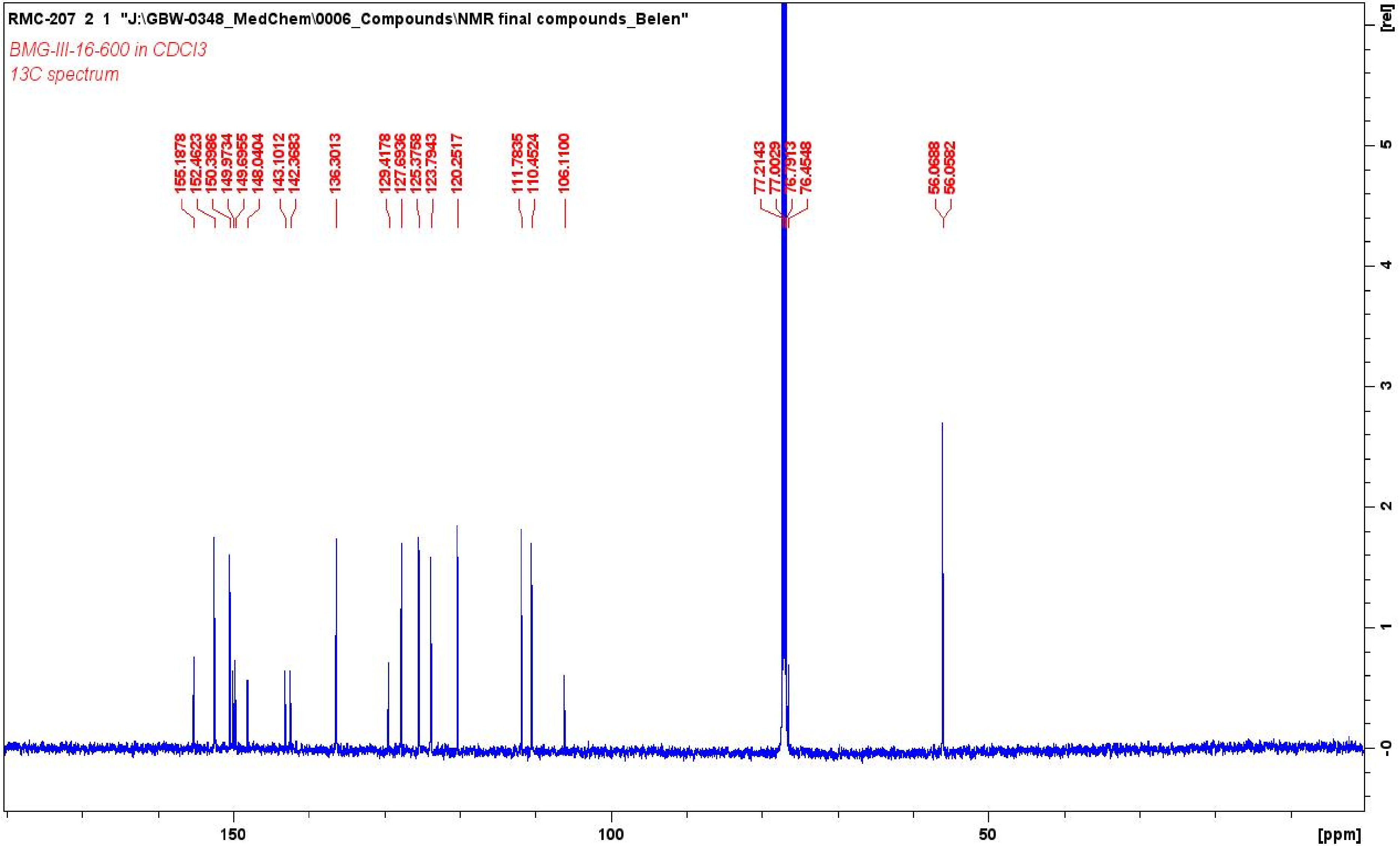

^{1}H NMR spectrum of **11a**

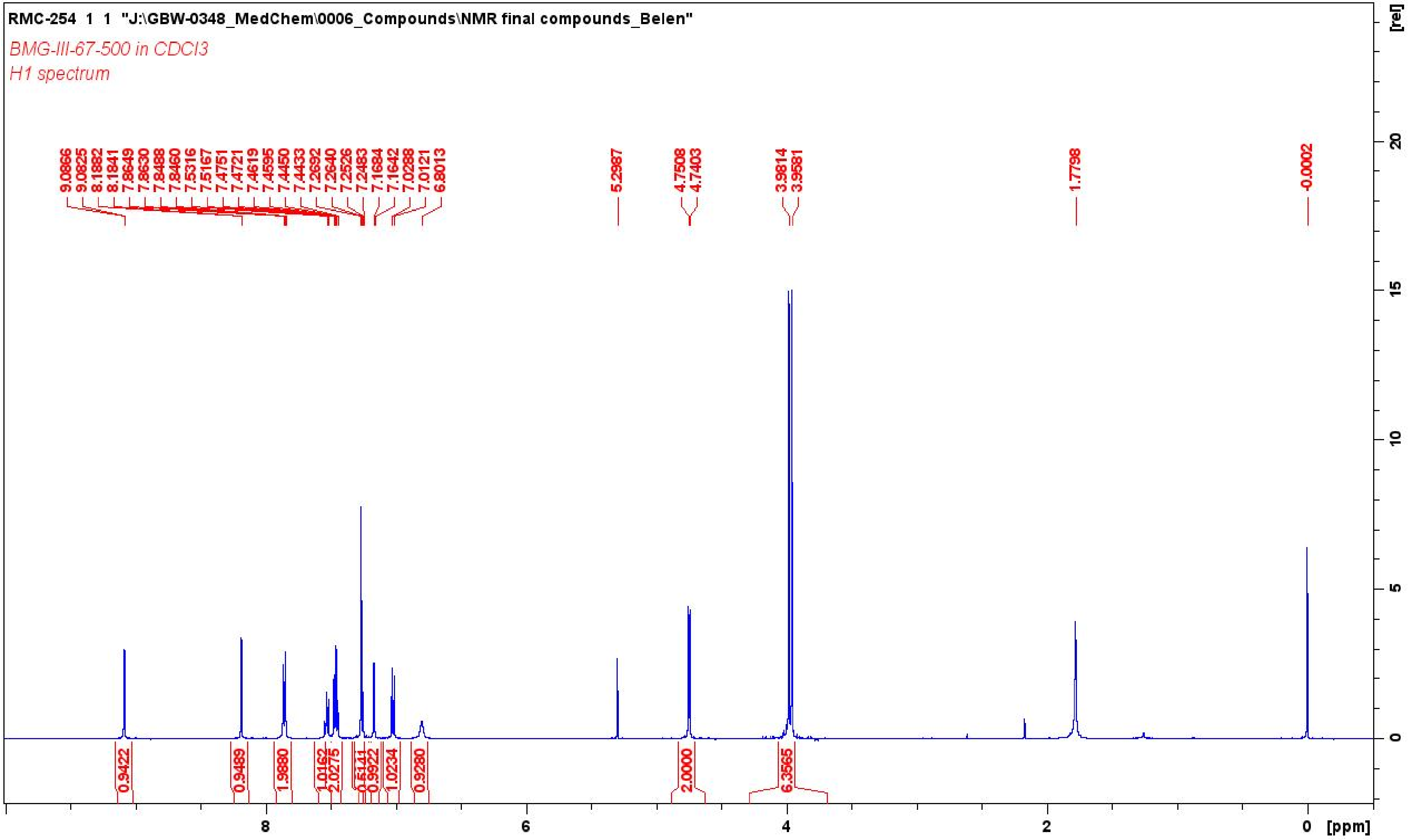

^{13}C NMR spectrum of **11a**

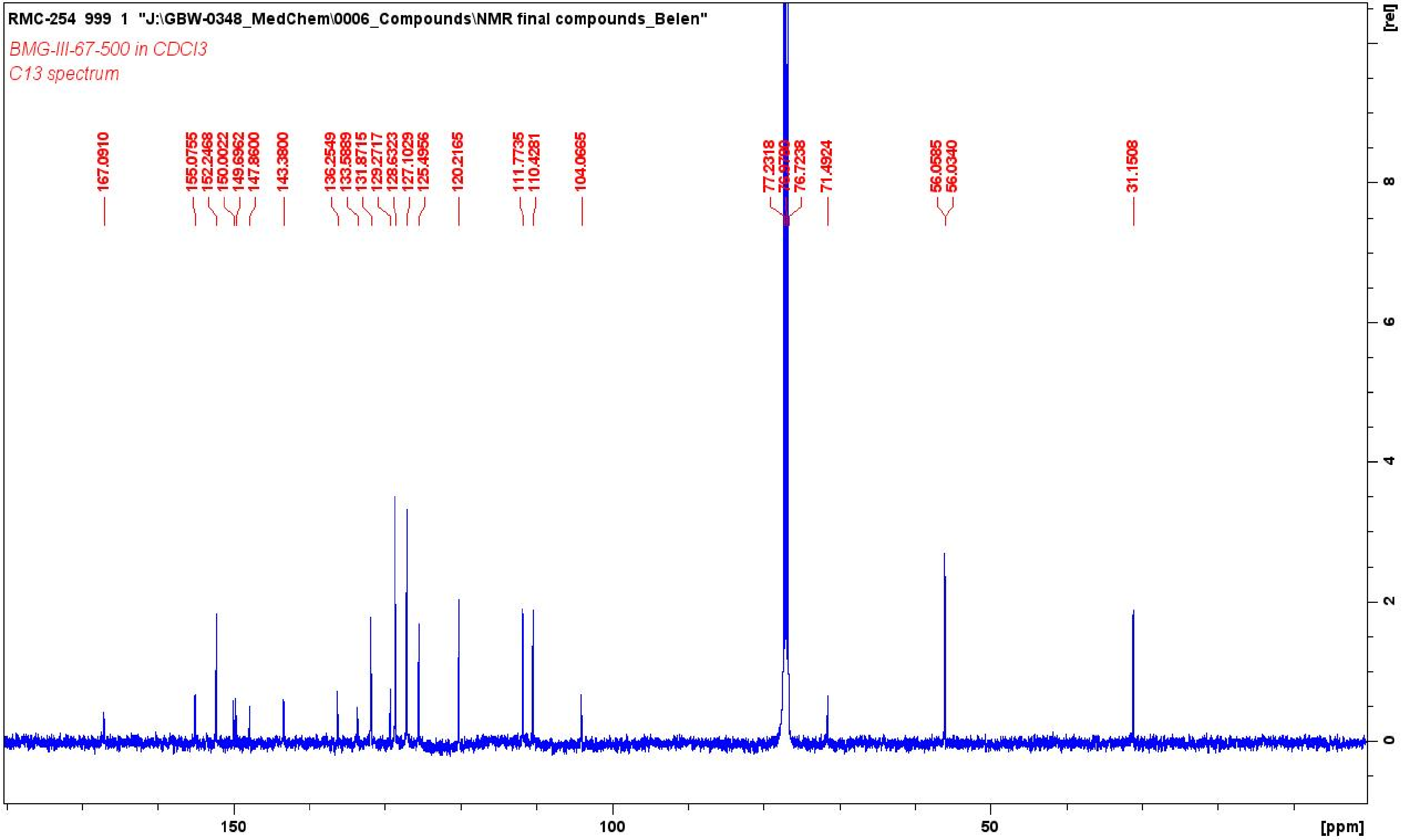

^{1}H NMR spectrum of **11b**

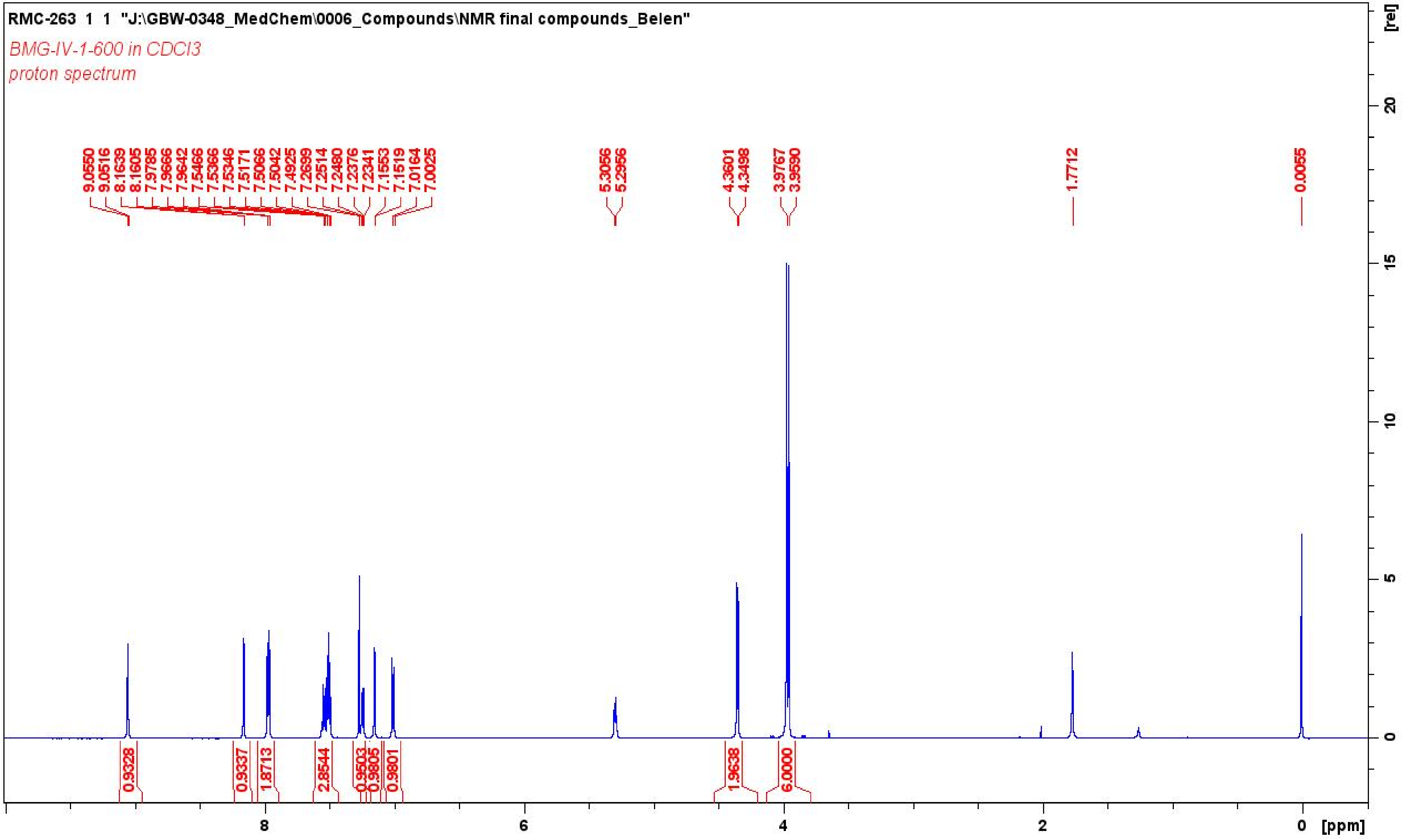

^{13}C NMR spectrum of **11b**

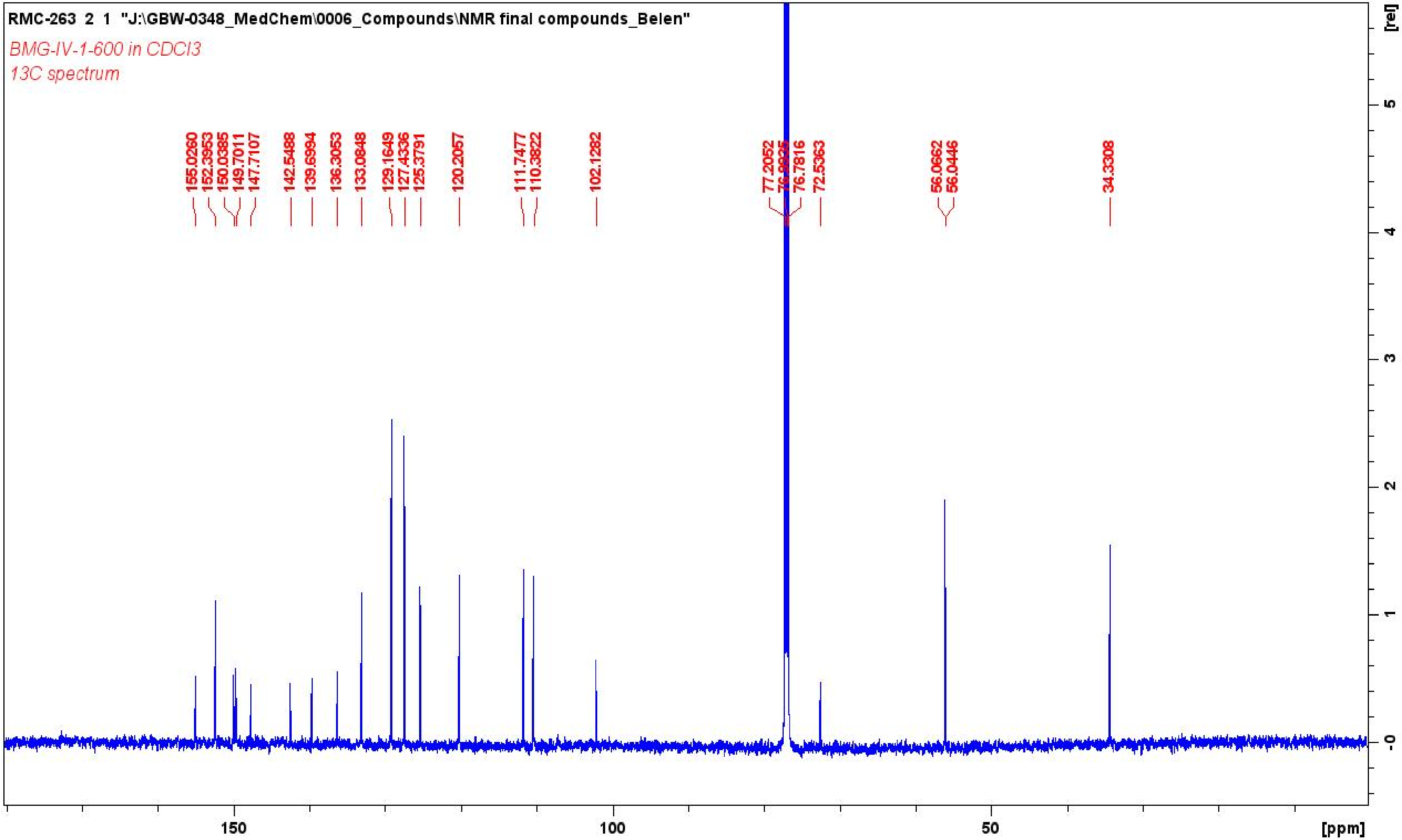

^{1}H NMR spectrum of **11c**

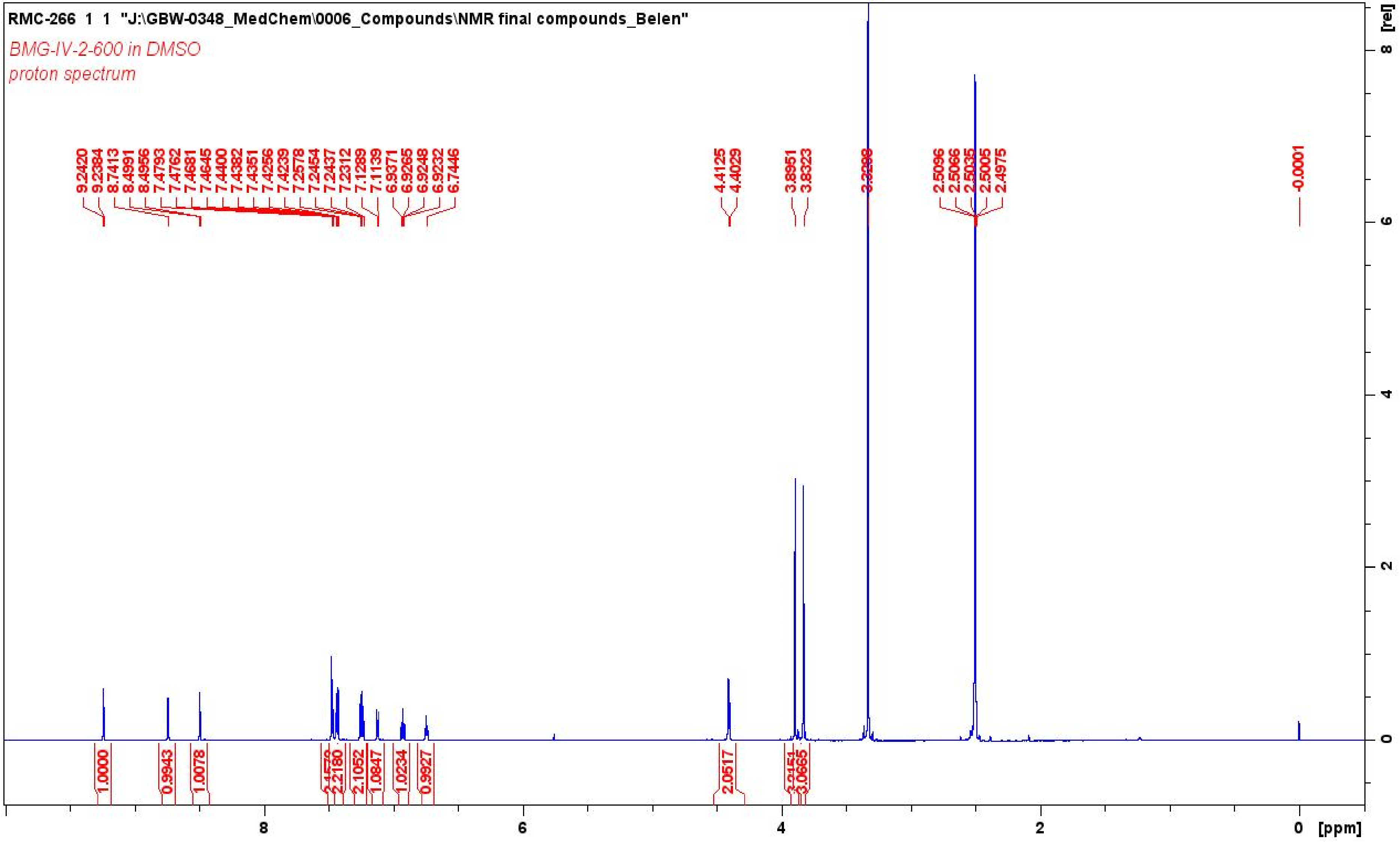

^{13}C NMR spectrum of **11c**

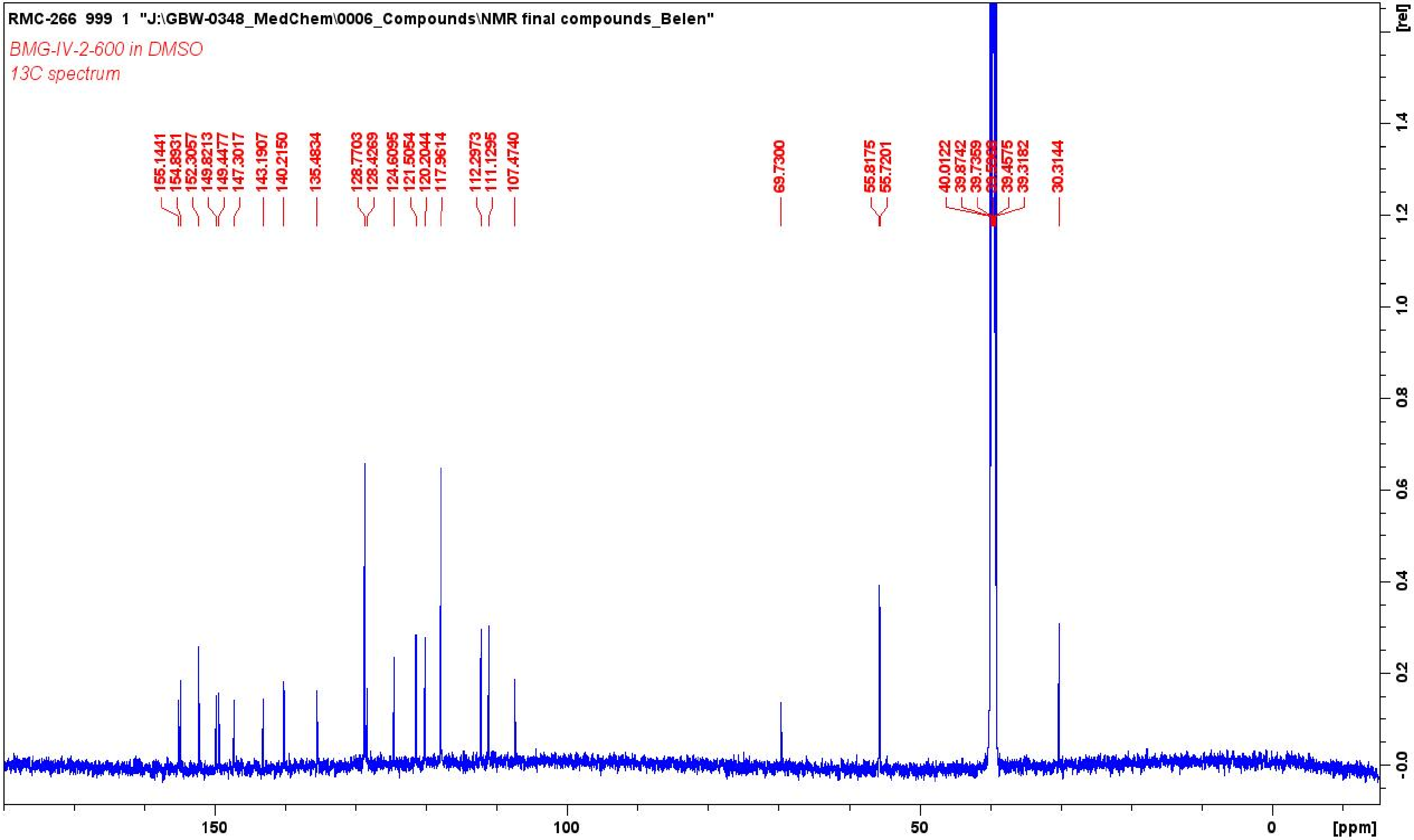

^{1}H NMR spectrum of **13**

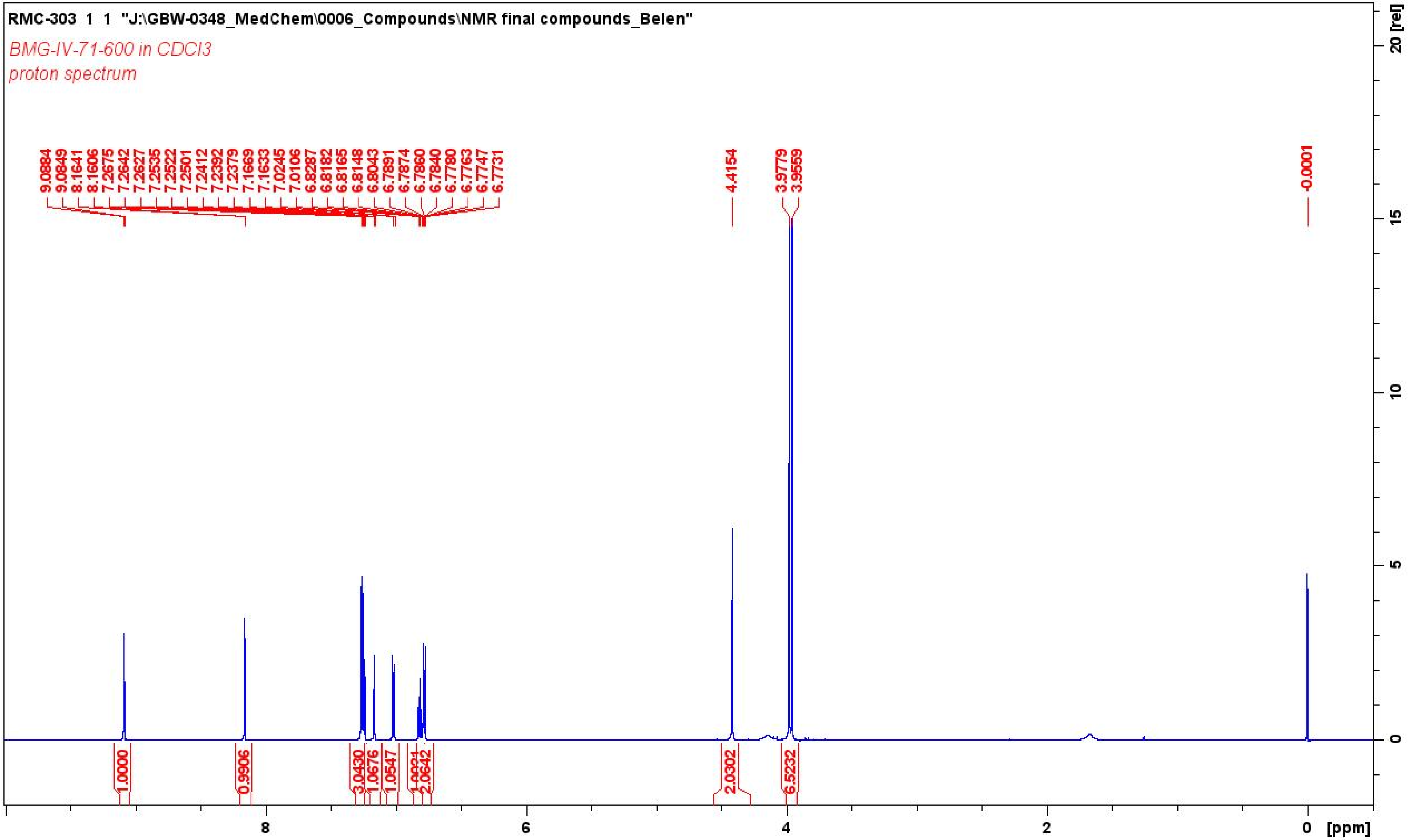

^{13}C NMR spectrum of **13**

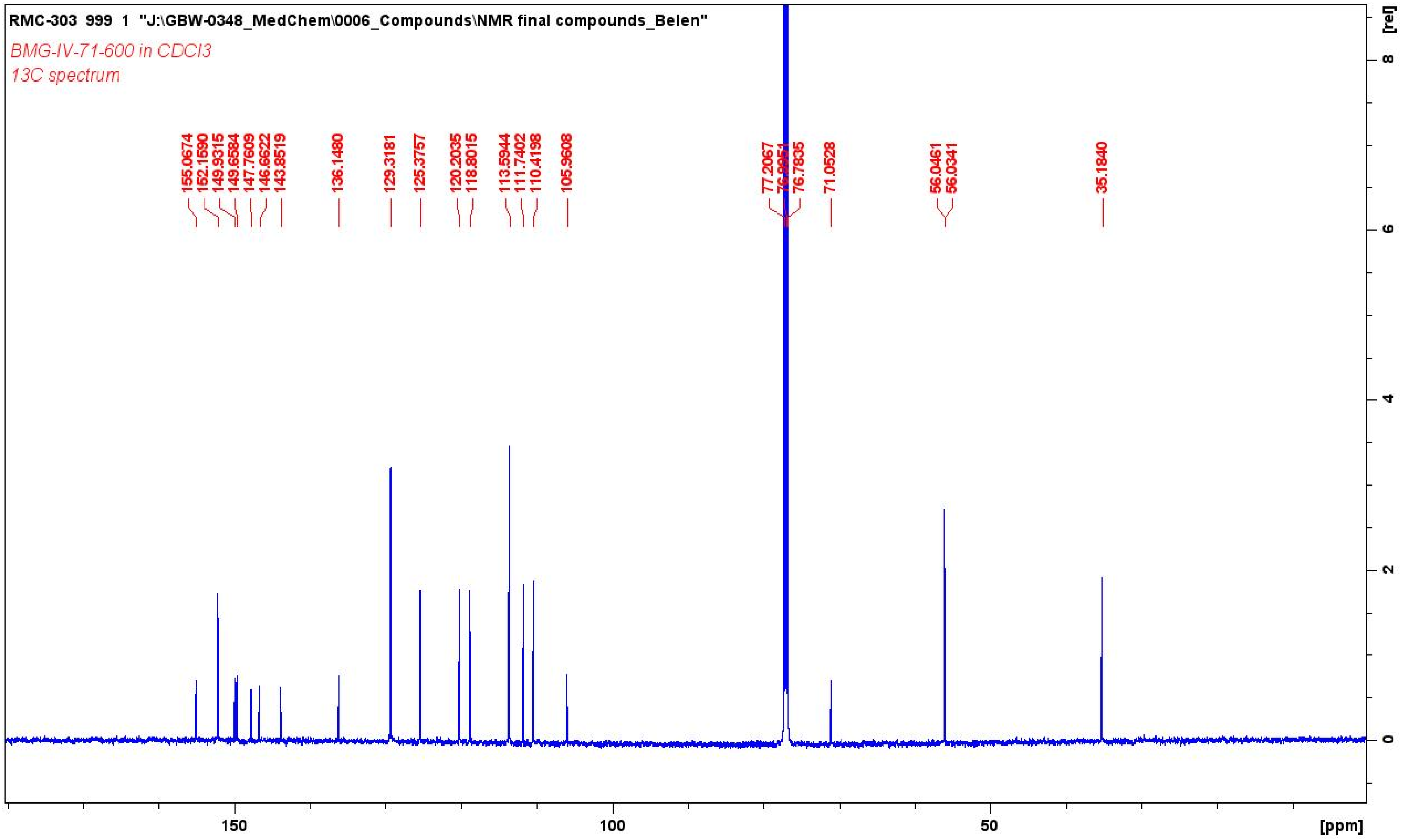

^{1}H NMR spectrum of **15**

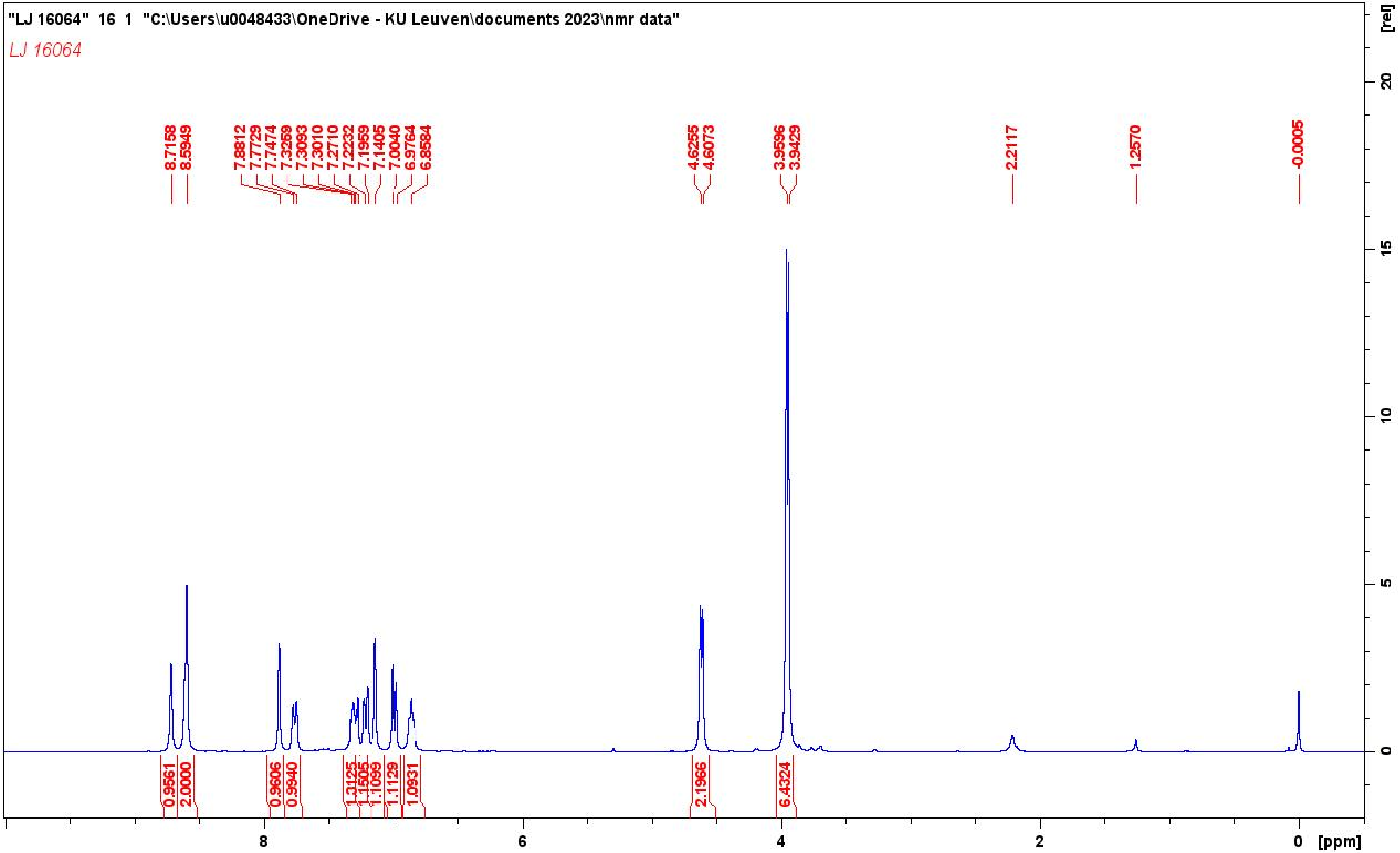

^{13}C NMR spectrum of **15**

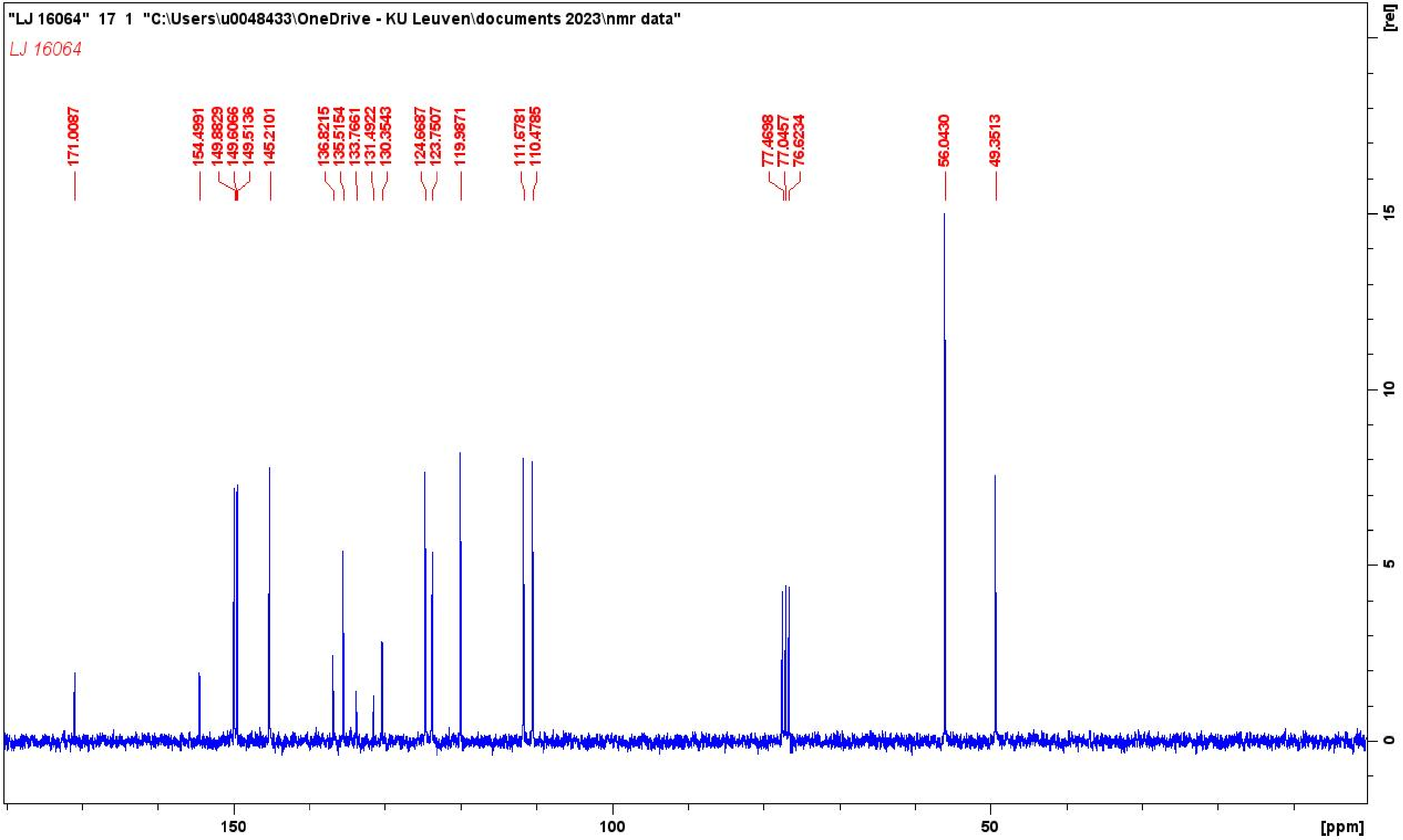

^{1}H NMR spectrum of **17**

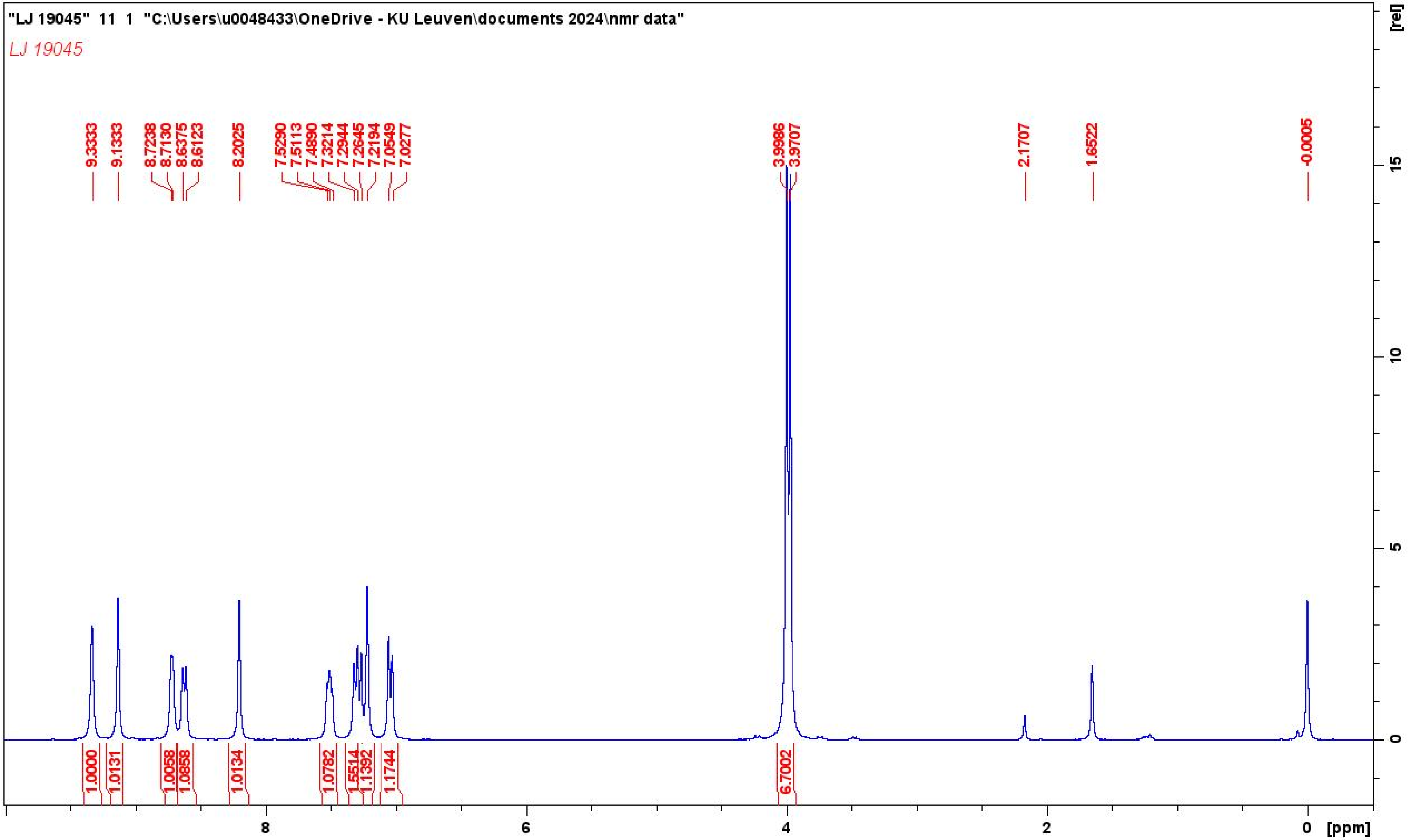

^{13}C NMR spectrum of **17**

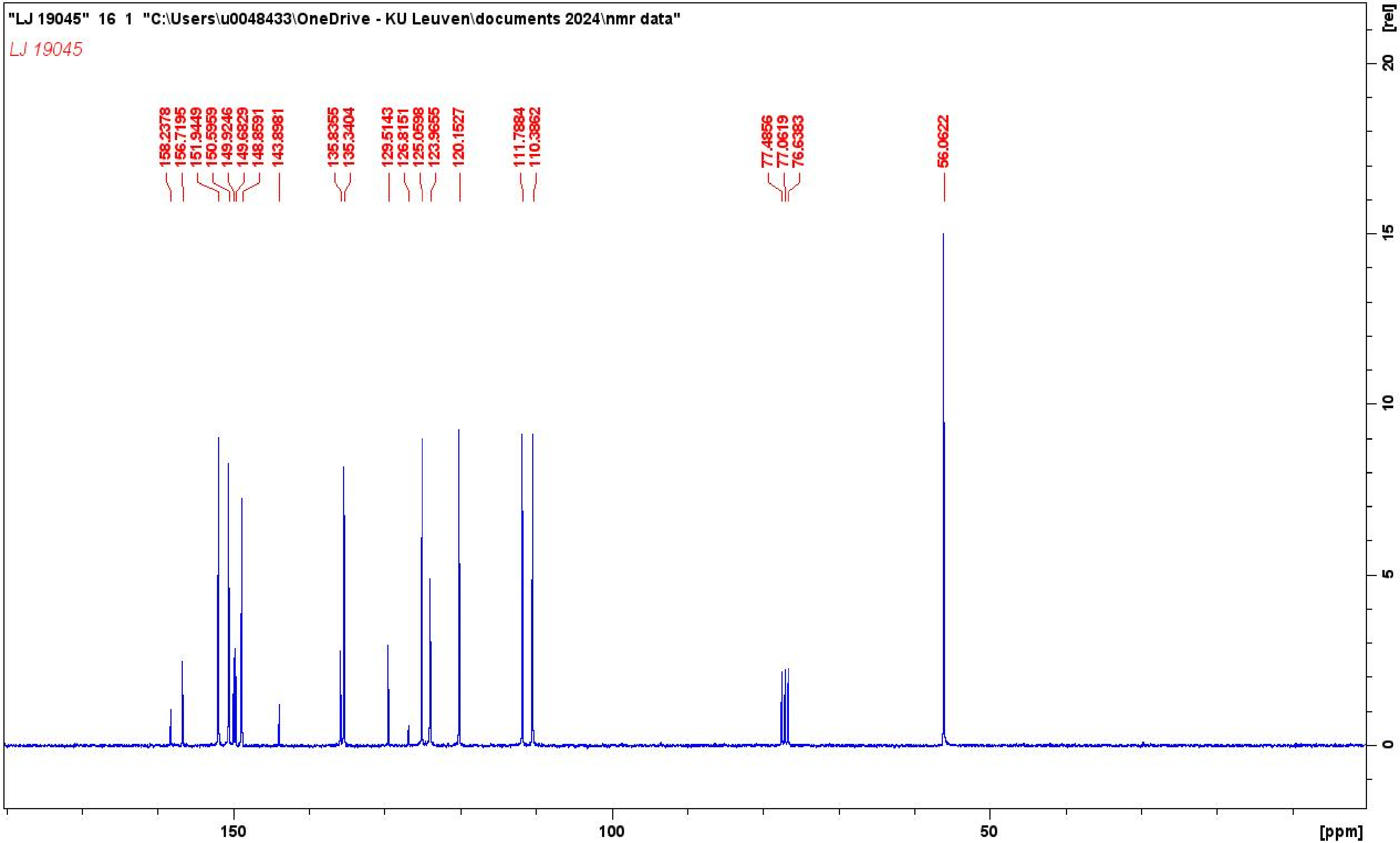

^{1}H NMR spectrum of **20**

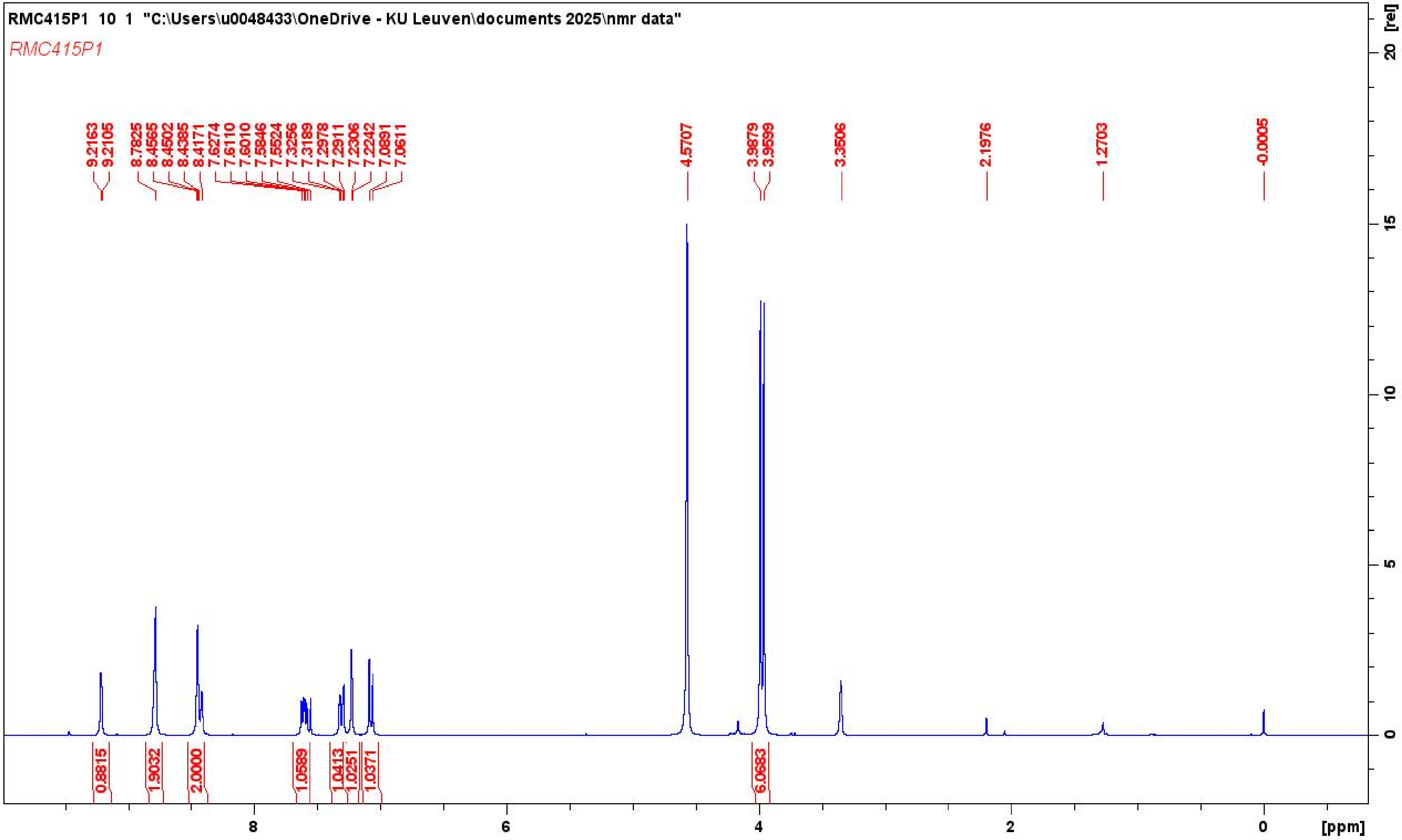

^{13}C NMR spectrum of **20**

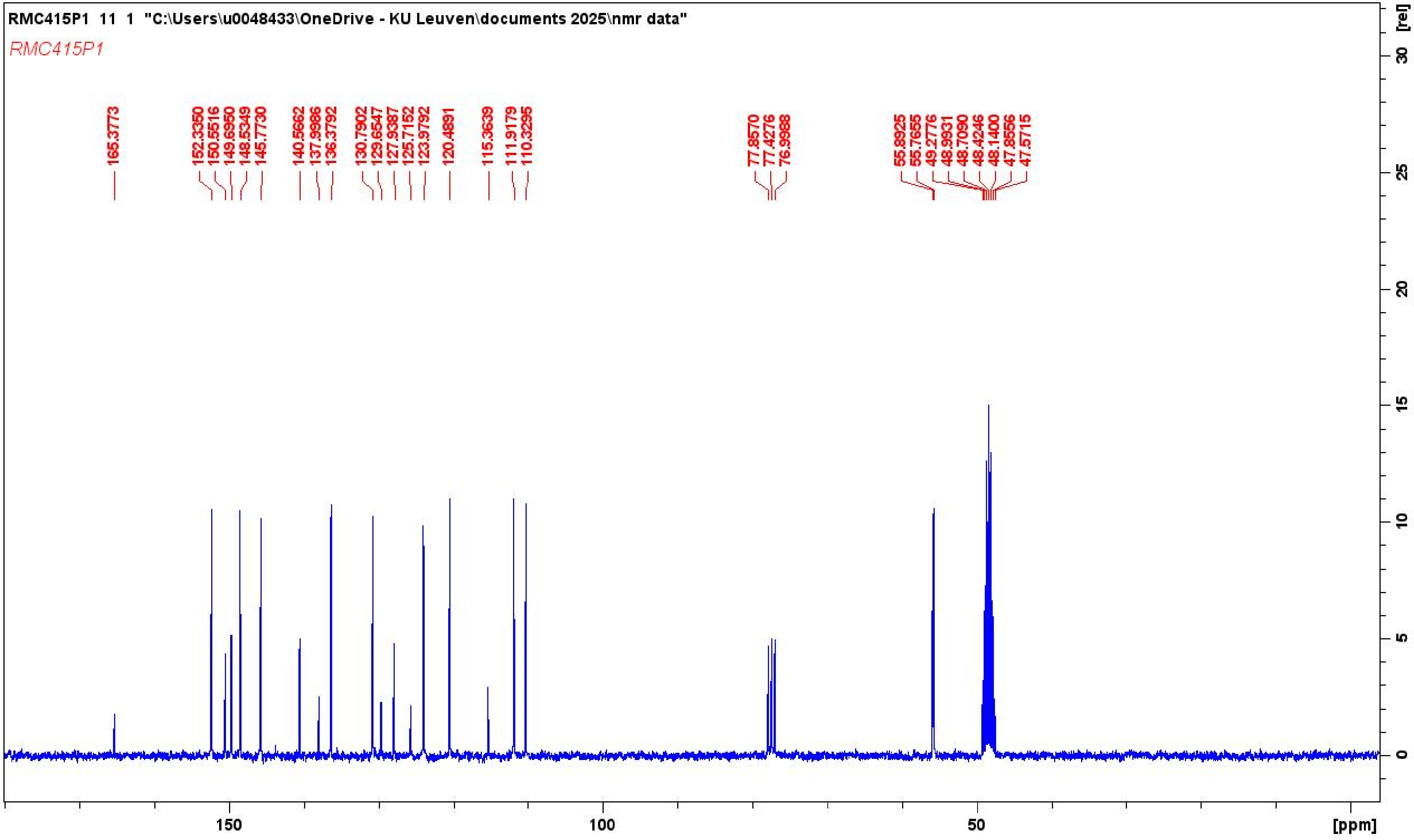

^{1}H NMR spectrum of **22**

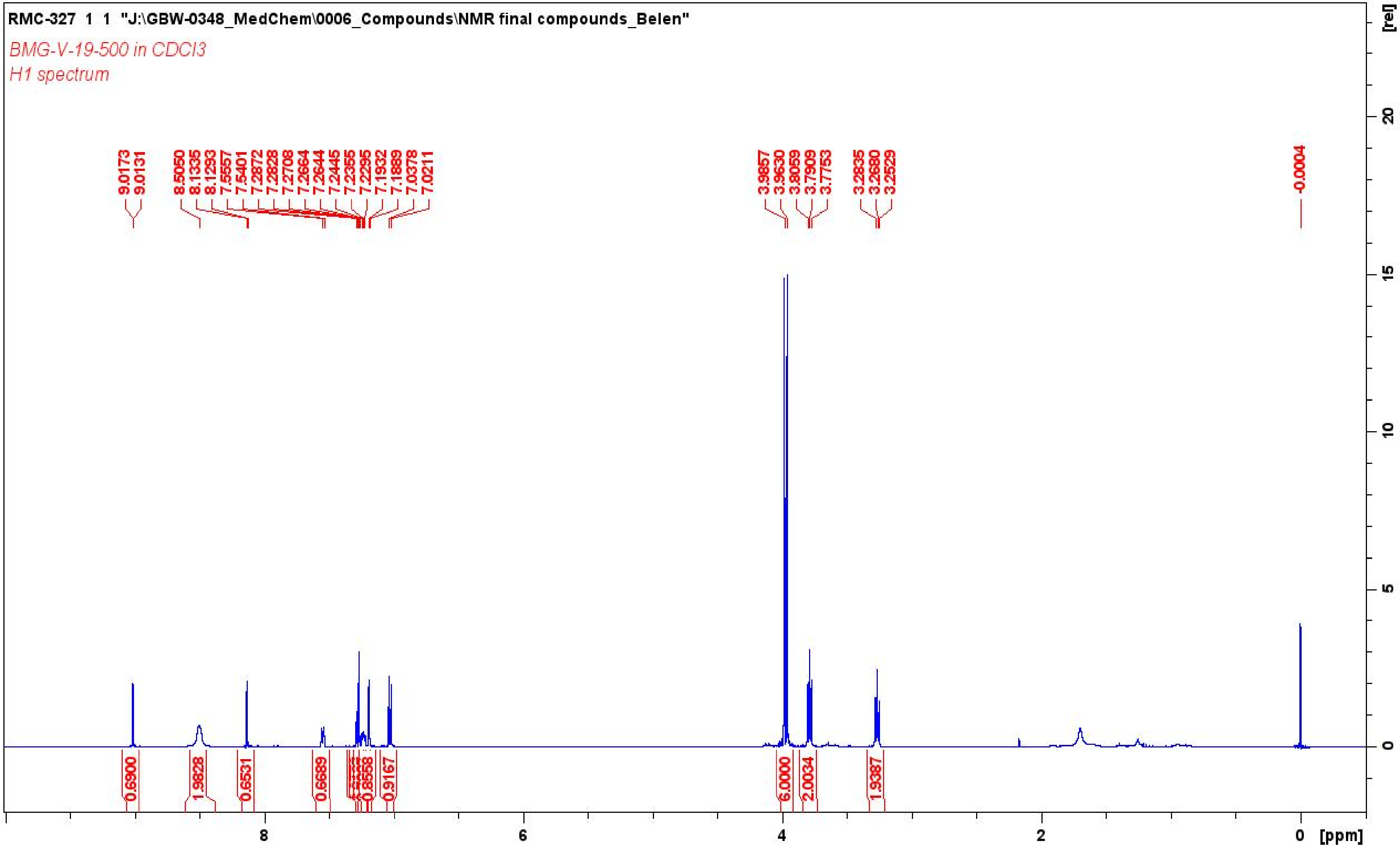

^{13}C NMR spectrum of **22**

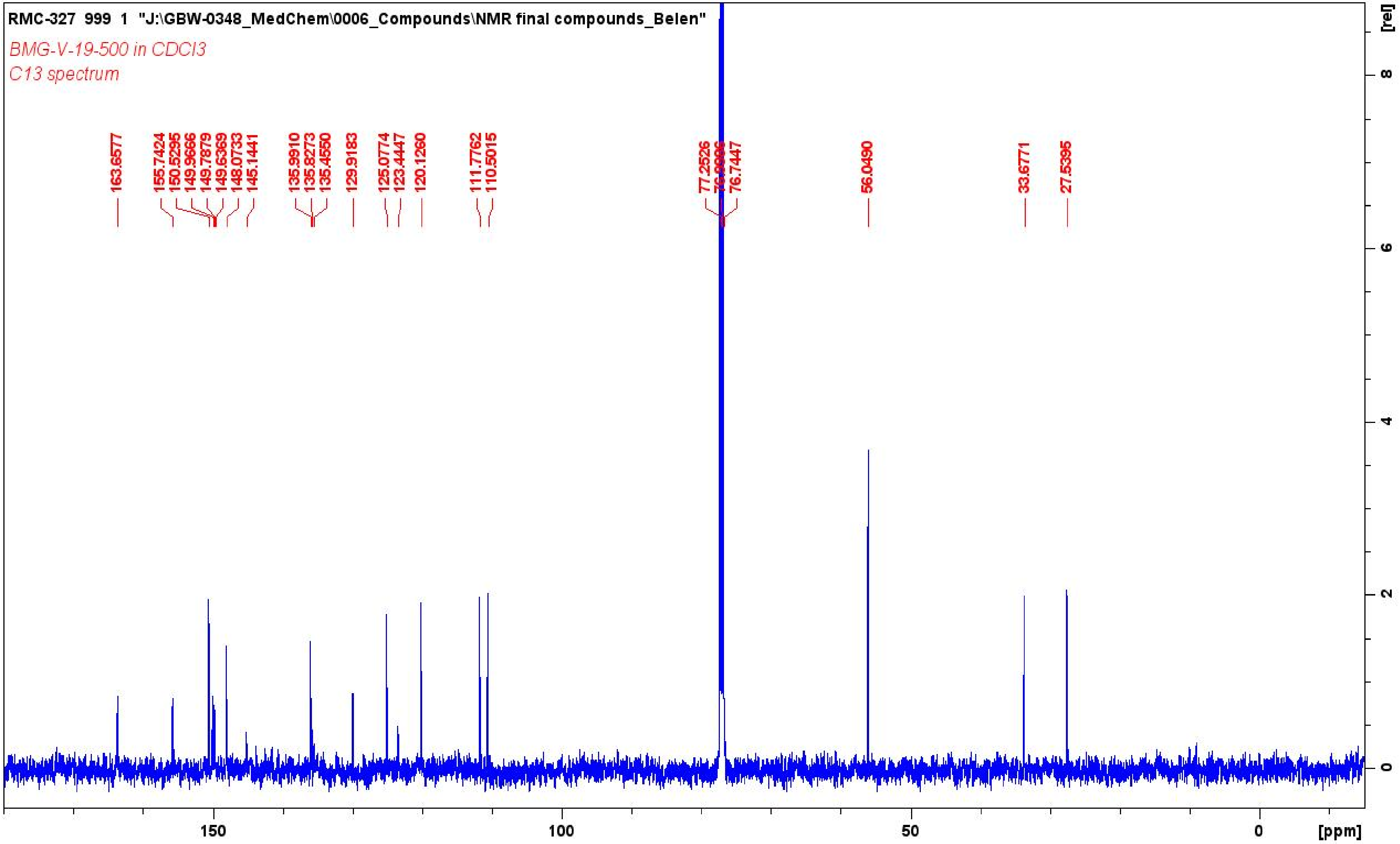

^{1}H NMR spectrum of **23a**

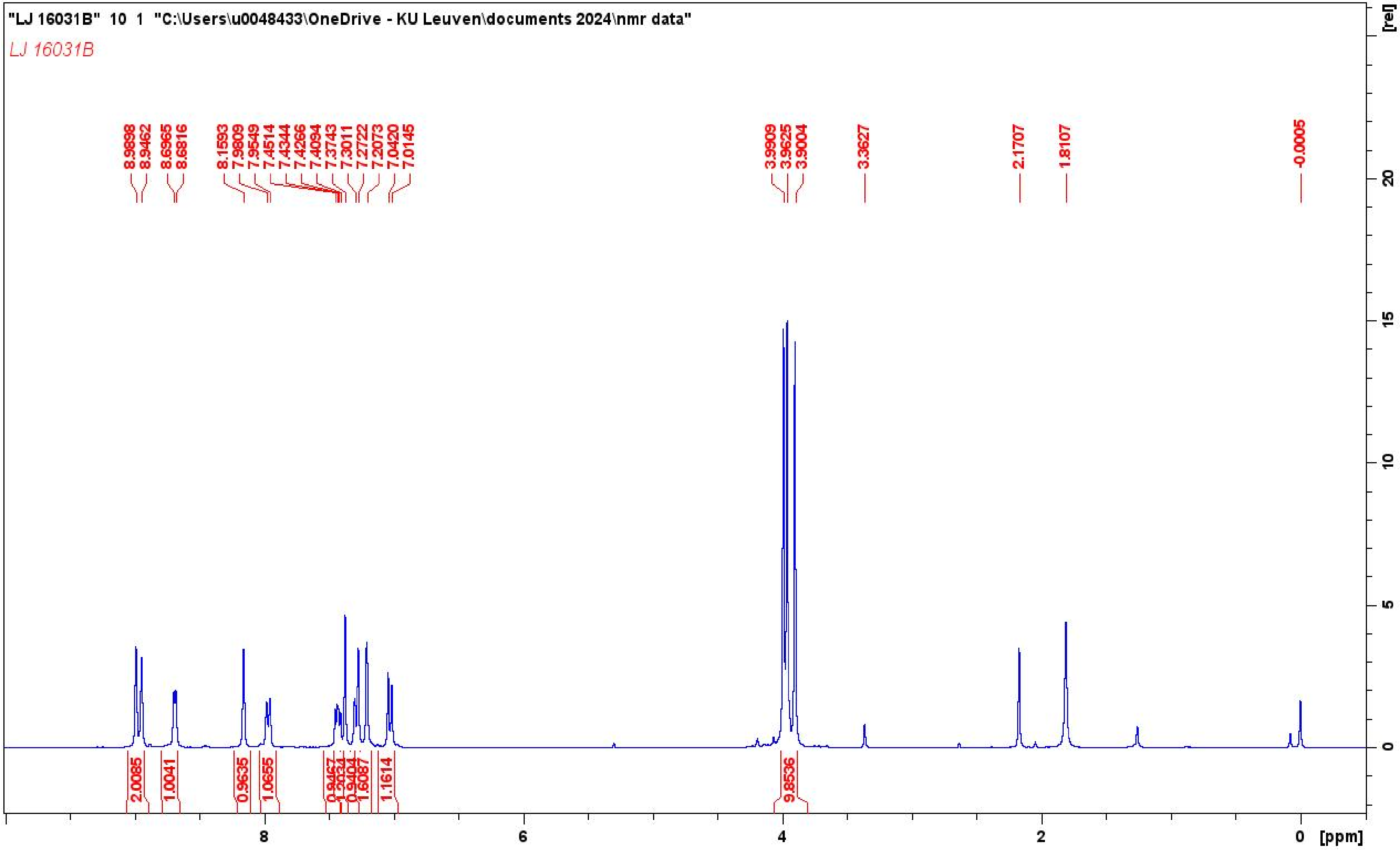

^{13}C NMR spectrum of **23a**

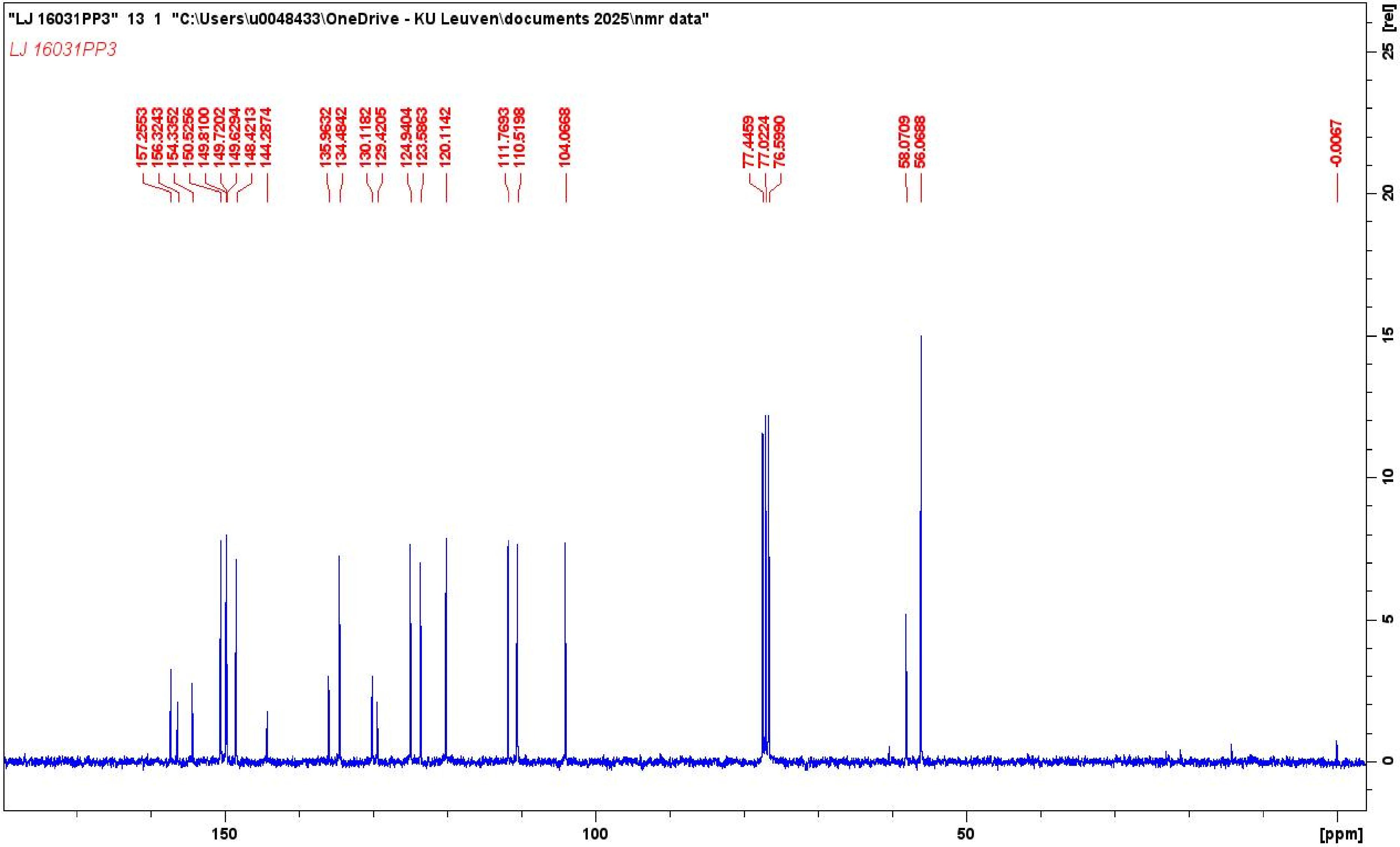

H-H COSY NMR spectrum of **23a**

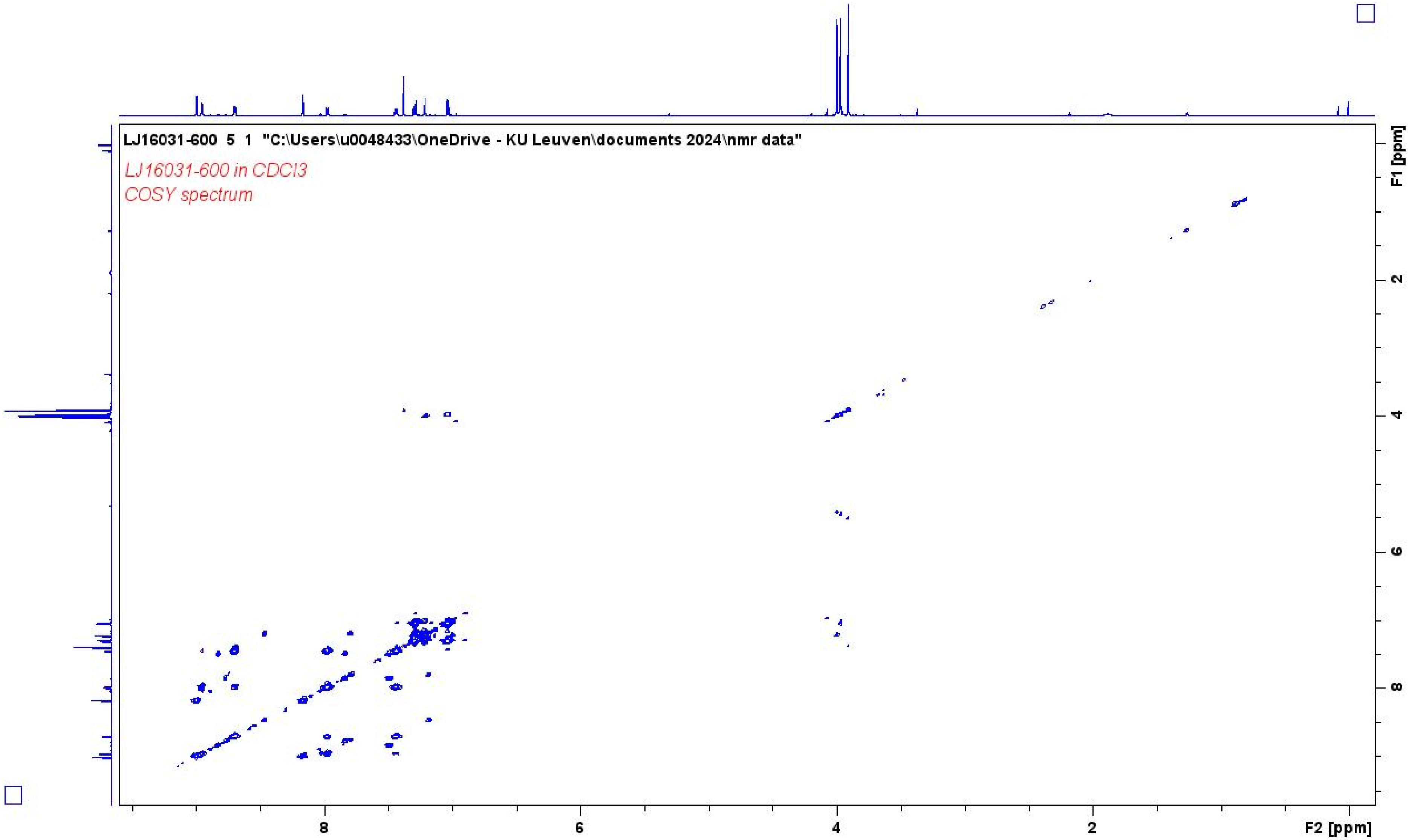

HSQC NMR spectrum of **23a**

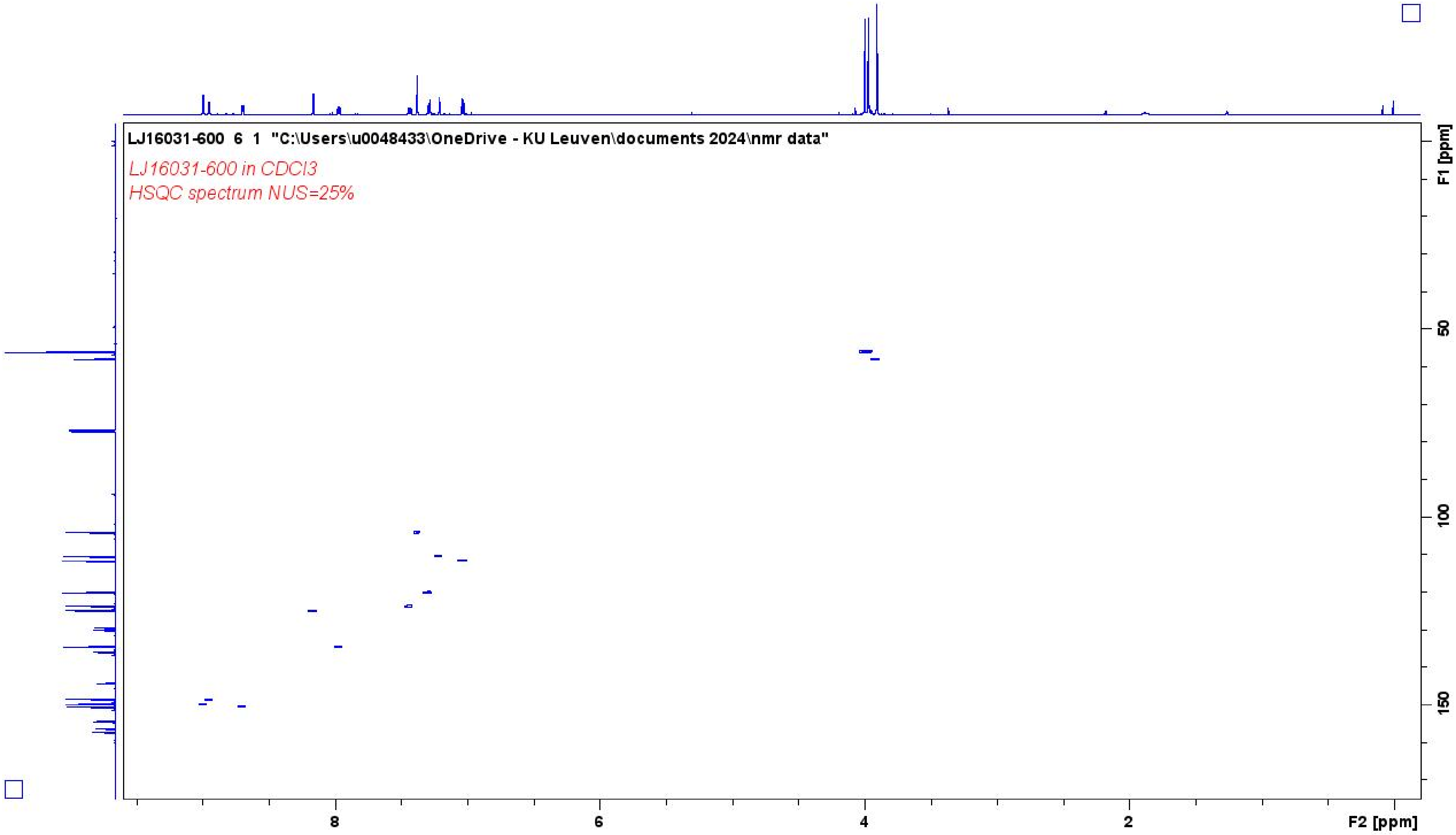

HMBC NMR spectrum of **23a**

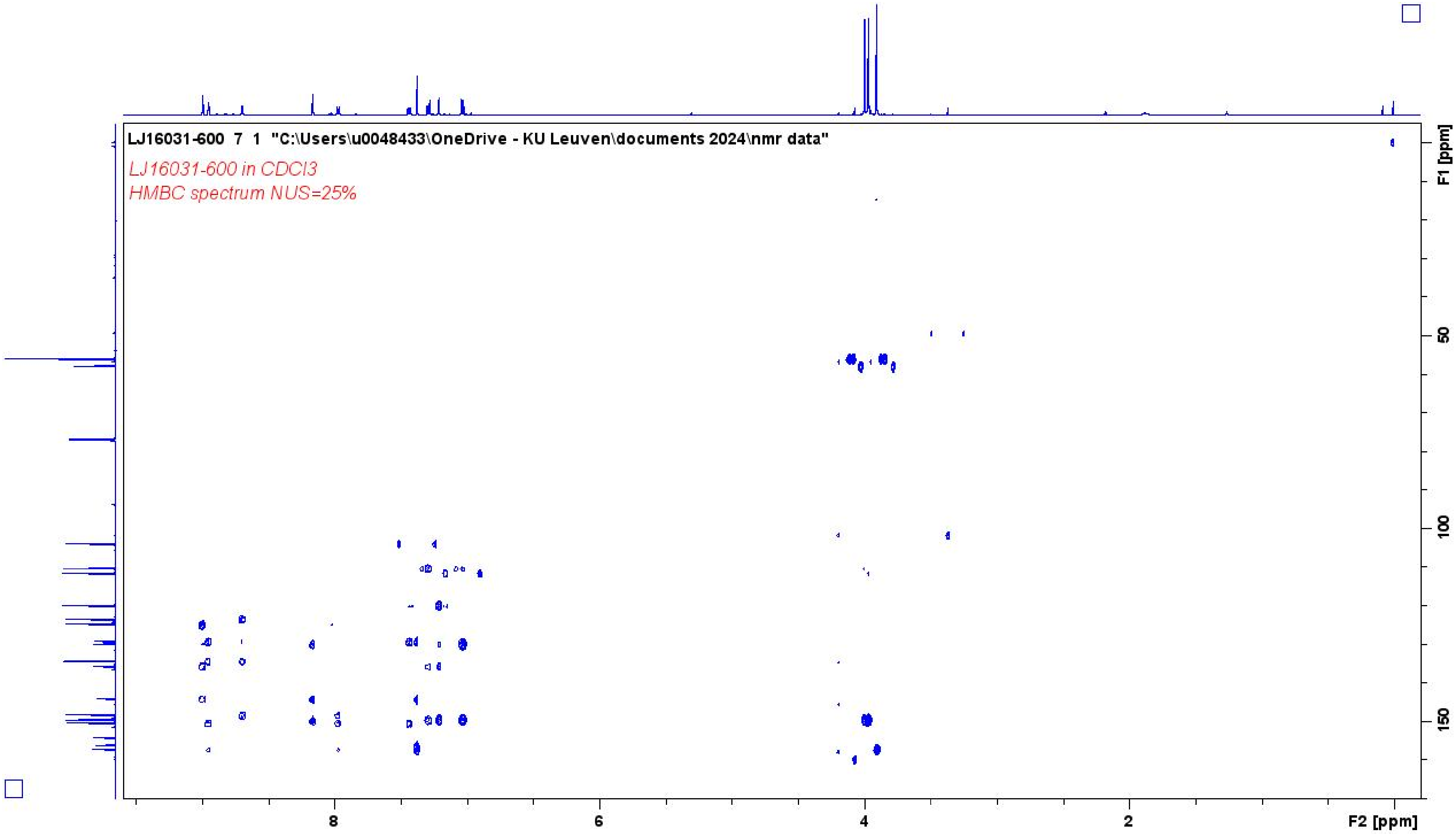

NOESY NMR spectrum of **23a**

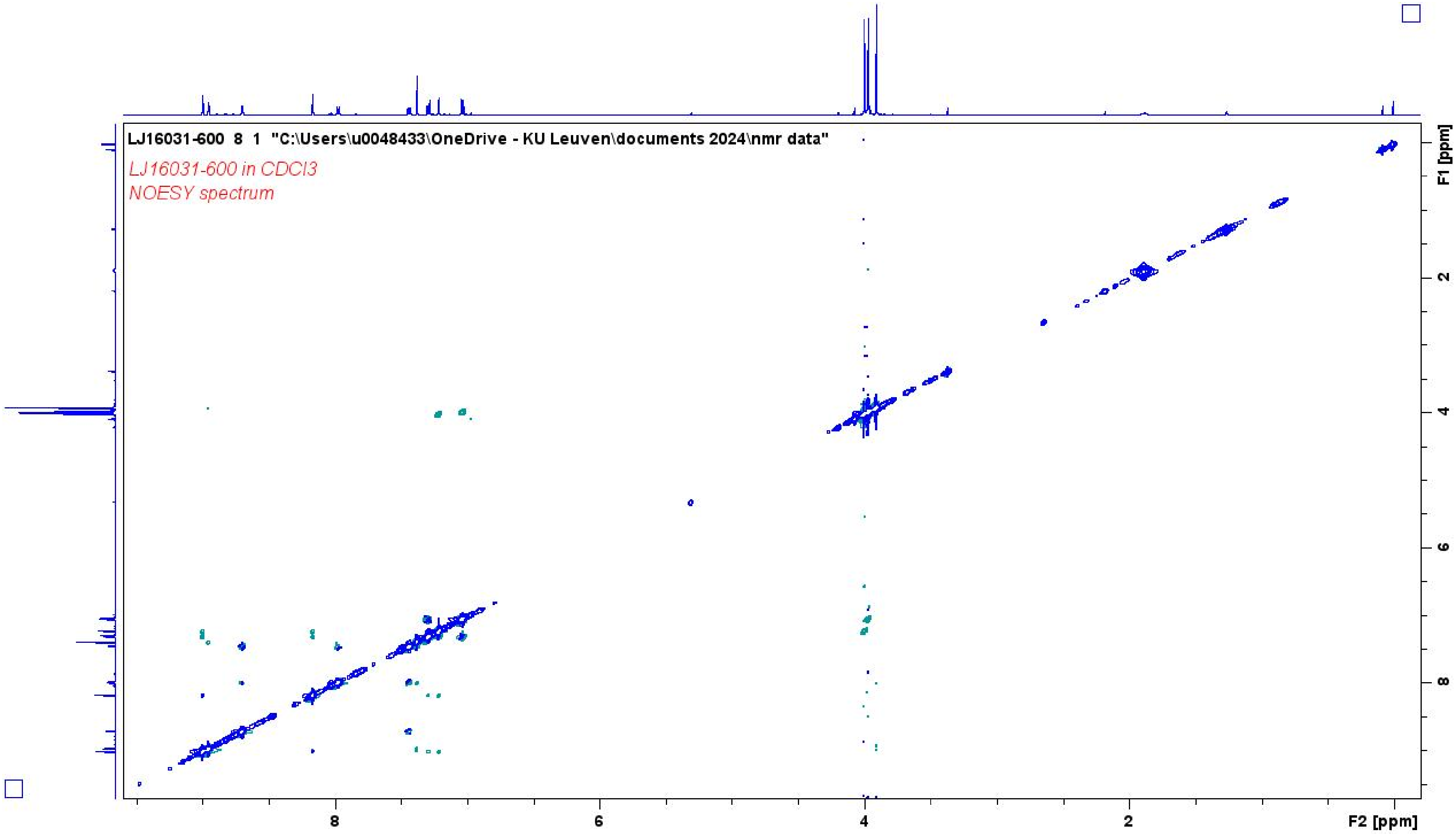

^{1}H NMR spectrum of **23b**

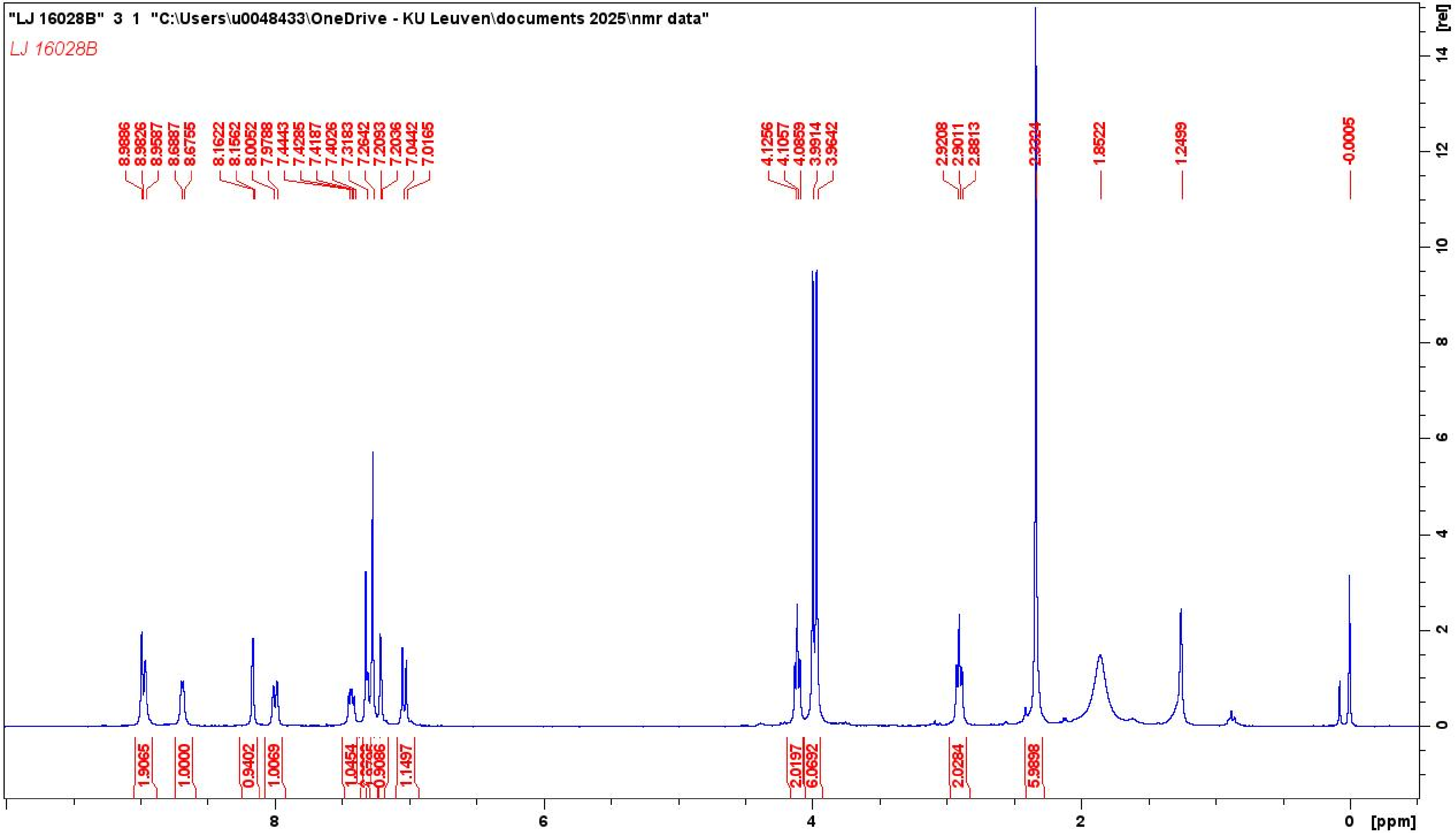

^{13}C NMR spectrum of **23b**

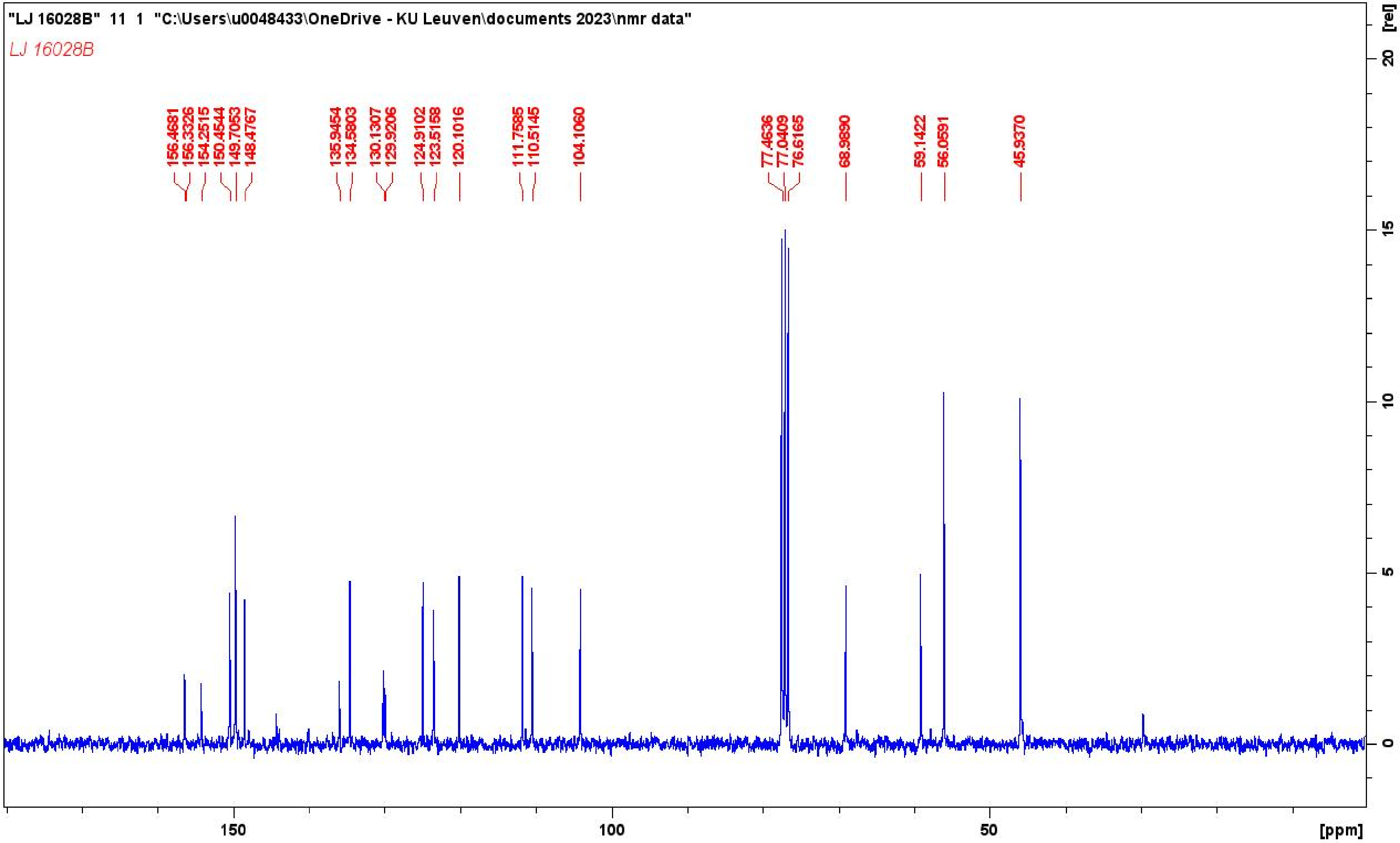

^{1}H NMR spectrum of **23c**

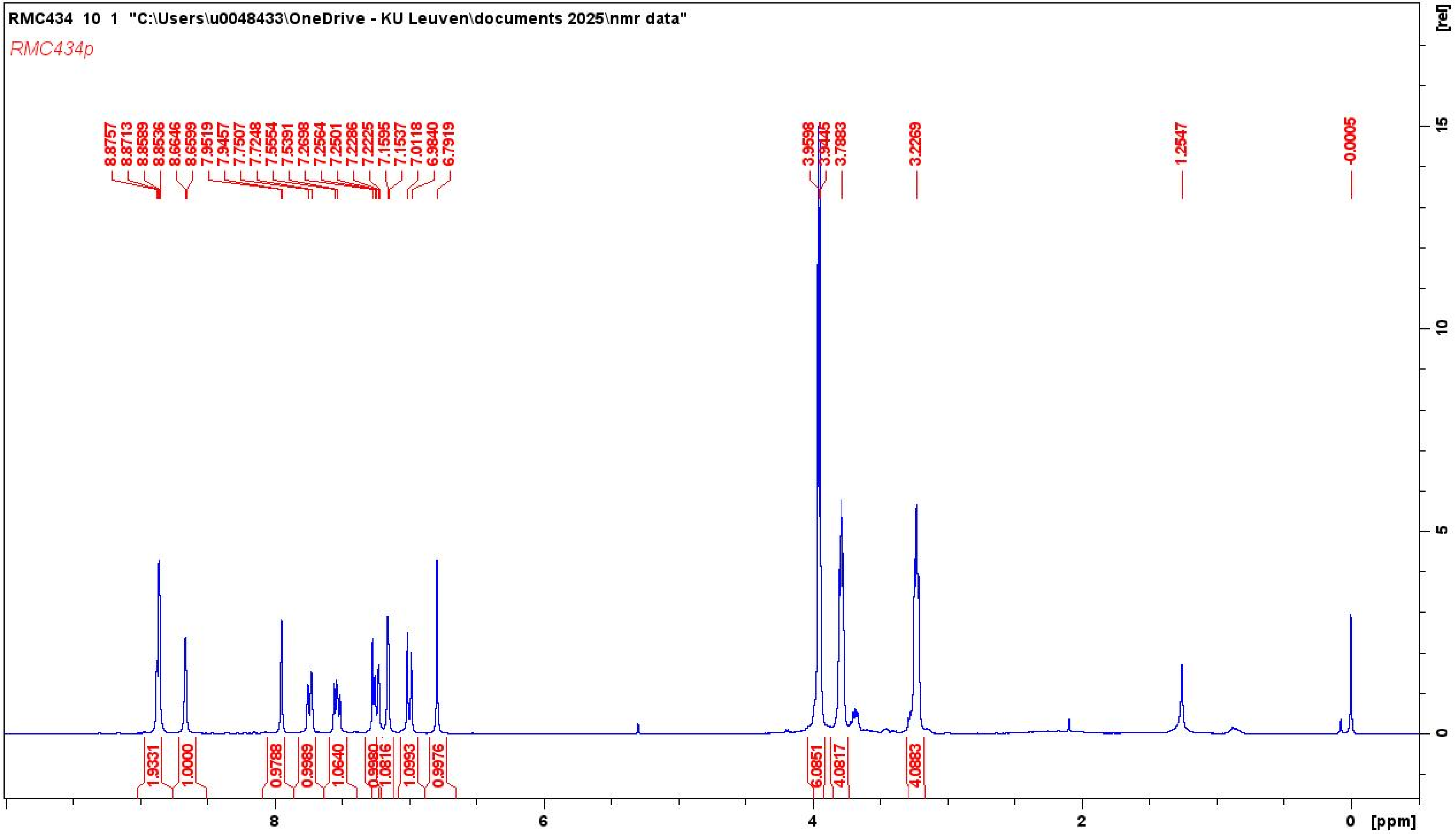

^{13}C NMR spectrum of **23c**

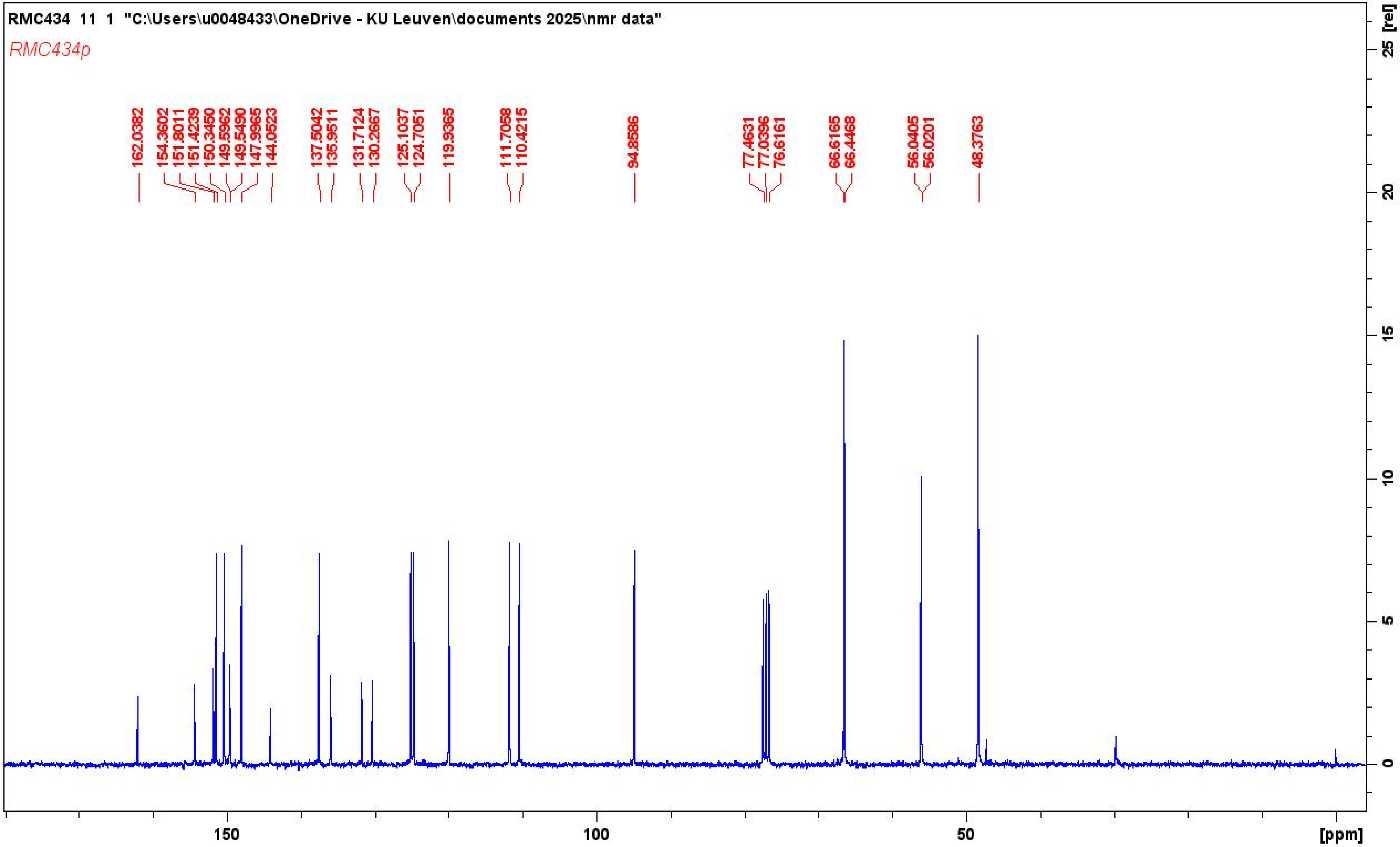

^{1}H NMR spectrum of **23d**

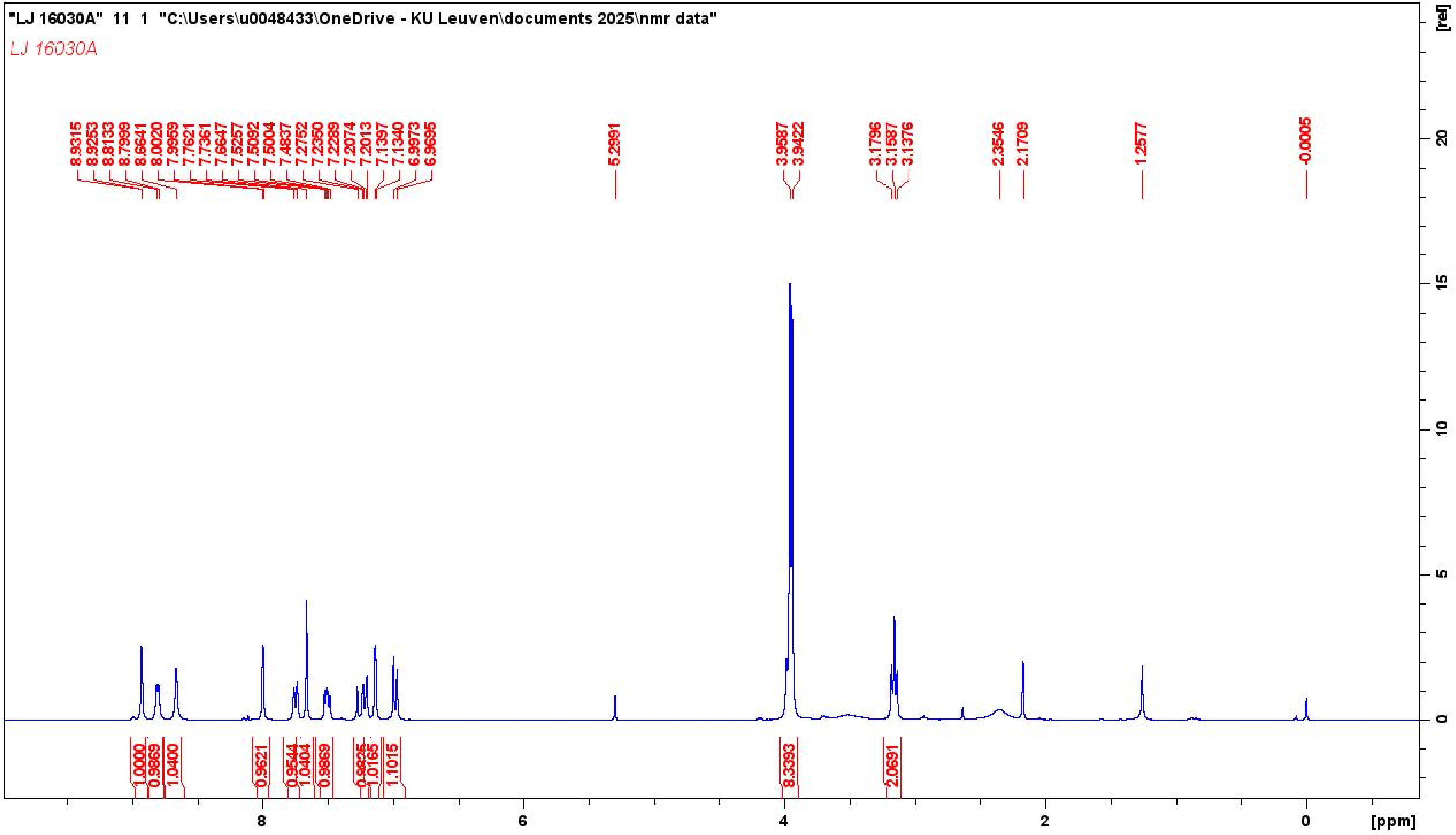

^{13}C NMR spectrum of **23d**

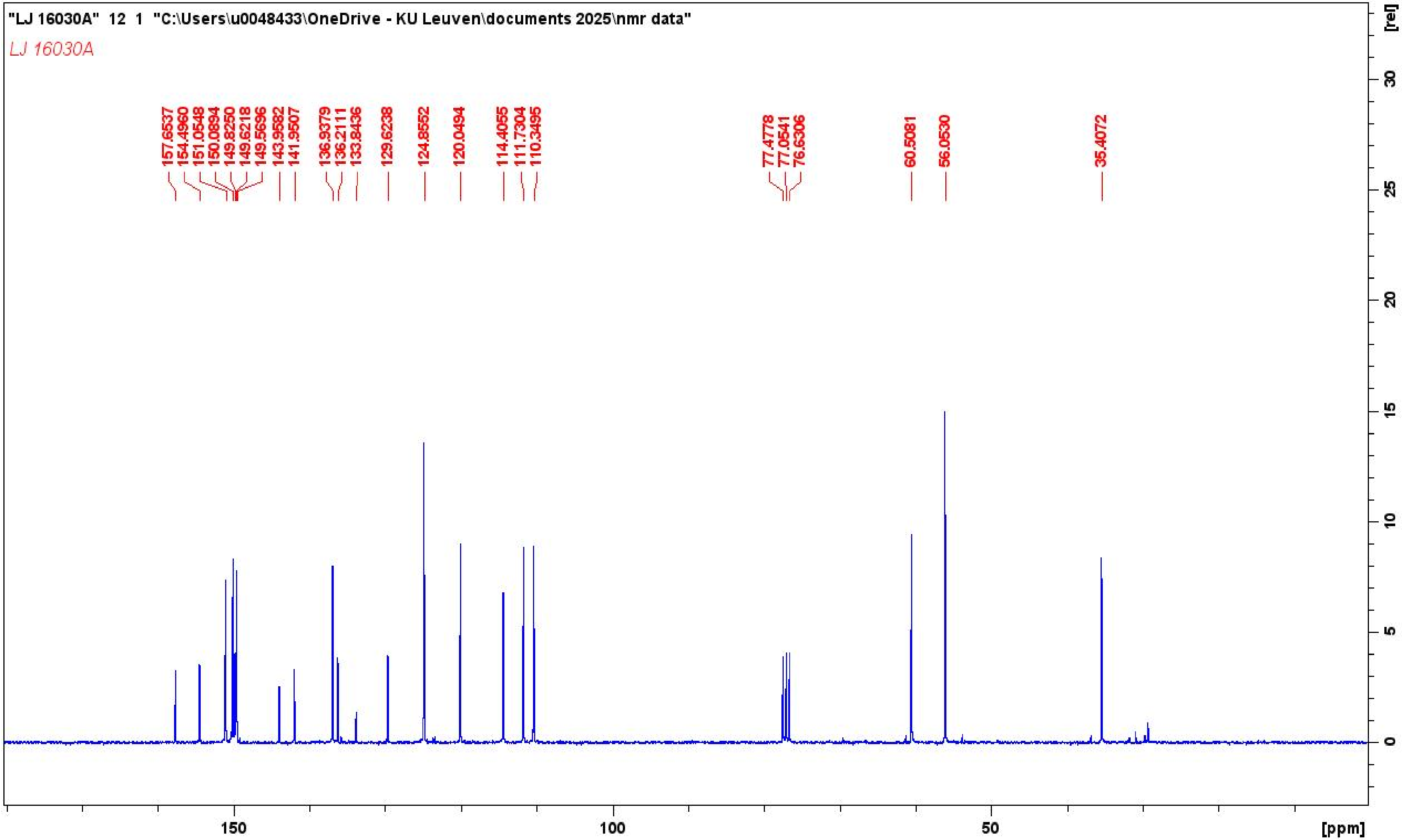

Supplement: Supplementary file 1 [file pharmaceuticals-18-01341-s001.zip › pharmaceuticals-3726359-supplementary.pdf]
